# A Quest for Effective ^19^F NMR Spectra Modeling: What Brings a Good Balance Between Accuracy and Computational Cost in Fluorine Chemical Shift Calculations?

**DOI:** 10.3390/ijms26146930

**Published:** 2025-07-18

**Authors:** Stepan A. Ukhanev, Yuriy Yu. Rusakov, Irina L. Rusakova

**Affiliations:** A. E. Favorsky Irkutsk Institute of Chemistry, Siberian Branch of the Russian Academy of Sciences, Favorsky St. 1, 664033 Irkutsk, Russia; stepa_ukhanev1998@bk.ru (S.A.U.); rusakov82@mail.ru (Y.Y.R.)

**Keywords:** ^19^F NMR, fluorine, shielding constant, chemical shift, basis set, LDBS, DFT, CCSD

## Abstract

This work proposes a systematic study of different computational schemes for fluorine Nuclear Magnetic Resonance (^19^F NMR) chemical shifts, with special emphasis placed on the basis set issue. This study encompasses two stages of calculation, namely, the development of the computational schemes for the geometry optimization of fluorine compounds and the NMR chemical shift calculations. In both stages, the performance of different density functional theory functionals is considered against the method of coupled-cluster singles and doubles (CCSD), with the latter representing a theoretical reference in this work. This exchange-correlation functional study is accompanied with a basis set study in both stages of calculation. Basis sets of different families, sizes, and valence-splitting levels are considered. Various locally dense basis sets (LDBSs) are proposed for the calculation of ^19^F NMR chemical shifts, and their performance is assessed by comparison of the calculated chemical shifts with both theoretical and experimental reference data. Overall, the pcS-3/pcS-2 LDBS scheme is recommended as the most balanced locally dense basis set scheme for fluorine chemical shift calculations.

## 1. Introduction

The incorporation of fluorine atoms into biologically active molecules is currently becoming an increasingly popular strategy for improving their bioactivity characteristics [1]. At least 25% of modern widespread drugs contain fluorine atoms [2], and nearly 300 fluorine-containing drugs have been officially approved for use as medicines [3]. Specifically, the high electron affinity of fluorine atoms leads to substantial changes in the electron density around protonated moieties such as carboxylic acid, alcohol, amine, etc., withdrawing the electron density from the protons. Due to the high electronegativity of the fluorine atom, powerful effects, consisting of, e.g., substantial changes in bond polarity, reactivity, and molecular geometry, are present in fluorine-containing compounds, determining, for the most part, their unique chemical properties and placing their utility in the production of pharmaceuticals, functionalized materials, and agrochemicals. In particular, approximately one-third of FDA-approved anti-HIV drugs are found to contain fluorine atoms in their structure [4]. Some fluorine compounds can manifest anti-inflammatory and anti-cancer activity, such as fluorinated benzofuran and its derivatives [5], while others may serve as scaffolds for antimalarial drugs [6], etc. Fluorine compounds have also found applications as soluble semiconductors [7,8,9,10], polymers [11,12,13], blue-light-emitting materials [14], liquid crystals [15], fungicidal agents [16], positron emission tomography (PET) tracers [17], etc.

In this respect, the synthesis of fluorine compounds usually involves ^19^F NMR spectroscopy, representing one of the most useful techniques for elucidating and confirming chemical structures of fluorine-containing compounds. Indeed, the fluorine nucleus has a single naturally occurring one-half spin isotope with 100% abundance. Overall, ^19^F NMR chemical shifts vary in the range of about 1300 ppm (with organic fluorine compounds having NMR signals in the diapason of approx. 400 ppm). In general, ^19^F NMR chemical shifts are known to be very sensitive to the chemical environment, so they present a great means of chemical structure study [18] and reaction mechanisms [19,20], involving both medium- and large-sized biological systems. The latter holds particular significance now, as modern fields of research are directed at structural and functional studies of proteins, nucleic acids, drug discovery, metabolic analysis, and in vivo tracking of bioactive molecules [21,22,23,24,25,26]. Moreover, due to the high sensitivity of fluorine chemical shifts to the chemical environment, the ^19^F signals allow us to distinguish enantiomers with chiral centers several carbons away from the binding site [26].

However, there are many cases when modern ^19^F NMR spectra of fluorine-containing compounds are difficult to interpret, especially when several fluorine atoms are present in a newly synthesized molecule, and their fluorine chemical shifts differ from each other by only a few ppm [27,28]. In this regard, high-quality quantum chemical calculations of the fluorine NMR chemical shifts may be of great assistance in order to properly interpret ^19^F NMR spectra.

Many efforts were made in the development of effective computational protocols for ^19^F NMR chemical shifts. Due to the very specific nature of the extremely electronegative fluorine atom, possessing as many as three lone pairs, computational protocols for fluorine chemical shifts are very difficult to work out, especially pertaining to the description of the electron correlation effects. Krivdin [29] presented a thorough review up to the year 2020 on the computational simulation of ^19^F NMR spectra. The review covered many computational protocols, but no particular approach was recommended as a ready-to-use, self-sufficient technique. This is not surprising because there is no consistency between multiple studies of computational protocols, especially among those regarding schemes based on the density functional theory (DFT) method [30].

Indeed, modern popular protocols rely on the DFT method, which allows us to deal with large molecules containing fluorine atoms at modest computational costs. In this respect, the accuracy of the DFT protocol is strongly determined by the exchange-correlation functional and one-electron basis set used. Different functional/basis set combinations lead to a substantially varying accuracy of calculated fluorine chemical shifts, which may deviate from experimental values by only 2–5 ppm for some combinations [31,32,33,34,35,36,37] and as much as 15–30 ppm for other combinations [37,38,39,40,41]. Based on the analysis of the calculations presented in the contemporary worldwide literature, we may assume that the BHandHLYP [42] and ωB97XD [43] functionals, as being applied in combination with rather large basis sets, can provide the accuracy characterized by errors of ca. 1–3 ppm for ^19^F chemical shifts against the experiment [31,32,33,34,36,37].

On the other hand, the basis set issue remains totally unclear. In various works, an acceptable accuracy was reached with basis sets of different types and quality. There were also some examples of apparent fortunate cancelation of method/basis set errors, when one of the least statistically suitable functionals has provided fine accuracy in combination with a small basis set of double-zeta quality [35]. Moreover, in some papers, it was found that extra diffuse functions markedly reduce the difference between the calculated and experimental fluorine chemical shifts [38], while in the others, the role of diffuse functions was rendered as negligible or even negative for the overall accuracy [33]. In this situation, it is reasonable to resort to as large as possible basis sets in combination with some of the verified functions, like the quadruple-zeta quality pcSseg-3 basis set [44] in combination with the BHandHLYP functional, as was recommended by Fedorov et al. [33]. However, the pcSseg-3 basis set is rather large in size, consisting of 97 contracted functions for the first-row elements, so for the systems of more than 100 non-hydrogen atoms, setting this basis set on all atoms rapidly becomes impractical. In the case of high-quality correlated approaches with systematic covering of electron correlation effects, such as the CCSD method [45], the calculations with the basis sets of quadruple-zeta quality are only affordable for small systems [46].

For now, it is indeed an open question as to how the accuracy of fluorine chemical shifts can be altered when using different LDBSs [47]. This approach prescribes higher-quality basis sets for the atoms of importance (the atoms that can be considered as representing a special interest from the perspective of a particular quantum chemical problem) and lower-quality basis sets for the unimportant ones. The LDBS schemes represent a natural gradual improvement in accuracy, going from smaller basis sets to larger basis sets on account of the increasing computational costs. To our knowledge, there are no systematic studies on the LDBS schemes presented for the calculation of fluorine chemical shifts so far.

This work embodies a methodological study aimed at the development of an effective computational protocol for ^19^F NMR chemical shifts with special emphasis placed on the basis set issue. Two stages of calculation were considered, namely, the geometry optimization of fluorine compounds and the NMR chemical shift calculation. In both stages, the performance of different density functional theory functionals was considered against the CCSD method, with the latter representing the theoretical standard in this work.

The study of the performance of different DFT exchange-correlation functionals for fluorine chemical shifts is in accordance with many methodological works, though the use of the CCSD method as the reference method is completely different from what was previously carried out. Namely, in practically all papers presenting DFT calculations of ^19^F NMR chemical shifts, the performance of the DFT functionals was judged based on comparisons with the experiments. As a matter of fact, the latter requires concurrent calculations of vibrational, solvent, and relativistic (in the case of the presence of heavy atoms [48]) corrections. These factors usually make their own contributions to final accuracy, and if they are not properly taken into account or not considered at all, the comparison with the experiment becomes obscure. In the majority of papers that deal with the assessment of functionals in relation to experimental data, omitting at least the vibrational contributions is usually the case.

The exchange-correlation functional study was accompanied by a basis set study at both stages of calculation. Basis sets of different families, sizes, and valence-splitting levels were considered. Various LDBS schemes were proposed for the calculation of ^19^F NMR chemical shifts, and their performance has been studied in terms of theoretical accuracy. The theoretically established hierarchy of the considered LDBS schemes was corroborated with the comparison of the theoretical data with the gas-phase experiment. For this comparison, CCSD calculations of fluorine chemical shifts have been carried out while taking into account vibrational effects.

## 2. Results and Discussion

### 2.1. Studying the Performance of Different Exchange-Correlation Functionals and One-Electron Basis Sets in the Calculation of Equilibrium Geometry Parameters for Fluorine Compounds Within the DFT Method

**Figure 1:**
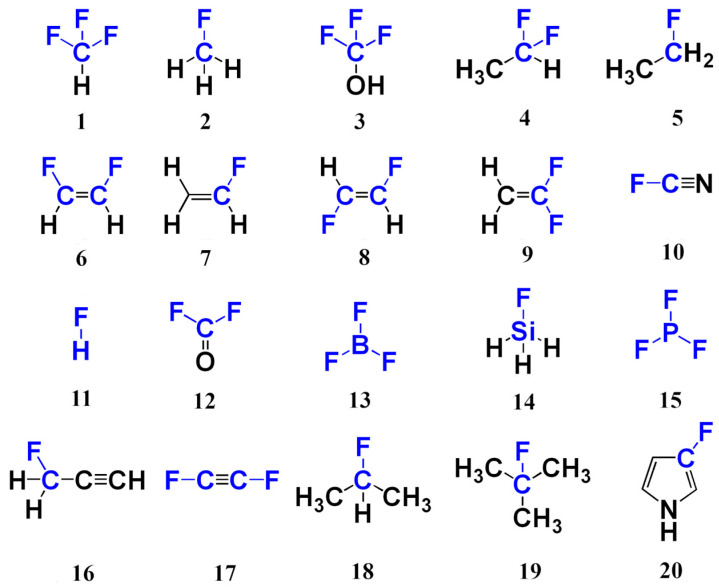
Compounds of set 1.

We started our methodological study with the investigation of the performance of different DFT functionals in the geometry optimization routine. Indeed, equilibrium geometry represents a starting point for the calculation of any molecular property. Inaccuracy in the equilibrium geometry inevitably rubs off on the molecular properties calculated from it. This is usually referred to as the geometry factor effect [49,50], and, if not carefully taken into account, it can result in misleading results. Thus, our first goal was to choose the DFT exchange-correlation functional that would give equilibrium geometries in the closest vicinity to the geometries calculated at the CCSD level. For that purpose, we chose twenty representative fluorine compounds (set 1), as shown in Figure 1. These molecules are rather small in size and mostly symmetric, which makes them a convenient set for multiple DFT calculations, as well as for computer-resource-demanding CCSD calculations. They possess rather versatile electronic structures. In this way, set 1 combines representativity and computational convenience.

As a primary indicator of DFT functional suitability for geometry optimization, we chose the averaged relative absolute differences between the lengths of fluorine-involving bonds, F-X (X = H, B, C, Si, P), in twenty molecules of set 1 (*N* = 20), calculated using the DFT method with different functionals ($l_{i,DFT}$), and the corresponding CCSD values ($l_{i,CCSD}$), expressed as the mean absolute percentage errors (MAPEs). These MAPEs were calculated through Equation (1) as follows:

(1) $$MAPE=\frac{1}{N}\sum_{i=1}^{N}\left|l_{i,CCSD}-l_{i,DFT}\right|/l_{i,CCSD}\times 100\%$$

One may ask why it was the F-X bond lengths that were chosen as the monitoring parameters and not the bond angles at fluorine in their stead, or both of them? We consider geometry optimization as an integral part of the computational protocol for fluorine chemical shifts; thus, it does matter what type of geometrical parameters affect the ^19^F NMR chemical shifts the most. In the case of fluorine compounds **1**–**20**, the alternation of the functional at the geometry optimization stage leads to changes in the lengths of fluorine-related bonds in thousands and even in hundreds of Å, while the bond angles vary only by a few tenths of an arc degree at best. As was shown in one of our previous papers [49], the former has a noticeable effect on the NMR chemical shift in the nucleus of interest, while the latter has practically vanishing influence.

The multitude of considered functionals comprises twenty functionals that can be used for geometry optimization problems, namely, the local spin density approximation (LSDA) with Slater, Vosko, Wilk, and Nusair correlation energy density—the SVWN [51,52,53,54]; the pure generalized gradient approximation (GGA) functionals: Becke 1988 exchange functional and the Perdew 86 correlation functional—the BP86 [55,56], Perdew–Burke–Ernzerhof functional—the PBE [57], Perdew–Wang functional—the PW91 [58]; the hybrid GGA functionals: half-and-half functionals—the BHandH [59] and BHandHLYP [42], hybrid functional on the basis of the pure tHCTH functional—the tHCTHhyb [60], Becke’s three-parameter Lee–Yang–Parr hybrid functional—the B3LYP [59,61], extended hybrid functional combined with the Lee–Yang–Parr correlation functional—the X3LYP [62], the three-parameter hybrid functional with the optimized exchange functional OPTX—the O3LYP [63], Becke’s re-parameterized B97 [64] functionals fitted to conventional thermochemical data—the B97-1 [65] and B97-2 [66], the modified functional of Perdew and Wang by Adamo and Barone—the mPW3PBE [57,67], the one-parameter hybrid Perdew–Burke–Ernzerhof functional—the PBE0 [68,69,70]; the range-separated hybrid GGA functionals: the revised range-separated Heyd–Scuseria–Ernzerhof functional—the HSE06 [71], the long-range corrected B3LYP functional—the CAM-B3LYP [72], the range-separated version of Becke’s 97 functional with additional dispersion correction—the ωB97-XD [43]; and the hybrid meta-GGA functionals: the Minnesota functionals of Zhao and Truhlar group—the M06-HF [73] and M062X [74], and the hybrid functional based on the Tao–Perdew–Staroverov–Scuseria TPSS functional [75]—the TPSSh [76,77].

The quality of the resulting equilibrium geometries is also very sensitive to the basis set used [49,50,78,79,80,81]. In particular, Helgaker et al. [78] and Rusakov et al. [49] have established an interesting correlation that bond lengths are noticeably contracted by an increase in the basis set size used in the geometry optimization procedure. Thus, it is of the utmost importance to exclude the basis set factor as a possible source of error at the stage of testing the performance of DFT functionals in the geometry optimization routine. So, we chose the pc-3 basis set, which is so large in size that it cannot be considered a contingent factor, interfering with the analysis of the DFT functionals. Indeed, this basis set represents nearly a quadruple-zeta quality basis set ([5s4p2d1f] for H, [6s5p4d2f1g] for the second period, and [6s5p4d2f1g] for the third period). All calculated values of equilibrium bond lengths of compounds **1**–**20** obtained within the CCSD and DFT level of theory with different exchange-correlation functionals using the pc-3 basis set are presented in Table S1 of Supplementary Materials.

**Figure 2:**
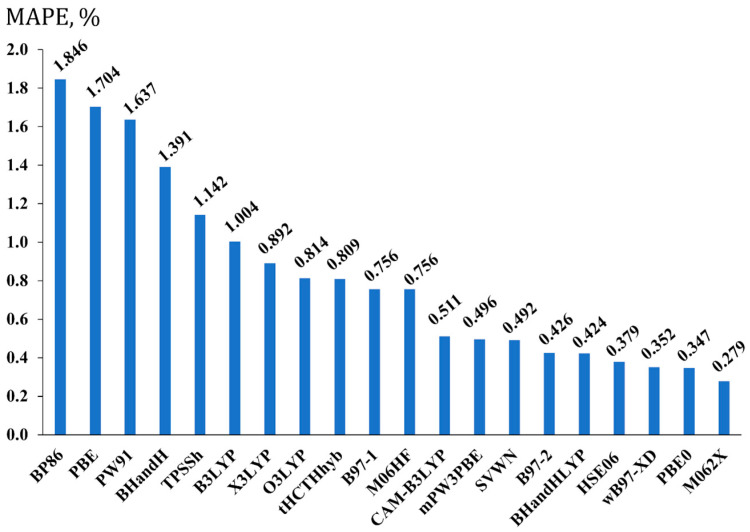
The performance of twenty exchange-correlation functionals in the geometry optimization stage, shown on the example of the mean absolute percentage errors (MAPEs, in %) for F-X (X = H, B, C, Si, P) bond lengths of set 1, calculated at the DFT level of theory with different functionals and the pc-3 basis set, against the corresponding bond lengths calculated at the CCSD/pc-3 level of theory.

The calculated MAPEs are present in Figure 2.

As shown in Figure 2, four functionals give bond lengths deviating from the CCSD values with MAPEs less than 0.4%. Interestingly, the three of them are the long-range attuned functionals, namely, HSE06, ωB97-XD, and M062X, with the latter being the best. The M062X functional was mentioned above as the hybrid meta-GGA functional, but it also bears a portion of Grimme’s long-range dispersion correction with the s6 scaling factor of 0.06 [82]. Evidently, the electronic structures of molecules bearing π-systems in the presence of such strong electron-withdrawing elements like fluorine must be described better if long-range dispersion correction is included. The π-systems constitute roughly half of set 1, and they can easily be traced to be the most responsible for the observed behavior of the long-range attuned functionals shown in Figure 2, with the M062X functional being the best of them. It is interesting to note that Tarakeshwar et al. also noted that fluorine substituents have a strong effect on the electronic structure of π-systems such as aromatic rings, and dispersion interactions are very important for such systems [83].

As shown in Figure 2, apart from the three best long-range attuned functionals, the other best functional is PBE0, which takes second place after the M062X functional. In actuality, this functional was expected to be among the best ones, because its good performance for the equilibrium geometry parameters in the context of various NMR calculations was already observed in our previous studies [49,50].

In any case, out of all considered functionals, it is the M062X functional that demonstrated the best performance relative to the CCSD results. Thus, we recommend using the M062X functional for the optimization of structures of fluorine compounds, and all further discussions presented in this work will rely on the equilibrium geometries of the DFT(M062X) level.

It is also worth mentioning that pure GGA functionals, BP86, PBE, and PW91, turned out to be the worst ones for the geometry optimization of fluorine compounds **1**–**20**. Their MAPEs exceeded 1.6%. This indicates the fact that the incorporation of a portion of exact exchange from the Hartree–Fock theory is very important for the correct description of the electron structure of fluorine compounds in the geometry optimization routine. So, we do not recommend using pure GGA functionals in the geometry optimization of molecules that bear fluorine atoms. At the same time, a MAPE of only 0.492% for the SVWN functional came as a surprise, because this places the SVWN in alignment with functionals with good performance, notwithstanding the fact that the SVWN functional embodies the lowest rung approximation of Jacob’s ladder [84]. Even more so, in many cases, it performs worse than other DFT functionals in the geometry optimization of different systems [85,86]. This time, however, the SVWN functional has proven otherwise.

With the best exchange-correlation functional chosen, we move to the issue of the performance of different basis sets for optimizing the geometry parameters of fluorine compounds within the DFT method.

In this study, the measure of the accuracy of the basis set in question is the MAPE for the lengths of F-X bonds in the compounds of set 1, calculated within the DFT(M062X) method with the given basis set, evaluated against the corresponding bond lengths obtained with the aug-pc-4 basis set. These MAPEs are given by Equation (2):

(2) $$MAPE=\frac{1}{N}\sum_{i=1}^{N}\left|l_{i,aug\text{-}pc\text{-}4}-l_{i,basis\;set}\right|/l_{i,aug\text{-}pc\text{-}4}\times 100\%$$

Four families of basis sets were considered: Dunning’s correlation consistent polarized valence basis sets of double-, triple-, quadruple-, quintuple-, and sextuple-zeta quality with and without the augmentation with diffuse functions (indicated by adding the prefix “aug-” to the acronym), the (aug-)cc-pVXZ (X = D, T, Q, 5) [87,88,89], and cc-pV6Z [90,91,92]; Pople’s split-valence basis sets of double- and triple-zeta quality without and with the augmentation with one set of diffuse functions (denoted as prefix “++” before letter G) and with different number of polarization functions (following the letter G in parentheses), the 6-31(++)G(x,y) [93,94,95,96,97,98,99,100], and 6-311(++)G(x,y) [95,99,100,101,102,103], respectively; Jensen’s polarization consistent basis sets, (aug-)pc-*n* (*n* = 1–4) [104,105,106,107,108]; and Rusakov’s geometry-oriented pecG-*n* (*n* = 1, 2) basis sets [49,50,109,110]. All these basis sets have their peculiarities and different rates of convergence towards the complete basis set (CBS) limit with the increase in their cardinal numbers (the split valence levels). As has been already mentioned, the aug-pc-4 basis set was chosen to represent the reference basis set, because it is of quintuple-zeta quality and very large in size: [8s7p4d3f2g]—hydrogen (88 contracted spherical functions); [9s8p7d4f3g2h]—the first-row elements (145 contracted spherical functions); [8s7p7d4f3g2h]—the second-row elements (141 contracted spherical functions). In addition, Jensen’s series was optimized for the DFT-type calculations, and the (aug-)pc-*n* basis sets are capable of quickly and systematically approaching the CBS limit within the DFT method [111]. Thus, in the present study, the aug-pc-4 basis set represents a top-quality basis set that can be regarded as the basis set that provides the results in very close proximity to the CBS limit. All values of equilibrium bond lengths of molecules **1**–**20** obtained within the DFT(M062X) method with different basis sets are presented in Table S2 of Supplementary Materials. 

**Figure 3:**
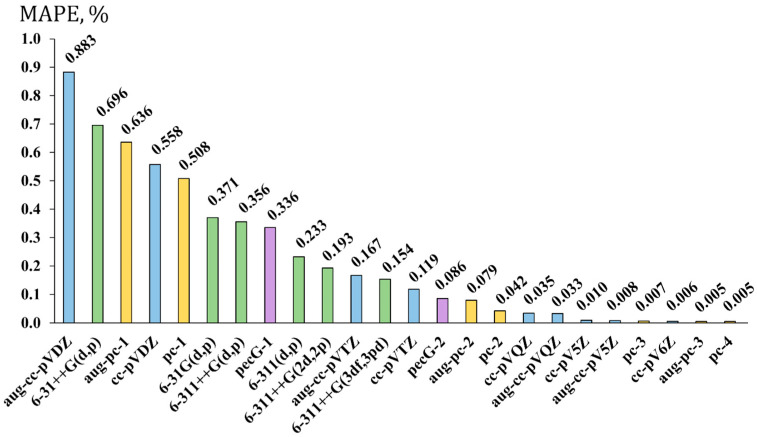
The performance of twenty-four basis sets for the geometry optimization of fluorine compounds carried out within the DFT (M062X) method demonstrated on the example of the mean absolute percentage errors (MAPEs, in %) for the F-X (X = B, C, Si, P) bond lengths in molecules of set 1 against the corresponding values obtained with the aug-pc-4 basis set using the same functional.

The resulting MAPEs for the bond lengths of molecules **1**–**20** are presented in Figure 3. Different families of basis sets are colored differently: Dunning’s, Pople’s, Jensen’s, and Rusakov’s series are shown with blue, green, yellow, and violet bars, respectively.

As shown in Figure 3, the considered basis sets can be roughly divided into three groups in terms of accuracy. The first group comprises the basis sets that provide the worst accuracy for the F-X bond lengths (MAPE > 0.5%). These are mostly augmented and non-augmented double-zeta quality basis sets of Dunning and Jensen, with one Pople’s augmented basis set of double-zeta quality. The second group can be said to give mediocre accuracy, with an MAPE between 0.1 and 0.5%. This group mostly consists of the augmented and non-augmented triple-zeta quality basis sets of Dunning and Pople (with the only exception of the double-zeta quality 6-31G(d,p) basis set, for which the MAPE is 0.371%), and the first-level Rusakov’s basis set, the pecG-1 (approx. of double-zeta quality). The third group includes the basis sets providing a supreme accuracy, characterized by MAPE < 0.1%. As can be seen from Figure 3, the basis sets constituting this group are Rusakov’s second-level pecG-2 basis set (approx. of triple-zeta quality), Jensen’s and Dunning’s basis sets of triple- and quadruple-zeta quality, respectively, and so on.

From the general MAPE distribution shown in Figure 3, one can conclude that the level of valence splitting is the main factor governing the accuracy of F-X bond lengths. However, this is not the factor that can be considered without referring to the family of basis sets. Indeed, the convergence behavior of different families of basis sets towards the ideal theoretical geometry is different; for example, the third group of supreme accuracy starts from the triple-zeta basis sets of Rusakov and Jensen and quadruple-zeta quality of Dunning’s series. This speaks in favor of a rather faster convergence of the former two series compared to the latter one. Another example is the comparison of the pecG-2 basis set with the 6-311++G(3df,3pd) basis set. Based on the size of the pecG-2 basis set (i.e., 35 functions for the first-row elements), it is at the same triple-zeta quality level as the 6-311++G(3df,3pd) basis set, though the latter is even larger due to the additional diffuse functions. At the same time, the pecG-2 basis set provides the results with an MAPE of only 0.086% (the supreme accuracy group), while the 6-311++G(3df,3pd) basis set gives an MAPE of as much as 0.154% (the mediocre accuracy group).

Other nuances are also important. In particular, additional polarization functions apparently improve the accuracy of equilibrium geometries of fluorine compounds. This can be directly observed in Figure 3 with the example of the gradual decrease in MAPE going from 6-311++G(d,p) to 6-311++G(2d,2p) to 6-311++G(3df,3pd). In general, this is not an unexpected finding, because there are some exemplary papers that demonstrate the importance of additional polarization functions for the equilibrium geometry of the systems with atoms possessing lone electron pairs [112].

Another interesting issue is the role of extra diffuse functions. There is a widespread opinion that the addition of diffuse functions to the basis sets used on the atoms possessing lone electron pairs should give a better description of these lone pairs as they reach out far from the nucleus. In addition, it was found that including the diffuse functions in such cases is needed not only for the atoms bearing lone electron pairs, but also for the rest of molecule, in order to describe the delocalization of the lone pair properly and improve, in that way, the description of the electron system in a whole [113]. The actual situation for the bond lengths of fluorine compounds from set 1 turns out to be a bit more divergent from this statement. As one can see from Figure 3, Dunning’s, Pople’s, and Jensen’s basis sets of double- and triple-zeta quality demonstrate the worsening of the accuracy of bond lengths upon the extension of the basis sets with diffuse functions. At the same time, going beyond the triple-zeta quality makes no difference in the accuracy provided by augmented and non-augmented basis sets, while substantially increasing the computational costs. Thus, based on the presented observations, we abstain from recommending the augmented basis sets for the geometry optimization of fluorine compounds for now.

Concluding this subsection, we recommend using the M062X exchange-correlation functional for the optimization of geometry parameters of fluorine compounds within the DFT method, as it provides the best accuracy relative to the CCSD results. At that, the pecG-2 or pc-2 basis sets represent the best-balanced choice, because they are moderate in size and provide the accuracy somewhere in between the accuracy of the cc-pVTZ and cc-pVQZ basis sets. Extra polarization functions lead to an improvement in the accuracy of equilibrium geometries, while extra diffuse functions lead to deteriorations in accuracy or no effect at all. At that, adding extra diffuse functions results in substantial increase in computational costs. All additional results were obtained using the DFT (M062X) equilibrium geometries obtained with the pc-3 basis set in order to completely negate the geometry factor error.

### 2.2. Studying the Performance of Different Exchange-Correlation DFT Functionals and One-Electron Basis Sets in the Calculation of ^19^F NMR Chemical Shifts

In this section, we assess the performance of different DFT functionals applied to the calculation of ^19^F NMR chemical shifts in molecules in set 1 against the corresponding values obtained at the CCSD level of theory. In the context of chemical shifts, we have considered fourteen functionals that are applied more often than not to the calculations of fluorine shielding constants nowadays. These are the SVWN, PW91, PBE, B3LYP, X3LYP, O3LYP, B97-2, PBE0, BHandH, BHandHLYP, TPSSh, ωB97-XD, CAM-B3LYP, and M062X. In this study, all fluorine shielding constants were calculated using the shielding-oriented specialized basis set pcS-3 of quadruple-zeta quality. This basis set is large enough to guarantee a vanishingly small influence of the basis set factor on the building of the DFT models hierarchy. All values of ^19^F NMR shielding constants of molecules **1**–**20** calculated at the CCSD/pcS-3 level are given in Table S5 of Supplementary Materials, while the corresponding values obtained within the DFT method in combination with various exchange-correlation functionals are given in Table S3 of Supplementary Materials.

The scaled DFT NMR chemical shifts (${\tilde{\delta }}_{DFT,i}$) corresponding to the calculated shielding constants ($\sigma_{DFT,i}$) were evaluated from the linear regression model with the slope equal to −1, which was derived from the mapping of the shielding constants calculated using different functionals within the DFT method onto the chemical shifts obtained from the CCSD calculations ($\delta_{CCSD,i}$), which represent the ideal theoretical values in this study. The CCSD chemical shifts were evaluated as the difference between shielding constants of the chosen NMR standard and the compound under question (simplified IUPAC expression [114,115]). For linear regression analysis, it does not matter what compound is chosen as the standard, because statistical descriptors such as the MAE or MAPE calculated from the two sets of data (one set is the scaled DFT data under testing, and the other set is the ideal CCSD theoretical data) would not change their values, should one choose another NMR standard. In this test, we chose molecule **2** of set 1 (see Figure 1) to represent the NMR standard. The scaled chemical shifts were calculated in accordance with the least squares method (LSM). Overall, this approach represents a mathematical procedure for finding the best-fitting mapping of a given set of points on the other set of points by minimizing the sum of the squares (*S*) of the offsets (“the residuals”) of the points from the line with slope −1. Within this model, the mapping rule is given by Equation (3) as follows:

(3) $${\tilde{\delta }}_{DFT,i}=-\sigma_{DFT,i}+\alpha$$

In Equation (3), the coefficient α is determined from the minimization of the sum of the squares of the residuals. For the exact expression of α, see Equation (4):

(4) $$S=\sum_{i=1}^{N}{\left(\delta_{CCSD,i}-{\tilde{\delta }}_{DFT,i}\right)}^{2}\to min,\;\alpha =\frac{1}{N}\sum_{i=1}^{N}\left(\delta_{CCSD,i}+\sigma_{DFT,i}\right)$$

In these formulas, the coefficient α represents an approximated shielding constant of a standard for each functional, while *N* = 20 is the number of calculated fluorine shielding constants. The linear model in the form of Equation (3) is a physically motivated model due to the similarity in its mathematical structure to the simplified IUPAC expression. It also allows us to avoid any systematic errors between the two sets of data. In our case, this eliminates systematic errors in the results obtained with the DFT and CCSD methods. In general, the LSM has proven itself as a reliable mapping method in application to the calculation of NMR chemical shifts with different computational schemes [36,49,116,117,118].

For each set of calculated scaled DFT chemical shifts, the mean absolute errors (MAEs) were calculated against the CCSD values in accordance with Equation (5):

(5) $$MAE=\frac{1}{N}\sum_{i=1}^{N}\left|{\tilde{\delta }}_{DFT,i}-\delta_{CCSD,i}\right|$$

**Figure 4:**
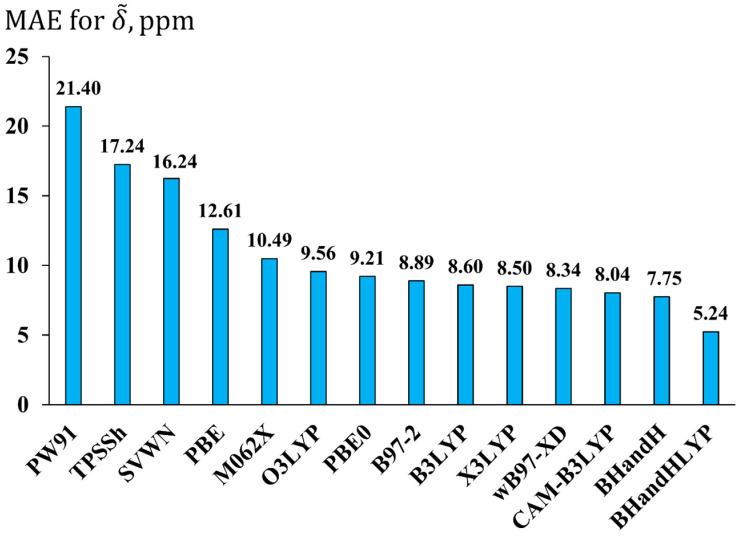
The performance of fourteen exchange-correlation functionals in the calculations of fluorine NMR chemical shifts exemplified by the mean absolute errors (MAEs, in ppm) for ^19^F NMR scaled chemical shifts (${\tilde{\delta }}_{DFT,i}$) in molecules of set 1 obtained using the DFT method with different functionals and pcS-3 basis set against the corresponding values of the CCSD/pcS-3 level of theory.

The evaluated MAE for each DFT functional is presented in Figure 4. As seen in this figure, the best performing functional is BHandHLYP with the MAE of 5.24 ppm. In terms of accuracy, the BHandHLYP functional is followed by a series of functionals with MAEs between 7 and 10 ppm. These are mostly hybrid GGA functionals, with two of them being long-range corrected (ωB97-XD and CAM-B3LYP). As to the least suitable functionals (with MAEs > 10 ppm), these comprise the simplest local SVWN functional and pure functionals such as PW91 and PBE. The hybrid meta-GGA TPSSh also turned out to be among the worst functionals (MAE = 17.24 ppm), repeating its poor performance shown at the geometry optimization stage.

The M062X functional was in fifth place from the left, showing the MAE of as much as 10.49 ppm. Roughly speaking, this functional is at the threshold between the functionals that provide the results of the lowest and mediocre accuracy of the calculated ^19^F NMR chemical shifts. So, notwithstanding its prominent performance for the geometry optimization of fluorine compounds, its usefulness for fluorine chemical shift calculations turned out to be doubtful; hence, it cannot be recommended for the NMR calculation stage. The functional that can be recommended indeed is the BHandHLYP. This is an interesting but not entirely surprising result. Based on the comparison of theoretical data with the experiment, presented in many literature sources, one can easily identify the BHandHLYP functional as representing one of the most suitable functionals for ^19^F NMR chemical shifts [31,32,33,37]. This time, we have corroborated this finding within the theoretical analysis, based on comparison with the superior CCSD method.

The following issue, which is to be studied, pertains to the performance of different basis sets in ^19^F NMR calculations. In contrast to multiple DFT functional studies presented in contemporary literature, the basis set issue for ^19^F NMR calculations has been studied scarcely. For this investigation, we have used the same pool of molecules (set 1, as shown in Figure 1) as before and all the facts established in this work so far. Namely, the equilibrium geometries for set 1 were obtained with the DFT (M062X) method using the pc-3 basis set, while fluorine shielding constants were calculated using the BHandHLYP functional with varying basis sets.

The collection of considered basis sets in this study is practically the same as that which has been taken under scrutiny in the optimized bond length analysis, though some alterations are present. Namely, this time, only three groups of basis sets are taken into account: Dunning’s, Jensen’s, and Pople’s. The property energy consistent (PEC) group of basis sets is not considered here, because the specialized NMR-oriented pecS-*n* (*n* = 1, 2) basis sets exist only for H, C, N, O, and P atoms [116,119,120,121,122], but for the fluorine atom, they have yet to be generated. On the other hand, the geometry-oriented pecG-*n* basis sets are not suitable for NMR calculations, so it is not a fair point to take them into consideration in this study. Jensen’s series was extended by the NMR-oriented group of basis sets, the (aug-)pcS-*n* (*n* = 1–4), for the elements of 1–3 periods (H–Ar) [123]. The top-quality quintuple-zeta augmented basis set of this kind, the aug-pcS-4, has been chosen as the reference basis set, which provides ideal theoretical values. This basis set has the structure [9s,11p,7d,4f,3g,2h] for the second-period atoms and [8s,7p,4d,3f,2g] for the hydrogen atom. Thus, its total size is 154 and 88 functions for the second-period atoms and hydrogen, respectively. The aug-pcS-4 represents a very large polarization-consistent basis set of quintuple-zeta quality extended by extra diffuse functions. By virtue of its size and specification, this basis set ought to provide results in very close proximity to the theoretical CBS limits for fluorine NMR shielding constants of all considered molecules. In particular, Rzepiela et al. [124] have recently demonstrated this fact on the example of ^31^P shielding constant of the PH_3_ molecule, where Jensen’s shielding-oriented augmented basis set of quintuple-zeta quality has been shown to give the result deviating from the CBS limit by only 0.04% (corresponding to tenths of a ppm at the full value of ca. 557 ppm). All calculated ^19^F NMR shielding constants of molecules **1**–**20** calculated within the DFT(BHandHLYP) method with different basis sets are given in Table S4 of Supplementary Materials.

**Figure 5:**
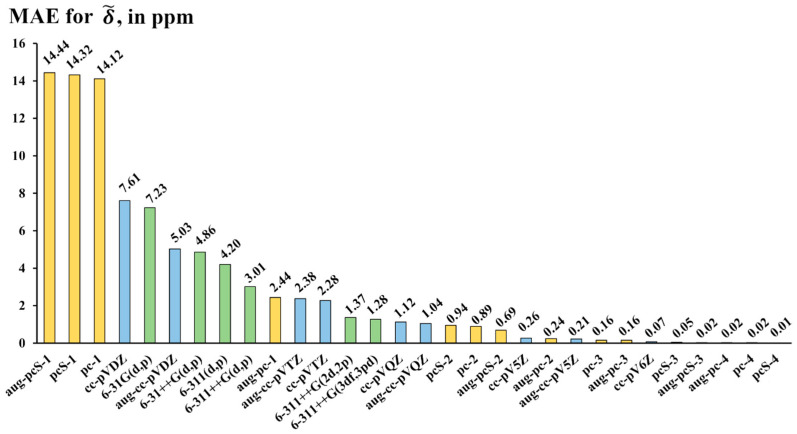
The performance of thirty basis sets of different families (Dunning’s—blue bars, Pople’s—green bars, and Jensen’s—yellow bars) against the aug-pcS-4 basis set exemplified on the mean absolute errors (MAEs, in ppm) for ^19^F NMR scaled chemical shifts ($\tilde{\delta }$) of molecules of set 1 calculated using the DFT (BHandHLYP) method and different basis sets, with the aug-pcS-4 values used as the theoretical reference data.

For each basis set, the MAEs were calculated in accordance with the scheme outlined above (see Equations (3)–(5)), though the ideal ($\delta_{CCSD,i}$) and scaled (${\tilde{\delta }}_{DFT,i}$) values in these formulae should be replaced with $\delta_{aug\text{-}pcS\text{-}4,i}$ and ${\tilde{\delta }}_{bs,i}$, respectively, where “bs” designates the “basis set under study.” The calculated MAEs are presented in Figure 5. Based on the accuracy diagram shown in Figure 5, one can distinguish four categories of basis sets, namely, those providing supreme accuracy with MAE less than 1 ppm (category 1), good accuracy with MAE from 1 to 3 ppm (category 2), mediocre accuracy with MAE from 3 to 8 ppm (category 3), and poor accuracy with MAE of more than 14 ppm (category 4).

Let us start with the discussion of the left-hand side of the diagram in Figure 5, i.e., category 4. This category includes three Jensen’s basis sets of the lowest double-zeta quality, namely, the specialized NMR-oriented (aug-)pcS-1 basis sets and the usual energy-optimized pc-1 basis set. These basis sets demonstrate the worst accuracy, and the problem, apparently, is not in their small sizes. For example, the pc-1 basis set is equal to the cc-pVDZ basis set in structure and size but has twice as large MAE as that of the pc-1 basis set. These three cannot be recommended for the calculation of ^19^F NMR chemical shifts in various fluorine compounds.

Category 3 includes mostly the augmented and nonaugmented Pople’s basis sets of double- and triple-zeta quality with only one set of d-polarization functions for non-hydrogen atoms and one set of p-polarization functions for hydrogen atoms. Dunning’s basis sets of the double-zeta quality, the (aug-)cc-pVDZ, possessing the same number of polarization functions as Pople’s basis sets of category 3, also belong in this category. The addition of more polarization functions, especially to the first polarization shell, noticeably improves accuracy, transferring these basis sets to the second category and beyond. This correlates with our previous findings, made for the NMR chemical shifts in various nuclei, that the polarization functions, especially those of the first polarization shell, are of utmost importance for the NMR calculations [116,121,125].

The basis sets of the second category provide good accuracy with the MAEs between 1 and 3 ppm relative to the aug-pcS-4 basis sets. This category is an interesting one because it allows us to achieve rather precise results; however, not all basis sets of this category are as efficient as one would desire. Upon careful examination of Figure 5, one can notice that roughly half of the basis sets in this category are Dunning’s basis sets of triple- and quadruple-zeta quality, (aug-)cc-pVXZ (X = T, Q). The rest of the category includes augmented Pople’s basis sets of triple-zeta quality with enlarged polarization space and Jensen’s double-zeta quality aug-pc-1 basis set. The best choice of category 2 would be the 6-311++G(3df,3pd) basis set. Indeed, although the 6-311++G(3df,3pd) basis set is slightly inferior to the (aug-)cc-pVQZ basis sets in accuracy (which are the best ones in category 2), its size is significantly smaller (approx. 1.5 and 2 times, respectively). In terms of size, the 6-311++G(3df,3pd) basis set represents the median of category 2.

The most interesting category is category 1. The basis sets of this category provide outstanding results deviating from ideal theoretical data by no more than 1 ppm. As one can see from Figure 5, this category almost entirely consists of Jensen’s basis sets starting from the triple-zeta quality and beyond ((aug-)pc(S)-*n*, *n* = 2, 3, 4) and a speck of Dunning’s basis sets of the topmost hierarchy ((aug-)cc-pV5Z and cc-pV6Z). The sizes of basis sets in category 1 vary in range between 30 functions, for the pc-2 basis set, and 145 functions, for the aug-pc-4 basis set (the sizes are given for the first-row elements). The basis sets that are located in the lower-half part of the size scale, with sizes varying from 30 to 90 functions, are of the topmost practical interest. Out of these seven lower-size-part basis sets, the pcS-3 basis set (consisting of 73 functions) has the smallest MAE of only 0.05 ppm. Such a small absolute average deviation from the ideal theoretical values makes the pcS-3 practically in par with the aug-pcS-4 basis set (with a size of as many as 154 functions). Thus, out of all basis sets of category 1, we recommend the pcS-3 basis set as the most balanced in terms of the trade-off between the accuracy and computational costs for the calculations of fluorine chemical shifts with supreme accuracy.

Looking once more at Figure 5, one can also notice that the augmentation with diffuse functions improves accuracy, especially in categories 3 and 4. This is coherent with our previous findings about the NMR chemical shifts calculations of other elements [122].

At that point, we move on to the investigation of different locally dense basis set (LDBS) schemes. As was mentioned in the introductory section, this approach aims to gradually improve the accuracy of the results by means of step-wise heightening the basis set quality for the atoms of special importance for a given quantum-chemical problem. Our consideration is based on four LDBS schemes and two full basis set schemes built on Jensen’s basis sets: (a) pcS-2/pcS-1; (b) pcS-3/pcS-1; (c) pcS-2; (d) pcS-3/pcS-2; (e) pcS-3; and (f) pcS-4/pcS-3. In these notations, the basis set that goes before the slash designates the basis set that is set on fluorine atoms, and the basis set that goes after the slash designates the basis set used on the rest of the atoms. In this study, we have used molecules from set 1 (see Figure 1) and two different methods of electron theory, the DFT (BHandHLYP) and CCSD. The ideal theoretical values for both methods were calculated using the pcS-4/pcS-3 LDBS scheme, which apparently must give the ideal values close to the CBS limit, as can be deduced from Figure 5. All calculated fluorine shielding constants obtained at the CCSD and DFT(BHandHLYP) levels with different basis set schemes are presented in Tables S5 and S6 of Supplementary Materials, respectively.

**Figure 6:**
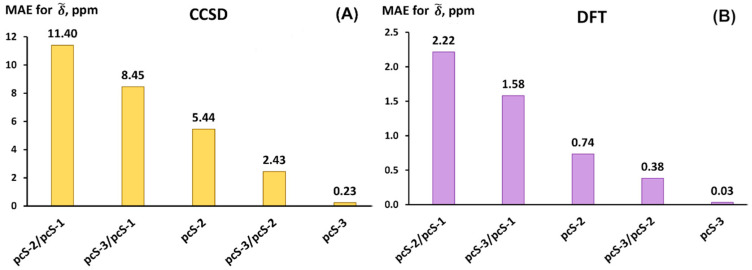
The performance of different basis set schemes against the pcS-4/pcS-3 results exemplified on the MAEs for ^19^F scaled chemical shifts ($\tilde{\delta }$) of molecules of set 1 calculated using the (**A**) CCSD and (**B**) DFT (BHandHLYP) method.

The MAEs for each basis set scheme were calculated against the reference theoretical values in accordance with standard formulae introduced above (Equations (3)–(5)). Thus, the performance of different basis set schemes against the pcS-4/pcS-3 scheme is shown in Figure 6.

It can be seen from Figure 6 that the basis set schemes for both CCSD and DFT calculations are arranged in the same order in terms of accuracy, though the diapason of MAEs in the case of the CCSD method is wider than that for the DFT method. In other words, the CCSD method is much more sensitive to the basis sets used. This is not unexpected, because, as is widely known, the convergence of the results on the basis set in the CCSD calculations is significantly slower compared to that within the DFT method [111]. Thus, the starting scheme pcS-2/pcS-1 with an MAE of about 11.4 ppm for the CCSD method is apparently very far from the threshold of convergence for this method, while for the DFT method, the situation is more favorable.

As one can see from Figure 6, one achieves a considerable improvement in accuracy when increasing the quality of the basis set used only on the fluorine atoms. Based on the diagrams in Figure 6, we can say that the most commendable scheme is the pcS-3/pcS-2 which gives an MAE of only 2.43 ppm for the CCSD method and 0.38 ppm for the DFT method. This is as much as approx. two times smaller than the values of the corresponding MAEs for the pcS-2 basis set. The computational efficiency of the pcS-3/pcS-2 basis sets compared to the pcS-2 basis set can be demonstrated on the example of one of the compounds of set 1. Namely, for instance, in the case of fluorethan (molecule **5** of set 1), the number of contracted basis set functions participating in the calculations with the pcS-2 basis set is 169, while that number for the pcS-3/pcS-2 scheme is 209. The difference in these numbers equates to 40 functions, which is only approx. 24% of the number of basis set functions for the pcS-2 calculation. At that, the absolute error for this compound drops by ca. 2.5 and 5.5 times in going from pcS-2 to pcS-3/pcS-2 in the CCSD and DFT calculations, respectively.

In general, one can conclude that the LDBS schemes with the fluorine atoms considered as the atoms of interest indeed represent a powerful means of heightening the accuracy of the ^19^F NMR chemical shifts calculations on account of a moderate increase in the total number of basis set functions.

### 2.3. Testing Different Basis Set Schemes in the Calculations of ^19^NMR Chemical Shifts Against the Experimental Values

**Figure 7:**
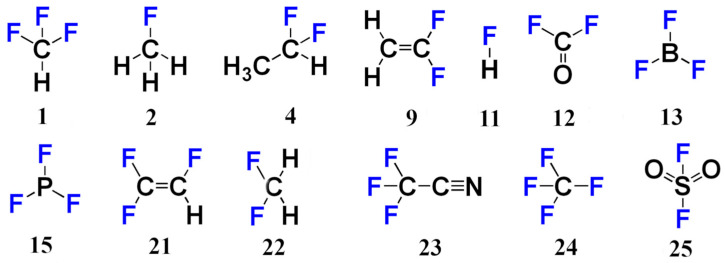
Fluorine compounds of set 2.

In this section, we have examined the performance of different basis set schemes (pcS-2/pcS-1, pcS-3/pcS-1, pcS-2, pcS-3/pcS-2, pcS-3, and pcS-4/pcS-3) against the experimental data in order to check if their accuracy hierarchy, established in the theoretical study, would hold in this case. For this investigation, we have found the gas-phase NMR experimental values for fluorine chemical shifts of 13 small and highly symmetrical compounds, which are shown in Figure 7 (set 2). The experimental values were mostly taken from the two works of Jameson with co-authors [126,127] and from the work of Hindermann et al. [128].

The calculations of fluorine shielding constants were carried out using the CCSD method, taking into account vibrational corrections. The latter were calculated at the DFT (BHandHLYP) level of theory in combination with the pcS-2 basis set in all stages, at zero temperature (zero-point vibrational corrections, ZPVC). The second-order vibrational perturbation theory (VPT2) [129] was applied in combination with the effective geometry approach by Ruud et al. [130]. The solvent corrections were not calculated as they are not needed for comparison with the gas-phase NMR experiment. The chemical shifts were estimated from the calculated shielding constants using the linear regression formulae presented earlier in the article (see Equations (3)–(5)), with the ideal values taken from the experiment.

All fluorine shielding constants of compounds of set 2 calculated at the CCSD level of theory with different basis set schemes are given in Table S7 of Supplementary Materials, while their corresponding vibrational corrections are given in Table S8 of Supplementary Materials. The scaled calculated fluorine chemical shifts and the experimental values are presented in Table 1.

As one can observe from Table 1, the previously recommended LDBS scheme, pcS-3/pcS-2, works well for practically most of the compounds of set 2; the only exception is molecule **25**, for which the results are apparently more strongly dependent on the basis set used on the fluorine atom. The calculated value of its fluorine chemical shift approaches the experimental value only within the largest pcS-4/pcS-3 basis set scheme. It is also interesting to note that the vibrational corrections to fluorine shielding constants occurred to be not negligible and capable of tangibly affecting the final accuracy of the results. To be more precise, their values vary in the range from −13 to −3 ppm, depending on the compound, and, in percentages, these are about 1–6% of the total value of the fluorine shielding constant. Their values are presented in the Supplementary Materials.

**Figure 8:**
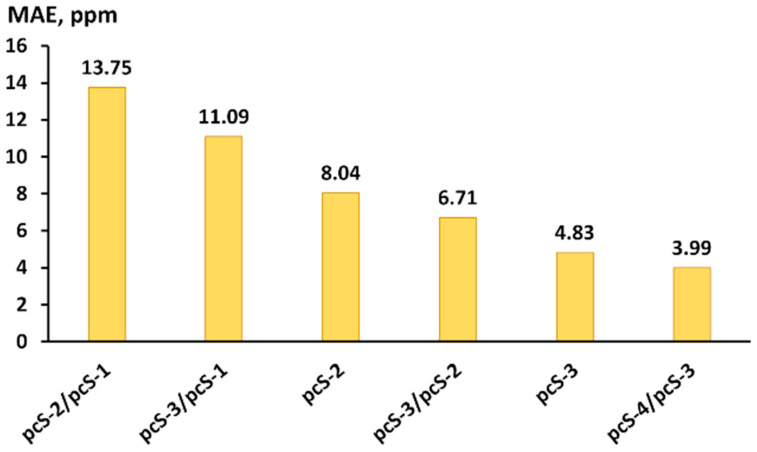
The MAEs (in ppm) of scaled calculated ^19^F NMR chemical shifts in compounds of set 2 against the gas-phase experiment. Fluorine shielding constants were calculated at the CCSD level with different basis set schemes, taking into account vibrational corrections.

The MAEs of fluorine chemical shifts calculated with the six considered basis set schemes were evaluated against the experiment and plotted in Figure 8. As one can see from Figure 8, the sequence order of the basis set schemes is the same as that found in the theoretical study (see Figure 6).

From the perspective of practical utility, the pcS-3/pcS-2 scheme represents the most commendable choice. Indeed, the pcS-3/pcS-2 scheme provides the accuracy being intermediate between the accuracy of pcS-2 and pcS-3 basis set, while basis functional space for the pcS-3/pcS-2 scheme is only a little larger than that for the problem considered within the pcS-2 full basis scheme, because it implies the usage of the pcS-3 basis set only on fluorine atoms, which are usually considerably less in number than the carbon and hydrogen atoms included in medium- or large-sized fluorine compounds. Moreover, as shown in Figure 6, the distinctions between the errors provided by the pcS-2, pcS-3/pcS-2, and pcS-3 basis set schemes are less pronounced within the DFT computational protocol than within the CCSD method. So, in the case of medium- or large-sized fluorine compounds, when there is no other alternative but to use the DFT method, the advantage of the pcS-3/pcS-2 scheme in the sense of accuracy of the results will be more obvious. To demonstrate this, we have performed the calculation of fluorine NMR chemical shifts in perfluoro-1,1-diphenylpropan-1-ol [131] (composed of 32 atoms, including 15 fluorine atoms) using the DFT-based computational protocol that was proposed in this paper, with the pcS-2, pcS-3/pcS-2, and pcS-3 basis set schemes applied at the shielding calculation stage. The theoretical results were compared with the experimental data.

To be more precise, the equilibrium geometry of perfluoro-1,1-diphenylpropan-1-ol was calculated at the DFT(M062X)/pc-2 level, while NMR shielding constants were calculated within the GIAO-DFT(BHandHLYP) method with the three basis set schemes mentioned in the previous sentence. Solvent effects were taken into account within the integral equation formalism of the polarizable continuum model, the IEF-PCM [132,133]. NMR shielding constants were conformationally averaged via the Boltzmann distribution at room temperature.

**Figure 9:**
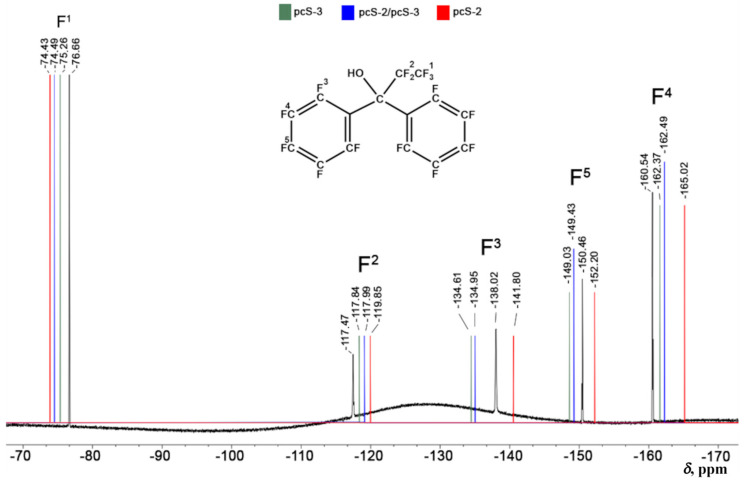
The ^19^F NMR spectrum of perfluoro-1,1-diphenylpropan-1-ol has been recorded on a Bruker AVANCE 400 spectrometer (376.43 MHz, respectively) in chloroform at 20–25 °C, using CFCl_3_ as the internal standard. The experimental spectrum is shown in black, and the theoretical values that were calculated, with the pcS-2, pcS-3/pcS-2, and pcS-3 basis set schemes shown with red, blue, and green vertical lines, respectively. All NMR chemical shifts are in ppm units.

NMR chemical shifts were obtained from shielding constants using the simplified IUPAC expression [114,115] as the difference between the shielding constant of the standard and that of the compound under consideration. Compound CFCl_3_ was used as the standard. The experimental ^19^F NMR spectrum of perfluoro-1,1-diphenylpropan-1-ol is presented in Figure 9. Theoretical values that were obtained with the pcS-2, pcS-3/pcS-2, and pcS-3 basis set schemes are shown in Figure 9 as vertical lines of different colors, namely, red for pcS-2, blue for pcS-3/pcS-2, and green for pcS-3.

As shown in Figure 9, there are five ^19^F NMR signals, and, in most cases, the pcS-3/pcS-2 scheme gives values that are practically inseparable from the results of the pcS-3 scheme, which, in turn, gives values in close agreement with the experiment. The mean absolute errors calculated for the three basis set schemes under consideration over all five fluorine chemical shifts against experiment are as follows: 2.93 ppm for pcS-2, 1.76 ppm for pcS-3/pcS-2, and 1.70 ppm for pcS-3. This result suggests that the pcS-3/pcS-2 scheme is practically of the same accuracy as the pcS-3 scheme, while pcS-2 gives noticeably inferior accuracy. Thus, for the calculation of ^19^F NMR chemical shifts in medium- or large-sized compounds with our proposed DFT protocol, the most reasonable choice between different basis sets intended for the NMR shielding constant calculation stage would be the pcS-3/pcS-2 LDBS.

## 3. Materials and Methods

The optimization of the structures for all considered molecules was carried out within either the CCSD or DFT level of theory using various basis sets and exchange-correlation functionals (in the latter case) in the gas phase. The functionals and basis sets used were described in the main text, and the appropriate references were given. The geometry optimizations of all molecules were carried out within the Gaussian 09 program package [134]. All calculated molecular equilibrium geometries are presented in the Supplementary Materials.

All calculations of fluorine shielding constants were carried out using the gauge-independent atomic orbital (GIAO) method [135] in the gas phase. The CCSD and DFT calculations of fluorine shielding constants were performed within the CFOUR [136] and Gaussian 09 program packages, respectively. The vibrational corrections were calculated using the Dalton program [137]. All calculated ^19^F NMR shielding constants and chemical shifts are presented in the Supplementary Materials.

## 4. Conclusions

This work was aimed at theoretical and systematical study of computational schemes for ^19^F NMR chemical shifts, with special emphasis placed on the basis set issue.

The performance of twenty different DFT exchange-correlation functionals was tested on the calculation of equilibrium bond lengths in a representative series of fluorine compounds against the CCSD method. Four hybrid GGA functionals gave bond lengths in the closest vicinity to the CCSD values, with MAPEs less than 0.4%. These are HSE06 (MAPE = 0.379%), ωB97-XD (MAPE = 0.352%), PBE0 (MAPE = 0.347%), and M062X (MAPE = 0.279%). At the same time, pure GGA functionals, such as the BP86, PBE, and PW91, turned out to be the least suitable for the geometry optimization of fluorine compounds. In general, the M062X exchange-correlation functional can be recommended for the optimization of geometry parameters of fluorine compounds as the functional that gives the best accuracy relative to high-quality CCSD results.

The performance of twenty-four different basis sets of different types and quality has been explored in the geometry optimization stage within the DFT(M062X) method. Dunning’s ((aug-)cc-pVXZ, X = D, T, Q, 5), Pople’s (6-31(++)G(x,y) and 6-311(++)G(x,y)), Jensen’s ((aug-)pc-n, *n* = 1–4), and Rusakov’s (pecG-*n*, *n* = 1, 2) series were considered. It was shown that the pecG-2 or pc-2 basis sets represent the most balanced choice for the geometry optimization of fluorine compounds, because they are moderate in size and provide accuracy between that of the cc-pVTZ and cc-pVQZ basis sets. Extra polarization functions were found to improve the accuracy of equilibrium geometries of fluorine compounds, while extra diffuse functions were found to deteriorate the accuracy or have no effect at all, provided that the computational time increases substantially when they are in use.

Fourteen exchange-correlation functionals were applied to the calculation of fluorine NMR chemical shifts, using the pcS-3 basis set. The results were compared with the data obtained at the CCSD/pcS-3 level. It was found that the best functional for fluorine NMR chemical shifts calculation is the BHandHLYP, which provides the lowest MAE of only 5.24 ppm against the CCSD level.

The performance of different basis sets was studied on the calculation of fluorine chemical shifts. In this study, three families of basis sets were considered, namely, Dunning’s series ((aug-)cc-pVXZ, X = D, T, Q, 5), Pople’s series (6-31(++)G(x,y) and 6-311(++)G(x,y)), and Jensen’s series ((aug-)pc-*n*, *n* = 1–4), including the shielding-oriented basis sets (aug-)pcS-*n*, *n* = 1–4. Based on the obtained results, the pcS-3 (with MAE of 0.05 ppm) can be recommended as the basis set that represents the best-balanced choice for the calculation of fluorine chemical shifts with excellent accuracy. In addition, it was found that the increase in the number of polarization functions, especially in the first polarization shell, noticeably improves the accuracy of fluorine chemical shifts. At the same time, adding extra diffuse functions improves the accuracy of the calculated fluorine chemical shifts in general; however, the effect is more pronounced in the case of double-zeta quality basis sets.

Different LDBS schemes were considered along with the full basis sets against the pcS-4/pcS-3 LDBS scheme, which was regarded as the one giving theoretical reference data. It was found that the improvement in the quality of the basis set only on fluorine atoms gives a significant benefit in the accuracy of the results at an insignificant increase in computational costs. In particular, the MAE of the pcS-3/pcS-2 scheme was found to be only half the MAE of the pcS-2 scheme in both the CCSD and DFT calculations of fluorine chemical shifts. Overall, the pcS-3/pcS-2 LDBS scheme is recommended as the most balanced LDBS scheme for fluorine chemical shift calculations.

## Figures and Tables

**Table 1:** Calculated scaled fluorine chemical shifts (in ppm) against gas-phase experiment.

Molecule	pcS-2/pcS-1	pcS-2	pcS-3/pcS-2	pcS-3/pcS-1	pcS-3	pcS-4/pcS-3	Exp.^ [2]^
**1**	−76.61	−76.45	−75.07	−71.25	−78.78	−77.30	−78.49
**2**	−244.45	−257.03	−261.96	−255.75	−267.90	−266.77	−275.26
**4**	−111.88	−106.26	−109.26	−111.10	−111.64	−110.44	−110.72
**9**	−84.18	−80.88	−85.22	−89.67	−85.92	−84.72	−83.46
**11**	−167.82	−198.27	−196.65	−184.62	−205.13	−203.47	−214.40
**12**	−39.72	−32.53	−33.96	−36.33	−31.90	−30.60	−26.05
**13**	−118.75	−127.88	−126.68	−121.62	−129.54	−128.25	−131.53
**15**	−64.17	−56.72	−43.97	−55.45	−38.52	−36.25	−32.72
**21** ^ [1]^	−206.70	−203.69	−209.93	−208.68	−213.71	−212.41	−208.55
	−102.23	−101.86	−103.89	−104.25	−106.56	−104.88	−101.82
	−134.01	−129.88	−135.21	−135.50	−137.85	−136.24	−129.66
**22**	−134.26	−135.13	−137.97	−135.32	−141.88	−140.43	−143.48
**23**	−67.995	−61.080	−59.208	−61.315	−59.216	−57.307	−56.41
**24**	−66.39	−64.45	−59.28	−54.74	−62.67	−60.63	−63.34
**25**	−5.73	7.19	13.36	0.68	46.28	24.77	30.98

^[1]^ Fluorine chemical shifts in molecule **21** follow the order: $\tilde{\delta }(=\mathrm{CHF})$, $\tilde{\delta }(\mathrm{F trans to H})$, $\tilde{\delta }(\mathrm{F cis to H})$. ^[2]^ For the experimental values of molecules **1**, **2**, **12**, **13**, **15**, see reference [126]; for molecules **4**, **9**, **21**–**25**, see ref. [127]; and for molecule **11**, see ref. [128].

## Data Availability

The original contributions presented in the study are included in the article and Supplementary Materials. Further inquiries can be directed to the corresponding author.

## Supplementary Materials

The following supporting information can be downloaded at: https://www.mdpi.com/article/10.3390/ijms26146930/s1.
