# Supplementary material for: A Quest for Effective 19F NMR Spectra Modeling: What Brings a Good Balance Between Accuracy and Computational Cost in Fluorine Chemical Shift Calculations?
Source: Int J Mol Sci. 2025 Jul 18;26(14):6930. doi: 10.3390/ijms26146930 (PMC12296128; doi:10.3390/ijms26146930)
Supplement: Supplementary file 1 [file ijms-26-06930-s001.zip › ijms-3721089-supplementary.pdf]

Supporting Information

« A quest for effective ^{19}F NMR spectra modeling: what brings a good balance between the accuracy and computational cost in fluorine chemical shift calculations?»

Stepan A. Ukhanev, Yuriy Yu. Rusakov and Irina L. Rusakova

Contents

Equilibrium Cartesian coordinates of compounds **1-20** optimized at the CCSD/pc-3 level of theory in the gas phase 4
Equilibrium Cartesian coordinates of compounds **1-20** optimized at the BP86/pc-3 level of theory in the gas phase 8
Equilibrium Cartesian coordinates of compounds **1-20** optimized at the PBE/pc-3 level of theory in the gas phase 11
Equilibrium Cartesian coordinates of compounds **1-20** optimized at the PW91/pc-3 level of theory in the gas phase 15
Equilibrium Cartesian coordinates of compounds **1-20** optimized at the BHandH/pc-3 level of theory in the gas phase 19
Equilibrium Cartesian coordinates of compounds **1-20** optimized at the TPSSh/pc-3 level of theory in the gas phase 22
Equilibrium Cartesian coordinates of compounds **1-20** optimized at the B3LYP/pc-3 level of theory in the gas phase 26
Equilibrium Cartesian coordinates of compounds **1-20** optimized at the X3LYP/pc-3 level of theory in the gas phase 30
Equilibrium Cartesian coordinates of compounds **1-20** optimized at the O3LYP/pc-3 level of theory in the gas phase 33
Equilibrium Cartesian coordinates of compounds **1-20** optimized at the tHCTHhyb/pc-3 level of theory in the gas phase 37
Equilibrium Cartesian coordinates of compounds **1-20** optimized at the B97-1/pc-3 level of theory in the gas phase 41
Equilibrium Cartesian coordinates of compounds **1-20** optimized at the M06HF/pc-3 level of theory in the gas phase 44
Equilibrium Cartesian coordinates of compounds **1-20** optimized at the CAM-B3LYP/pc-3 level of theory in the gas phase 48
Equilibrium Cartesian coordinates of compounds **1-20** optimized at the mPW3PBE/pc-3 level of theory in the gas phase 52

Equilibrium Cartesian coordinates of compounds **1-20** optimized at the SVWN/pc-3 level of theory in the gas phase ... 55
Equilibrium Cartesian coordinates of compounds **1-20** optimized at the B97-2/pc-3 level of theory in the gas phase ... 59
Equilibrium Cartesian coordinates of compounds **1-20** optimized at the BHandHLYP/pc-3 level of theory in the gas phase ... 63
Equilibrium Cartesian coordinates of compounds **1-20** optimized at the HSE06/pc-3 level of theory in the gas phase ... 66
Equilibrium Cartesian coordinates of compounds **1-20** optimized at the ωB97XD /pc-3 level of theory in the gas phase ... 70
Equilibrium Cartesian coordinates of compounds **1-20** optimized at the PBE0/pc-3 level of theory in the gas phase... 74
Equilibrium Cartesian coordinates of compounds **1-20** optimized at the M062X/pc-3 level of theory in the gas phase ... 77

Table S1. Values of equilibrium bond lengths of compounds **1-20** calculated at the CCSD and DFT level of theory with various exchange-correlation functionals... 82

Equilibrium Cartesian coordinates of compounds **1-20** optimized at the DFT(M062X) level of theory with different basis sets in gas phase ... 84
Geometries optimized with the cc-pVDZ basis set ... 84
Geometries optimized with the aug-cc-pVDZ basis set ... 89
Geometries optimized with the cc-pVTZ basis set ... 94
Geometries optimized with the aug-cc-pVTZ basis set ... 100
Geometries optimized with the cc-pVQZ basis set ... 105
Geometries optimized with the aug-cc-pVQZ basis set ... 111
Geometries optimized with the cc-pV5Z basis set ... 116
Geometries optimized with the aug-cc-pV5Z basis set ... 122
Geometries optimized with the cc-pV6Z basis set ... 127
Geometries optimized with the pc-1 basis set ... 133
Geometries optimized with the aug-pc-1 basis set ... 138
Geometries optimized with the pc-2 basis set ... 144
Geometries optimized with the aug-pc-2 basis set ... 149
Geometries optimized with the pc-3 basis set (including molecules **21-25**) ... 155
Geometries optimized with the aug-pc-3 basis set ... 161
Geometries optimized with the pc-4 basis set ... 167
Geometries optimized with the aug-pc-4 basis set ... 172
Geometries optimized with the 6-31G(d,p) basis set ... 178
Geometries optimized with the 6-311G(d,p) basis set ... 183
Geometries optimized with the 6-31++G(d,p) basis set ... 189

Geometries optimized with the 6-311++G(d,p) basis set 194
Geometries optimized with the 6-311++G(2d,2p) basis set 200
Geometries optimized with the 6-311++G(3df,3pd) basis set 205
Geometries optimized with the pecG-1 basis set 211
Geometries optimized with the pecG-2 basis set 216

Table S2. Values of equilibrium bond lengths of compounds **1-20** calculated at the DFT(M062X) level of theory with various one-electron basis sets 222

Table S3. ^{19}F NMR shielding constants of molecules **1-20** calculated within the GIAO-DFT method with different exchange-correlation functionals using the pcS-3 basis set, in the gas phase 225

Table S4. ^{19}F NMR shielding constants of molecules **1-20** calculated within the GIAO-DFT(BHandHLYP) method with different basis sets, in the gas phase 226

Table S5. ^{19}F NMR shielding constants of molecules **1-20** calculated within the GIAO-CCSD method with different basis set schemes, in the gas phase 229

Table S6. ^{19}F NMR shielding constants of molecules **1-20** calculated within the GIAO-DFT(BHandHLYP) method with different basis set schemes, in the gas phase 230

Table S7. ^{19}F NMR shielding constants of molecules **1, 2, 4, 9, 11-13, 15, 21-25** calculated within the GIAO-CCSD method with different basis set schemes, in the gas phase 231

Table S8. Vibrational corrections to ^{19}F NMR shielding constants of molecules **1, 2, 4, 9, 11-13, 15, 21-25** calculated within the GIAO-DFT(BHandHLYP) method with the pcS-2 basis set, in the gas phase 231

Equilibrium Cartesian coordinates of compounds 1-20 optimized at the CCSD/pc-3 level of theory in the gas phase (all coordinates are given in Angstroms, Å)

Compound 1

C	-0.003204000	-0.035284000	0.050291000
F	1.136945000	0.636618000	0.029977000
F	-1.001788000	0.833177000	0.029965000
F	-0.071327000	-0.776625000	-1.043974000
H	-0.060427000	-0.657886000	0.933642000

Compound 2

C	-0.032269000	-0.055847000	0.000002000
F	0.655299000	1.134359000	-0.000220000
H	0.680059000	-0.872269000	-0.000211000
H	-0.651322000	-0.103080000	0.887988000
H	-0.651767000	-0.103164000	-0.887659000

Compound 3

C	-1.069826000	0.235287000	-0.010851000
F	-1.683500000	-0.214077000	1.056948000
F	-1.679903000	-0.249591000	-1.087289000
F	0.168745000	-0.246129000	-0.023255000
O	-1.090258000	1.572342000	0.020201000
H	-0.646259000	1.911169000	-0.755753000

Compound 4

C	0.549569000	-0.315776000	-0.112147000
C	-0.741994000	0.439056000	-0.099049000
F	1.595768000	0.546540000	-0.091537000
F	0.652061000	-1.087111000	0.998297000
H	0.675542000	-0.963244000	-0.973613000
H	-1.573025000	-0.256554000	-0.094873000
H	-0.807588000	1.068492000	-0.978828000
H	-0.785235000	1.057298000	0.790251000

Compound 5

C	-0.768380000	0.012003000	-0.030396000
C	0.733143000	-0.007749000	-0.046037000
F	1.214187000	-0.073787000	1.247622000
H	-1.133375000	0.874731000	0.515755000
H	-1.146246000	0.061735000	-1.047356000
H	1.116115000	-0.872435000	-0.579429000
H	-1.155967000	-0.886199000	0.437489000
H	1.140421000	0.891600000	-0.497649000

Compound 6

C	-0.234100000	-0.645773000	0.000000000
C	0.424508000	0.494916000	0.000000000
F	-1.562953000	-0.700998000	0.000000000
F	-0.192042000	1.673370000	0.000000000
H	0.258400000	-1.599354000	0.000000000
H	1.496586000	0.545141000	0.000000000

Compound 7

C	-0.222488000	-0.557413000	0.000000000
C	0.416313000	0.592557000	0.000000000
F	-1.558603000	-0.624460000	0.000000000
H	0.232597000	-1.532029000	0.000000000
H	1.490224000	0.594020000	0.000000000
H	-0.113743000	1.528125000	0.000000000

Compound 8

C	-0.315325000	-0.576990000	0.000000000
C	0.315325000	0.576990000	0.000000000
F	-1.647564000	-0.609337000	0.000000000
F	1.647564000	0.609337000	0.000000000
H	0.159687000	-1.540339000	0.000000000
H	-0.159687000	1.540339000	0.000000000

Compound 9

C	-0.240115000	-0.468103000	0.000000000
C	0.415402000	0.667342000	0.000000000
F	-1.544126000	-0.584748000	0.000000000
F	0.310926000	-1.655708000	0.000000000
H	1.486730000	0.658314000	0.000000000
H	-0.128117000	1.590604000	0.000000000

Compound 10

N	0.592269000	1.025810000	0.000000000
C	0.018769000	0.032507000	0.000000000
F	-0.611038000	-1.058318000	0.000000000

Compound 11

F	0.415753000	-0.189079000	0.000000000
H	-0.415753000	0.189079000	0.000000000

Compound 12

C	-0.556680000	0.059718000	0.000000000
O	0.601213000	0.183957000	0.000000000
F	-1.429125000	1.025327000	0.000000000
F	-1.204408000	-1.069001000	0.000000000

Compound 13

B	-0.450970000	0.132264000	-0.205809000
F	-0.668422000	1.361096000	0.170843000
F	0.635141000	-0.482319000	0.170771000
F	-1.319750000	-0.482042000	-0.958806000

Compound 14

Si	-0.969726000	0.233068000	0.033280000
H	-1.673868000	-0.264814000	1.203974000
F	-1.713650000	-0.292795000	-1.257065000
H	0.396398000	-0.264329000	0.010259000
H	-0.983154000	1.686871000	0.010551000

Compound 15

F	-0.571776000	1.473180000	0.002467000
P	-0.724717000	-0.049186000	0.269012000
F	0.758330000	-0.446836000	0.035535000
F	-1.265837000	-0.448160000	-1.131015000

Compound 16

C	0.122938000	0.165388000	-0.000230000
C	0.707499000	1.209188000	-0.000308000
C	-0.615465000	-1.092026000	-0.000130000
F	0.244237000	-2.167278000	-0.000332000
H	1.231457000	2.128224000	-0.000379000
H	-1.241012000	-1.162678000	0.883527000
H	-1.241355000	-1.162618000	-0.883548000

Compound 17

C	0.294498000	-0.510070000	0.000000000
C	-0.294498000	0.510070000	0.000000000
F	-0.934109000	1.617876000	0.000000000
F	0.934109000	-1.617876000	0.000000000

Compound 18

F	-1.485450000	-0.112530000	-0.630938000
C	-0.093050000	-0.097255000	-0.603120000
C	0.361737000	1.337076000	-0.637295000
C	0.360485000	-0.843507000	0.622736000
H	0.231535000	-0.617182000	-1.502570000
H	1.446979000	1.385096000	-0.666045000
H	-0.029967000	1.838868000	-1.516091000
H	0.013558000	1.861836000	0.248102000
H	1.445671000	-0.893326000	0.650525000
H	-0.032086000	-1.855129000	0.618450000
H	0.012291000	-0.338152000	1.519344000

Compound 19

C	0.018544000	0.188124000	-0.348096000
C	1.278034000	1.020560000	-0.313774000
C	-1.217107000	1.040828000	-0.510903000
F	0.101692000	-0.629179000	-1.484563000
C	-0.083261000	-0.730648000	0.846030000
H	2.158504000	0.376325000	-0.254425000
H	1.271320000	1.682709000	0.556257000
H	1.354919000	1.631811000	-1.216131000
H	-2.106255000	0.410965000	-0.591377000
H	-1.336409000	1.703880000	0.350241000
H	-1.141468000	1.652101000	-1.413350000
H	0.796526000	-1.375762000	0.905946000
H	-0.151389000	-0.147524000	1.768399000
H	-0.971849000	-1.361389000	0.766244000

Compound 20

C	0.986370000	0.110407000	-0.125202000
C	0.190615000	-0.708462000	0.620968000
C	-1.164435000	-0.367278000	0.424482000
C	-1.158093000	0.680404000	-0.461569000
N	0.135451000	0.961705000	-0.787099000
F	0.626372000	-1.685867000	1.417619000
H	2.048049000	0.152146000	-0.232493000
H	-2.018548000	-0.828593000	0.872372000
H	-1.969945000	1.237793000	-0.877670000
H	0.423763000	1.677943000	-1.412108000

Equilibrium Cartesian coordinates of compounds 1-20 optimized at the BP86/pc-3 level of theory in the gas phase

Compound 1

C	-0.003307000	-0.036397000	0.051506000
F	1.161792000	0.649641000	0.032503000
F	-1.023845000	0.850513000	0.032497000
F	-0.073034000	-0.795241000	-1.065489000
H	-0.061406000	-0.668516000	0.948883000

Compound 2

C	-0.033695000	-0.058339000	-0.000015000
F	0.665946000	1.152887000	-0.000239000
H	0.689863000	-0.885114000	-0.000166000
H	-0.660813000	-0.104707000	0.900912000
H	-0.661301000	-0.104729000	-0.900592000

Compound 3

C	-1.071197000	0.236714000	-0.008496000
F	-1.698019000	-0.223552000	1.082245000
F	-1.694361000	-0.266195000	-1.111781000
F	0.197232000	-0.262603000	-0.023056000
O	-1.093939000	1.592278000	0.026447000
H	-0.640717000	1.932359000	-0.765360000

Compound 4

C	0.552130000	-0.318344000	-0.113779000
C	-0.748973000	0.443807000	-0.097971000
F	1.623337000	0.557533000	-0.095449000
F	0.660450000	-1.109319000	1.016534000
H	0.686126000	-0.977743000	-0.986182000
H	-1.591470000	-0.258907000	-0.095907000
H	-0.818033000	1.079989000	-0.989102000
H	-0.798468000	1.071684000	0.800356000

Compound 5

C	-0.776418000	0.012103000	-0.030365000
C	0.737507000	-0.007782000	-0.048528000
F	1.238218000	-0.078056000	1.268995000
H	-1.150128000	0.883490000	0.522621000
H	-1.158913000	0.067493000	-1.059866000
H	1.129992000	-0.883646000	-0.587235000
H	-1.174835000	-0.898282000	0.435752000
H	1.154477000	0.904579000	-0.501375000

Compound 6

C	-0.239246000	-0.651474000	0.000000000
C	0.426864000	0.502231000	0.000000000
F	-1.585288000	-0.718210000	0.000000000
F	-0.188334000	1.701361000	0.000000000
H	0.262137000	-1.617602000	0.000000000
H	1.514265000	0.550996000	0.000000000

Compound 7

C	-0.224315000	-0.561179000	0.000000000
C	0.418681000	0.599185000	0.000000000
F	-1.578790000	-0.636988000	0.000000000
H	0.233822000	-1.550912000	0.000000000
H	1.506088000	0.600893000	0.000000000
H	-0.111186000	1.549801000	0.000000000

Compound 8

C	-0.318224000	-0.584545000	0.000000000
C	0.318224000	0.584545000	0.000000000
F	-1.671572000	-0.621514000	0.000000000
F	1.671572000	0.621514000	0.000000000
H	0.158118000	-1.563696000	0.000000000
H	-0.158118000	1.563696000	0.000000000

Compound 9

C	-0.242935000	-0.472983000	0.000000000
C	0.419821000	0.674997000	0.000000000
F	-1.570089000	-0.594200000	0.000000000
F	0.315726000	-1.682918000	0.000000000
H	1.504576000	0.670601000	0.000000000
H	-0.126398000	1.612204000	0.000000000

Compound 10

N	0.600572000	1.040189000	0.000000000
C	0.018278000	0.031656000	0.000000000
F	-0.618849000	-1.071847000	0.000000000

Compound 11

F	0.423643000	-0.192667000	0.000000000
H	-0.423643000	0.192667000	0.000000000

Compound 12

C	-0.548922000	0.060551000	0.000000000
O	0.625206000	0.186532000	0.000000000

F	-1.447251000	1.044603000	0.000000000
F	-1.218033000	-1.091684000	0.000000000

Compound 13

B	-0.450520000	0.132584000	-0.206582000
F	-0.672063000	1.380693000	0.177136000
F	0.652404000	-0.492285000	0.177062000
F	-1.333822000	-0.491994000	-0.970617000

Compound 14

Si	-0.968848000	0.233690000	0.034843000
H	-1.691109000	-0.277043000	1.232122000
F	-1.729769000	-0.304156000	-1.285048000
H	0.429399000	-0.276551000	0.009422000
H	-0.983672000	1.722060000	0.009661000

Compound 15

F	-0.576405000	1.512222000	0.009980000
P	-0.730163000	-0.052806000	0.278746000
F	0.793158000	-0.464698000	0.044055000
F	-1.290591000	-0.465719000	-1.156783000

Compound 16

C	0.117323000	0.167762000	-0.000255000
C	0.712766000	1.220168000	-0.000316000
C	-0.617152000	-1.087436000	-0.000137000
F	0.255902000	-2.192022000	-0.000325000
H	1.245437000	2.147948000	-0.000356000
H	-1.252801000	-1.169131000	0.895486000
H	-1.253176000	-1.169088000	-0.895498000

Compound 17

C	0.298894000	-0.517685000	0.000000000
C	-0.298894000	0.517685000	0.000000000
F	-0.944693000	1.636207000	0.000000000
F	0.944693000	-1.636207000	0.000000000

Compound 18

F	-1.511230000	-0.119205000	-0.642514000
C	-0.088469000	-0.097376000	-0.603324000
C	0.362094000	1.352611000	-0.643604000
C	0.360826000	-0.856731000	0.633045000
H	0.236893000	-0.624659000	-1.515505000
H	1.459170000	1.408879000	-0.679320000
H	-0.037498000	1.857732000	-1.532063000
H	0.016505000	1.888606000	0.251052000

H	1.457834000	-0.916714000	0.664508000
H	-0.039640000	-1.878381000	0.626814000
H	0.015216000	-0.348966000	1.544012000

Compound 19

C	0.017963000	0.194081000	-0.339727000
C	1.294643000	1.029411000	-0.316344000
C	-1.233014000	1.049870000	-0.515714000
F	0.103028000	-0.642940000	-1.503382000
C	-0.084307000	-0.744484000	0.858957000
H	2.177314000	0.379315000	-0.257710000
H	1.300236000	1.700436000	0.553742000
H	1.369852000	1.640370000	-1.225306000
H	-2.123995000	0.414072000	-0.599717000
H	-1.365611000	1.720076000	0.344858000
H	-1.153950000	1.662967000	-1.422934000
H	0.799882000	-1.392712000	0.915214000
H	-0.152884000	-0.170204000	1.793236000
H	-0.977358000	-1.377458000	0.775328000

Compound 20

C	0.996675000	0.111306000	-0.126667000
C	0.189101000	-0.716871000	0.628184000
C	-1.170891000	-0.373018000	0.429766000
C	-1.166954000	0.685361000	-0.465158000
N	0.137642000	0.966048000	-0.790916000
F	0.633482000	-1.705993000	1.434161000
H	2.071950000	0.157089000	-0.238303000
H	-2.036753000	-0.840436000	0.883611000
H	-1.987325000	1.252224000	-0.888688000
H	0.432673000	1.694490000	-1.426691000

Equilibrium Cartesian coordinates of compounds 1-20 optimized at the PBE/pc-3 level of theory in the gas phase

Compound 1

C	-0.003278000	-0.036092000	0.051090000
F	1.160398000	0.648972000	0.032265000
F	-1.022594000	0.849598000	0.032255000
F	-0.072934000	-0.794117000	-1.064381000
H	-0.061391000	-0.668362000	0.948672000

Compound 2

C	-0.032892000	-0.056947000	0.000001000
F	0.665209000	1.151581000	-0.000259000
H	0.689345000	-0.884836000	-0.000173000
H	-0.660602000	-0.104899000	0.900409000
H	-0.661060000	-0.104899000	-0.900077000

Compound 3

C	-1.071119000	0.237206000	-0.008631000
F	-1.697267000	-0.222809000	1.080933000
F	-1.693540000	-0.265174000	-1.110709000
F	0.195891000	-0.261588000	-0.023228000
O	-1.093921000	1.590930000	0.026424000
H	-0.641044000	1.930435000	-0.764788000

Compound 4

C	0.551389000	-0.317526000	-0.113194000
C	-0.747683000	0.443055000	-0.097981000
F	1.620835000	0.557063000	-0.095039000
F	0.659370000	-1.107329000	1.015302000
H	0.684654000	-0.976814000	-0.986065000
H	-1.589856000	-0.259543000	-0.095897000
H	-0.816607000	1.079028000	-0.988875000
H	-0.797003000	1.070766000	0.800249000

Compound 5

C	-0.774628000	0.012070000	-0.030286000
C	0.736382000	-0.007947000	-0.047096000
F	1.236441000	-0.076730000	1.267855000
H	-1.149467000	0.883739000	0.520956000
H	-1.156264000	0.066285000	-1.059794000
H	1.128345000	-0.883903000	-0.586248000
H	-1.173968000	-0.897357000	0.436086000
H	1.153060000	0.903744000	-0.501474000

Compound 6

C	-0.239577000	-0.651017000	0.000000000
C	0.426295000	0.502289000	0.000000000
F	-1.583666000	-0.716771000	0.000000000
F	-0.188763000	1.699239000	0.000000000
H	0.262398000	-1.616819000	0.000000000
H	1.513711000	0.550380000	0.000000000

Compound 7

C	-0.224891000	-0.560704000	0.000000000
C	0.418402000	0.598868000	0.000000000
F	-1.577040000	-0.636359000	0.000000000
H	0.234001000	-1.550169000	0.000000000
H	1.505573000	0.599957000	0.000000000
H	-0.111746000	1.549207000	0.000000000

Compound 8

C	-0.318800000	-0.583983000	0.000000000
C	0.318800000	0.583983000	0.000000000
F	-1.669789000	-0.620684000	0.000000000
F	1.669789000	0.620684000	0.000000000
H	0.158537000	-1.562789000	0.000000000
H	-0.158537000	1.562789000	0.000000000

Compound 9

C	-0.242884000	-0.472895000	0.000000000
C	0.419569000	0.674560000	0.000000000
F	-1.568350000	-0.593870000	0.000000000
F	0.315142000	-1.681248000	0.000000000
H	1.504176000	0.669731000	0.000000000
H	-0.126952000	1.611422000	0.000000000

Compound 10

N	0.600286000	1.039695000	0.000000000
C	0.018009000	0.031192000	0.000000000
F	-0.618296000	-1.070888000	0.000000000

Compound 11

F	0.423328000	-0.192523000	0.000000000
H	-0.423328000	0.192523000	0.000000000

Compound 12

C	-0.549357000	0.060504000	0.000000000
O	0.624342000	0.186439000	0.000000000
F	-1.446475000	1.043487000	0.000000000
F	-1.217510000	-1.090429000	0.000000000

Compound 13

B	-0.450987000	0.132245000	-0.205759000
F	-0.671699000	1.379577000	0.176480000
F	0.651485000	-0.491566000	0.176415000
F	-1.332801000	-0.491259000	-0.970136000

Compound 14

Si	-0.968920000	0.233658000	0.034755000
H	-1.692142000	-0.277811000	1.233229000
F	-1.729940000	-0.304281000	-1.285352000
H	0.430852000	-0.277276000	0.009077000
H	-0.983850000	1.723709000	0.009291000

Compound 15

F	-0.576197000	1.511458000	0.009640000
P	-0.730349000	-0.052961000	0.279030000
F	0.792589000	-0.464235000	0.043757000
F	-1.290043000	-0.465263000	-1.156428000

Compound 16

C	0.116751000	0.166416000	-0.000254000
C	0.712240000	1.218891000	-0.000316000
C	-0.615786000	-1.087670000	-0.000137000
F	0.255356000	-2.190127000	-0.000325000
H	1.244639000	2.146964000	-0.000357000
H	-1.252263000	-1.168159000	0.895055000
H	-1.252637000	-1.168114000	-0.895067000

Compound 17

C	0.298955000	-0.517790000	0.000000000
C	-0.298955000	0.517790000	0.000000000
F	-0.943859000	1.634764000	0.000000000
F	0.943859000	-1.634764000	0.000000000

Compound 18

F	-1.508961000	-0.118955000	-0.642081000
C	-0.089654000	-0.096991000	-0.602660000
C	0.361671000	1.350128000	-0.642555000
C	0.360405000	-0.854581000	0.631417000
H	0.237617000	-0.624177000	-1.514671000
H	1.458632000	1.404935000	-0.677826000
H	-0.037224000	1.856002000	-1.530877000
H	0.016283000	1.885972000	0.252268000
H	1.457300000	-0.913446000	0.661839000
H	-0.039362000	-1.876491000	0.625907000
H	0.014994000	-0.346599000	1.542337000

Compound 19

C	0.018114000	0.193651000	-0.340160000
C	1.292086000	1.028334000	-0.315752000
C	-1.230431000	1.048837000	-0.514864000
F	0.103125000	-0.641342000	-1.501634000
C	-0.084217000	-0.742902000	0.857011000
H	2.174660000	0.378842000	-0.257479000
H	1.296884000	1.698519000	0.554402000
H	1.366763000	1.639769000	-1.224010000
H	-2.121599000	0.413611000	-0.595839000
H	-1.360759000	1.720560000	0.344245000
H	-1.152141000	1.659907000	-1.423001000
H	0.799936000	-1.390432000	0.914395000
H	-0.154047000	-0.168527000	1.790510000

H	-0.976574000	-1.376026000	0.772678000

Compound 20

C	0.994998000	0.111723000	-0.126906000
C	0.188514000	-0.716227000	0.627680000
C	-1.169691000	-0.372751000	0.429459000
C	-1.165220000	0.684845000	-0.464839000
N	0.137211000	0.964706000	-0.789752000
F	0.632499000	-1.703802000	1.432375000
H	2.070309000	0.157321000	-0.238388000
H	-2.035546000	-0.840125000	0.883266000
H	-1.985525000	1.251714000	-0.888379000
H	0.432051000	1.692796000	-1.425217000

Equilibrium Cartesian coordinates of compounds 1-20 optimized at the PW91/pc-3 level of theory in the gas phase

Compound 1

C	-0.003292000	-0.036237000	0.051294000
F	1.159479000	0.648358000	0.032402000
F	-1.021803000	0.848827000	0.032391000
F	-0.072888000	-0.793617000	-1.063398000
H	-0.061296000	-0.667332000	0.947211000

Compound 2

C	-0.033167000	-0.057438000	0.000004000
F	0.664650000	1.150636000	-0.000244000
H	0.688185000	-0.883540000	-0.000191000
H	-0.659606000	-0.104811000	0.898963000
H	-0.660061000	-0.104848000	-0.898632000

Compound 3

C	-1.071131000	0.237323000	-0.008616000
F	-1.696768000	-0.222299000	1.080068000
F	-1.693084000	-0.264903000	-1.109839000
F	0.194908000	-0.261311000	-0.023191000
O	-1.093499000	1.589612000	0.025678000
H	-0.641427000	1.930578000	-0.764099000

Compound 4

C	0.550522000	-0.317231000	-0.113502000
C	-0.747230000	0.442736000	-0.098068000
F	1.619402000	0.556878000	-0.095257000
F	0.658501000	-1.106538000	1.014433000
H	0.683903000	-0.975381000	-0.984567000
H	-1.587988000	-0.258758000	-0.095877000
H	-0.815941000	1.077732000	-0.987466000

H	-0.796070000	1.069263000	0.798803000

Compound 5

C	-0.774193000	0.013086000	-0.030611000
C	0.735698000	-0.008936000	-0.047987000
F	1.234334000	-0.078729000	1.267001000
H	-1.145826000	0.883644000	0.521083000
H	-1.155535000	0.068777000	-1.058292000
H	1.126064000	-0.884122000	-0.585333000
H	-1.173338000	-0.894911000	0.434758000
H	1.152695000	0.901092000	-0.500619000

Compound 6

C	-0.238510000	-0.650580000	0.000000000
C	0.426433000	0.501159000	0.000000000
F	-1.581859000	-0.715387000	0.000000000
F	-0.189057000	1.697000000	0.000000000
H	0.261513000	-1.615232000	0.000000000
H	1.511878000	0.550342000	0.000000000

Compound 7

C	-0.224015000	-0.560152000	0.000000000
C	0.418189000	0.597994000	0.000000000
F	-1.575546000	-0.635390000	0.000000000
H	0.233482000	-1.548086000	0.000000000
H	1.503548000	0.599855000	0.000000000
H	-0.111358000	1.546579000	0.000000000

Compound 8

C	-0.318126000	-0.583287000	0.000000000
C	0.318126000	0.583287000	0.000000000
F	-1.668378000	-0.620196000	0.000000000
F	1.668378000	0.620196000	0.000000000
H	0.158150000	-1.560457000	0.000000000
H	-0.158150000	1.560457000	0.000000000

Compound 9

C	-0.242410000	-0.472073000	0.000000000
C	0.419151000	0.673835000	0.000000000
F	-1.566967000	-0.592990000	0.000000000
F	0.315213000	-1.679610000	0.000000000
H	1.501984000	0.669253000	0.000000000
H	-0.126270000	1.609285000	0.000000000

Compound 10

N	0.599490000	1.038316000	0.000000000
C	0.018136000	0.031411000	0.000000000
F	-0.617626000	-1.069728000	0.000000000

Compound 11

F	0.422985000	-0.192367000	0.000000000
H	-0.422985000	0.192367000	0.000000000

Compound 12

C	-0.549297000	0.060510000	0.000000000
O	0.622930000	0.186288000	0.000000000
F	-1.445709000	1.042731000	0.000000000
F	-1.216923000	-1.089528000	0.000000000

Compound 13

B	-0.450998000	0.132252000	-0.205772000
F	-0.671430000	1.378180000	0.176067000
F	0.650232000	-0.490853000	0.175985000
F	-1.331805000	-0.490580000	-0.969281000

Compound 14

Si	-0.968747000	0.233770000	0.034988000
H	-1.690512000	-0.276661000	1.230762000
F	-1.728786000	-0.303465000	-1.283407000
H	0.427862000	-0.276184000	0.009223000
H	-0.983817000	1.720540000	0.009434000

Compound 15

F	-0.575997000	1.509698000	0.009324000
P	-0.730077000	-0.052766000	0.278560000
F	0.790987000	-0.463441000	0.043369000
F	-1.288914000	-0.464493000	-1.155254000

Compound 16

C	0.116215000	0.166508000	-0.000255000
C	0.711260000	1.217128000	-0.000316000
C	-0.615803000	-1.086026000	-0.000137000
F	0.255432000	-2.188027000	-0.000324000
H	1.243114000	2.143371000	-0.000355000
H	-1.250771000	-1.167399000	0.893636000
H	-1.251146000	-1.167355000	-0.893648000

Compound 17

C	0.298519000	-0.517035000	0.000000000
C	-0.298519000	0.517035000	0.000000000
F	-0.942943000	1.633177000	0.000000000
F	0.942943000	-1.633177000	0.000000000

Compound 18

F	-1.507712000	-0.118864000	-0.641921000
C	-0.088672000	-0.096844000	-0.602403000
C	0.361692000	1.349265000	-0.642130000
C	0.360427000	-0.853783000	0.630882000
H	0.237509000	-0.623141000	-1.512878000
H	1.456863000	1.404267000	-0.677651000
H	-0.037062000	1.854105000	-1.528876000
H	0.016804000	1.883824000	0.251417000
H	1.455532000	-0.912960000	0.661346000
H	-0.039198000	-1.873810000	0.625263000
H	0.015516000	-0.346263000	1.540051000

Compound 19

C	0.017887000	0.194214000	-0.339673000
C	1.291046000	1.027875000	-0.315236000
C	-1.229830000	1.048249000	-0.514475000
F	0.102732000	-0.640655000	-1.500642000
C	-0.083999000	-0.741656000	0.856328000
H	2.171952000	0.378979000	-0.255555000
H	1.295129000	1.697823000	0.552954000
H	1.366493000	1.637314000	-1.222695000
H	-2.119103000	0.413423000	-0.596713000
H	-1.360699000	1.717946000	0.344044000
H	-1.150838000	1.658878000	-1.420839000
H	0.798469000	-1.388807000	0.911191000
H	-0.151478000	-0.168165000	1.788646000
H	-0.975959000	-1.372617000	0.773165000

Compound 20

C	0.994009000	0.111613000	-0.126746000
C	0.188222000	-0.715257000	0.626880000
C	-1.168803000	-0.372098000	0.428847000
C	-1.164662000	0.684406000	-0.464506000
N	0.136976000	0.964360000	-0.789444000
F	0.631709000	-1.702305000	1.431164000
H	2.067535000	0.156773000	-0.237737000
H	-2.032999000	-0.838901000	0.882059000
H	-1.983818000	1.250083000	-0.887116000
H	0.431432000	1.691526000	-1.424102000

Equilibrium Cartesian coordinates of compounds 1-20 optimized at the BHandH/pc-3 level of theory in the gas phase

Compound 1

C	-0.003016000	-0.033237000	0.047251000
F	1.123518000	0.631448000	0.026266000
F	-0.989525000	0.825644000	0.026255000
F	-0.070261000	-0.765010000	-1.034936000
H	-0.060516000	-0.658846000	0.935065000

Compound 2

C	-0.028385000	-0.049094000	0.000004000
F	0.648581000	1.122674000	-0.000206000
H	0.681622000	-0.870950000	-0.000229000
H	-0.650691000	-0.101259000	0.888595000
H	-0.651126000	-0.101372000	-0.888264000

Compound 3

C	-1.069891000	0.236279000	-0.010712000
F	-1.677034000	-0.209660000	1.045671000
F	-1.672855000	-0.245448000	-1.075177000
F	0.154738000	-0.242055000	-0.023263000
O	-1.088200000	1.557419000	0.016691000
H	-0.647758000	1.912465000	-0.753209000

Compound 4

C	0.545811000	-0.311587000	-0.109121000
C	-0.732761000	0.433573000	-0.099273000
F	1.575124000	0.539946000	-0.085911000
F	0.645883000	-1.068662000	0.987216000
H	0.672390000	-0.961960000	-0.974418000
H	-1.566719000	-0.260737000	-0.093840000
H	-0.800484000	1.065691000	-0.978717000
H	-0.774146000	1.052436000	0.792564000

Compound 5

C	-0.759334000	0.022751000	-0.019691000
C	0.727083000	-0.019082000	-0.050282000
F	1.212873000	-0.084781000	1.219572000
H	-1.106183000	0.893599000	0.529567000
H	-1.153360000	0.075505000	-1.031739000
H	1.092645000	-0.892021000	-0.588530000
H	-1.157342000	-0.868368000	0.457645000
H	1.143518000	0.872296000	-0.516543000

Compound 6

C	-0.237282000	-0.637274000	0.000000000
C	0.415553000	0.493440000	0.000000000
F	-1.544385000	-0.687385000	0.000000000
F	-0.194581000	1.650515000	0.000000000
H	0.259986000	-1.592102000	0.000000000
H	1.491107000	0.540107000	0.000000000

Compound 7

C	-0.226385000	-0.550647000	0.000000000
C	0.410883000	0.585559000	0.000000000
F	-1.538826000	-0.615977000	0.000000000
H	0.233193000	-1.527127000	0.000000000
H	1.486480000	0.586153000	0.000000000
H	-0.121045000	1.522839000	0.000000000

Compound 8

C	-0.316733000	-0.569917000	0.000000000
C	0.316733000	0.569917000	0.000000000
F	-1.629090000	-0.600746000	0.000000000
F	1.629090000	0.600746000	0.000000000
H	0.163028000	-1.535285000	0.000000000
H	-0.163028000	1.535285000	0.000000000

Compound 9

C	-0.238874000	-0.465951000	0.000000000
C	0.411248000	0.660147000	0.000000000
F	-1.524639000	-0.584001000	0.000000000
F	0.301832000	-1.638458000	0.000000000
H	1.485035000	0.650655000	0.000000000
H	-0.133900000	1.585308000	0.000000000

Compound 10

N	0.584178000	1.011796000	0.000000000
C	0.017712000	0.030677000	0.000000000
F	-0.601890000	-1.042474000	0.000000000

Compound 11

F	0.414869000	-0.188676000	0.000000000
H	-0.414869000	0.188676000	0.000000000

Compound 12

C	-0.559593000	0.059406000	0.000000000
O	0.588387000	0.182581000	0.000000000
F	-1.419827000	1.012862000	0.000000000
F	-1.197967000	-1.054848000	0.000000000

Compound 13

B	-0.451003000	0.132254000	-0.205754000
F	-0.666190000	1.348550000	0.166980000
F	0.624038000	-0.476039000	0.166896000
F	-1.310847000	-0.475767000	-0.951122000

Compound 14

Si	-0.970760000	0.232318000	0.031453000
H	-1.687294000	-0.274163000	1.213985000
F	-1.710913000	-0.290944000	-1.252269000
H	0.411784000	-0.273630000	0.003672000
H	-0.986818000	1.704419000	0.004160000

Compound 15

F	-0.569584000	1.458586000	-0.001391000
P	-0.724179000	-0.048826000	0.268130000
F	0.745497000	-0.439750000	0.031299000
F	-1.255733000	-0.441011000	-1.122039000

Compound 16

C	0.120921000	0.157280000	-0.000254000
C	0.699295000	1.192006000	-0.000315000
C	-0.603058000	-1.088766000	-0.000138000
F	0.241003000	-2.147423000	-0.000318000
H	1.222425000	2.114850000	-0.000355000
H	-1.235961000	-1.154898000	0.883349000
H	-1.236326000	-1.154849000	-0.883368000

Compound 17

C	0.292346000	-0.506343000	0.000000000
C	-0.292346000	0.506343000	0.000000000
F	-0.921967000	1.596847000	0.000000000
F	0.921967000	-1.596847000	0.000000000

Compound 18

F	-1.462357000	-0.109200000	-0.625152000
C	-0.092772000	-0.096182000	-0.601262000
C	0.358857000	1.323803000	-0.633895000
C	0.357617000	-0.833930000	0.612932000
H	0.234017000	-0.617145000	-1.502505000
H	1.444146000	1.376875000	-0.663379000
H	-0.035749000	1.831806000	-1.508847000
H	0.012101000	1.844500000	0.256172000
H	1.442846000	-0.886904000	0.644735000
H	-0.037854000	-1.845323000	0.615945000
H	0.010848000	-0.322504000	1.508353000

Compound 19

C	0.018299000	0.190342000	-0.344853000
C	1.266065000	1.014625000	-0.311495000
C	-1.205738000	1.034953000	-0.506692000
F	0.100012000	-0.612967000	-1.462028000
C	-0.082434000	-0.720474000	0.837363000
H	2.139863000	0.371117000	-0.252363000
H	1.263153000	1.675324000	0.551635000
H	1.340241000	1.620094000	-1.210963000
H	-2.088383000	0.405905000	-0.586492000
H	-1.327789000	1.696449000	0.347171000
H	-1.128078000	1.640715000	-1.405685000
H	0.793882000	-1.360824000	0.893791000
H	-0.151044000	-0.145044000	1.756960000
H	-0.966250000	-1.347414000	0.754155000

Compound 20

C	0.972910000	0.111002000	-0.124799000
C	0.187351000	-0.703291000	0.616825000
C	-1.153030000	-0.364012000	0.420941000
C	-1.144534000	0.674089000	-0.457150000
N	0.131467000	0.949374000	-0.776402000
F	0.615964000	-1.663224000	1.399199000
H	2.038650000	0.151686000	-0.231479000
H	-2.010302000	-0.826989000	0.870448000
H	-1.959983000	1.232884000	-0.874194000
H	0.421106000	1.668681000	-1.404089000

Equilibrium Cartesian coordinates of compounds 1-20 optimized at the TPSSh/pc-3 level of theory in the gas phase

Compound 1

C	-0.003384000	-0.037183000	0.052656000
F	1.152333000	0.644729000	0.031611000
F	-1.015433000	0.843951000	0.031595000

F	-0.072399000	-0.788258000	-1.057401000
H	-0.060917000	-0.663240000	0.941439000

Compound 2

C	-0.034980000	-0.060557000	-0.000005000
F	0.660879000	1.144088000	-0.000247000
H	0.686406000	-0.878204000	-0.000172000
H	-0.655918000	-0.102657000	0.895342000
H	-0.656386000	-0.102671000	-0.895019000

Compound 3

C	-1.070349000	0.236836000	-0.009917000
F	-1.692648000	-0.218896000	1.072854000
F	-1.688516000	-0.261718000	-1.102553000
F	0.186306000	-0.258245000	-0.023424000
O	-1.092250000	1.584343000	0.023673000
H	-0.643543000	1.926681000	-0.760634000

Compound 4

C	0.549582000	-0.317854000	-0.115250000
C	-0.747509000	0.442849000	-0.098139000
F	1.612787000	0.552507000	-0.094137000
F	0.657691000	-1.100858000	1.008847000
H	0.683771000	-0.971536000	-0.978916000
H	-1.583250000	-0.256260000	-0.095370000
H	-0.814383000	1.074724000	-0.983287000
H	-0.793591000	1.065128000	0.794751000

Compound 5

C	-0.775194000	0.010853000	-0.029451000
C	0.735513000	-0.006620000	-0.052400000
F	1.231606000	-0.073593000	1.257846000
H	-1.143276000	0.876647000	0.521400000
H	-1.156865000	0.063919000	-1.051508000
H	1.125689000	-0.877363000	-0.583217000
H	-1.164754000	-0.894098000	0.437246000
H	1.147182000	0.900154000	-0.499917000

Compound 6

C	-0.235886000	-0.648525000	0.000000000
C	0.425971000	0.497841000	0.000000000
F	-1.574346000	-0.712503000	0.000000000
F	-0.187791000	1.689046000	0.000000000
H	0.257901000	-1.608854000	0.000000000
H	1.504550000	0.550296000	0.000000000

Compound 7

C	-0.221904000	-0.558797000	0.000000000
C	0.416767000	0.595721000	0.000000000
F	-1.568851000	-0.633165000	0.000000000
H	0.231200000	-1.541110000	0.000000000
H	1.496633000	0.598672000	0.000000000
H	-0.109545000	1.539478000	0.000000000

Compound 8

C	-0.314875000	-0.581334000	0.000000000
C	0.314875000	0.581334000	0.000000000
F	-1.661148000	-0.618652000	0.000000000
F	1.661148000	0.618652000	0.000000000
H	0.155414000	-1.553610000	0.000000000
H	-0.155414000	1.553610000	0.000000000

Compound 9

C	-0.241146000	-0.469877000	0.000000000
C	0.417894000	0.671660000	0.000000000
F	-1.559065000	-0.591363000	0.000000000
F	0.312676000	-1.671950000	0.000000000
H	1.495165000	0.666981000	0.000000000
H	-0.124823000	1.602249000	0.000000000

Compound 10

N	0.595964000	1.032210000	0.000000000
C	0.018584000	0.032188000	0.000000000
F	-0.614549000	-1.064398000	0.000000000

Compound 11

F	0.420489000	-0.191232000	0.000000000
H	-0.420489000	0.191232000	0.000000000

Compound 12

C	-0.551214000	0.060305000	0.000000000
O	0.615570000	0.185498000	0.000000000
F	-1.440324000	1.036265000	0.000000000
F	-1.213032000	-1.082066000	0.000000000

Compound 13

B	-0.450831000	0.132353000	-0.206040000
F	-0.670495000	1.372594000	0.174460000
F	0.645288000	-0.488102000	0.174388000

F	-1.327963000	-0.487847000	-0.965808000

Compound 14

Si	-0.968093000	0.234122000	0.035954000
H	-1.686800000	-0.273648000	1.221897000
F	-1.723173000	-0.299691000	-1.273606000
H	0.418435000	-0.273341000	0.008032000
H	-0.984368000	1.710558000	0.008723000

Compound 15

F	-0.574710000	1.495914000	0.006329000
P	-0.728086000	-0.051600000	0.275545000
F	0.778732000	-0.457420000	0.040243000
F	-1.279936000	-0.457894000	-1.146118000

Compound 16

C	0.117871000	0.168484000	-0.000251000
C	0.709138000	1.212973000	-0.000316000
C	-0.618736000	-1.085126000	-0.000133000
F	0.251983000	-2.178490000	-0.000329000
H	1.238815000	2.134282000	-0.000359000
H	-1.245203000	-1.166984000	0.890473000
H	-1.245568000	-1.166939000	-0.890486000

Compound 17

C	0.296327000	-0.513238000	0.000000000
C	-0.296327000	0.513238000	0.000000000
F	-0.938764000	1.625938000	0.000000000
F	0.938764000	-1.625938000	0.000000000

Compound 18

F	-1.499892000	-0.116672000	-0.638119000
C	-0.086823000	-0.097119000	-0.602877000
C	0.362230000	1.349570000	-0.642018000
C	0.360964000	-0.853838000	0.631203000
H	0.233337000	-0.620365000	-1.508079000
H	1.452245000	1.402933000	-0.675837000
H	-0.035245000	1.849076000	-1.525608000
H	0.016315000	1.880124000	0.246441000
H	1.450916000	-0.910719000	0.661093000
H	-0.037376000	-1.868468000	0.622540000
H	0.015030000	-0.348726000	1.534361000

Compound 19

C	0.017876000	0.194839000	-0.338591000
C	1.290745000	1.027561000	-0.315709000

C	-1.229292000	1.048126000	-0.513927000
F	0.102277000	-0.635643000	-1.493283000
C	-0.084120000	-0.741146000	0.856254000
H	2.166033000	0.380007000	-0.256725000
H	1.293503000	1.693902000	0.548758000
H	1.363871000	1.632487000	-1.219934000
H	-2.113169000	0.415120000	-0.597219000
H	-1.358168000	1.712544000	0.342407000
H	-1.148714000	1.656315000	-1.415352000
H	0.795455000	-1.383215000	0.910904000
H	-0.153624000	-0.168280000	1.782752000
H	-0.970872000	-1.369814000	0.770165000

Compound 20

C	0.990402000	0.110639000	-0.125672000
C	0.188260000	-0.711622000	0.623799000
C	-1.166622000	-0.369910000	0.426854000
C	-1.161760000	0.681979000	-0.462652000
N	0.135707000	0.961785000	-0.787184000
F	0.629551000	-1.695886000	1.425873000
H	2.058204000	0.156740000	-0.237066000
H	-2.026494000	-0.834199000	0.877650000
H	-1.976070000	1.245307000	-0.883606000
H	0.428422000	1.685366000	-1.418697000

Equilibrium Cartesian coordinates of compounds 1-20 optimized at the B3LYP/pc-3 level of theory in the gas phase

Compound 1

C	-0.003290000	-0.036187000	0.051532000
F	1.151083000	0.643640000	0.031768000
F	-1.014414000	0.842671000	0.031766000
F	-0.072307000	-0.787387000	-1.055573000
H	-0.060872000	-0.662737000	0.940408000

Compound 2

C	-0.033550000	-0.058116000	-0.000036000
F	0.661415000	1.145059000	-0.000180000
H	0.683299000	-0.877949000	-0.000185000
H	-0.655319000	-0.104438000	0.892841000
H	-0.655843000	-0.104557000	-0.892540000

Compound 3

C	-1.070444000	0.235951000	-0.009779000
F	-1.691933000	-0.219428000	1.071629000
F	-1.688378000	-0.261462000	-1.100297000
F	0.184285000	-0.257949000	-0.022428000
O	-1.090398000	1.581677000	0.020420000

H	-0.644132000	1.930212000	-0.759545000

Compound 4

C	0.550462000	-0.317106000	-0.113368000
C	-0.746087000	0.441842000	-0.098417000
F	1.612110000	0.551079000	-0.094113000
F	0.658206000	-1.100222000	1.007495000
H	0.680589000	-0.969135000	-0.978074000
H	-1.581590000	-0.255499000	-0.095780000
H	-0.814018000	1.073243000	-0.982201000
H	-0.794574000	1.064499000	0.792958000

Compound 5

C	-0.773746000	0.010966000	-0.032630000
C	0.735611000	-0.006750000	-0.047625000
F	1.227183000	-0.075486000	1.261834000
H	-1.144889000	0.875172000	0.516364000
H	-1.152221000	0.064670000	-1.054313000
H	1.126622000	-0.874558000	-0.580059000
H	-1.166757000	-0.892385000	0.431602000
H	1.148096000	0.898271000	-0.495173000

Compound 6

C	-0.235079000	-0.647309000	0.000000000
C	0.425344000	0.496544000	0.000000000
F	-1.572141000	-0.709570000	0.000000000
F	-0.189235000	1.685639000	0.000000000
H	0.258809000	-1.606176000	0.000000000
H	1.502701000	0.548172000	0.000000000

Compound 7

C	-0.222585000	-0.557664000	0.000000000
C	0.416972000	0.593636000	0.000000000
F	-1.567434000	-0.630898000	0.000000000
H	0.232477000	-1.538027000	0.000000000
H	1.495269000	0.598031000	0.000000000
H	-0.110399000	1.535722000	0.000000000

Compound 8

C	-0.315676000	-0.579138000	0.000000000
C	0.315676000	0.579138000	0.000000000
F	-1.659916000	-0.616931000	0.000000000
F	1.659916000	0.616931000	0.000000000
H	0.157062000	-1.549210000	0.000000000
H	-0.157062000	1.549210000	0.000000000

Compound 9

C	-0.240629000	-0.468982000	0.000000000
C	0.416883000	0.669908000	0.000000000
F	-1.556945000	-0.589582000	0.000000000
F	0.313157000	-1.669225000	0.000000000
H	1.492953000	0.665826000	0.000000000
H	-0.124718000	1.599754000	0.000000000

Compound 10

N	0.593911000	1.028654000	0.000000000
C	0.019136000	0.033143000	0.000000000
F	-0.613047000	-1.061798000	0.000000000

Compound 11

F	0.419623000	-0.190838000	0.000000000
H	-0.419623000	0.190838000	0.000000000

Compound 12

C	-0.551727000	0.060250000	0.000000000
O	0.611284000	0.185038000	0.000000000
F	-1.437853000	1.035852000	0.000000000
F	-1.210705000	-1.081138000	0.000000000

Compound 13

B	-0.450946000	0.132272000	-0.205862000
F	-0.670048000	1.370291000	0.173685000
F	0.643276000	-0.486914000	0.173618000
F	-1.326283000	-0.486651000	-0.964441000

Compound 14

Si	-0.968186000	0.234109000	0.035849000
H	-1.686294000	-0.273437000	1.221498000
F	-1.723190000	-0.299630000	-1.273518000
H	0.417865000	-0.272993000	0.008331000
H	-0.984196000	1.709951000	0.008840000

Compound 15

F	-0.574466000	1.495981000	0.006738000
P	-0.728021000	-0.051384000	0.275000000
F	0.778706000	-0.457258000	0.040414000
F	-1.280218000	-0.458341000	-1.146154000

Compound 16

C	0.119650000	0.168326000	-0.000248000
C	0.707899000	1.211130000	-0.000314000
C	-0.615594000	-1.085186000	-0.000133000
F	0.250585000	-2.179018000	-0.000329000
H	1.235376000	2.132053000	-0.000361000
H	-1.244627000	-1.164577000	0.887714000
H	-1.244990000	-1.164529000	-0.887728000

Compound 17

C	0.295572000	-0.511931000	0.000000000
C	-0.295572000	0.511931000	0.000000000
F	-0.937481000	1.623718000	0.000000000
F	0.937481000	-1.623718000	0.000000000

Compound 18

F	-1.499026000	-0.116382000	-0.637617000
C	-0.088057000	-0.096004000	-0.600950000
C	0.362110000	1.349025000	-0.642583000
C	0.360845000	-0.854055000	0.630448000
H	0.234181000	-0.618376000	-1.504635000
H	1.450501000	1.404179000	-0.676670000
H	-0.034227000	1.847644000	-1.525624000
H	0.016924000	1.882666000	0.242892000
H	1.449171000	-0.912061000	0.661756000
H	-0.036356000	-1.867768000	0.621290000
H	0.015634000	-0.353072000	1.534791000

Compound 19

C	0.017945000	0.194740000	-0.338918000
C	1.290366000	1.027459000	-0.315087000
C	-1.228903000	1.047743000	-0.514228000
F	0.102351000	-0.634789000	-1.492254000
C	-0.084131000	-0.740476000	0.856096000
H	2.165754000	0.382048000	-0.259255000
H	1.296304000	1.692022000	0.548809000
H	1.364301000	1.633812000	-1.216693000
H	-2.112156000	0.416177000	-0.597782000
H	-1.360232000	1.713253000	0.338888000
H	-1.150696000	1.654296000	-1.415324000
H	0.792483000	-1.384359000	0.910913000
H	-0.151645000	-0.170662000	1.782769000
H	-0.969940000	-1.368462000	0.772565000

Compound 20

C	0.989008000	0.110809000	-0.125721000
C	0.189089000	-0.710850000	0.623090000
C	-1.165468000	-0.368728000	0.425777000

C	-1.160940000	0.682122000	-0.462829000
N	0.135728000	0.962598000	-0.787872000
F	0.628767000	-1.694011000	1.424341000
H	2.055977000	0.153647000	-0.234300000
H	-2.023723000	-0.832998000	0.876447000
H	-1.976621000	1.242658000	-0.881330000
H	0.427784000	1.684951000	-1.418303000

Equilibrium Cartesian coordinates of compounds 1-20 optimized at the X3LYP/pc-3 level of theory in the gas phase

Compound 1

C	-0.003281000	-0.036094000	0.051353000
F	1.149585000	0.642990000	0.031527000
F	-1.013059000	0.841758000	0.031524000
F	-0.072203000	-0.786251000	-1.054450000
H	-0.060842000	-0.662404000	0.939946000

Compound 2

C	-0.033570000	-0.058153000	-0.000035000
F	0.660799000	1.143992000	-0.000181000
H	0.683362000	-0.877494000	-0.000184000
H	-0.655033000	-0.104115000	0.892691000
H	-0.655557000	-0.104231000	-0.892391000

Compound 3

C	-1.070414000	0.235940000	-0.009829000
F	-1.691163000	-0.218994000	1.070287000
F	-1.687579000	-0.260715000	-1.098937000
F	0.182706000	-0.257209000	-0.022436000
O	-1.090230000	1.580422000	0.020135000
H	-0.644321000	1.929556000	-0.759220000

Compound 4

C	0.550093000	-0.316863000	-0.113323000
C	-0.745438000	0.441398000	-0.098520000
F	1.610061000	0.550599000	-0.093628000
F	0.657448000	-1.098468000	1.006490000
H	0.680276000	-0.968790000	-0.977828000
H	-1.580808000	-0.255747000	-0.095650000
H	-0.813312000	1.072864000	-0.981984000
H	-0.793220000	1.063707000	0.792942000

Compound 5

C	-0.771071000	-0.053884000	-0.036842000
C	0.733408000	0.054400000	-0.043224000
F	1.220464000	0.036626000	1.268071000

H	-1.216609000	0.779757000	0.503645000
H	-1.146388000	-0.039161000	-1.060748000
H	1.198278000	-0.781260000	-0.567263000
H	-1.089616000	-0.983673000	0.431956000
H	1.071435000	0.987095000	-0.495595000

Compound 6

C	-0.234788000	-0.646956000	0.000000000
C	0.425182000	0.496117000	0.000000000
F	-1.570586000	-0.707676000	0.000000000
F	-0.190097000	1.683347000	0.000000000
H	0.258461000	-1.605832000	0.000000000
H	1.502227000	0.548301000	0.000000000

Compound 7

C	-0.222471000	-0.557378000	0.000000000
C	0.416744000	0.593190000	0.000000000
F	-1.565973000	-0.629867000	0.000000000
H	0.232310000	-1.537554000	0.000000000
H	1.494778000	0.597652000	0.000000000
H	-0.111088000	1.534758000	0.000000000

Compound 8

C	-0.315618000	-0.578675000	0.000000000
C	0.315618000	0.578675000	0.000000000
F	-1.658387000	-0.616060000	0.000000000
F	1.658387000	0.616060000	0.000000000
H	0.157378000	-1.548397000	0.000000000
H	-0.157378000	1.548397000	0.000000000

Compound 9

C	-0.240466000	-0.468700000	0.000000000
C	0.416616000	0.669446000	0.000000000
F	-1.555324000	-0.589306000	0.000000000
F	0.312585000	-1.667683000	0.000000000
H	1.492457000	0.665021000	0.000000000
H	-0.125167000	1.598922000	0.000000000

Compound 10

N	0.593390000	1.027750000	0.000000000
C	0.019109000	0.033096000	0.000000000
F	-0.612499000	-1.060848000	0.000000000

Compound 11

F	0.419289000	-0.190687000	0.000000000

H	-0.419289000	0.190687000	0.000000000

Compound 12

C	-0.552107000	0.060209000	0.000000000
O	0.610073000	0.184908000	0.000000000
F	-1.436917000	1.034625000	0.000000000
F	-1.210050000	-1.079741000	0.000000000

Compound 13

B	-0.450980000	0.132250000	-0.205803000
F	-0.669830000	1.369125000	0.173305000
F	0.642241000	-0.486321000	0.173234000
F	-1.325432000	-0.486056000	-0.963735000

Compound 14

Si	-0.968124000	0.234156000	0.035962000
H	-1.686321000	-0.273466000	1.220996000
F	-1.722640000	-0.299234000	-1.272565000
H	0.417446000	-0.273021000	0.008057000
H	-0.984361000	1.709565000	0.008550000

Compound 15

F	-0.574151000	1.493886000	0.006240000
P	-0.727897000	-0.051284000	0.274766000
F	0.776819000	-0.456243000	0.039824000
F	-1.278771000	-0.457359000	-1.144831000

Compound 16

C	0.119592000	0.167926000	-0.000248000
C	0.707496000	1.210146000	-0.000314000
C	-0.615186000	-1.085255000	-0.000133000
F	0.250282000	-2.177101000	-0.000329000
H	1.234829000	2.130932000	-0.000361000
H	-1.244175000	-1.164248000	0.887457000
H	-1.244537000	-1.164201000	-0.887471000

Compound 17

C	0.295372000	-0.511584000	0.000000000
C	-0.295372000	0.511584000	0.000000000
F	-0.936740000	1.622434000	0.000000000
F	0.936740000	-1.622434000	0.000000000

Compound 18

F	-1.497005000	-0.115955000	-0.636876000
C	-0.088014000	-0.096078000	-0.601077000
C	0.361996000	1.347730000	-0.642144000
C	0.360732000	-0.853027000	0.629545000
H	0.234197000	-0.618353000	-1.504596000
H	1.450152000	1.402953000	-0.676092000
H	-0.034521000	1.846795000	-1.524583000
H	0.016639000	1.880333000	0.243694000
H	1.448823000	-0.910948000	0.660983000
H	-0.036649000	-1.866442000	0.621074000
H	0.015352000	-0.351211000	1.533171000

Compound 19

C	0.017975000	0.194557000	-0.339014000
C	1.289224000	1.026800000	-0.315061000
C	-1.227568000	1.047621000	-0.513085000
F	0.101958000	-0.633394000	-1.490701000
C	-0.084149000	-0.740044000	0.854772000
H	2.164444000	0.381792000	-0.255695000
H	1.293139000	1.693997000	0.546544000
H	1.364307000	1.630044000	-1.218447000
H	-2.111238000	0.416707000	-0.595080000
H	-1.357058000	1.713370000	0.339890000
H	-1.150076000	1.653691000	-1.414350000
H	0.793526000	-1.382114000	0.910227000
H	-0.154197000	-0.170710000	1.781266000
H	-0.968487000	-1.369515000	0.769234000

Compound 20

C	0.988237000	0.110773000	-0.125638000
C	0.188985000	-0.710386000	0.622705000
C	-1.164863000	-0.368484000	0.425530000
C	-1.160225000	0.681764000	-0.462575000
N	0.135530000	0.962077000	-0.787418000
F	0.628128000	-1.692567000	1.423163000
H	2.055009000	0.153413000	-0.234036000
H	-2.022861000	-0.832738000	0.876169000
H	-1.975777000	1.242132000	-0.880943000
H	0.427435000	1.684215000	-1.417658000

Equilibrium Cartesian coordinates of compounds 1-20 optimized at the O3LYP/pc-3 level of theory in the gas phase

Compound 1

C	-0.003148000	-0.034670000	0.049410000
F	1.151054000	0.643338000	0.032191000
F	-1.014436000	0.842358000	0.032180000
F	-0.072342000	-0.787677000	-1.055093000

H	-0.060928000	-0.663349000	0.941213000

Compound 2

C	-0.032314000	-0.055948000	-0.000012000
F	0.660341000	1.143145000	-0.000210000
H	0.685093000	-0.879069000	-0.000193000
H	-0.656317000	-0.104022000	0.894707000
H	-0.656801000	-0.104107000	-0.894391000

Compound 3

C	-1.070047000	0.236790000	-0.010475000
F	-1.691424000	-0.217992000	1.070735000
F	-1.687994000	-0.260134000	-1.100769000
F	0.184494000	-0.256617000	-0.023007000
O	-1.091365000	1.582999000	0.022096000
H	-0.644665000	1.923954000	-0.758579000

Compound 4

C	0.553069000	-0.316099000	-0.109600000
C	-0.745866000	0.442696000	-0.096946000
F	1.614164000	0.548041000	-0.094966000
F	0.661094000	-1.101818000	1.005680000
H	0.678395000	-0.968733000	-0.979370000
H	-1.581507000	-0.256664000	-0.096465000
H	-0.813506000	1.072821000	-0.983381000
H	-0.800743000	1.068456000	0.793547000

Compound 5

C	-0.780718000	0.024017000	-0.041598000
C	0.744966000	0.022588000	-0.039296000
F	1.237077000	-0.629546000	1.093617000
H	-1.186412000	1.035674000	-0.025551000
H	-1.186387000	-0.492995000	-0.911332000
H	1.155927000	-0.502513000	-0.904677000
H	-1.140607000	-0.491908000	0.848711000
H	1.156054000	1.034583000	-0.019874000

Compound 6

C	-0.240901000	-0.646648000	0.000000000
C	0.421845000	0.501247000	0.000000000
F	-1.574036000	-0.719892000	0.000000000
F	-0.181231000	1.692456000	0.000000000
H	0.262382000	-1.603714000	0.000000000
H	1.502341000	0.543853000	0.000000000

Compound 7

C	-0.226263000	-0.557828000	0.000000000
C	0.416590000	0.596062000	0.000000000
F	-1.566793000	-0.635317000	0.000000000
H	0.232814000	-1.539667000	0.000000000
H	1.496815000	0.596199000	0.000000000
H	-0.108864000	1.541351000	0.000000000

Compound 8

C	-0.319037000	-0.580429000	0.000000000
C	0.319037000	0.580429000	0.000000000
F	-1.658717000	-0.618831000	0.000000000
F	1.658717000	0.618831000	0.000000000
H	0.155904000	-1.552669000	0.000000000
H	-0.155904000	1.552669000	0.000000000

Compound 9

C	-0.242150000	-0.471615000	0.000000000
C	0.417556000	0.671074000	0.000000000
F	-1.556471000	-0.590723000	0.000000000
F	0.311933000	-1.669385000	0.000000000
H	1.495296000	0.666319000	0.000000000
H	-0.125462000	1.602030000	0.000000000

Compound 10

N	0.595678000	1.031713000	0.000000000
C	0.017930000	0.031054000	0.000000000
F	-0.613608000	-1.062768000	0.000000000

Compound 11

F	0.418288000	-0.190231000	0.000000000
H	-0.418288000	0.190231000	0.000000000

Compound 12

C	-0.552887000	0.060125000	0.000000000
O	0.613130000	0.185236000	0.000000000
F	-1.438144000	1.035335000	0.000000000
F	-1.211099000	-1.080695000	0.000000000

Compound 13

B	-0.450793000	0.132391000	-0.206110000
F	-0.670600000	1.373018000	0.174608000
F	0.645661000	-0.488348000	0.174539000
F	-1.328269000	-0.488063000	-0.966038000

Compound 14

Si	-0.969593000	0.233122000	0.033448000
H	-1.688251000	-0.274894000	1.226713000
F	-1.725655000	-0.301348000	-1.277938000
H	0.423262000	-0.274467000	0.009174000
H	-0.983764000	1.715588000	0.009603000

Compound 15

F	-0.575233000	1.498733000	0.008413000
P	-0.726906000	-0.050509000	0.272881000
F	0.780660000	-0.458941000	0.041871000
F	-1.282521000	-0.460284000	-1.147166000

Compound 16

C	0.121654000	0.164855000	-0.000248000
C	0.709070000	1.213991000	-0.000314000
C	-0.611838000	-1.089780000	-0.000133000
F	0.245344000	-2.183317000	-0.000330000
H	1.234614000	2.138836000	-0.000362000
H	-1.245094000	-1.163216000	0.888937000
H	-1.245451000	-1.163168000	-0.888951000

Compound 17

C	0.296821000	-0.514094000	0.000000000
C	-0.296821000	0.514094000	0.000000000
F	-0.937492000	1.623737000	0.000000000
F	0.937492000	-1.623737000	0.000000000

Compound 18

F	-1.498112000	-0.117303000	-0.639211000
C	-0.093739000	-0.093526000	-0.596666000
C	0.360830000	1.350927000	-0.643136000
C	0.359562000	-0.855483000	0.631819000
H	0.236411000	-0.616342000	-1.501113000
H	1.450507000	1.403277000	-0.677870000
H	-0.032564000	1.846941000	-1.530864000
H	0.017814000	1.894146000	0.238935000
H	1.449176000	-0.912648000	0.660378000
H	-0.034694000	-1.871958000	0.618065000
H	0.016509000	-0.362234000	1.542762000

Compound 19

C	0.017961000	0.192112000	-0.342760000
C	1.290743000	1.027100000	-0.315340000
C	-1.229565000	1.047662000	-0.515265000

F	0.101855000	-0.633851000	-1.491250000
C	-0.083853000	-0.741068000	0.855886000
H	2.168604000	0.382841000	-0.259444000
H	1.297473000	1.691394000	0.550032000
H	1.367434000	1.636568000	-1.216116000
H	-2.115879000	0.418039000	-0.598219000
H	-1.361005000	1.714543000	0.338013000
H	-1.154164000	1.655648000	-1.417214000
H	0.793326000	-1.386138000	0.913002000
H	-0.151042000	-0.171017000	1.783621000
H	-0.970089000	-1.371034000	0.775556000

Compound 20

C	0.988452000	0.111790000	-0.126513000
C	0.188316000	-0.712393000	0.624447000
C	-1.165302000	-0.369955000	0.426803000
C	-1.159136000	0.681956000	-0.462811000
N	0.135397000	0.960410000	-0.786001000
F	0.628482000	-1.692816000	1.423351000
H	2.057304000	0.157282000	-0.237463000
H	-2.026493000	-0.833830000	0.877338000
H	-1.974948000	1.245157000	-0.883556000
H	0.427526000	1.682596000	-1.416295000

Equilibrium Cartesian coordinates of compounds 1-20 optimized at the tHCTHhyb/pc-3 level of theory in the gas phase

Compound 1

C	-0.003206000	-0.035349000	0.050314000
F	1.149957000	0.643340000	0.031325000
F	-1.013354000	0.842154000	0.031316000
F	-0.072221000	-0.786340000	-1.054973000
H	-0.060975000	-0.663805000	0.941917000

Compound 2

C	-0.032901000	-0.057046000	-0.000035000
F	0.660263000	1.143125000	-0.000253000
H	0.685671000	-0.878966000	-0.000121000
H	-0.656258000	-0.103576000	0.895035000
H	-0.656773000	-0.103537000	-0.894727000

Compound 3

C	-1.070422000	0.236474000	-0.009806000
F	-1.691172000	-0.218659000	1.070298000
F	-1.687504000	-0.259561000	-1.099807000
F	0.183414000	-0.256075000	-0.022931000
O	-1.091110000	1.581602000	0.021680000
H	-0.644207000	1.925220000	-0.759434000

Compound 4

C	0.552645000	-0.317333000	-0.111817000
C	-0.746716000	0.442495000	-0.097982000
F	1.611353000	0.549999000	-0.093678000
F	0.658623000	-1.099270000	1.006575000
H	0.681484000	-0.970989000	-0.980078000
H	-1.583101000	-0.257118000	-0.095664000
H	-0.813860000	1.074513000	-0.984013000
H	-0.795330000	1.066404000	0.795158000

Compound 5

C	-0.781965000	0.024354000	-0.042185000
C	0.746088000	0.023693000	-0.041207000
F	1.232305000	-0.630169000	1.094587000
H	-1.187972000	1.036401000	-0.025187000
H	-1.187950000	-0.493767000	-0.911728000
H	1.157900000	-0.503068000	-0.905236000
H	-1.136531000	-0.492896000	0.850535000
H	1.158025000	1.035352000	-0.019580000

Compound 6

C	-0.238400000	-0.647668000	0.000000000
C	0.423968000	0.499570000	0.000000000
F	-1.572312000	-0.711973000	0.000000000
F	-0.187205000	1.686958000	0.000000000
H	0.260061000	-1.607256000	0.000000000
H	1.504287000	0.547670000	0.000000000

Compound 7

C	-0.224881000	-0.558307000	0.000000000
C	0.416958000	0.595383000	0.000000000
F	-1.566163000	-0.631713000	0.000000000
H	0.232654000	-1.540731000	0.000000000
H	1.497510000	0.596909000	0.000000000
H	-0.111776000	1.539259000	0.000000000

Compound 8

C	-0.317927000	-0.580422000	0.000000000
C	0.317927000	0.580422000	0.000000000
F	-1.658253000	-0.616625000	0.000000000
F	1.658253000	0.616625000	0.000000000
H	0.157642000	-1.552404000	0.000000000
H	-0.157642000	1.552404000	0.000000000

Compound 9

C	-0.241852000	-0.471100000	0.000000000
C	0.417482000	0.670948000	0.000000000
F	-1.555845000	-0.590410000	0.000000000
F	0.311891000	-1.668685000	0.000000000
H	1.495700000	0.665150000	0.000000000
H	-0.126675000	1.601797000	0.000000000

Compound 10

N	0.594808000	1.030207000	0.000000000
C	0.018276000	0.031653000	0.000000000
F	-0.613084000	-1.061861000	0.000000000

Compound 11

F	0.418781000	-0.190456000	0.000000000
H	-0.418781000	0.190456000	0.000000000

Compound 12

C	-0.552697000	0.060146000	0.000000000
O	0.611897000	0.185104000	0.000000000
F	-1.437533000	1.034550000	0.000000000
F	-1.210668000	-1.079798000	0.000000000

Compound 13

B	-0.450858000	0.132342000	-0.205995000
F	-0.670373000	1.371894000	0.174223000
F	0.644687000	-0.487757000	0.174160000
F	-1.327457000	-0.487481000	-0.965388000

Compound 14

Si	-0.969115000	0.233572000	0.034455000
H	-1.685995000	-0.273504000	1.222692000
F	-1.723622000	-0.299848000	-1.274492000
H	0.418573000	-0.272961000	0.009068000
H	-0.983841000	1.710741000	0.009277000

Compound 15

F	-0.574540000	1.494766000	0.006821000
P	-0.727265000	-0.050925000	0.273844000
F	0.777482000	-0.456886000	0.040471000
F	-1.279676000	-0.457957000	-1.145136000

Compound 16

C	0.120991000	0.166610000	-0.000246000
C	0.709031000	1.213459000	-0.000314000
C	-0.614238000	-1.089586000	-0.000132000
F	0.248173000	-2.180059000	-0.000330000
H	1.236371000	2.137049000	-0.000364000
H	-1.245835000	-1.164661000	0.889918000
H	-1.246193000	-1.164612000	-0.889932000

Compound 17

C	0.296520000	-0.513573000	0.000000000
C	-0.296520000	0.513573000	0.000000000
F	-0.937164000	1.623167000	0.000000000
F	0.937164000	-1.623167000	0.000000000

Compound 18

F	-1.499009000	-0.116631000	-0.638048000
C	-0.093106000	-0.096973000	-0.602631000
C	0.361900000	1.348992000	-0.642177000
C	0.360635000	-0.853687000	0.630622000
H	0.233029000	-0.620530000	-1.508364000
H	1.452792000	1.399667000	-0.673699000
H	-0.032155000	1.850759000	-1.527222000
H	0.015862000	1.882562000	0.245893000
H	1.451467000	-0.907234000	0.659333000
H	-0.034286000	-1.870710000	0.623191000
H	0.014571000	-0.350419000	1.536199000

Compound 19

C	0.018180000	0.191079000	-0.344002000
C	1.290747000	1.027080000	-0.315862000
C	-1.229307000	1.047798000	-0.514911000
F	0.102292000	-0.635414000	-1.493140000
C	-0.084094000	-0.741381000	0.855894000
H	2.168506000	0.380900000	-0.258999000
H	1.292555000	1.690526000	0.551803000
H	1.365222000	1.636935000	-1.217900000
H	-2.116142000	0.416829000	-0.596060000
H	-1.355336000	1.713302000	0.341966000
H	-1.151818000	1.657066000	-1.417162000
H	0.796132000	-1.383999000	0.914697000
H	-0.154269000	-0.165727000	1.781452000
H	-0.970867000	-1.372193000	0.772725000

Compound 20

C	0.990175000	0.111401000	-0.126301000
C	0.189417000	-0.713232000	0.625082000
C	-1.167081000	-0.370393000	0.427293000

C	-1.160746000	0.682595000	-0.463241000
N	0.135894000	0.961761000	-0.787176000
F	0.629453000	-1.693690000	1.424023000
H	2.059275000	0.155976000	-0.236492000
H	-2.028019000	-0.834730000	0.878203000
H	-1.976886000	1.245661000	-0.883850000
H	0.428117000	1.684851000	-1.418241000

Equilibrium Cartesian coordinates of compounds 1-20 optimized at the B97-1/pc-3 level of theory in the gas phase

Compound 1

C	-0.003209000	-0.035352000	0.050325000
F	1.149371000	0.643008000	0.031287000
F	-1.012839000	0.841723000	0.031277000
F	-0.072181000	-0.785908000	-1.054450000
H	-0.060942000	-0.663472000	0.941461000

Compound 2

C	-0.032813000	-0.056759000	-0.000011000
F	0.660217000	1.142917000	-0.000221000
H	0.685304000	-0.878722000	-0.000189000
H	-0.656116000	-0.103686000	0.894765000
H	-0.656591000	-0.103751000	-0.894444000

Compound 3

C	-1.070307000	0.235645000	-0.010011000
F	-1.690848000	-0.218942000	1.069735000
F	-1.687265000	-0.259402000	-1.099038000
F	0.182630000	-0.255902000	-0.022762000
O	-1.091036000	1.582393000	0.021537000
H	-0.644176000	1.925207000	-0.759461000

Compound 4

C	0.554246000	-0.318324000	-0.111916000
C	-0.747356000	0.442771000	-0.098124000
F	1.611372000	0.548931000	-0.092698000
F	0.659754000	-1.098413000	1.006272000
H	0.681581000	-0.971446000	-0.980678000
H	-1.583745000	-0.256749000	-0.095538000
H	-0.814447000	1.074981000	-0.983951000
H	-0.796306000	1.066951000	0.795134000

Compound 5

C	-0.782636000	0.024296000	-0.042085000
C	0.747159000	0.023635000	-0.041107000

F	1.232100000	-0.630006000	1.094336000
H	-1.188325000	1.036391000	-0.025258000
H	-1.188308000	-0.493674000	-0.911770000
H	1.158275000	-0.502872000	-0.905130000
H	-1.136765000	-0.493032000	0.850738000
H	1.158400000	1.035162000	-0.019722000

Compound 6

C	-0.238037000	-0.647777000	0.000000000
C	0.424268000	0.499314000	0.000000000
F	-1.571772000	-0.710930000	0.000000000
F	-0.187839000	1.685965000	0.000000000
H	0.259725000	-1.607205000	0.000000000
H	1.504054000	0.547934000	0.000000000

Compound 7

C	-0.225204000	-0.558517000	0.000000000
C	0.417054000	0.595253000	0.000000000
F	-1.565960000	-0.631652000	0.000000000
H	0.232675000	-1.540235000	0.000000000
H	1.497341000	0.597034000	0.000000000
H	-0.111606000	1.538917000	0.000000000

Compound 8

C	-0.318128000	-0.580218000	0.000000000
C	0.318128000	0.580218000	0.000000000
F	-1.658124000	-0.616716000	0.000000000
F	1.658124000	0.616716000	0.000000000
H	0.157539000	-1.551722000	0.000000000
H	-0.157539000	1.551722000	0.000000000

Compound 9

C	-0.241891000	-0.471167000	0.000000000
C	0.417526000	0.671023000	0.000000000
F	-1.555320000	-0.590582000	0.000000000
F	0.311480000	-1.668316000	0.000000000
H	1.495521000	0.665116000	0.000000000
H	-0.126615000	1.601625000	0.000000000

Compound 10

N	0.594585000	1.029820000	0.000000000
C	0.018394000	0.031857000	0.000000000
F	-0.612978000	-1.061678000	0.000000000

Compound 11

F	0.418396000	-0.190280000	0.000000000

H	-0.418396000	0.190280000	0.000000000

Compound 12

C	-0.553538000	0.060055000	0.000000000
O	0.611596000	0.185072000	0.000000000
F	-1.436914000	1.034170000	0.000000000
F	-1.210144000	-1.079296000	0.000000000

Compound 13

B	-0.450940000	0.132288000	-0.205868000
F	-0.670172000	1.370933000	0.173881000
F	0.643830000	-0.487249000	0.173807000
F	-1.326720000	-0.486973000	-0.964820000

Compound 14

Si	-0.969054000	0.233511000	0.034432000
H	-1.686500000	-0.273620000	1.223231000
F	-1.723970000	-0.300137000	-1.275004000
H	0.419417000	-0.273215000	0.008951000
H	-0.983893000	1.711460000	0.009391000

Compound 15

F	-0.574352000	1.494365000	0.006602000
P	-0.727570000	-0.051069000	0.274122000
F	0.777191000	-0.456591000	0.040199000
F	-1.279269000	-0.457706000	-1.144924000

Compound 16

C	0.121791000	0.167288000	-0.000247000
C	0.709376000	1.213918000	-0.000315000
C	-0.614625000	-1.091092000	-0.000131000
F	0.247482000	-2.179868000	-0.000331000
H	1.236199000	2.137443000	-0.000363000
H	-1.245784000	-1.164768000	0.889758000
H	-1.246140000	-1.164721000	-0.889772000

Compound 17

C	0.296367000	-0.513308000	0.000000000
C	-0.296367000	0.513308000	0.000000000
F	-0.937133000	1.623114000	0.000000000
F	0.937133000	-1.623114000	0.000000000

Compound 18

F	-1.498158000	-0.116752000	-0.638258000

C	-0.093982000	-0.097417000	-0.603400000
C	0.362158000	1.350262000	-0.642718000
C	0.360890000	-0.854790000	0.631452000
H	0.232902000	-0.620577000	-1.508446000
H	1.452826000	1.401046000	-0.674467000
H	-0.032424000	1.851958000	-1.527433000
H	0.015919000	1.883371000	0.245430000
H	1.451499000	-0.908587000	0.660147000
H	-0.034555000	-1.871492000	0.624125000
H	0.014625000	-0.351225000	1.536668000

Compound 19

C	0.018206000	0.190508000	-0.344743000
C	1.292080000	1.028251000	-0.315439000
C	-1.230797000	1.048552000	-0.515304000
F	0.102393000	-0.634489000	-1.492402000
C	-0.084062000	-0.743118000	0.856562000
H	2.170038000	0.382791000	-0.255418000
H	1.291892000	1.693699000	0.550474000
H	1.367638000	1.635708000	-1.218899000
H	-2.117626000	0.417532000	-0.595403000
H	-1.356092000	1.714963000	0.340696000
H	-1.153572000	1.656092000	-1.418588000
H	0.794781000	-1.387806000	0.911878000
H	-0.150744000	-0.168138000	1.782588000
H	-0.972333000	-1.371744000	0.774499000

Compound 20

C	0.990623000	0.111018000	-0.126007000
C	0.189997000	-0.713498000	0.625267000
C	-1.167497000	-0.370466000	0.427383000
C	-1.161296000	0.682708000	-0.463299000
N	0.136039000	0.962508000	-0.787818000
F	0.629470000	-1.693794000	1.424110000
H	2.059476000	0.155463000	-0.236072000
H	-2.028123000	-0.834771000	0.878244000
H	-1.977331000	1.245458000	-0.883648000
H	0.428241000	1.685573000	-1.418860000

Equilibrium Cartesian coordinates of compounds 1-20 optimized at the M06HF/pc-3 level of theory in the gas phase

Compound 1

C	-0.003539000	-0.037596000	0.053039000
F	1.144691000	0.641339000	0.030938000
F	-1.008653000	0.839500000	0.030921000
F	-0.071829000	-0.783308000	-1.051498000
H	-0.060469000	-0.659935000	0.936500000

Compound 2

C	-0.037642000	-0.065012000	-0.000312000
F	0.657858000	1.137697000	0.000116000
H	0.686439000	-0.872779000	-0.000127000
H	-0.652901000	-0.099722000	0.892825000
H	-0.653753000	-0.100186000	-0.892603000

Compound 3

C	-1.071648000	0.235860000	-0.008142000
F	-1.689091000	-0.222621000	1.067039000
F	-1.685514000	-0.255082000	-1.092709000
F	0.176248000	-0.250654000	-0.021740000
O	-1.088087000	1.573374000	0.014945000
H	-0.642909000	1.928123000	-0.759394000

Compound 4

C	0.547697000	-0.318574000	-0.117962000
C	-0.750466000	0.442625000	-0.101036000
F	1.599381000	0.552680000	-0.091626000
F	0.650292000	-1.090289000	1.004423000
H	0.683749000	-0.968633000	-0.974585000
H	-1.578611000	-0.259104000	-0.093717000
H	-0.808922000	1.073304000	-0.982584000
H	-0.778022000	1.056693000	0.795588000

Compound 5

C	-0.777680000	0.011419000	-0.031457000
C	0.736648000	-0.006960000	-0.054279000
F	1.219062000	-0.073274000	1.253966000
H	-1.135616000	0.878738000	0.516828000
H	-1.152716000	0.061879000	-1.052020000
H	1.122765000	-0.878139000	-0.576100000
H	-1.158055000	-0.891994000	0.437578000
H	1.145492000	0.898232000	-0.494517000

Compound 6

C	-0.229641000	-0.644987000	0.000000000
C	0.426081000	0.490544000	0.000000000
F	-1.566624000	-0.695481000	0.000000000
F	-0.198652000	1.673660000	0.000000000
H	0.259679000	-1.601805000	0.000000000
H	1.499556000	0.545369000	0.000000000

Compound 7

C	-0.216095000	-0.555753000	0.000000000

C	0.418924000	0.590352000	0.000000000
F	-1.561760000	-0.617792000	0.000000000
H	0.230631000	-1.534995000	0.000000000
H	1.495233000	0.596736000	0.000000000
H	-0.122632000	1.522252000	0.000000000

Compound 8

C	-0.313186000	-0.574764000	0.000000000
C	0.313186000	0.574764000	0.000000000
F	-1.656358000	-0.609131000	0.000000000
F	1.656358000	0.609131000	0.000000000
H	0.162948000	-1.539434000	0.000000000
H	-0.162948000	1.539434000	0.000000000

Compound 9

C	-0.236800000	-0.462357000	0.000000000
C	0.416814000	0.669781000	0.000000000
F	-1.551177000	-0.583769000	0.000000000
F	0.315298000	-1.661328000	0.000000000
H	1.490381000	0.653848000	0.000000000
H	-0.133815000	1.591525000	0.000000000

Compound 10

N	0.588975000	1.020104000	0.000000000
C	0.022534000	0.039029000	0.000000000
F	-0.611509000	-1.059134000	0.000000000

Compound 11

F	0.418756000	-0.190444000	0.000000000
H	-0.418756000	0.190444000	0.000000000

Compound 12

C	-0.553705000	0.060037000	0.000000000
O	0.599612000	0.183786000	0.000000000
F	-1.430704000	1.033571000	0.000000000
F	-1.204203000	-1.077393000	0.000000000

Compound 13

B	-0.450957000	0.132187000	-0.205912000
F	-0.670448000	1.372386000	0.174313000
F	0.645178000	-0.487955000	0.174295000
F	-1.327774000	-0.487621000	-0.965696000

Compound 14

Si	-0.966185000	0.235609000	0.039611000
H	-1.679895000	-0.268805000	1.212633000
F	-1.722689000	-0.299436000	-1.271777000
H	0.407413000	-0.267676000	0.009907000
H	-0.982645000	1.698308000	0.010627000

Compound 15

F	-0.571455000	1.485455000	0.002905000
P	-0.730103000	-0.052587000	0.277999000
F	0.770266000	-0.450884000	0.036316000
F	-1.272708000	-0.452985000	-1.141221000

Compound 16

C	0.123863000	0.170945000	-0.000179000
C	0.705209000	1.210358000	-0.000293000
C	-0.624340000	-1.096097000	-0.000113000
F	0.245384000	-2.172516000	-0.000350000
H	1.234634000	2.130706000	-0.000431000
H	-1.238078000	-1.162657000	0.891495000
H	-1.238373000	-1.162540000	-0.891529000

Compound 17

C	0.293866000	-0.508976000	0.000000000
C	-0.293866000	0.508976000	0.000000000
F	-0.937410000	1.623595000	0.000000000
F	0.937410000	-1.623595000	0.000000000

Compound 18

F	-1.500565000	-0.102092000	-0.612888000
C	-0.098320000	-0.099022000	-0.606182000
C	0.367565000	1.344351000	-0.640361000
C	0.366306000	-0.849801000	0.627509000
H	0.205213000	-0.621318000	-1.509756000
H	1.455231000	1.375526000	-0.645391000
H	-0.006442000	1.838458000	-1.532542000
H	-0.000695000	1.864535000	0.241759000
H	1.453942000	-0.870664000	0.652548000
H	-0.008571000	-1.869196000	0.609894000
H	-0.001962000	-0.344981000	1.518509000

Compound 19

C	0.020922000	0.190092000	-0.346640000
C	1.293552000	1.027970000	-0.316817000
C	-1.214044000	1.046108000	-0.515822000
F	0.107589000	-0.633545000	-1.483194000

C	-0.099386000	-0.736295000	0.858762000
H	2.168405000	0.376777000	-0.245489000
H	1.271721000	1.685020000	0.540350000
H	1.365751000	1.627103000	-1.220755000
H	-2.099567000	0.405145000	-0.589715000
H	-1.324829000	1.707547000	0.344864000
H	-1.124821000	1.642484000	-1.415631000
H	0.766631000	-1.383452000	0.909950000
H	-0.163612000	-0.147116000	1.769453000
H	-0.996508000	-1.345036000	0.771187000

Compound 20

C	0.981439000	0.111741000	-0.125994000
C	0.190058000	-0.707919000	0.620546000
C	-1.166098000	-0.366455000	0.423899000
C	-1.155262000	0.680234000	-0.461618000
N	0.133457000	0.958178000	-0.783983000
F	0.632557000	-1.689545000	1.420307000
H	2.046196000	0.151772000	-0.232051000
H	-2.020038000	-0.831528000	0.874956000
H	-1.963596000	1.242979000	-0.882485000
H	0.420885000	1.680742000	-1.414278000

Equilibrium Cartesian coordinates of compounds 1-20 optimized at the CAM-B3LYP/pc-3 level of theory in the gas phase

Compound 1

C	-0.003266000	-0.035959000	0.051066000
F	1.144782000	0.641074000	0.030386000
F	-1.008681000	0.838988000	0.030378000
F	-0.071844000	-0.782269000	-1.051140000
H	-0.060792000	-0.661834000	0.939210000

Compound 2

C	-0.033141000	-0.057347000	0.000021000
F	0.658434000	1.139794000	-0.000272000
H	0.683415000	-0.875962000	-0.000192000
H	-0.654146000	-0.103245000	0.892123000
H	-0.654561000	-0.103242000	-0.891780000

Compound 3

C	-1.070602000	0.235974000	-0.009489000
F	-1.688643000	-0.218248000	1.065895000
F	-1.684860000	-0.257501000	-1.094301000
F	0.177325000	-0.254035000	-0.022452000
O	-1.089612000	1.575854000	0.019106000
H	-0.644610000	1.926955000	-0.758758000

Compound 4

C	0.548391000	-0.315975000	-0.113465000
C	-0.742968000	0.439809000	-0.098764000
F	1.602331000	0.549284000	-0.091857000
F	0.654240000	-1.091955000	1.003039000
H	0.679360000	-0.967931000	-0.977332000
H	-1.577599000	-0.256594000	-0.095018000
H	-0.810484000	1.071359000	-0.980912000
H	-0.788173000	1.060704000	0.792811000

Compound 5

C	-0.770328000	0.012059000	-0.030606000
C	0.733252000	-0.007613000	-0.048636000
F	1.221447000	-0.077012000	1.253314000
H	-1.137456000	0.875766000	0.519427000
H	-1.151854000	0.065543000	-1.049761000
H	1.120770000	-0.874995000	-0.582368000
H	-1.161570000	-0.889760000	0.435282000
H	1.145639000	0.895913000	-0.496651000

Compound 6

C	-0.233197000	-0.644794000	0.000000000
C	0.424113000	0.493625000	0.000000000
F	-1.564947000	-0.700331000	0.000000000
F	-0.193587000	1.674739000	0.000000000
H	0.257316000	-1.604745000	0.000000000
H	1.500699000	0.548807000	0.000000000

Compound 7

C	-0.221124000	-0.555145000	0.000000000
C	0.415621000	0.590729000	0.000000000
F	-1.560265000	-0.624739000	0.000000000
H	0.232034000	-1.535750000	0.000000000
H	1.493139000	0.595569000	0.000000000
H	-0.115105000	1.530136000	0.000000000

Compound 8

C	-0.314749000	-0.576080000	0.000000000
C	0.314749000	0.576080000	0.000000000
F	-1.652798000	-0.612303000	0.000000000
F	1.652798000	0.612303000	0.000000000
H	0.159076000	-1.545314000	0.000000000
H	-0.159076000	1.545314000	0.000000000

Compound 9

C	-0.239223000	-0.466550000	0.000000000
C	0.415194000	0.666983000	0.000000000
F	-1.549464000	-0.587639000	0.000000000
F	0.311097000	-1.661775000	0.000000000
H	1.490660000	0.661218000	0.000000000
H	-0.127563000	1.595463000	0.000000000

Compound 10

N	0.591144000	1.023860000	0.000000000
C	0.019444000	0.033677000	0.000000000
F	-0.610588000	-1.057538000	0.000000000

Compound 11

F	0.419142000	-0.190620000	0.000000000
H	-0.419142000	0.190620000	0.000000000

Compound 12

C	-0.553321000	0.060078000	0.000000000
O	0.605625000	0.184431000	0.000000000
F	-1.433648000	1.030850000	0.000000000
F	-1.207657000	-1.075358000	0.000000000

Compound 13

B	-0.451002000	0.132219000	-0.205767000
F	-0.669254000	1.365871000	0.172288000
F	0.639363000	-0.484686000	0.172218000
F	-1.323108000	-0.484407000	-0.961739000

Compound 14

Si	-0.968647000	0.233823000	0.035152000
H	-1.684853000	-0.272586000	1.218484000
F	-1.720528000	-0.297679000	-1.268990000
H	0.414475000	-0.272108000	0.008020000
H	-0.984447000	1.706549000	0.008333000

Compound 15

F	-0.572991000	1.486441000	0.004579000
P	-0.727356000	-0.050901000	0.273658000
F	0.770297000	-0.452589000	0.037958000
F	-1.273950000	-0.453952000	-1.140197000

Compound 16

C	0.119926000	0.167593000	-0.000246000
C	0.705526000	1.205232000	-0.000314000
C	-0.614347000	-1.086326000	-0.000132000
F	0.249492000	-2.169262000	-0.000331000
H	1.232842000	2.126644000	-0.000363000
H	-1.242392000	-1.162864000	0.886831000
H	-1.242748000	-1.162817000	-0.886846000

Compound 17

C	0.294269000	-0.509674000	0.000000000
C	-0.294269000	0.509674000	0.000000000
F	-0.934287000	1.618186000	0.000000000
F	0.934287000	-1.618186000	0.000000000

Compound 18

F	-1.489265000	-0.113584000	-0.632767000
C	-0.087998000	-0.096128000	-0.601164000
C	0.361541000	1.342467000	-0.640221000
C	0.360281000	-0.848734000	0.625945000
H	0.233376000	-0.618130000	-1.504209000
H	1.448563000	1.397066000	-0.672699000
H	-0.034330000	1.842366000	-1.521225000
H	0.015014000	1.872462000	0.245630000
H	1.447240000	-0.905062000	0.657580000
H	-0.036449000	-1.861322000	0.618913000
H	0.013729000	-0.345604000	1.527317000

Compound 19

C	0.017932000	0.194588000	-0.339116000
C	1.284105000	1.024388000	-0.313990000
C	-1.222886000	1.044602000	-0.511914000
F	0.101590000	-0.628534000	-1.483932000
C	-0.083805000	-0.735275000	0.851384000
H	2.158203000	0.379100000	-0.257287000
H	1.288162000	1.688463000	0.548777000
H	1.357849000	1.629077000	-1.215431000
H	-2.104813000	0.412935000	-0.594394000
H	-1.352026000	1.709188000	0.340703000
H	-1.144376000	1.649635000	-1.412691000
H	0.792419000	-1.377859000	0.905537000
H	-0.151851000	-0.164975000	1.776274000
H	-0.968703000	-1.362530000	0.766581000

Compound 20

C	0.983771000	0.110359000	-0.124985000
C	0.189034000	-0.707247000	0.620048000
C	-1.162013000	-0.366387000	0.423564000

C	-1.156163000	0.679019000	-0.460530000
N	0.134561000	0.959499000	-0.785174000
F	0.625939000	-1.686717000	1.418366000
H	2.050598000	0.151944000	-0.232495000
H	-2.019644000	-0.830737000	0.874260000
H	-1.972080000	1.239040000	-0.878580000
H	0.425596000	1.681425000	-1.415175000

Equilibrium Cartesian coordinates of compounds 1-20 optimized at the mPW3PBE/pc-3 level of theory in the gas phase

Compound 1

C	-0.003164000	-0.034841000	0.049563000
F	1.146601000	0.641618000	0.031031000
F	-1.010369000	0.839851000	0.031018000
F	-0.071997000	-0.783904000	-1.052134000
H	-0.060871000	-0.662724000	0.940421000

Compound 2

C	-0.031711000	-0.054906000	-0.000021000
F	0.658832000	1.140540000	-0.000209000
H	0.684565000	-0.878073000	-0.000182000
H	-0.655595000	-0.103744000	0.893885000
H	-0.656089000	-0.103819000	-0.893572000

Compound 3

C	-1.070344000	0.236663000	-0.009948000
F	-1.689256000	-0.217591000	1.066963000
F	-1.685531000	-0.257353000	-1.096573000
F	0.179622000	-0.253868000	-0.023024000
O	-1.090968000	1.578548000	0.021435000
H	-0.644524000	1.922601000	-0.758853000

Compound 4

C	0.551049000	-0.315761000	-0.110843000
C	-0.743339000	0.440529000	-0.098006000
F	1.605789000	0.548689000	-0.092768000
F	0.656393000	-1.094810000	1.003636000
H	0.678372000	-0.968745000	-0.979408000
H	-1.579469000	-0.257937000	-0.095509000
H	-0.810991000	1.072373000	-0.982975000
H	-0.792706000	1.064364000	0.794374000

Compound 5

C	-0.770609000	0.012054000	-0.028926000

C	0.734283000	-0.008261000	-0.045253000
F	1.227583000	-0.073136000	1.252933000
H	-1.140970000	0.879590000	0.517926000
H	-1.150627000	0.063123000	-1.051299000
H	1.120687000	-0.878675000	-0.581596000
H	-1.165905000	-0.890074000	0.438768000
H	1.145458000	0.895280000	-0.502552000

Compound 6

C	-0.239020000	-0.645011000	0.000000000
C	0.421367000	0.498812000	0.000000000
F	-1.568807000	-0.711089000	0.000000000
F	-0.186254000	1.683534000	0.000000000
H	0.261870000	-1.602735000	0.000000000
H	1.501243000	0.543791000	0.000000000

Compound 7

C	-0.224850000	-0.556584000	0.000000000
C	0.416352000	0.593618000	0.000000000
F	-1.562259000	-0.629457000	0.000000000
H	0.232335000	-1.538518000	0.000000000
H	1.496266000	0.595571000	0.000000000
H	-0.113544000	1.536170000	0.000000000

Compound 8

C	-0.317726000	-0.578254000	0.000000000
C	0.317726000	0.578254000	0.000000000
F	-1.654148000	-0.614530000	0.000000000
F	1.654148000	0.614530000	0.000000000
H	0.158315000	-1.549366000	0.000000000
H	-0.158315000	1.549366000	0.000000000

Compound 9

C	-0.241108000	-0.469798000	0.000000000
C	0.416427000	0.669121000	0.000000000
F	-1.551356000	-0.588935000	0.000000000
F	0.310932000	-1.664057000	0.000000000
H	1.494056000	0.662376000	0.000000000
H	-0.128250000	1.598992000	0.000000000

Compound 10

N	0.593323000	1.027635000	0.000000000
C	0.018194000	0.031512000	0.000000000
F	-0.611517000	-1.059148000	0.000000000

Compound 11

F	0.418488000	-0.190322000	0.000000000
H	-0.418488000	0.190322000	0.000000000

Compound 12

C	-0.553728000	0.060035000	0.000000000
O	0.608822000	0.184774000	0.000000000
F	-1.435143000	1.031632000	0.000000000
F	-1.208951000	-1.076440000	0.000000000

Compound 13

B	-0.451001000	0.132246000	-0.205756000
F	-0.669606000	1.367868000	0.172901000
F	0.641114000	-0.485695000	0.172818000
F	-1.324508000	-0.485421000	-0.962962000

Compound 14

Si	-0.969481000	0.233215000	0.033670000
H	-1.687972000	-0.274723000	1.223847000
F	-1.722771000	-0.299297000	-1.272904000
H	0.420655000	-0.274260000	0.007995000
H	-0.984431000	1.713065000	0.008392000

Compound 15

F	-0.573817000	1.490501000	0.006018000
P	-0.726936000	-0.050624000	0.272892000
F	0.773787000	-0.454754000	0.039506000
F	-1.277034000	-0.456123000	-1.142416000

Compound 16

C	0.121051000	0.163783000	-0.000249000
C	0.706930000	1.209084000	-0.000315000
C	-0.610887000	-1.089037000	-0.000133000
F	0.246606000	-2.175895000	-0.000329000
H	1.232819000	2.133490000	-0.000361000
H	-1.243930000	-1.161636000	0.888263000
H	-1.244289000	-1.161589000	-0.888277000

Compound 17

C	0.295849000	-0.512411000	0.000000000
C	-0.295849000	0.512411000	0.000000000
F	-0.935074000	1.619549000	0.000000000
F	0.935074000	-1.619549000	0.000000000

Compound 18

F	-1.492761000	-0.116131000	-0.637176000
C	-0.092819000	-0.095820000	-0.600635000
C	0.360768000	1.343426000	-0.640305000
C	0.359508000	-0.849284000	0.626733000
H	0.235168000	-0.618670000	-1.505142000
H	1.450398000	1.395709000	-0.673093000
H	-0.033485000	1.846005000	-1.523635000
H	0.016372000	1.877161000	0.247141000
H	1.449076000	-0.904731000	0.656205000
H	-0.035610000	-1.865227000	0.620863000
H	0.015086000	-0.346641000	1.532143000

Compound 19

C	0.018191000	0.191276000	-0.343635000
C	1.284654000	1.024344000	-0.314386000
C	-1.223512000	1.044491000	-0.512909000
F	0.101952000	-0.631310000	-1.487719000
C	-0.083710000	-0.736333000	0.851151000
H	2.162520000	0.380205000	-0.255291000
H	1.285688000	1.689034000	0.550890000
H	1.360727000	1.632639000	-1.216188000
H	-2.109500000	0.414000000	-0.593726000
H	-1.350460000	1.710080000	0.342280000
H	-1.146991000	1.652756000	-1.414686000
H	0.793772000	-1.381221000	0.907507000
H	-0.150201000	-0.162610000	1.776799000
H	-0.971331000	-1.364551000	0.770412000

Compound 20

C	0.986111000	0.111365000	-0.125993000
C	0.188313000	-0.710909000	0.623192000
C	-1.163320000	-0.368878000	0.425759000
C	-1.157176000	0.680631000	-0.461824000
N	0.134995000	0.959267000	-0.785008000
F	0.626380000	-1.688351000	1.419715000
H	2.054878000	0.155473000	-0.235767000
H	-2.023905000	-0.833071000	0.876523000
H	-1.973644000	1.242893000	-0.881731000
H	0.426968000	1.681779000	-1.415567000

Equilibrium Cartesian coordinates of compounds 1-20 optimized at the SVWN/pc-3 level of theory in the gas phase

Compound 1

C	-0.003063000	-0.033752000	0.047822000
F	1.142277000	0.641531000	0.027663000
F	-1.006133000	0.838979000	0.027655000
F	-0.071510000	-0.778627000	-1.051549000

H	-0.061371000	-0.668131000	0.948309000

Compound 2

C	-0.028012000	-0.048468000	0.000000000
F	0.657533000	1.138223000	-0.000233000
H	0.689169000	-0.882527000	-0.000200000
H	-0.659120000	-0.103587000	0.899269000
H	-0.659571000	-0.103642000	-0.898936000

Compound 3

C	-1.071119000	0.238231000	-0.008584000
F	-1.687945000	-0.217094000	1.064686000
F	-1.683249000	-0.256018000	-1.094228000
F	0.176443000	-0.252592000	-0.023796000
O	-1.093022000	1.572430000	0.025073000
H	-0.642109000	1.924043000	-0.763152000

Compound 4

C	0.544968000	-0.311879000	-0.110289000
C	-0.736480000	0.435800000	-0.099156000
F	1.592880000	0.552031000	-0.089120000
F	0.647737000	-1.084104000	1.002372000
H	0.679704000	-0.973305000	-0.985090000
H	-1.579959000	-0.265112000	-0.094424000
H	-0.806167000	1.074398000	-0.988027000
H	-0.777584000	1.060872000	0.802233000

Compound 5

C	-0.761663000	0.009399000	-0.030147000
C	0.728253000	-0.005819000	-0.040196000
F	1.209384000	-0.069373000	1.253731000
H	-1.139238000	0.881231000	0.519600000
H	-1.145970000	0.059071000	-1.058431000
H	1.124195000	-0.879971000	-0.582849000
H	-1.157806000	-0.899952000	0.440228000
H	1.142744000	0.905314000	-0.501935000

Compound 6

C	-0.240799000	-0.644290000	0.000000000
C	0.419871000	0.499997000	0.000000000
F	-1.563758000	-0.696258000	0.000000000
F	-0.196592000	1.671737000	0.000000000
H	0.262398000	-1.611686000	0.000000000
H	1.509278000	0.547801000	0.000000000

Compound 7

C	-0.227515000	-0.555378000	0.000000000
C	0.414169000	0.592289000	0.000000000
F	-1.557089000	-0.624220000	0.000000000
H	0.234393000	-1.545963000	0.000000000
H	1.501781000	0.593092000	0.000000000
H	-0.121440000	1.540981000	0.000000000

Compound 8

C	-0.320052000	-0.577166000	0.000000000
C	0.320052000	0.577166000	0.000000000
F	-1.648328000	-0.608401000	0.000000000
F	1.648328000	0.608401000	0.000000000
H	0.164292000	-1.555490000	0.000000000
H	-0.164292000	1.555490000	0.000000000

Compound 9

C	-0.241664000	-0.470773000	0.000000000
C	0.415156000	0.666917000	0.000000000
F	-1.546968000	-0.590132000	0.000000000
F	0.307693000	-1.660858000	0.000000000
H	1.500752000	0.659300000	0.000000000
H	-0.134268000	1.603246000	0.000000000

Compound 10

N	0.594414000	1.029525000	0.000000000
C	0.016525000	0.028621000	0.000000000
F	-0.610939000	-1.058147000	0.000000000

Compound 11

F	0.423533000	-0.192617000	0.000000000
H	-0.423533000	0.192617000	0.000000000

Compound 12

C	-0.553823000	0.060025000	0.000000000
O	0.611035000	0.185011000	0.000000000
F	-1.435867000	1.028403000	0.000000000
F	-1.210345000	-1.073438000	0.000000000

Compound 13

B	-0.450999000	0.132238000	-0.205760000
F	-0.668941000	1.364077000	0.171736000
F	0.637783000	-0.483800000	0.171668000
F	-1.321844000	-0.483517000	-0.960644000

Compound 14

Si	-0.970291000	0.232668000	0.032309000
H	-1.693387000	-0.278651000	1.227760000
F	-1.720983000	-0.297945000	-1.269822000
H	0.426767000	-0.278203000	0.005255000
H	-0.986106000	1.720131000	0.005499000

Compound 15

F	-0.572359000	1.486040000	0.003588000
P	-0.728767000	-0.051841000	0.276079000
F	0.770159000	-0.451879000	0.036951000
F	-1.273033000	-0.453321000	-1.140620000

Compound 16

C	0.114233000	0.156245000	-0.000241000
C	0.704941000	1.203344000	-0.000311000
C	-0.603946000	-1.086578000	-0.000134000
F	0.251713000	-2.167084000	-0.000331000
H	1.237272000	2.134001000	-0.000367000
H	-1.247777000	-1.160888000	0.893210000
H	-1.248136000	-1.160840000	-0.893224000

Compound 17

C	0.297490000	-0.515252000	0.000000000
C	-0.297490000	0.515252000	0.000000000
F	-0.933692000	1.617155000	0.000000000
F	0.933692000	-1.617155000	0.000000000

Compound 18

F	-1.482530000	-0.113574000	-0.632741000
C	-0.089996000	-0.096921000	-0.602537000
C	0.358958000	1.327508000	-0.635047000
C	0.357714000	-0.836779000	0.615567000
H	0.239632000	-0.624850000	-1.515833000
H	1.455485000	1.384458000	-0.668166000
H	-0.041597000	1.843644000	-1.516660000
H	0.012414000	1.850953000	0.267631000
H	1.454176000	-0.894845000	0.648922000
H	-0.043714000	-1.857999000	0.622297000
H	0.011159000	-0.315799000	1.519666000

Compound 19

C	0.018006000	0.192752000	-0.341517000
C	1.270726000	1.017273000	-0.311932000
C	-1.210334000	1.037331000	-0.508603000
F	0.101452000	-0.625517000	-1.479964000

C	-0.082799000	-0.724447000	0.841131000
H	2.153225000	0.366395000	-0.253031000
H	1.272303000	1.685360000	0.560015000
H	1.344274000	1.630607000	-1.219910000
H	-2.101623000	0.400822000	-0.588755000
H	-1.337760000	1.707847000	0.352093000
H	-1.130719000	1.649054000	-1.417152000
H	0.802503000	-1.371857000	0.897032000
H	-0.150304000	-0.146527000	1.772828000
H	-0.977150000	-1.356293000	0.758266000

Compound 20

C	0.983169000	0.112197000	-0.126506000
C	0.185233000	-0.710689000	0.623222000
C	-1.159250000	-0.369627000	0.426109000
C	-1.153780000	0.679109000	-0.460767000
N	0.134380000	0.954639000	-0.781050000
F	0.622204000	-1.681758000	1.414442000
H	2.060293000	0.154722000	-0.235515000
H	-2.025501000	-0.838153000	0.880916000
H	-1.976314000	1.245508000	-0.883758000
H	0.429166000	1.684249000	-1.417795000

Equilibrium Cartesian coordinates of compounds 1-20 optimized at the B97-2/pc-3 level of theory in the gas phase

Compound 1

C	-0.003155000	-0.034752000	0.049410000
F	1.145942000	0.640821000	0.031722000
F	-1.009866000	0.838949000	0.031709000
F	-0.072008000	-0.784018000	-1.050886000
H	-0.060713000	-0.661000000	0.937944000

Compound 2

C	-0.031956000	-0.055249000	0.000038000
F	0.658053000	1.138995000	-0.000271000
H	0.683057000	-0.876259000	-0.000222000
H	-0.654384000	-0.103731000	0.891989000
H	-0.654768000	-0.103757000	-0.891635000

Compound 3

C	-1.070068000	0.236816000	-0.010437000
F	-1.688536000	-0.216442000	1.065710000
F	-1.685039000	-0.256658000	-1.095807000
F	0.178709000	-0.253163000	-0.023073000
O	-1.090399000	1.578306000	0.020431000
H	-0.645668000	1.920141000	-0.756823000

Compound 4

C	0.551756000	-0.315763000	-0.110233000
C	-0.743987000	0.441132000	-0.097662000
F	1.606616000	0.547115000	-0.093880000
F	0.657450000	-1.095985000	1.002258000
H	0.677659000	-0.966786000	-0.977089000
H	-1.577898000	-0.256205000	-0.095760000
H	-0.811243000	1.070949000	-0.981120000
H	-0.795255000	1.064244000	0.791987000

Compound 5

C	-0.771399000	0.011743000	-0.027465000
C	0.734456000	-0.008021000	-0.046134000
F	1.231952000	-0.072138000	1.249520000
H	-1.141122000	0.877597000	0.517986000
H	-1.150856000	0.062185000	-1.047745000
H	1.119092000	-0.876615000	-0.582008000
H	-1.165445000	-0.888467000	0.439690000
H	1.143223000	0.893616000	-0.503844000

Compound 6

C	-0.239527000	-0.644659000	0.000000000
C	0.420816000	0.499061000	0.000000000
F	-1.568249000	-0.714606000	0.000000000
F	-0.182918000	1.684798000	0.000000000
H	0.261897000	-1.599418000	0.000000000
H	1.498379000	0.542125000	0.000000000

Compound 7

C	-0.225207000	-0.556437000	0.000000000
C	0.415539000	0.593860000	0.000000000
F	-1.561599000	-0.631583000	0.000000000
H	0.232518000	-1.535496000	0.000000000
H	1.493205000	0.594422000	0.000000000
H	-0.110155000	1.536033000	0.000000000

Compound 8

C	-0.317587000	-0.578324000	0.000000000
C	0.317587000	0.578324000	0.000000000
F	-1.653242000	-0.615429000	0.000000000
F	1.653242000	0.615429000	0.000000000
H	0.156734000	-1.547501000	0.000000000
H	-0.156734000	1.547501000	0.000000000

Compound 9

C	-0.241010000	-0.469628000	0.000000000

C	0.416466000	0.669190000	0.000000000
F	-1.550514000	-0.588517000	0.000000000
F	0.310872000	-1.663119000	0.000000000
H	1.491698000	0.662675000	0.000000000
H	-0.126811000	1.597099000	0.000000000

Compound 10

N	0.592869000	1.026849000	0.000000000
C	0.018141000	0.031421000	0.000000000
F	-0.611011000	-1.058271000	0.000000000

Compound 11

F	0.416667000	-0.189494000	0.000000000
H	-0.416667000	0.189494000	0.000000000

Compound 12

C	-0.553991000	0.060007000	0.000000000
O	0.607213000	0.184601000	0.000000000
F	-1.434106000	1.030800000	0.000000000
F	-1.208115000	-1.075407000	0.000000000

Compound 13

B	-0.450995000	0.132244000	-0.205768000
F	-0.669655000	1.368138000	0.172987000
F	0.641369000	-0.485828000	0.172916000
F	-1.324720000	-0.485555000	-0.963135000

Compound 14

Si	-0.969747000	0.233025000	0.033252000
H	-1.686495000	-0.273655000	1.222587000
F	-1.722647000	-0.299217000	-1.272619000
H	0.418868000	-0.273139000	0.008679000
H	-0.983979000	1.710986000	0.009101000

Compound 15

F	-0.574035000	1.489994000	0.006463000
P	-0.726050000	-0.050036000	0.271321000
F	0.773229000	-0.454770000	0.039941000
F	-1.277144000	-0.456189000	-1.141726000

Compound 16

C	0.122142000	0.164345000	-0.000244000
C	0.706990000	1.209679000	-0.000313000
C	-0.611247000	-1.089568000	-0.000132000

F	0.244394000	-2.176207000	-0.000330000
H	1.231245000	2.132532000	-0.000365000
H	-1.242434000	-1.161316000	0.886552000
H	-1.242789000	-1.161266000	-0.886567000

Compound 17

C	0.295557000	-0.511904000	0.000000000
C	-0.295557000	0.511904000	0.000000000
F	-0.934266000	1.618149000	0.000000000
F	0.934266000	-1.618149000	0.000000000

Compound 18

F	-1.492789000	-0.116356000	-0.637566000
C	-0.093732000	-0.095096000	-0.599384000
C	0.360779000	1.345178000	-0.640908000
C	0.359517000	-0.850682000	0.627951000
H	0.234183000	-0.616697000	-1.501729000
H	1.448261000	1.396110000	-0.673243000
H	-0.031271000	1.844842000	-1.524083000
H	0.017250000	1.881063000	0.242663000
H	1.446939000	-0.905059000	0.656477000
H	-0.033395000	-1.865037000	0.619634000
H	0.015959000	-0.352470000	1.533288000

Compound 19

C	0.018298000	0.190959000	-0.344091000
C	1.285986000	1.024802000	-0.314941000
C	-1.224683000	1.044987000	-0.513499000
F	0.101986000	-0.631428000	-1.487519000
C	-0.083807000	-0.737329000	0.852173000
H	2.162642000	0.382663000	-0.257689000
H	1.288089000	1.686943000	0.549488000
H	1.361836000	1.633309000	-1.213885000
H	-2.109488000	0.416776000	-0.594675000
H	-1.351757000	1.708958000	0.340099000
H	-1.148802000	1.652620000	-1.413007000
H	0.791795000	-1.380776000	0.910390000
H	-0.151300000	-0.164541000	1.775752000
H	-0.968995000	-1.365141000	0.771903000

Compound 20

C	0.985547000	0.111509000	-0.126077000
C	0.188418000	-0.710452000	0.622798000
C	-1.163230000	-0.368500000	0.425433000
C	-1.156580000	0.680464000	-0.461723000
N	0.134734000	0.958874000	-0.784658000
F	0.626304000	-1.687467000	1.418974000
H	2.051903000	0.156327000	-0.236288000
H	-2.022267000	-0.831390000	0.874990000

H	-1.970916000	1.241923000	-0.881096000
H	0.425685000	1.678910000	-1.413055000

Equilibrium Cartesian coordinates of compounds 1-20 optimized at the BHandHLYP/pc-3 level of theory in the gas phase

Compound 1

C	-0.003187000	-0.035055000	0.049675000
F	1.134637000	0.635305000	0.030365000
F	-0.999762000	0.831478000	0.030372000
F	-0.071218000	-0.775589000	-1.041804000
H	-0.060271000	-0.656140000	0.931291000

Compound 2

C	-0.032068000	-0.055525000	-0.000008000
F	0.653288000	1.130955000	-0.000226000
H	0.678691000	-0.870149000	-0.000180000
H	-0.649719000	-0.102617000	0.886038000
H	-0.650191000	-0.102664000	-0.885723000

Compound 3

C	-1.069732000	0.235249000	-0.011013000
F	-1.682620000	-0.212716000	1.055406000
F	-1.679151000	-0.251883000	-1.084531000
F	0.165999000	-0.248412000	-0.022521000
O	-1.087438000	1.567387000	0.015289000
H	-0.648059000	1.919375000	-0.752631000

Compound 4

C	0.548753000	-0.314704000	-0.111246000
C	-0.740459000	0.438409000	-0.098689000
F	1.593256000	0.542673000	-0.091486000
F	0.653025000	-1.084960000	0.994333000
H	0.674234000	-0.960370000	-0.970438000
H	-1.569841000	-0.254405000	-0.095164000
H	-0.807121000	1.065938000	-0.975982000
H	-0.786749000	1.056119000	0.787173000

Compound 5

C	-0.768017000	0.011707000	-0.030201000
C	0.732657000	-0.007532000	-0.046042000
F	1.216246000	-0.072824000	1.243992000
H	-1.134690000	0.871399000	0.514496000
H	-1.145783000	0.062185000	-1.044254000
H	1.116333000	-0.869784000	-0.576810000
H	-1.156978000	-0.884117000	0.435102000

H	1.140131000	0.888867000	-0.496283000

Compound 6

C	-0.234028000	-0.640946000	0.000000000
C	0.420362000	0.492424000	0.000000000
F	-1.554753000	-0.700477000	0.000000000
F	-0.188375000	1.665994000	0.000000000
H	0.257561000	-1.591816000	0.000000000
H	1.489633000	0.542122000	0.000000000

Compound 7

C	-0.222800000	-0.553167000	0.000000000
C	0.413293000	0.588018000	0.000000000
F	-1.550373000	-0.623769000	0.000000000
H	0.231590000	-1.524859000	0.000000000
H	1.484070000	0.591514000	0.000000000
H	-0.111480000	1.523063000	0.000000000

Compound 8

C	-0.314200000	-0.573086000	0.000000000
C	0.314200000	0.573086000	0.000000000
F	-1.641799000	-0.609544000	0.000000000
F	1.641799000	0.609544000	0.000000000
H	0.156998000	-1.534854000	0.000000000
H	-0.156998000	1.534854000	0.000000000

Compound 9

C	-0.238458000	-0.465222000	0.000000000
C	0.413323000	0.663743000	0.000000000
F	-1.537296000	-0.584792000	0.000000000
F	0.307481000	-1.649812000	0.000000000
H	1.481896000	0.657678000	0.000000000
H	-0.126245000	1.586106000	0.000000000

Compound 10

N	0.586369000	1.015591000	0.000000000
C	0.019244000	0.033330000	0.000000000
F	-0.605613000	-1.048922000	0.000000000

Compound 11

F	0.414014000	-0.188288000	0.000000000
H	-0.414014000	0.188288000	0.000000000

Compound 12

C	-0.556798000	0.059705000	0.000000000
O	0.594051000	0.183189000	0.000000000
F	-1.425112000	1.022238000	0.000000000
F	-1.201142000	-1.065131000	0.000000000

Compound 13

B	-0.450889000	0.132331000	-0.205965000
F	-0.667709000	1.356915000	0.169623000
F	0.631424000	-0.480258000	0.169543000
F	-1.316827000	-0.479990000	-0.956201000

Compound 14

Si	-0.969484000	0.233227000	0.033704000
H	-1.681906000	-0.270438000	1.212231000
F	-1.715433000	-0.294101000	-1.260173000
H	0.407582000	-0.269981000	0.007427000
H	-0.984759000	1.699294000	0.007811000

Compound 15

F	-0.571835000	1.473348000	0.002610000
P	-0.724576000	-0.049094000	0.268795000
F	0.758465000	-0.446945000	0.035686000
F	-1.266054000	-0.448309000	-1.131092000

Compound 16

C	0.122880000	0.165493000	-0.000220000
C	0.702454000	1.199919000	-0.000302000
C	-0.611249000	-1.086758000	-0.000134000
F	0.242411000	-2.160712000	-0.000328000
H	1.223835000	2.116647000	-0.000387000
H	-1.235842000	-1.158230000	0.880865000
H	-1.236189000	-1.158160000	-0.880893000

Compound 17

C	0.292206000	-0.506100000	0.000000000
C	-0.292206000	0.506100000	0.000000000
F	-0.927381000	1.606224000	0.000000000
F	0.927381000	-1.606224000	0.000000000

Compound 18

F	-1.479631000	-0.112292000	-0.630521000
C	-0.091061000	-0.095382000	-0.599875000
C	0.361210000	1.339370000	-0.639311000

C	0.359952000	-0.846397000	0.623717000
H	0.230732000	-0.613544000	-1.496276000
H	1.442215000	1.391559000	-0.669578000
H	-0.030350000	1.836269000	-1.516239000
H	0.015742000	1.867627000	0.240252000
H	1.440898000	-0.899599000	0.654368000
H	-0.032458000	-1.853962000	0.616120000
H	0.014452000	-0.347852000	1.520442000

Compound 19

C	0.018118000	0.192643000	-0.341829000
C	1.280712000	1.022465000	-0.313631000
C	-1.219637000	1.042608000	-0.511095000
F	0.100952000	-0.622342000	-1.475322000
C	-0.083557000	-0.732575000	0.848566000
H	2.150802000	0.382119000	-0.256759000
H	1.282695000	1.681923000	0.544831000
H	1.355234000	1.624616000	-1.209288000
H	-2.097621000	0.415880000	-0.592559000
H	-1.345993000	1.702759000	0.337436000
H	-1.142879000	1.644908000	-1.406432000
H	0.787372000	-1.371878000	0.903818000
H	-0.151074000	-0.163665000	1.767021000
H	-0.963326000	-1.356660000	0.765745000

Compound 20

C	0.978973000	0.110128000	-0.124473000
C	0.189746000	-0.704863000	0.617993000
C	-1.158575000	-0.364138000	0.421422000
C	-1.151664000	0.677086000	-0.459200000
N	0.132979000	0.956140000	-0.782223000
F	0.621762000	-1.675949000	1.409564000
H	2.039018000	0.151763000	-0.231572000
H	-2.011309000	-0.825002000	0.868835000
H	-1.963116000	1.232909000	-0.874002000
H	0.421786000	1.672124000	-1.407044000

Equilibrium Cartesian coordinates of compounds 1-20 optimized at the HSE06/pc-3 level of theory in the gas phase

Compound 1

C	-0.003157000	-0.034770000	0.049458000
F	1.144546000	0.640674000	0.030664000
F	-1.008519000	0.838549000	0.030649000
F	-0.071848000	-0.782262000	-1.050549000
H	-0.060822000	-0.662192000	0.939679000

Compound 2

C	-0.031338000	-0.054262000	0.000015000
F	0.657984000	1.139051000	-0.000265000
H	0.683993000	-0.877355000	-0.000192000
H	-0.655108000	-0.103714000	0.893155000
H	-0.655530000	-0.103721000	-0.892813000

Compound 3

C	-1.070286000	0.236809000	-0.010053000
F	-1.688028000	-0.216767000	1.064828000
F	-1.684345000	-0.255911000	-1.094616000
F	0.177330000	-0.252427000	-0.023075000
O	-1.090659000	1.576732000	0.020893000
H	-0.645013000	1.920565000	-0.757978000

Compound 4

C	0.550211000	-0.315286000	-0.110856000
C	-0.742295000	0.439797000	-0.098200000
F	1.602790000	0.548123000	-0.092169000
F	0.655146000	-1.092342000	1.002211000
H	0.677676000	-0.967902000	-0.978748000
H	-1.578082000	-0.258267000	-0.095341000
H	-0.809838000	1.071639000	-0.982538000
H	-0.790509000	1.062940000	0.794141000

Compound 5

C	-0.769478000	0.011162000	-0.029298000
C	0.733672000	-0.007329000	-0.044791000
F	1.224090000	-0.073122000	1.251947000
H	-1.140633000	0.877103000	0.518540000
H	-1.149267000	0.062923000	-1.051117000
H	1.121047000	-0.876006000	-0.582022000
H	-1.163195000	-0.891593000	0.437374000
H	1.143665000	0.896762000	-0.500632000

Compound 6

C	-0.238746000	-0.644386000	0.000000000
C	0.420972000	0.498254000	0.000000000
F	-1.566740000	-0.708852000	0.000000000
F	-0.187156000	1.680626000	0.000000000
H	0.261842000	-1.601588000	0.000000000
H	1.500226000	0.543246000	0.000000000

Compound 7

C	-0.225130000	-0.556086000	0.000000000

C	0.415286000	0.592965000	0.000000000
F	-1.560485000	-0.628874000	0.000000000
H	0.233137000	-1.536934000	0.000000000
H	1.494747000	0.593967000	0.000000000
H	-0.113254000	1.535762000	0.000000000

Compound 8

C	-0.317515000	-0.577560000	0.000000000
C	0.317515000	0.577560000	0.000000000
F	-1.652215000	-0.613350000	0.000000000
F	1.652215000	0.613350000	0.000000000
H	0.158728000	-1.547940000	0.000000000
H	-0.158728000	1.547940000	0.000000000

Compound 9

C	-0.240709000	-0.469106000	0.000000000
C	0.416064000	0.668492000	0.000000000
F	-1.549136000	-0.588568000	0.000000000
F	0.310139000	-1.661951000	0.000000000
H	1.493187000	0.661188000	0.000000000
H	-0.128845000	1.597646000	0.000000000

Compound 10

N	0.592433000	1.026093000	0.000000000
C	0.018202000	0.031526000	0.000000000
F	-0.610635000	-1.057620000	0.000000000

Compound 11

F	0.417786000	-0.190003000	0.000000000
H	-0.417786000	0.190003000	0.000000000

Compound 12

C	-0.554236000	0.059980000	0.000000000
O	0.606657000	0.184542000	0.000000000
F	-1.433600000	1.029856000	0.000000000
F	-1.207821000	-1.074377000	0.000000000

Compound 13

B	-0.451091000	0.132156000	-0.205603000
F	-0.669323000	1.366493000	0.172421000
F	0.639998000	-0.484958000	0.172393000
F	-1.323585000	-0.484692000	-0.962212000

Compound 14

Si	-0.969876000	0.232965000	0.033033000
H	-1.687761000	-0.274688000	1.223454000
F	-1.721965000	-0.298666000	-1.271600000
H	0.420175000	-0.274266000	0.007930000
H	-0.984573000	1.712654000	0.008183000

Compound 15

F	-0.573406000	1.488113000	0.005264000
P	-0.727028000	-0.050678000	0.273075000
F	0.771707000	-0.453551000	0.038685000
F	-1.275273000	-0.454885000	-1.141025000

Compound 16

C	0.121135000	0.163125000	-0.000204000
C	0.706687000	1.207574000	-0.000297000
C	-0.610051000	-1.088969000	-0.000128000
F	0.245754000	-2.173743000	-0.000342000
H	1.231495000	2.132182000	-0.000402000
H	-1.243194000	-1.161020000	0.887679000
H	-1.243526000	-1.160949000	-0.887706000

Compound 17

C	0.295458000	-0.511733000	0.000000000
C	-0.295458000	0.511733000	0.000000000
F	-0.933909000	1.617530000	0.000000000
F	0.933909000	-1.617530000	0.000000000

Compound 18

F	-1.490388000	-0.115342000	-0.635810000
C	-0.092961000	-0.096095000	-0.601111000
C	0.360719000	1.341110000	-0.639553000
C	0.359461000	-0.847477000	0.625102000
H	0.234495000	-0.618731000	-1.505249000
H	1.449879000	1.392969000	-0.671279000
H	-0.033066000	1.844782000	-1.521824000
H	0.015736000	1.872837000	0.248367000
H	1.448560000	-0.901790000	0.654738000
H	-0.035189000	-1.863048000	0.620708000
H	0.014454000	-0.343419000	1.529009000

Compound 19

C	0.018174000	0.191108000	-0.343950000
C	1.282676000	1.023047000	-0.314245000
C	-1.221569000	1.043464000	-0.512332000
F	0.101695000	-0.630074000	-1.485822000

C	-0.083544000	-0.734338000	0.849771000
H	2.159819000	0.378508000	-0.257349000
H	1.284184000	1.685383000	0.552272000
H	1.357349000	1.632926000	-1.214559000
H	-2.107398000	0.413469000	-0.592828000
H	-1.347313000	1.708594000	0.342816000
H	-1.144758000	1.651407000	-1.413773000
H	0.794628000	-1.377417000	0.908257000
H	-0.152384000	-0.159632000	1.774158000
H	-0.969758000	-1.363644000	0.768085000

Compound 20

C	0.984638000	0.111487000	-0.125996000
C	0.188235000	-0.710164000	0.622567000
C	-1.162361000	-0.368427000	0.425313000
C	-1.155711000	0.680134000	-0.461503000
N	0.134569000	0.958301000	-0.784163000
F	0.625544000	-1.686266000	1.418010000
H	2.052967000	0.155424000	-0.235595000
H	-2.022523000	-0.832453000	0.875907000
H	-1.971839000	1.242154000	-0.881229000
H	0.426080000	1.680010000	-1.414013000

Equilibrium Cartesian coordinates of compounds 1-20 optimized at the ωB97XD /pc-3 level of theory in the gas phase

Compound 1

C	-0.003215000	-0.034774000	0.049459000
F	1.144493000	0.640384000	0.030882000
F	-1.008551000	0.838347000	0.030854000
F	-0.071816000	-0.782318000	-1.050286000
H	-0.060710000	-0.661639000	0.938991000

Compound 2

C	-0.031455000	-0.054580000	0.000023000
F	0.657525000	1.138971000	0.000132000
H	0.683249000	-0.877129000	-0.000482000
H	-0.654371000	-0.103227000	0.892461000
H	-0.654947000	-0.104037000	-0.892235000

Compound 3

C	-1.070244000	0.235622000	-0.010155000
F	-1.687809000	-0.216644000	1.064466000
F	-1.684457000	-0.256662000	-1.093974000
F	0.176830000	-0.253137000	-0.022670000
O	-1.089773000	1.576936000	0.019310000
H	-0.645547000	1.922885000	-0.756976000

Compound 4

C	0.552091000	-0.316039000	-0.110358000
C	-0.744370000	0.441437000	-0.097538000
F	1.604828000	0.546639000	-0.092858000
F	0.657085000	-1.093998000	1.001636000
H	0.676979000	-0.967004000	-0.978005000
H	-1.577500000	-0.257847000	-0.095629000
H	-0.809872000	1.070992000	-0.982114000
H	-0.794142000	1.064521000	0.793366000

Compound 5

C	-0.771935000	0.011939000	-0.030347000
C	0.735368000	-0.008130000	-0.043362000
F	1.224036000	-0.073385000	1.253831000
H	-1.141289000	0.877862000	0.516344000
H	-1.145234000	0.064504000	-1.053244000
H	1.120061000	-0.877424000	-0.578798000
H	-1.165549000	-0.890416000	0.434385000
H	1.144441000	0.894950000	-0.498809000

Compound 6

C	-0.238444000	-0.643912000	0.000000000
C	0.420363000	0.497919000	0.000000000
F	-1.566196000	-0.710108000	0.000000000
F	-0.185698000	1.680962000	0.000000000
H	0.261589000	-1.600268000	0.000000000
H	1.498785000	0.542708000	0.000000000

Compound 7

C	-0.225316000	-0.555553000	0.000000000
C	0.414507000	0.592467000	0.000000000
F	-1.560035000	-0.629153000	0.000000000
H	0.234199000	-1.534711000	0.000000000
H	1.493023000	0.592506000	0.000000000
H	-0.112078000	1.535244000	0.000000000

Compound 8

C	-0.317187000	-0.577083000	0.000000000
C	0.317187000	0.577083000	0.000000000
F	-1.651980000	-0.613779000	0.000000000
F	1.651980000	0.613779000	0.000000000
H	0.158043000	-1.546884000	0.000000000
H	-0.158043000	1.546884000	0.000000000

Compound 9

C	-0.240676000	-0.469068000	0.000000000
C	0.415734000	0.667910000	0.000000000
F	-1.548151000	-0.587855000	0.000000000
F	0.310248000	-1.660750000	0.000000000
H	1.491931000	0.661012000	0.000000000
H	-0.128384000	1.596451000	0.000000000

Compound 10

N	0.591629000	1.024700000	0.000000000
C	0.018442000	0.031941000	0.000000000
F	-0.610071000	-1.056642000	0.000000000

Compound 11

F	0.416891000	-0.189596000	0.000000000
H	-0.416891000	0.189596000	0.000000000

Compound 12

C	-0.555171000	0.059880000	0.000000000
O	0.605758000	0.184445000	0.000000000
F	-1.432681000	1.029936000	0.000000000
F	-1.206906000	-1.074260000	0.000000000

Compound 13

B	-0.451529000	0.131877000	-0.204889000
F	-0.669397000	1.367769000	0.172540000
F	0.641104000	-0.485467000	0.172464000
F	-1.324179000	-0.485181000	-0.963115000

Compound 14

Si	-0.970069000	0.232791000	0.032548000
H	-1.684316000	-0.272434000	1.220899000
F	-1.722163000	-0.298607000	-1.271798000
H	0.416354000	-0.272086000	0.009711000
H	-0.983806000	1.708337000	0.009640000

Compound 15

F	-0.573896000	1.488430000	0.005744000
P	-0.726248000	-0.050196000	0.271754000
F	0.771919000	-0.454099000	0.039258000
F	-1.275775000	-0.455136000	-1.140757000

Compound 16

C	0.121667000	0.166440000	-0.000214000
C	0.706808000	1.207677000	-0.000302000
C	-0.612946000	-1.091448000	-0.000129000
F	0.245894000	-2.171995000	-0.000338000
H	1.232216000	2.131147000	-0.000393000
H	-1.242501000	-1.161845000	0.888027000
H	-1.242838000	-1.161776000	-0.888052000

Compound 17

C	0.294977000	-0.510901000	0.000000000
C	-0.294977000	0.510901000	0.000000000
F	-0.933492000	1.616808000	0.000000000
F	0.933492000	-1.616808000	0.000000000

Compound 18

F	-1.491484000	-0.117941000	-0.640308000
C	-0.094588000	-0.097117000	-0.602881000
C	0.360516000	1.343559000	-0.639312000
C	0.359256000	-0.848490000	0.627345000
H	0.235159000	-0.618453000	-1.504767000
H	1.448750000	1.391550000	-0.672454000
H	-0.032521000	1.847451000	-1.520310000
H	0.017554000	1.873372000	0.249200000
H	1.447432000	-0.902097000	0.652922000
H	-0.034644000	-1.863070000	0.623776000
H	0.016271000	-0.342967000	1.529889000

Compound 19

C	0.018842000	0.189533000	-0.346104000
C	1.285940000	1.023942000	-0.315982000
C	-1.223672000	1.044429000	-0.512698000
F	0.101912000	-0.631762000	-1.486649000
C	-0.083872000	-0.735906000	0.852095000
H	2.161965000	0.379701000	-0.258700000
H	1.285075000	1.684780000	0.550510000
H	1.359155000	1.633213000	-1.215536000
H	-2.108277000	0.414871000	-0.596856000
H	-1.348738000	1.704099000	0.345620000
H	-1.146668000	1.656179000	-1.410321000
H	0.794064000	-1.377247000	0.912961000
H	-0.154966000	-0.158197000	1.773339000
H	-0.968960000	-1.364832000	0.768821000

Compound 20

C	0.984575000	0.110433000	-0.125100000
C	0.189404000	-0.709561000	0.621978000
C	-1.162880000	-0.367606000	0.424654000

C	-1.156036000	0.679091000	-0.460599000
N	0.134630000	0.958828000	-0.784613000
F	0.625513000	-1.685845000	1.417656000
H	2.051562000	0.155126000	-0.235249000
H	-2.021897000	-0.831232000	0.874832000
H	-1.970696000	1.241003000	-0.880333000
H	0.425424000	1.679963000	-1.413928000

Equilibrium Cartesian coordinates of compounds 1-20 optimized at the PBE0/pc-3 level of theory in the gas phase

Compound 1

C	-0.003135000	-0.034534000	0.049142000
F	1.143891000	0.640390000	0.030481000
F	-1.007926000	0.838148000	0.030467000
F	-0.071794000	-0.781670000	-1.050087000
H	-0.060835000	-0.662333000	0.939896000

Compound 2

C	-0.031074000	-0.053773000	0.000019000
F	0.657598000	1.138309000	-0.000248000
H	0.684355000	-0.877413000	-0.000215000
H	-0.655231000	-0.103537000	0.893450000
H	-0.655647000	-0.103587000	-0.893107000

Compound 3

C	-1.070267000	0.236621000	-0.010089000
F	-1.687632000	-0.216692000	1.064140000
F	-1.683972000	-0.255347000	-1.094028000
F	0.176633000	-0.251859000	-0.023110000
O	-1.090833000	1.576728000	0.021185000
H	-0.644931000	1.919549000	-0.758098000

Compound 4

C	0.550591000	-0.315271000	-0.110505000
C	-0.742249000	0.439849000	-0.098082000
F	1.602179000	0.547478000	-0.091923000
F	0.655290000	-1.091681000	1.001586000
H	0.677738000	-0.968072000	-0.978948000
H	-1.578180000	-0.258611000	-0.095317000
H	-0.809665000	1.071764000	-0.982826000
H	-0.790606000	1.063246000	0.794515000

Compound 5

C	-0.769394000	0.011491000	-0.030225000
C	0.734020000	-0.007916000	-0.043025000

F	1.222161000	-0.072428000	1.252742000
H	-1.141178000	0.878261000	0.516686000
H	-1.147382000	0.062966000	-1.053044000
H	1.121442000	-0.877336000	-0.579882000
H	-1.164515000	-0.891030000	0.436418000
H	1.144744000	0.895892000	-0.499670000

Compound 6

C	-0.239429000	-0.644237000	0.000000000
C	0.420502000	0.498771000	0.000000000
F	-1.566372000	-0.709159000	0.000000000
F	-0.186705000	1.680448000	0.000000000
H	0.262129000	-1.601461000	0.000000000
H	1.500273000	0.542939000	0.000000000

Compound 7

C	-0.225623000	-0.556114000	0.000000000
C	0.415142000	0.593161000	0.000000000
F	-1.559861000	-0.628930000	0.000000000
H	0.233158000	-1.537274000	0.000000000
H	1.495024000	0.593608000	0.000000000
H	-0.113542000	1.536348000	0.000000000

Compound 8

C	-0.317793000	-0.577661000	0.000000000
C	0.317793000	0.577661000	0.000000000
F	-1.651486000	-0.613251000	0.000000000
F	1.651486000	0.613251000	0.000000000
H	0.158685000	-1.548472000	0.000000000
H	-0.158685000	1.548472000	0.000000000

Compound 9

C	-0.241101000	-0.469802000	0.000000000
C	0.415878000	0.668169000	0.000000000
F	-1.548349000	-0.588729000	0.000000000
F	0.309597000	-1.661353000	0.000000000
H	1.493407000	0.661454000	0.000000000
H	-0.128731000	1.597962000	0.000000000

Compound 10

N	0.592510000	1.026226000	0.000000000
C	0.018049000	0.031260000	0.000000000
F	-0.610558000	-1.057487000	0.000000000

Compound 11

F	0.417786000	-0.190003000	0.000000000
H	-0.417786000	0.190003000	0.000000000

Compound 12

C	-0.554934000	0.059905000	0.000000000
O	0.606599000	0.184535000	0.000000000
F	-1.433181000	1.029509000	0.000000000
F	-1.207484000	-1.073949000	0.000000000

Compound 13

B	-0.451002000	0.132220000	-0.205760000
F	-0.669281000	1.366045000	0.172341000
F	0.639531000	-0.484770000	0.172278000
F	-1.323249000	-0.484497000	-0.961859000

Compound 14

Si	-0.970057000	0.232814000	0.032717000
H	-1.688136000	-0.274928000	1.223859000
F	-1.721892000	-0.298577000	-1.271454000
H	0.420723000	-0.274496000	0.007819000
H	-0.984638000	1.713187000	0.008059000

Compound 15

F	-0.573338000	1.487388000	0.005165000
P	-0.726869000	-0.050578000	0.272773000
F	0.771059000	-0.453233000	0.038574000
F	-1.274852000	-0.454578000	-1.140513000

Compound 16

C	0.121714000	0.162721000	-0.000205000
C	0.706902000	1.207753000	-0.000298000
C	-0.609733000	-1.090156000	-0.000128000
F	0.244884000	-2.173576000	-0.000341000
H	1.231321000	2.133121000	-0.000401000
H	-1.243228000	-1.160867000	0.888009000
H	-1.243560000	-1.160795000	-0.888036000

Compound 17

C	0.295570000	-0.511928000	0.000000000
C	-0.295570000	0.511928000	0.000000000
F	-0.933752000	1.617258000	0.000000000
F	0.933752000	-1.617258000	0.000000000

Compound 18

F	-1.489376000	-0.115351000	-0.635824000
C	-0.093792000	-0.096036000	-0.601011000
C	0.360591000	1.341251000	-0.639556000
C	0.359334000	-0.847549000	0.625223000
H	0.234737000	-0.618738000	-1.505261000
H	1.450068000	1.392714000	-0.671387000
H	-0.033255000	1.844895000	-1.522250000
H	0.015653000	1.873405000	0.248527000
H	1.448750000	-0.901756000	0.654463000
H	-0.035378000	-1.863473000	0.620594000
H	0.014370000	-0.343564000	1.529580000

Compound 19

C	0.018278000	0.190427000	-0.344997000
C	1.282684000	1.023257000	-0.313994000
C	-1.221723000	1.043189000	-0.512433000
F	0.101881000	-0.629164000	-1.484977000
C	-0.083557000	-0.734663000	0.849493000
H	2.160803000	0.379542000	-0.254550000
H	1.282185000	1.687143000	0.551706000
H	1.358871000	1.631912000	-1.215492000
H	-2.107818000	0.412853000	-0.592854000
H	-1.347172000	1.708075000	0.343274000
H	-1.145345000	1.651545000	-1.414066000
H	0.793588000	-1.379911000	0.905792000
H	-0.149062000	-0.159425000	1.774108000
H	-0.971814000	-1.361981000	0.769491000

Compound 20

C	0.984632000	0.111428000	-0.125946000
C	0.188358000	-0.710454000	0.622804000
C	-1.162416000	-0.368612000	0.425473000
C	-1.155544000	0.680005000	-0.461405000
N	0.134572000	0.958125000	-0.784014000
F	0.625422000	-1.685740000	1.417573000
H	2.053351000	0.155607000	-0.235776000
H	-2.023069000	-0.832630000	0.876094000
H	-1.971818000	1.242395000	-0.881434000
H	0.426113000	1.680076000	-1.414071000

Equilibrium Cartesian coordinates of compounds 1-20 optimized at the M062X/pc-3 level of theory in the gas phase

Compound 1

C	-0.003254000	-0.036167000	0.051358000
F	1.142144000	0.640330000	0.029763000
F	-1.006142000	0.837606000	0.029550000
F	-0.071821000	-0.780164000	-1.049490000

H	-0.060727000	-0.661604000	0.938719000

Compound 2

C	-0.031791000	-0.055235000	-0.000119000
F	0.656730000	1.137256000	0.000318000
H	0.682919000	-0.875702000	-0.000451000
H	-0.653531000	-0.102643000	0.891588000
H	-0.654325000	-0.103677000	-0.891435000

Compound 3

C	-1.070781000	0.235143000	-0.009154000
F	-1.687334000	-0.219471000	1.063585000
F	-1.683427000	-0.254032000	-1.092014000
F	0.174647000	-0.250564000	-0.022592000
O	-1.090211000	1.576876000	0.020095000
H	-0.643896000	1.921048000	-0.759920000

Compound 4

C	0.549955000	-0.316855000	-0.113428000
C	-0.744501000	0.440210000	-0.099490000
F	1.598904000	0.548304000	-0.090211000
F	0.653451000	-1.088370000	1.001643000
H	0.679569000	-0.968246000	-0.977622000
H	-1.577723000	-0.257615000	-0.094506000
H	-0.809673000	1.071953000	-0.981475000
H	-0.784884000	1.059320000	0.793589000

Compound 5

C	-0.778767000	0.024309000	-0.042121000
C	0.744400000	0.023306000	-0.040570000
F	1.220404000	-0.626803000	1.089383000
H	-1.183646000	1.032929000	-0.024831000
H	-1.183660000	-0.491811000	-0.908858000
H	1.154036000	-0.501385000	-0.902230000
H	-1.127040000	-0.492529000	0.849284000
H	1.154173000	1.031883000	-0.020058000

Compound 6

C	-0.234134000	-0.645486000	0.000000000
C	0.424218000	0.494806000	0.000000000
F	-1.564393000	-0.698673000	0.000000000
F	-0.194771000	1.673478000	0.000000000
H	0.258805000	-1.603860000	0.000000000
H	1.500674000	0.547036000	0.000000000

Compound 7

C	-0.222580000	-0.556104000	0.000000000
C	0.416546000	0.591881000	0.000000000
F	-1.559449000	-0.623889000	0.000000000
H	0.232510000	-1.535827000	0.000000000
H	1.493972000	0.594769000	0.000000000
H	-0.116699000	1.529970000	0.000000000

Compound 8

C	-0.316149000	-0.576710000	0.000000000
C	0.316149000	0.576710000	0.000000000
F	-1.652009000	-0.610497000	0.000000000
F	1.652009000	0.610497000	0.000000000
H	0.160981000	-1.544271000	0.000000000
H	-0.160981000	1.544271000	0.000000000

Compound 9

C	-0.239408000	-0.466826000	0.000000000
C	0.416258000	0.668831000	0.000000000
F	-1.548169000	-0.587999000	0.000000000
F	0.310163000	-1.660823000	0.000000000
H	1.491561000	0.659243000	0.000000000
H	-0.129704000	1.595274000	0.000000000

Compound 10

N	0.591504000	1.024484000	0.000000000
C	0.019444000	0.033677000	0.000000000
F	-0.610948000	-1.058162000	0.000000000

Compound 11

F	0.417786000	-0.190003000	0.000000000
H	-0.417786000	0.190003000	0.000000000

Compound 12

C	-0.554543000	0.059947000	0.000000000
O	0.604484000	0.184308000	0.000000000
F	-1.432278000	1.029222000	0.000000000
F	-1.206663000	-1.073477000	0.000000000

Compound 13

B	-0.451041000	0.131973000	-0.205679000
F	-0.669093000	1.365023000	0.171964000
F	0.638996000	-0.484154000	0.172153000
F	-1.322863000	-0.483844000	-0.961437000

Compound 14

Si	-0.969176000	0.233332000	0.033951000
H	-1.683015000	-0.270929000	1.216851000
F	-1.719749000	-0.297647000	-1.268031000
H	0.412011000	-0.270931000	0.008591000
H	-0.984071000	1.704174000	0.009639000

Compound 15

F	-0.571092000	1.481364000	0.002458000
P	-0.729001000	-0.051943000	0.276022000
F	0.766357000	-0.449094000	0.035710000
F	-1.270264000	-0.451327000	-1.138191000

Compound 16

C	0.122030000	0.167393000	-0.000251000
C	0.707091000	1.208961000	-0.000317000
C	-0.615126000	-1.092429000	-0.000129000
F	0.246337000	-2.168809000	-0.000334000
H	1.234445000	2.131128000	-0.000358000
H	-1.243062000	-1.164041000	0.887507000
H	-1.243414000	-1.164002000	-0.887518000

Compound 17

C	0.294855000	-0.510690000	0.000000000
C	-0.294855000	0.510690000	0.000000000
F	-0.934988000	1.619399000	0.000000000
F	0.934988000	-1.619399000	0.000000000

Compound 18

F	-1.486283000	-0.113769000	-0.633084000
C	-0.091698000	-0.099189000	-0.606464000
C	0.362437000	1.341270000	-0.639468000
C	0.361179000	-0.847484000	0.625285000
H	0.232312000	-0.620976000	-1.509136000
H	1.449687000	1.393217000	-0.670006000
H	-0.034582000	1.846570000	-1.517062000
H	0.014128000	1.861879000	0.251655000
H	1.448369000	-0.900807000	0.655591000
H	-0.036702000	-1.859815000	0.624634000
H	0.012853000	-0.335099000	1.521155000

Compound 19

C	0.018582000	0.190295000	-0.345076000
C	1.284503000	1.023894000	-0.315066000
C	-1.222832000	1.044370000	-0.511737000

F	0.101347000	-0.628721000	-1.482507000
C	-0.083743000	-0.734592000	0.851843000
H	2.158245000	0.377301000	-0.257839000
H	1.282042000	1.684695000	0.550541000
H	1.356280000	1.630070000	-1.216280000
H	-2.104864000	0.412253000	-0.596603000
H	-1.346958000	1.702878000	0.346740000
H	-1.142354000	1.654062000	-1.409874000
H	0.794480000	-1.375114000	0.908482000
H	-0.154045000	-0.156369000	1.772024000
H	-0.968883000	-1.362222000	0.765854000

Compound 20

C	0.985276000	0.110669000	-0.125347000
C	0.189330000	-0.709511000	0.621941000
C	-1.164226000	-0.367887000	0.424983000
C	-1.157272000	0.679918000	-0.461215000
N	0.134569000	0.959462000	-0.785144000
F	0.626693000	-1.686919000	1.418484000
H	2.051930000	0.153568000	-0.233957000
H	-2.021541000	-0.832504000	0.875884000
H	-1.971033000	1.241673000	-0.880877000
H	0.425874000	1.681731000	-1.415453000

Table S1. Values of equilibrium bond lengths (in Å) of compounds **1-20** calculated at the CCSD and DFT level of theory with various exchange-correlation functionals.

Mol.	CCSD	DFT exchange-correlation functionals											
		BP86	PBE	PW91	BHandH	TPSSh	B3LYP	X3LYP	O3LYP	tHCTHhyb	B97-1	M06HF	CAM-B3LYP
**1**	1.327	1.352	1.350	1.349	1.308	1.342	1.340	1.338	1.339	1.338	1.337	1.334	1.333
**2**	1.375	1.399	1.396	1.395	1.353	1.391	1.389	1.388	1.385	1.386	1.385	1.389	1.383
**3**	1.325	1.355	1.354	1.353	1.309	1.344	1.342	1.340	1.341	1.340	1.339	1.334	1.334
**4**	1.356	1.384	1.382	1.381	1.336	1.374	1.372	1.370	1.369	1.369	1.367	1.366	1.364
**5**	1.387	1.411	1.409	1.408	1.361	1.403	1.400	1.399	1.397	1.398	1.397	1.396	1.392
**6**	1.330	1.348	1.346	1.345	1.308	1.340	1.339	1.337	1.335	1.335	1.335	1.338	1.333
**7**	1.338	1.357	1.354	1.354	1.314	1.349	1.347	1.345	1.343	1.343	1.343	1.347	1.341
**8**	1.335	1.354	1.351	1.351	1.313	1.347	1.345	1.343	1.340	1.341	1.340	1.344	1.339
**9**	1.309	1.333	1.331	1.330	1.291	1.324	1.322	1.320	1.320	1.319	1.319	1.320	1.316
**10**	1.260	1.274	1.273	1.272	1.239	1.266	1.264	1.263	1.263	1.263	1.263	1.268	1.260
**11**	0.913	0.931	0.930	0.929	0.912	0.924	0.922	0.921	0.919	0.920	0.919	0.920	0.921
**12**	1.301	1.332	1.331	1.330	1.284	1.320	1.318	1.316	1.317	1.316	1.315	1.310	1.310
**13**	1.304	1.324	1.323	1.322	1.290	1.316	1.313	1.312	1.316	1.315	1.314	1.316	1.309
**14**	1.580	1.616	1.616	1.614	1.571	1.603	1.603	1.602	1.605	1.602	1.603	1.606	1.596
**15**	1.553	1.595	1.595	1.593	1.539	1.578	1.578	1.576	1.579	1.576	1.576	1.571	1.568
**16**	1.376	1.408	1.405	1.405	1.354	1.398	1.395	1.393	1.389	1.390	1.389	1.384	1.385
**17**	1.279	1.292	1.290	1.289	1.259	1.285	1.284	1.283	1.281	1.281	1.282	1.287	1.280
**18**	1.393	1.423	1.420	1.420	1.370	1.414	1.412	1.410	1.405	1.406	1.405	1.402	1.402
**19**	1.402	1.436	1.433	1.433	1.378	1.425	1.423	1.421	1.417	1.418	1.416	1.396	1.412
**20**	1.334	1.351	1.349	1.348	1.310	1.344	1.342	1.341	1.339	1.339	1.339	1.341	1.337

Table S1. Continue

Mol.	DFT exchange-correlation functionals							
	mPW3PBE	SVWN	B97-2	BHandHLYP	HSE06	ωB97XD	PBE0	M062X
**1**	1.334	1.330	1.333	1.321	1.332	1.332	1.331	1.330
**2**	1.381	1.370	1.379	1.370	1.378	1.378	1.377	1.377
**3**	1.336	1.333	1.335	1.321	1.333	1.333	1.333	1.331
**4**	1.364	1.358	1.363	1.351	1.362	1.361	1.360	1.360
**5**	1.390	1.382	1.389	1.379	1.388	1.388	1.386	1.388
**6**	1.331	1.324	1.331	1.322	1.330	1.329	1.329	1.331
**7**	1.339	1.331	1.339	1.329	1.337	1.337	1.336	1.339
**8**	1.337	1.329	1.336	1.328	1.335	1.335	1.334	1.336
**9**	1.316	1.311	1.315	1.304	1.314	1.313	1.313	1.314
**10**	1.259	1.255	1.258	1.250	1.258	1.257	1.257	1.261
**11**	0.919	0.931	0.915	0.910	0.918	0.916	0.918	0.918
**12**	1.312	1.310	1.310	1.296	1.309	1.308	1.308	1.308
**13**	1.311	1.307	1.311	1.299	1.309	1.310	1.309	1.308
**14**	1.599	1.594	1.599	1.584	1.597	1.597	1.596	1.594
**15**	1.572	1.570	1.570	1.553	1.570	1.569	1.569	1.566
**16**	1.384	1.378	1.383	1.372	1.382	1.380	1.380	1.379
**17**	1.278	1.272	1.277	1.270	1.277	1.277	1.276	1.280
**18**	1.401	1.393	1.400	1.389	1.398	1.398	1.396	1.395
**19**	1.412	1.404	1.411	1.399	1.409	1.408	1.407	1.404
**20**	1.335	1.327	1.334	1.325	1.333	1.333	1.332	1.335

Equilibrium Cartesian coordinates of compounds 1-20 optimized at the DFT(M062X) level of theory with different basis sets in gas phase

Geometries optimized with the cc-pVDZ basis set

Compound 1

C	-0.003388000	-0.035092000	0.049742000
F	1.145076000	0.642106000	0.028772000
F	-1.008896000	0.840385000	0.028676000
F	-0.071630000	-0.781136000	-1.053362000
H	-0.060962000	-0.666263000	0.946070000

Compound 2

C	-0.027798000	-0.048207000	-0.000079000
F	0.659832000	1.142692000	-0.000287000
H	0.687319000	-0.883821000	-0.000038000
H	-0.659396000	-0.105412000	0.898676000
H	-0.659958000	-0.105252000	-0.898374000

Compound 3

C	-1.071155000	0.237696000	-0.008613000
F	-1.688803000	-0.221739000	1.066237000
F	-1.684054000	-0.250471000	-1.095655000
F	0.178078000	-0.246881000	-0.023961000
O	-1.093948000	1.580523000	0.026378000
H	-0.641119000	1.909873000	-0.764387000

Compound 4

C	0.553521000	-0.318357000	-0.112594000
C	-0.743821000	0.438583000	-0.101340000
F	1.598907000	0.553254000	-0.088490000
F	0.650982000	-1.087699000	1.006216000

H	0.684567000	-0.976359000	-0.985457000
H	-1.587609000	-0.262944000	-0.092940000
H	-0.811712000	1.080211000	-0.988975000
H	-0.779737000	1.062013000	0.802079000

Compound 5

C	-0.770857000	0.009667000	-0.033783000
C	0.739586000	-0.006048000	-0.041203000
F	1.211303000	-0.068741000	1.258561000
H	-1.141034000	0.881538000	0.523111000
H	-1.158003000	0.059245000	-1.061408000
H	1.129998000	-0.881039000	-0.584438000
H	-1.159987000	-0.899897000	0.444334000
H	1.148894000	0.905175000	-0.505172000

Compound 6

C	-0.238179000	-0.648079000	0.000000000
C	0.424434000	0.499499000	0.000000000
F	-1.571948000	-0.700277000	0.000000000
F	-0.197230000	1.680993000	0.000000000
H	0.261048000	-1.615944000	0.000000000
H	1.512274000	0.551109000	0.000000000

Compound 7

C	-0.228317000	-0.558457000	0.000000000
C	0.420034000	0.594915000	0.000000000
F	-1.566387000	-0.625374000	0.000000000
H	0.234297000	-1.547555000	0.000000000
H	1.508261000	0.597795000	0.000000000
H	-0.123589000	1.539475000	0.000000000

Compound 8

C	-0.321140000	-0.578991000	0.000000000
C	0.321140000	0.578991000	0.000000000
F	-1.659131000	-0.610759000	0.000000000
F	1.659131000	0.610759000	0.000000000
H	0.165938000	-1.554149000	0.000000000
H	-0.165938000	1.554149000	0.000000000

Compound 9

C	-0.241895000	-0.471171000	0.000000000
C	0.418139000	0.672089000	0.000000000
F	-1.554113000	-0.591467000	0.000000000
F	0.310115000	-1.667706000	0.000000000
H	1.503902000	0.659749000	0.000000000
H	-0.135447000	1.606206000	0.000000000

Compound 10

N	0.596178000	1.032580000	0.000000000
C	0.018941000	0.032805000	0.000000000
F	-0.615119000	-1.065386000	0.000000000

Compound 11

F	0.419737000	-0.190890000	0.000000000
H	-0.419737000	0.190890000	0.000000000

Compound 12

C	-0.554352000	0.059968000	0.000000000
O	0.610575000	0.184962000	0.000000000
F	-1.435769000	1.032152000	0.000000000
F	-1.209453000	-1.077081000	0.000000000

Compound 13

B	-0.451063000	0.132285000	-0.205660000
F	-0.671038000	1.376168000	0.175426000
F	0.648325000	-0.489849000	0.175250000
F	-1.330226000	-0.489606000	-0.968014000

Compound 14

Si	-0.963480000	0.237750000	0.043891000
H	-1.688542000	-0.275541000	1.232894000
F	-1.737949000	-0.310119000	-1.299613000
H	0.428419000	-0.275157000	0.011846000
H	-0.982448000	1.721066000	0.011982000

Compound 15

F	-0.572712000	1.514379000	0.003341000
P	-0.740995000	-0.060243000	0.297866000
F	0.797416000	-0.462268000	0.038100000
F	-1.287708000	-0.462868000	-1.163308000

Compound 16

C	0.121392000	0.164213000	-0.000223000
C	0.713095000	1.217435000	-0.000307000
C	-0.614471000	-1.102692000	-0.000128000
F	0.247868000	-2.177367000	-0.000337000
H	1.243366000	2.150416000	-0.000388000
H	-1.251305000	-1.166938000	0.894521000
H	-1.251646000	-1.166867000	-0.894539000

Compound 17

C	0.297899000	-0.515961000	0.000000000
C	-0.297899000	0.515961000	0.000000000
F	-0.941389000	1.630485000	0.000000000
F	0.941389000	-1.630485000	0.000000000

Compound 18

F	-1.486580000	-0.112285000	-0.630516000
C	-0.093158000	-0.099139000	-0.606380000
C	0.363179000	1.343776000	-0.641453000
C	0.361918000	-0.850456000	0.626465000
H	0.240454000	-0.625298000	-1.516608000
H	1.460293000	1.402268000	-0.676405000
H	-0.045500000	1.851178000	-1.525702000
H	0.010518000	1.870346000	0.257661000
H	1.458964000	-0.910883000	0.660239000
H	-0.047630000	-1.869591000	0.624308000
H	0.009243000	-0.334118000	1.531489000

Compound 19

C	0.018261000	0.189713000	-0.345643000
C	1.286373000	1.025948000	-0.313570000
C	-1.225259000	1.046886000	-0.512037000
F	0.101172000	-0.628121000	-1.482767000
C	-0.083971000	-0.737078000	0.854099000
H	2.168111000	0.371969000	-0.259762000
H	1.288215000	1.694014000	0.559537000
H	1.358964000	1.635803000	-1.225071000
H	-2.115425000	0.407654000	-0.595300000
H	-1.351530000	1.718584000	0.349041000
H	-1.144938000	1.653806000	-1.424879000
H	0.802555000	-1.384668000	0.906742000
H	-0.154910000	-0.158651000	1.786264000

H	-0.975817000	-1.373059000	0.763844000

Compound 20

C	0.989066000	0.111280000	-0.126123000
C	0.190250000	-0.715193000	0.626683000
C	-1.167811000	-0.370940000	0.427806000
C	-1.160210000	0.682801000	-0.463452000
N	0.135666000	0.961356000	-0.786818000
F	0.631448000	-1.694571000	1.424633000
H	2.065858000	0.155789000	-0.236780000
H	-2.033725000	-0.839623000	0.882726000
H	-1.981177000	1.250771000	-0.887879000
H	0.430234000	1.688531000	-1.421496000

Geometries optimized with the aug-cc-pVDZ basis set

Compound 1

C	-0.003309000	-0.037159000	0.052813000
F	1.148710000	0.644415000	0.028604000
F	-1.012001000	0.843060000	0.028723000
F	-0.071890000	-0.783568000	-1.056445000
H	-0.061310000	-0.666748000	0.946204000

Compound 2

C	-0.034631000	-0.059530000	-0.000071000
F	0.659775000	1.142130000	0.000177000
H	0.691433000	-0.880674000	-0.000392000
H	-0.657932000	-0.100569000	0.900339000
H	-0.658647000	-0.101355000	-0.900154000

Compound 3

C	-1.070886000	0.237519000	-0.009102000
F	-1.690838000	-0.222294000	1.069790000
F	-1.686623000	-0.256880000	-1.098174000
F	0.181565000	-0.253225000	-0.023015000
O	-1.092043000	1.582439000	0.023003000
H	-0.642175000	1.921441000	-0.762502000

Compound 4

C	0.548765000	-0.317843000	-0.115940000
C	-0.747266000	0.441984000	-0.099225000
F	1.604459000	0.552599000	-0.088992000
F	0.654659000	-1.091600000	1.007880000
H	0.684504000	-0.975515000	-0.984246000
H	-1.585352000	-0.263149000	-0.094104000
H	-0.810673000	1.077897000	-0.988732000
H	-0.783999000	1.064328000	0.801860000

Compound 5

C	-0.774909000	0.010328000	-0.030998000
C	0.735766000	-0.006287000	-0.049791000
F	1.219133000	-0.070786000	1.257640000
H	-1.140472000	0.882226000	0.523254000
H	-1.155246000	0.062044000	-1.058977000
H	1.128270000	-0.882773000	-0.581391000
H	-1.161590000	-0.899876000	0.441465000
H	1.148947000	0.905024000	-0.501201000

Compound 6

C	-0.233693000	-0.650707000	0.000000000
C	0.428968000	0.496900000	0.000000000
F	-1.572173000	-0.694234000	0.000000000

F	-0.202610000	1.678223000	0.000000000
H	0.256401000	-1.620048000	0.000000000
H	1.513506000	0.557168000	0.000000000

Compound 7

C	-0.222532000	-0.560526000	0.000000000
C	0.418625000	0.596965000	0.000000000
F	-1.568463000	-0.625221000	0.000000000
H	0.233052000	-1.549391000	0.000000000
H	1.504690000	0.598063000	0.000000000
H	-0.121071000	1.540910000	0.000000000

Compound 8

C	-0.316028000	-0.581728000	0.000000000
C	0.316028000	0.581728000	0.000000000
F	-1.660449000	-0.613168000	0.000000000
F	1.660449000	0.613168000	0.000000000
H	0.161835000	-1.558191000	0.000000000
H	-0.161835000	1.558191000	0.000000000

Compound 9

C	-0.241124000	-0.469836000	0.000000000
C	0.418769000	0.673179000	0.000000000
F	-1.557787000	-0.593130000	0.000000000
F	0.310511000	-1.671721000	0.000000000
H	1.502551000	0.662698000	0.000000000
H	-0.132219000	1.606509000	0.000000000

Compound 10

N	0.596301000	1.032793000	0.000000000

C	0.019028000	0.032957000	0.000000000
F	-0.615329000	-1.065750000	0.000000000

Compound 11

F	0.419280000	-0.190682000	0.000000000
H	-0.419280000	0.190682000	0.000000000

Compound 12

C	-0.552673000	0.060148000	0.000000000
O	0.613324000	0.185257000	0.000000000
F	-1.438222000	1.034135000	0.000000000
F	-1.211429000	-1.079539000	0.000000000

Compound 13

B	-0.451069000	0.132199000	-0.205693000
F	-0.671533000	1.379164000	0.176362000
F	0.651126000	-0.491278000	0.176282000
F	-1.332525000	-0.491086000	-0.969950000

Compound 14

Si	-0.961096000	0.239259000	0.048443000
H	-1.691385000	-0.277681000	1.230672000
F	-1.735077000	-0.307829000	-1.294540000
H	0.428170000	-0.277290000	0.008311000
H	-0.984612000	1.721541000	0.008114000

Compound 15

F	-0.572204000	1.515795000	0.003558000
P	-0.742285000	-0.060815000	0.299108000

F	0.798080000	-0.462182000	0.037633000
F	-1.287591000	-0.463797000	-1.164300000

Compound 16

C	0.121315000	0.166148000	-0.000248000
C	0.713073000	1.218958000	-0.000317000
C	-0.621556000	-1.096350000	-0.000129000
F	0.249208000	-2.176714000	-0.000334000
H	1.245002000	2.147726000	-0.000363000
H	-1.249192000	-1.170808000	0.896492000
H	-1.249549000	-1.170762000	-0.896503000

Compound 17

C	0.298023000	-0.516177000	0.000000000
C	-0.298023000	0.516177000	0.000000000
F	-0.941602000	1.630855000	0.000000000
F	0.941602000	-1.630855000	0.000000000

Compound 18

F	-1.491687000	-0.113910000	-0.633332000
C	-0.087833000	-0.098023000	-0.604442000
C	0.362675000	1.346546000	-0.640765000
C	0.361413000	-0.851242000	0.629208000
H	0.234672000	-0.623958000	-1.514294000
H	1.458129000	1.398187000	-0.674162000
H	-0.040724000	1.848878000	-1.526819000
H	0.011190000	1.871630000	0.256234000
H	1.456805000	-0.906901000	0.657823000
H	-0.042853000	-1.869414000	0.621759000
H	0.009914000	-0.335997000	1.531889000

Compound 19

C	0.018015000	0.193334000	-0.341317000
C	1.287823000	1.026104000	-0.314338000
C	-1.226615000	1.046638000	-0.513132000
F	0.101654000	-0.630247000	-1.487186000
C	-0.083923000	-0.738439000	0.853647000
H	2.166046000	0.372589000	-0.257340000
H	1.285172000	1.691446000	0.557908000
H	1.358984000	1.634906000	-1.223095000
H	-2.113693000	0.407825000	-0.593672000
H	-1.349063000	1.714644000	0.348394000
H	-1.144750000	1.653493000	-1.422306000
H	0.801184000	-1.382902000	0.906485000
H	-0.153385000	-0.156921000	1.781331000
H	-0.975650000	-1.369669000	0.765124000

Compound 20

C	0.991016000	0.111562000	-0.126494000
C	0.189984000	-0.712980000	0.624830000
C	-1.168774000	-0.370949000	0.427879000
C	-1.160827000	0.683051000	-0.463622000
N	0.136131000	0.961626000	-0.787078000
F	0.631704000	-1.696184000	1.425979000
H	2.065973000	0.155550000	-0.236587000
H	-2.033807000	-0.838549000	0.881823000
H	-1.980751000	1.249523000	-0.886853000
H	0.428950000	1.687549000	-1.420578000

Geometries optimized with the cc-pVTZ basis set

Compound 1

C	-0.003320000	-0.036111000	0.050844000

F	1.142698000	0.640337000	0.030117000
F	-1.006779000	0.837921000	0.029901000
F	-0.071787000	-0.780697000	-1.050022000
H	-0.060611000	-0.661451000	0.939059000

Compound 2

C	-0.031130000	-0.054058000	-0.000047000
F	0.657570000	1.138430000	-0.000268000
H	0.682980000	-0.876632000	-0.000074000
H	-0.654436000	-0.103921000	0.891906000
H	-0.654985000	-0.103818000	-0.891617000

Compound 3

C	-1.071131000	0.235699000	-0.008608000
F	-1.687611000	-0.220516000	1.064139000
F	-1.683084000	-0.253097000	-1.092387000
F	0.174751000	-0.249555000	-0.023091000
O	-1.090780000	1.577551000	0.021030000
H	-0.643146000	1.918920000	-0.761083000

Compound 4

C	0.550003000	-0.316646000	-0.113074000
C	-0.743970000	0.439657000	-0.099859000
F	1.598002000	0.549930000	-0.090247000
F	0.651814000	-1.088014000	1.002452000
H	0.678660000	-0.968290000	-0.978475000
H	-1.577890000	-0.258983000	-0.094331000
H	-0.808814000	1.072361000	-0.982486000
H	-0.782707000	1.058685000	0.794521000

Compound 5

C	-0.778431000	0.024624000	-0.042665000
C	0.745204000	0.023011000	-0.039970000
F	1.218587000	-0.628225000	1.090251000
H	-1.183740000	1.033966000	-0.025629000
H	-1.183727000	-0.492989000	-0.909358000
H	1.153303000	-0.501019000	-0.903544000
H	-1.124675000	-0.492377000	0.850603000
H	1.153380000	1.032909000	-0.019687000

Compound 6

C	-0.234350000	-0.645495000	0.000000000
C	0.424155000	0.495052000	0.000000000
F	-1.565686000	-0.699312000	0.000000000
F	-0.195039000	1.674926000	0.000000000
H	0.259385000	-1.604720000	0.000000000
H	1.501934000	0.546849000	0.000000000

Compound 7

C	-0.223515000	-0.555688000	0.000000000
C	0.417275000	0.591872000	0.000000000
F	-1.560690000	-0.624661000	0.000000000
H	0.232659000	-1.536225000	0.000000000
H	1.495840000	0.595272000	0.000000000
H	-0.117269000	1.530230000	0.000000000

Compound 8

C	-0.316976000	-0.576528000	0.000000000
C	0.316976000	0.576528000	0.000000000
F	-1.653675000	-0.610626000	0.000000000
F	1.653675000	0.610626000	0.000000000

H	0.161856000	-1.544348000	0.000000000
H	-0.161856000	1.544348000	0.000000000

Compound 9

C	-0.239516000	-0.467039000	0.000000000
C	0.416395000	0.669066000	0.000000000
F	-1.548847000	-0.587867000	0.000000000
F	0.310604000	-1.661346000	0.000000000
H	1.492510000	0.658946000	0.000000000
H	-0.130444000	1.595939000	0.000000000

Compound 10

N	0.592178000	1.025651000	0.000000000
C	0.019440000	0.033669000	0.000000000
F	-0.611617000	-1.059321000	0.000000000

Compound 11

F	0.418018000	-0.190109000	0.000000000
H	-0.418018000	0.190109000	0.000000000

Compound 12

C	-0.554252000	0.059979000	0.000000000
O	0.606090000	0.184481000	0.000000000
F	-1.433211000	1.028971000	0.000000000
F	-1.207628000	-1.073429000	0.000000000

Compound 13

B	-0.450523000	0.132612000	-0.206556000
F	-0.669558000	1.366283000	0.172689000

F	0.639750000	-0.485134000	0.172668000
F	-1.323670000	-0.484762000	-0.961800000

Compound 14

Si	-0.967525000	0.234526000	0.037333000
H	-1.684549000	-0.272295000	1.220196000
F	-1.723624000	-0.300041000	-1.274834000
H	0.415531000	-0.271880000	0.008893000
H	-0.983832000	1.707689000	0.009412000

Compound 15

F	-0.572170000	1.489021000	0.002518000
P	-0.731299000	-0.053617000	0.280933000
F	0.773031000	-0.452745000	0.036031000
F	-1.273562000	-0.453659000	-1.143483000

Compound 16

C	0.120901000	0.167159000	-0.000216000
C	0.707855000	1.208792000	-0.000303000
C	-0.614794000	-1.093939000	-0.000128000
F	0.247410000	-2.169065000	-0.000338000
H	1.234506000	2.131706000	-0.000392000
H	-1.243620000	-1.163261000	0.888097000
H	-1.243957000	-1.163191000	-0.888120000

Compound 17

C	0.295154000	-0.511207000	0.000000000
C	-0.295154000	0.511207000	0.000000000
F	-0.936063000	1.621262000	0.000000000
F	0.936063000	-1.621262000	0.000000000

Compound 18

F	-1.485459000	-0.111992000	-0.630008000
C	-0.091407000	-0.099614000	-0.607201000
C	0.363115000	1.340859000	-0.639807000
C	0.361856000	-0.847573000	0.624761000
H	0.232787000	-0.621846000	-1.510642000
H	1.451258000	1.394707000	-0.669849000
H	-0.036051000	1.846193000	-1.517580000
H	0.012555000	1.859878000	0.252549000
H	1.449938000	-0.901413000	0.656965000
H	-0.038168000	-1.860076000	0.624046000
H	0.011277000	-0.333326000	1.519867000

Compound 19

C	0.018436000	0.190332000	-0.344893000
C	1.284045000	1.024390000	-0.313445000
C	-1.222684000	1.044968000	-0.511550000
F	0.101752000	-0.627191000	-1.482194000
C	-0.083947000	-0.735304000	0.851377000
H	2.158198000	0.376728000	-0.256719000
H	1.281440000	1.685940000	0.552826000
H	1.355330000	1.630105000	-1.216131000
H	-2.105791000	0.411923000	-0.590616000
H	-1.343716000	1.709274000	0.344102000
H	-1.143309000	1.648809000	-1.414808000
H	0.795734000	-1.375655000	0.905642000
H	-0.154096000	-0.157887000	1.773190000
H	-0.969591000	-1.363629000	0.763721000

Compound 20

C	0.985668000	0.110627000	-0.125338000
C	0.189169000	-0.709798000	0.622194000
C	-1.164352000	-0.367913000	0.425013000

C	-1.157831000	0.680184000	-0.461402000
N	0.134563000	0.959934000	-0.785543000
F	0.627100000	-1.687797000	1.419198000
H	2.053250000	0.153042000	-0.233601000
H	-2.021984000	-0.833299000	0.876586000
H	-1.972499000	1.242290000	-0.881299000
H	0.426516000	1.682929000	-1.416510000

Geometries optimized with the aug-cc-pVTZ basis set

Compound 1

C	-0.003368000	-0.036698000	0.051746000
F	1.142985000	0.640772000	0.029561000
F	-1.006980000	0.838394000	0.029338000
F	-0.071766000	-0.780387000	-1.050726000
H	-0.060670000	-0.662082000	0.939980000

Compound 2

C	-0.032651000	-0.056353000	-0.000028000
F	0.657681000	1.137926000	-0.000223000
H	0.684360000	-0.875818000	-0.000213000
H	-0.654484000	-0.102836000	0.892706000
H	-0.654907000	-0.102917000	-0.892344000

Compound 3

C	-1.071156000	0.235393000	-0.008564000
F	-1.687937000	-0.221039000	1.064708000
F	-1.683661000	-0.254682000	-1.092516000
F	0.175158000	-0.251132000	-0.022656000
O	-1.090248000	1.577786000	0.020102000
H	-0.643157000	1.922674000	-0.761074000

Compound 4

C	0.549273000	-0.316616000	-0.113662000
C	-0.744900000	0.440364000	-0.099604000
F	1.599863000	0.548834000	-0.090044000
F	0.653777000	-1.088935000	1.002538000
H	0.679561000	-0.968673000	-0.978269000
H	-1.578457000	-0.258682000	-0.094437000
H	-0.809410000	1.072613000	-0.982560000
H	-0.784609000	1.059796000	0.794538000

Compound 5

C	-0.779187000	0.024323000	-0.042154000
C	0.744471000	0.023744000	-0.041240000
F	1.219811000	-0.628025000	1.089843000
H	-1.183505000	1.034071000	-0.025362000
H	-1.183505000	-0.493328000	-0.909285000
H	1.154344000	-0.501514000	-0.903436000
H	-1.126940000	-0.492440000	0.850781000
H	1.154411000	1.033068000	-0.019146000

Compound 6

C	-0.234041000	-0.645877000	0.000000000
C	0.424654000	0.494954000	0.000000000
F	-1.565595000	-0.698453000	0.000000000
F	-0.195712000	1.674380000	0.000000000
H	0.258925000	-1.605253000	0.000000000
H	1.502168000	0.547551000	0.000000000

Compound 7

C	-0.222514000	-0.556171000	0.000000000
C	0.416805000	0.592341000	0.000000000

F	-1.560749000	-0.624391000	0.000000000
H	0.232659000	-1.536982000	0.000000000
H	1.495255000	0.594887000	0.000000000
H	-0.117156000	1.531116000	0.000000000

Compound 8

C	-0.316216000	-0.576975000	0.000000000
C	0.316216000	0.576975000	0.000000000
F	-1.653466000	-0.611063000	0.000000000
F	1.653466000	0.611063000	0.000000000
H	0.161051000	-1.545510000	0.000000000
H	-0.161051000	1.545510000	0.000000000

Compound 9

C	-0.239312000	-0.466685000	0.000000000
C	0.416556000	0.669344000	0.000000000
F	-1.549114000	-0.588484000	0.000000000
F	0.310203000	-1.661885000	0.000000000
H	1.492671000	0.659203000	0.000000000
H	-0.130302000	1.596207000	0.000000000

Compound 10

N	0.592025000	1.025386000	0.000000000
C	0.019518000	0.033805000	0.000000000
F	-0.611543000	-1.059193000	0.000000000

Compound 11

F	0.418616000	-0.190380000	0.000000000
H	-0.418616000	0.190380000	0.000000000

Compound 12

C	-0.554183000	0.059986000	0.000000000
O	0.606194000	0.184492000	0.000000000
F	-1.433342000	1.029377000	0.000000000
F	-1.207670000	-1.073854000	0.000000000

Compound 13

B	-0.451023000	0.132264000	-0.205730000
F	-0.669474000	1.366968000	0.172597000
F	0.640416000	-0.485286000	0.172572000
F	-1.323919000	-0.484946000	-0.962438000

Compound 14

Si	-0.966657000	0.235474000	0.038158000
H	-1.685056000	-0.272818000	1.219549000
F	-1.723908000	-0.300574000	-1.274516000
H	0.415858000	-0.272687000	0.008566000
H	-0.984237000	1.708605000	0.009244000

Compound 15

F	-0.571225000	1.485917000	0.001722000
P	-0.731684000	-0.053706000	0.281222000
F	0.770806000	-0.450837000	0.035207000
F	-1.271897000	-0.452375000	-1.142152000

Compound 16

C	0.121981000	0.167110000	-0.000267000
C	0.707692000	1.209121000	-0.000323000
C	-0.615844000	-1.091988000	-0.000130000

F	0.246783000	-2.170184000	-0.000329000
H	1.234515000	2.132314000	-0.000343000
H	-1.243233000	-1.164101000	0.888524000
H	-1.243594000	-1.164072000	-0.888531000

Compound 17

C	0.295003000	-0.510946000	0.000000000
C	-0.295003000	0.510946000	0.000000000
F	-0.935794000	1.620795000	0.000000000
F	0.935794000	-1.620795000	0.000000000

Compound 18

F	-1.487096000	-0.112975000	-0.631712000
C	-0.090344000	-0.099457000	-0.606928000
C	0.362905000	1.341275000	-0.639597000
C	0.361646000	-0.847599000	0.625225000
H	0.231657000	-0.621912000	-1.510757000
H	1.451115000	1.393503000	-0.669424000
H	-0.035267000	1.846710000	-1.517740000
H	0.012976000	1.860629000	0.252827000
H	1.449796000	-0.900444000	0.656132000
H	-0.037385000	-1.860473000	0.624415000
H	0.011699000	-0.333459000	1.520657000

Compound 19

C	0.018367000	0.191107000	-0.344124000
C	1.284162000	1.024638000	-0.313343000
C	-1.223075000	1.044602000	-0.512229000
F	0.102395000	-0.628249000	-1.484042000
C	-0.083768000	-0.735388000	0.851249000
H	2.158198000	0.376913000	-0.256415000
H	1.280617000	1.685153000	0.553711000

H	1.355653000	1.631326000	-1.215292000
H	-2.105984000	0.411314000	-0.590729000
H	-1.343679000	1.708516000	0.343778000
H	-1.143588000	1.648809000	-1.415173000
H	0.794735000	-1.377331000	0.904701000
H	-0.151239000	-0.156939000	1.772623000
H	-0.970996000	-1.361671000	0.765788000

Compound 20

C	0.985940000	0.110475000	-0.125228000
C	0.189141000	-0.709356000	0.621822000
C	-1.164576000	-0.368068000	0.425160000
C	-1.157955000	0.679982000	-0.461223000
N	0.134752000	0.959815000	-0.785455000
F	0.627180000	-1.688039000	1.419397000
H	2.053461000	0.153439000	-0.233951000
H	-2.022504000	-0.833213000	0.876548000
H	-1.972281000	1.242329000	-0.881347000
H	0.426441000	1.682835000	-1.416425000

Geometries optimized with the cc-pVQZ basis set

Compound 1

C	-0.003282000	-0.036234000	0.051275000
F	1.142507000	0.640166000	0.029836000
F	-1.006613000	0.837679000	0.029815000
F	-0.071674000	-0.780319000	-1.049674000
H	-0.060738000	-0.661292000	0.938647000

Compound 2

C	-0.031890000	-0.055107000	0.000024000

F	0.657405000	1.137561000	-0.000317000
H	0.682876000	-0.875412000	-0.000236000
H	-0.654054000	-0.103535000	0.891408000
H	-0.654337000	-0.103507000	-0.890980000

Compound 3

C	-1.070695000	0.235407000	-0.009384000
F	-1.687178000	-0.219051000	1.063399000
F	-1.683316000	-0.254547000	-1.091996000
F	0.174527000	-0.250994000	-0.022688000
O	-1.090439000	1.576907000	0.020429000
H	-0.643900000	1.921278000	-0.759760000

Compound 4

C	0.549955000	-0.316855000	-0.113428000
C	-0.744501000	0.440210000	-0.099490000
F	1.598904000	0.548304000	-0.090211000
F	0.653451000	-1.088370000	1.001643000
H	0.679569000	-0.968246000	-0.977622000
H	-1.577723000	-0.257615000	-0.094506000
H	-0.809673000	1.071953000	-0.981475000
H	-0.784884000	1.059320000	0.793589000

Compound 5

C	-0.778694000	0.024280000	-0.042078000
C	0.744682000	0.023079000	-0.040087000
F	1.220081000	-0.627697000	1.089295000
H	-1.182943000	1.033046000	-0.025659000
H	-1.182938000	-0.492541000	-0.908556000
H	1.153283000	-0.500481000	-0.902871000
H	-1.126923000	-0.491845000	0.849732000
H	1.153352000	1.032059000	-0.019775000

Compound 6

C	-0.234134000	-0.645486000	0.000000000
C	0.424218000	0.494806000	0.000000000
F	-1.564393000	-0.698673000	0.000000000
F	-0.194771000	1.673478000	0.000000000
H	0.258805000	-1.603860000	0.000000000
H	1.500674000	0.547036000	0.000000000

Compound 7

C	-0.222580000	-0.556104000	0.000000000
C	0.416546000	0.591881000	0.000000000
F	-1.559449000	-0.623889000	0.000000000
H	0.232510000	-1.535827000	0.000000000
H	1.493972000	0.594769000	0.000000000
H	-0.116699000	1.529970000	0.000000000

Compound 8

C	-0.316149000	-0.576710000	0.000000000
C	0.316149000	0.576710000	0.000000000
F	-1.652009000	-0.610497000	0.000000000
F	1.652009000	0.610497000	0.000000000
H	0.160981000	-1.544271000	0.000000000
H	-0.160981000	1.544271000	0.000000000

Compound 9

C	-0.239408000	-0.466826000	0.000000000
C	0.416258000	0.668831000	0.000000000
F	-1.548169000	-0.587999000	0.000000000
F	0.310163000	-1.660823000	0.000000000
H	1.491561000	0.659243000	0.000000000

H	-0.129704000	1.595274000	0.000000000

Compound 10

N	0.591504000	1.024484000	0.000000000
C	0.019444000	0.033677000	0.000000000
F	-0.610948000	-1.058162000	0.000000000

Compound 11

F	0.417786000	-0.190003000	0.000000000
H	-0.417786000	0.190003000	0.000000000

Compound 12

C	-0.554511000	0.059951000	0.000000000
O	0.604365000	0.184296000	0.000000000
F	-1.432240000	1.029277000	0.000000000
F	-1.206614000	-1.073522000	0.000000000

Compound 13

B	-0.451379000	0.132004000	-0.205131000
F	-0.668943000	1.365301000	0.171920000
F	0.638947000	-0.484225000	0.171853000
F	-1.322627000	-0.484081000	-0.961641000

Compound 14

Si	-0.968704000	0.234330000	0.035158000
H	-1.683483000	-0.272206000	1.217498000
F	-1.721065000	-0.298082000	-1.269446000
H	0.413445000	-0.271787000	0.008966000
H	-0.984193000	1.705745000	0.008824000

Compound 15

F	-0.570982000	1.483824000	0.002428000
P	-0.730135000	-0.052826000	0.277927000
F	0.768607000	-0.449820000	0.035785000
F	-1.271490000	-0.452177000	-1.140141000

Compound 16

C	0.122158000	0.167100000	-0.000264000
C	0.707081000	1.208516000	-0.000322000
C	-0.614444000	-1.093177000	-0.000131000
F	0.246730000	-2.169051000	-0.000329000
H	1.233678000	2.130876000	-0.000345000
H	-1.243271000	-1.163047000	0.887118000
H	-1.243632000	-1.163016000	-0.887125000

Compound 17

C	0.294803000	-0.510599000	0.000000000
C	-0.294803000	0.510599000	0.000000000
F	-0.935091000	1.619578000	0.000000000
F	0.935091000	-1.619578000	0.000000000

Compound 18

F	-1.485865000	-0.113869000	-0.633256000
C	-0.091816000	-0.099269000	-0.606604000
C	0.362406000	1.341124000	-0.639450000
C	0.361148000	-0.847395000	0.625168000
H	0.233324000	-0.620893000	-1.508990000
H	1.449586000	1.393618000	-0.670625000
H	-0.035349000	1.846638000	-1.516562000
H	0.014371000	1.861419000	0.251945000

H	1.448267000	-0.901543000	0.655629000
H	-0.037468000	-1.859415000	0.624942000
H	0.013097000	-0.334618000	1.520902000

Compound 19

C	0.018705000	0.190412000	-0.344735000
C	1.284247000	1.024238000	-0.314679000
C	-1.222667000	1.044180000	-0.511663000
F	0.101817000	-0.628208000	-1.481732000
C	-0.083746000	-0.734575000	0.851897000
H	2.157871000	0.377354000	-0.258297000
H	1.282928000	1.685257000	0.550702000
H	1.355700000	1.630285000	-1.216090000
H	-2.104778000	0.412062000	-0.596015000
H	-1.347389000	1.703943000	0.345748000
H	-1.142065000	1.652789000	-1.410598000
H	0.793947000	-1.375911000	0.907514000
H	-0.153378000	-0.157494000	1.772852000
H	-0.969393000	-1.361530000	0.765596000

Compound 20

C	0.985026000	0.110720000	-0.125373000
C	0.189364000	-0.709661000	0.622065000
C	-1.164032000	-0.367796000	0.424893000
C	-1.157120000	0.679901000	-0.461211000
N	0.134485000	0.959401000	-0.785087000
F	0.626782000	-1.687081000	1.418614000
H	2.051641000	0.153644000	-0.234001000
H	-2.021507000	-0.832253000	0.875669000
H	-1.971041000	1.241490000	-0.880721000
H	0.426001000	1.681835000	-1.415550000

Geometries optimized with the aug-cc-pVQZ basis set

Compound 1

C	-0.003282000	-0.036234000	0.051275000
F	1.142507000	0.640166000	0.029836000
F	-1.006613000	0.837679000	0.029815000
F	-0.071674000	-0.780319000	-1.049674000
H	-0.060738000	-0.661292000	0.938647000

Compound 2

C	-0.032219000	-0.055972000	-0.000231000
F	0.656862000	1.137603000	-0.000025000
H	0.682912000	-0.875587000	0.000018000
H	-0.653320000	-0.102949000	0.891579000
H	-0.654236000	-0.103094000	-0.891442000

Compound 3

C	-1.070738000	0.235268000	-0.009308000
F	-1.687296000	-0.219312000	1.063606000
F	-1.683519000	-0.254749000	-1.092074000
F	0.174696000	-0.251194000	-0.022551000
O	-1.090321000	1.576951000	0.020225000
H	-0.643823000	1.922037000	-0.759898000

Compound 4

C	0.549955000	-0.316855000	-0.113428000
C	-0.744501000	0.440210000	-0.099490000
F	1.598904000	0.548304000	-0.090211000
F	0.653451000	-1.088370000	1.001643000
H	0.679569000	-0.968246000	-0.977622000

H	-1.577723000	-0.257615000	-0.094506000
H	-0.809673000	1.071953000	-0.981475000
H	-0.784884000	1.059320000	0.793589000

Compound 5

C	-0.779024000	0.024255000	-0.042034000
C	0.744432000	0.023373000	-0.040598000
F	1.220619000	-0.627555000	1.089044000
H	-1.183183000	1.033027000	-0.025573000
H	-1.183179000	-0.492611000	-0.908494000
H	1.153847000	-0.500712000	-0.902615000
H	-1.127528000	-0.491833000	0.849715000
H	1.153916000	1.031955000	-0.019443000

Compound 6

C	-0.234134000	-0.645486000	0.000000000
C	0.424218000	0.494806000	0.000000000
F	-1.564393000	-0.698673000	0.000000000
F	-0.194771000	1.673478000	0.000000000
H	0.258805000	-1.603860000	0.000000000
H	1.500674000	0.547036000	0.000000000

Compound 7

C	-0.222580000	-0.556104000	0.000000000
C	0.416546000	0.591881000	0.000000000
F	-1.559449000	-0.623889000	0.000000000
H	0.232510000	-1.535827000	0.000000000
H	1.493972000	0.594769000	0.000000000
H	-0.116699000	1.529970000	0.000000000

Compound 8

C	-0.316149000	-0.576710000	0.000000000
C	0.316149000	0.576710000	0.000000000
F	-1.652009000	-0.610497000	0.000000000
F	1.652009000	0.610497000	0.000000000
H	0.160981000	-1.544271000	0.000000000
H	-0.160981000	1.544271000	0.000000000

Compound 9

C	-0.239408000	-0.466826000	0.000000000
C	0.416258000	0.668831000	0.000000000
F	-1.548169000	-0.587999000	0.000000000
F	0.310163000	-1.660823000	0.000000000
H	1.491561000	0.659243000	0.000000000
H	-0.129704000	1.595274000	0.000000000

Compound 10

N	0.591504000	1.024484000	0.000000000
C	0.019444000	0.033677000	0.000000000
F	-0.610948000	-1.058162000	0.000000000

Compound 11

F	0.417971000	-0.190087000	0.000000000
H	-0.417971000	0.190087000	0.000000000

Compound 12

C	-0.554426000	0.059960000	0.000000000
O	0.604724000	0.184335000	0.000000000
F	-1.432451000	1.029148000	0.000000000
F	-1.206847000	-1.073441000	0.000000000

Compound 13

B	-0.451381000	0.132004000	-0.205136000
F	-0.668968000	1.365483000	0.171981000
F	0.639116000	-0.484311000	0.171917000
F	-1.322768000	-0.484177000	-0.961760000

Compound 14

Si	-0.968378000	0.234572000	0.035688000
H	-1.683673000	-0.272353000	1.217466000
F	-1.721182000	-0.298144000	-1.269603000
H	0.413554000	-0.271956000	0.008810000
H	-0.984321000	1.705881000	0.008640000

Compound 15

F	-0.571179000	1.484128000	0.002505000
P	-0.730007000	-0.052743000	0.277869000
F	0.768828000	-0.450109000	0.035879000
F	-1.271641000	-0.452276000	-1.140255000

Compound 16

C	0.122140000	0.167364000	-0.000264000
C	0.707099000	1.208952000	-0.000323000
C	-0.615101000	-1.092292000	-0.000129000
F	0.246838000	-2.168990000	-0.000333000
H	1.234308000	2.130908000	-0.000345000
H	-1.243314000	-1.163885000	0.887379000
H	-1.243671000	-1.163858000	-0.887385000

Compound 17

C	0.294855000	-0.510690000	0.000000000
C	-0.294855000	0.510690000	0.000000000
F	-0.934988000	1.619399000	0.000000000
F	0.934988000	-1.619399000	0.000000000

Compound 18

F	-1.486435000	-0.114281000	-0.633970000
C	-0.091471000	-0.099200000	-0.606484000
C	0.362301000	1.341335000	-0.639356000
C	0.361043000	-0.847419000	0.625397000
H	0.233058000	-0.620860000	-1.508935000
H	1.449513000	1.393209000	-0.670555000
H	-0.035168000	1.846755000	-1.516641000
H	0.014613000	1.861836000	0.252025000
H	1.448195000	-0.901280000	0.655309000
H	-0.037289000	-1.859541000	0.625005000
H	0.013341000	-0.334757000	1.521304000

Compound 19

C	0.018155000	0.190378000	-0.344754000
C	1.284027000	1.024061000	-0.314219000
C	-1.223029000	1.044490000	-0.512265000
F	0.101330000	-0.628610000	-1.482723000
C	-0.083676000	-0.734991000	0.851799000
H	2.158265000	0.378090000	-0.257289000
H	1.281441000	1.684578000	0.551624000
H	1.356197000	1.630757000	-1.215066000
H	-2.105193000	0.412331000	-0.595621000
H	-1.346596000	1.704949000	0.344702000
H	-1.142351000	1.652122000	-1.411752000
H	0.793909000	-1.376641000	0.905963000

H	-0.150878000	-0.157223000	1.772440000
H	-0.969799000	-1.361489000	0.767661000

Compound 20

C	0.985225000	0.110641000	-0.125320000
C	0.189350000	-0.709475000	0.621909000
C	-1.164165000	-0.367874000	0.424968000
C	-1.157268000	0.679849000	-0.461157000
N	0.134593000	0.959428000	-0.785117000
F	0.626723000	-1.687139000	1.418668000
H	2.051809000	0.153657000	-0.234023000
H	-2.021620000	-0.832309000	0.875725000
H	-1.971114000	1.241461000	-0.880692000
H	0.426067000	1.681960000	-1.415660000

Geometries optimized with the cc-pV5Z basis set

Compound 1

C	-0.003282000	-0.036234000	0.051275000
F	1.142507000	0.640166000	0.029836000
F	-1.006613000	0.837679000	0.029815000
F	-0.071674000	-0.780319000	-1.049674000
H	-0.060738000	-0.661292000	0.938647000

Compound 2

C	-0.032219000	-0.055972000	-0.000231000
F	0.656862000	1.137603000	-0.000025000
H	0.682912000	-0.875587000	0.000018000
H	-0.653320000	-0.102949000	0.891579000
H	-0.654236000	-0.103094000	-0.891442000

Compound 3

C	-1.070869000	0.235328000	-0.008991000
F	-1.687396000	-0.219599000	1.063686000
F	-1.683418000	-0.253832000	-1.092164000
F	0.174772000	-0.250370000	-0.022673000
O	-1.090194000	1.576926000	0.020078000
H	-0.643896000	1.920548000	-0.759936000

Compound 4

C	0.549955000	-0.316855000	-0.113428000
C	-0.744501000	0.440210000	-0.099490000
F	1.598904000	0.548304000	-0.090211000
F	0.653451000	-1.088370000	1.001643000
H	0.679569000	-0.968246000	-0.977622000
H	-1.577723000	-0.257615000	-0.094506000
H	-0.809673000	1.071953000	-0.981475000
H	-0.784884000	1.059320000	0.793589000

Compound 5

C	-0.779569000	0.024486000	-0.042436000
C	0.744572000	0.023420000	-0.040643000
F	1.220460000	-0.627723000	1.088631000
H	-1.183523000	1.032991000	-0.025831000
H	-1.183515000	-0.492967000	-0.908240000
H	1.153965000	-0.500612000	-0.902337000
H	-1.126496000	-0.491390000	0.849649000
H	1.154006000	1.031695000	-0.018792000

Compound 6

C	-0.234134000	-0.645486000	0.000000000

C	0.424218000	0.494806000	0.000000000
F	-1.564393000	-0.698673000	0.000000000
F	-0.194771000	1.673478000	0.000000000
H	0.258805000	-1.603860000	0.000000000
H	1.500674000	0.547036000	0.000000000

Compound 7

C	-0.222580000	-0.556104000	0.000000000
C	0.416546000	0.591881000	0.000000000
F	-1.559449000	-0.623889000	0.000000000
H	0.232510000	-1.535827000	0.000000000
H	1.493972000	0.594769000	0.000000000
H	-0.116699000	1.529970000	0.000000000

Compound 8

C	-0.316149000	-0.576710000	0.000000000
C	0.316149000	0.576710000	0.000000000
F	-1.652009000	-0.610497000	0.000000000
F	1.652009000	0.610497000	0.000000000
H	0.160981000	-1.544271000	0.000000000
H	-0.160981000	1.544271000	0.000000000

Compound 9

C	-0.239408000	-0.466826000	0.000000000
C	0.416258000	0.668831000	0.000000000
F	-1.548169000	-0.587999000	0.000000000
F	0.310163000	-1.660823000	0.000000000
H	1.491561000	0.659243000	0.000000000
H	-0.129704000	1.595274000	0.000000000

Compound 10

N	0.591504000	1.024484000	0.000000000
C	0.019444000	0.033677000	0.000000000
F	-0.610948000	-1.058162000	0.000000000

Compound 11

F	0.417660000	-0.189946000	0.000000000
H	-0.417660000	0.189946000	0.000000000

Compound 12

C	-0.554511000	0.059951000	0.000000000
O	0.604365000	0.184296000	0.000000000
F	-1.432240000	1.029277000	0.000000000
F	-1.206614000	-1.073522000	0.000000000

Compound 13

B	-0.451368000	0.132006000	-0.205120000
F	-0.668956000	1.365318000	0.171918000
F	0.638952000	-0.484241000	0.171847000
F	-1.322628000	-0.484083000	-0.961644000

Compound 14

Si	-0.969182000	0.233760000	0.034372000
H	-1.682789000	-0.271422000	1.216975000
F	-1.719949000	-0.297753000	-1.268452000
H	0.411872000	-0.271067000	0.008807000
H	-0.983951000	1.704481000	0.009297000

Compound 15

F	-0.571317000	1.482302000	0.002692000
P	-0.729019000	-0.051853000	0.276227000
F	0.767120000	-0.449657000	0.035876000
F	-1.270783000	-0.451791000	-1.138796000

Compound 16

C	0.121580000	0.167350000	-0.000214000
C	0.707079000	1.208544000	-0.000301000
C	-0.615248000	-1.092508000	-0.000130000
F	0.246728000	-2.168696000	-0.000335000
H	1.234261000	2.130772000	-0.000394000
H	-1.242880000	-1.163668000	0.887419000
H	-1.243220000	-1.163595000	-0.887444000

Compound 17

C	0.294781000	-0.510561000	0.000000000
C	-0.294781000	0.510561000	0.000000000
F	-0.934955000	1.619342000	0.000000000
F	0.934955000	-1.619342000	0.000000000

Compound 18

F	-1.486446000	-0.113875000	-0.633268000
C	-0.091520000	-0.099208000	-0.606497000
C	0.362408000	1.341228000	-0.639389000
C	0.361150000	-0.847394000	0.625288000
H	0.232346000	-0.620915000	-1.509030000
H	1.449532000	1.393024000	-0.669992000
H	-0.034536000	1.846509000	-1.516845000
H	0.014242000	1.861688000	0.251699000
H	1.448214000	-0.900699000	0.655430000

H	-0.036656000	-1.859597000	0.624690000
H	0.012968000	-0.334965000	1.521012000

Compound 19

C	0.018352000	0.190397000	-0.344638000
C	1.284058000	1.024022000	-0.314145000
C	-1.222788000	1.044576000	-0.512182000
F	0.101502000	-0.628603000	-1.482524000
C	-0.083850000	-0.735009000	0.851677000
H	2.158052000	0.377910000	-0.258041000
H	1.281651000	1.683960000	0.551995000
H	1.355635000	1.631126000	-1.214624000
H	-2.105182000	0.412776000	-0.593781000
H	-1.345298000	1.706032000	0.344113000
H	-1.142819000	1.650984000	-1.412425000
H	0.794307000	-1.375560000	0.906542000
H	-0.152785000	-0.157401000	1.772166000
H	-0.969036000	-1.362411000	0.766369000

Compound 20

C	0.985262000	0.110648000	-0.125329000
C	0.189266000	-0.709404000	0.621855000
C	-1.164217000	-0.367870000	0.424968000
C	-1.157279000	0.679911000	-0.461209000
N	0.134547000	0.959441000	-0.785125000
F	0.626693000	-1.686901000	1.418468000
H	2.051868000	0.153511000	-0.233904000
H	-2.021410000	-0.832560000	0.875922000
H	-1.970929000	1.241716000	-0.880920000
H	0.425798000	1.681706000	-1.415427000

Geometries optimized with the aug-cc-pV5Z basis set

Compound 1

C	-0.003282000	-0.036234000	0.051275000
F	1.142507000	0.640166000	0.029836000
F	-1.006613000	0.837679000	0.029815000
F	-0.071674000	-0.780319000	-1.049674000
H	-0.060738000	-0.661292000	0.938647000

Compound 2

C	-0.032219000	-0.055972000	-0.000231000
F	0.656862000	1.137603000	-0.000025000
H	0.682912000	-0.875587000	0.000018000
H	-0.653320000	-0.102949000	0.891579000
H	-0.654236000	-0.103094000	-0.891442000

Compound 3

C	-1.070869000	0.235328000	-0.008991000
F	-1.687396000	-0.219599000	1.063686000
F	-1.683418000	-0.253832000	-1.092164000
F	0.174772000	-0.250370000	-0.022673000
O	-1.090194000	1.576926000	0.020078000
H	-0.643896000	1.920548000	-0.759936000

Compound 4

C	0.549955000	-0.316855000	-0.113428000
C	-0.744501000	0.440210000	-0.099490000
F	1.598904000	0.548304000	-0.090211000
F	0.653451000	-1.088370000	1.001643000
H	0.679569000	-0.968246000	-0.977622000

H	-1.577723000	-0.257615000	-0.094506000
H	-0.809673000	1.071953000	-0.981475000
H	-0.784884000	1.059320000	0.793589000

Compound 5

C	-0.779569000	0.024486000	-0.042436000
C	0.744572000	0.023420000	-0.040643000
F	1.220460000	-0.627723000	1.088631000
H	-1.183523000	1.032991000	-0.025831000
H	-1.183515000	-0.492967000	-0.908240000
H	1.153965000	-0.500612000	-0.902337000
H	-1.126496000	-0.491390000	0.849649000
H	1.154006000	1.031695000	-0.018792000

Compound 6

C	-0.234134000	-0.645486000	0.000000000
C	0.424218000	0.494806000	0.000000000
F	-1.564393000	-0.698673000	0.000000000
F	-0.194771000	1.673478000	0.000000000
H	0.258805000	-1.603860000	0.000000000
H	1.500674000	0.547036000	0.000000000

Compound 7

C	-0.222580000	-0.556104000	0.000000000
C	0.416546000	0.591881000	0.000000000
F	-1.559449000	-0.623889000	0.000000000
H	0.232510000	-1.535827000	0.000000000
H	1.493972000	0.594769000	0.000000000
H	-0.116699000	1.529970000	0.000000000

Compound 8

C	-0.316149000	-0.576710000	0.000000000
C	0.316149000	0.576710000	0.000000000
F	-1.652009000	-0.610497000	0.000000000
F	1.652009000	0.610497000	0.000000000
H	0.160981000	-1.544271000	0.000000000
H	-0.160981000	1.544271000	0.000000000

Compound 9

C	-0.239408000	-0.466826000	0.000000000
C	0.416258000	0.668831000	0.000000000
F	-1.548169000	-0.587999000	0.000000000
F	0.310163000	-1.660823000	0.000000000
H	1.491561000	0.659243000	0.000000000
H	-0.129704000	1.595274000	0.000000000

Compound 10

N	0.591504000	1.024484000	0.000000000
C	0.019444000	0.033677000	0.000000000
F	-0.610948000	-1.058162000	0.000000000

Compound 11

F	0.417786000	-0.190003000	0.000000000
H	-0.417786000	0.190003000	0.000000000

Compound 12

C	-0.554511000	0.059951000	0.000000000
O	0.604365000	0.184296000	0.000000000
F	-1.432240000	1.029277000	0.000000000
F	-1.206614000	-1.073522000	0.000000000

Compound 13

B	-0.451368000	0.132005000	-0.205119000
F	-0.668957000	1.365317000	0.171917000
F	0.638952000	-0.484241000	0.171847000
F	-1.322629000	-0.484082000	-0.961645000

Compound 14

Si	-0.969144000	0.233780000	0.034429000
H	-1.682813000	-0.271427000	1.216963000
F	-1.719938000	-0.297749000	-1.268443000
H	0.411874000	-0.271094000	0.008774000
H	-0.983979000	1.704490000	0.009277000

Compound 15

F	-0.571379000	1.482357000	0.002777000
P	-0.728880000	-0.051793000	0.275995000
F	0.767205000	-0.449723000	0.036020000
F	-1.270946000	-0.451841000	-1.138792000

Compound 16

C	0.121480000	0.167441000	-0.000214000
C	0.707215000	1.208530000	-0.000301000
C	-0.615264000	-1.092429000	-0.000130000
F	0.246756000	-2.168667000	-0.000335000
H	1.234291000	2.130802000	-0.000394000
H	-1.242919000	-1.163775000	0.887399000
H	-1.243260000	-1.163702000	-0.887425000

Compound 17

C	0.294789000	-0.510574000	0.000000000
C	-0.294789000	0.510574000	0.000000000
F	-0.934928000	1.619295000	0.000000000
F	0.934928000	-1.619295000	0.000000000

Compound 18

F	-1.486374000	-0.113884000	-0.633283000
C	-0.091337000	-0.099169000	-0.606430000
C	0.362411000	1.341247000	-0.639377000
C	0.361153000	-0.847394000	0.625310000
H	0.232474000	-0.620915000	-1.509031000
H	1.449573000	1.393113000	-0.670130000
H	-0.034765000	1.846432000	-1.516838000
H	0.014235000	1.861676000	0.251774000
H	1.448254000	-0.900863000	0.655438000
H	-0.036885000	-1.859551000	0.624626000
H	0.012962000	-0.334894000	1.521039000

Compound 19

C	0.018277000	0.190437000	-0.344398000
C	1.283885000	1.024147000	-0.314287000
C	-1.222808000	1.044399000	-0.511820000
F	0.101406000	-0.628568000	-1.482355000
C	-0.083815000	-0.735015000	0.851705000
H	2.157842000	0.377974000	-0.257660000
H	1.281419000	1.684745000	0.551423000
H	1.356031000	1.630610000	-1.215189000
H	-2.105327000	0.412751000	-0.594167000
H	-1.345486000	1.705792000	0.344612000
H	-1.142041000	1.650944000	-1.411930000
H	0.794296000	-1.375627000	0.906368000
H	-0.152722000	-0.157523000	1.772357000

H	-0.969158000	-1.362267000	0.765843000

Compound 20

C	0.985333000	0.110632000	-0.125320000
C	0.189282000	-0.709392000	0.621843000
C	-1.164255000	-0.367892000	0.424989000
C	-1.157327000	0.679894000	-0.461191000
N	0.134587000	0.959449000	-0.785135000
F	0.626648000	-1.686896000	1.418467000
H	2.051943000	0.153512000	-0.233910000
H	-2.021463000	-0.832590000	0.875951000
H	-1.970978000	1.241710000	-0.880912000
H	0.425827000	1.681771000	-1.415484000

Geometries optimized with the cc-pV6Z basis set

Compound 1

C	-0.003282000	-0.036234000	0.051275000
F	1.142507000	0.640166000	0.029836000
F	-1.006613000	0.837679000	0.029815000
F	-0.071674000	-0.780319000	-1.049674000
H	-0.060738000	-0.661292000	0.938647000

Compound 2

C	-0.032219000	-0.055972000	-0.000231000
F	0.656862000	1.137603000	-0.000025000
H	0.682912000	-0.875587000	0.000018000
H	-0.653320000	-0.102949000	0.891579000
H	-0.654236000	-0.103094000	-0.891442000

Compound 3

C	-1.070869000	0.235328000	-0.008991000
F	-1.687396000	-0.219599000	1.063686000
F	-1.683418000	-0.253832000	-1.092164000
F	0.174772000	-0.250370000	-0.022673000
O	-1.090194000	1.576926000	0.020078000
H	-0.643896000	1.920548000	-0.759936000

Compound 4

C	0.549955000	-0.316855000	-0.113428000
C	-0.744501000	0.440210000	-0.099490000
F	1.598904000	0.548304000	-0.090211000
F	0.653451000	-1.088370000	1.001643000
H	0.679569000	-0.968246000	-0.977622000
H	-1.577723000	-0.257615000	-0.094506000
H	-0.809673000	1.071953000	-0.981475000
H	-0.784884000	1.059320000	0.793589000

Compound 5

C	-0.779569000	0.024486000	-0.042436000
C	0.744572000	0.023420000	-0.040643000
F	1.220460000	-0.627723000	1.088631000
H	-1.183523000	1.032991000	-0.025831000
H	-1.183515000	-0.492967000	-0.908240000
H	1.153965000	-0.500612000	-0.902337000
H	-1.126496000	-0.491390000	0.849649000
H	1.154006000	1.031695000	-0.018792000

Compound 6

C	-0.234134000	-0.645486000	0.000000000

C	0.424218000	0.494806000	0.000000000
F	-1.564393000	-0.698673000	0.000000000
F	-0.194771000	1.673478000	0.000000000
H	0.258805000	-1.603860000	0.000000000
H	1.500674000	0.547036000	0.000000000

Compound 7

C	-0.222580000	-0.556104000	0.000000000
C	0.416546000	0.591881000	0.000000000
F	-1.559449000	-0.623889000	0.000000000
H	0.232510000	-1.535827000	0.000000000
H	1.493972000	0.594769000	0.000000000
H	-0.116699000	1.529970000	0.000000000

Compound 8

C	-0.316149000	-0.576710000	0.000000000
C	0.316149000	0.576710000	0.000000000
F	-1.652009000	-0.610497000	0.000000000
F	1.652009000	0.610497000	0.000000000
H	0.160981000	-1.544271000	0.000000000
H	-0.160981000	1.544271000	0.000000000

Compound 9

C	-0.239408000	-0.466826000	0.000000000
C	0.416258000	0.668831000	0.000000000
F	-1.548169000	-0.587999000	0.000000000
F	0.310163000	-1.660823000	0.000000000
H	1.491561000	0.659243000	0.000000000
H	-0.129704000	1.595274000	0.000000000

Compound 10

N	0.591504000	1.024484000	0.000000000
C	0.019444000	0.033677000	0.000000000
F	-0.610948000	-1.058162000	0.000000000

Compound 11

F	0.417786000	-0.190003000	0.000000000
H	-0.417786000	0.190003000	0.000000000

Compound 12

C	-0.554511000	0.059951000	0.000000000
O	0.604365000	0.184296000	0.000000000
F	-1.432240000	1.029277000	0.000000000
F	-1.206614000	-1.073522000	0.000000000

Compound 13

B	-0.451359000	0.132006000	-0.205109000
F	-0.668970000	1.365342000	0.171918000
F	0.638990000	-0.484259000	0.171860000
F	-1.322662000	-0.484090000	-0.961668000

Compound 14

Si	-0.969085000	0.233288000	0.034299000
H	-1.682751000	-0.270791000	1.217580000
F	-1.719705000	-0.296936000	-1.268787000
H	0.411696000	-0.270573000	0.008745000
H	-0.984155000	1.703012000	0.009163000

Compound 15

F	-0.571995000	1.481770000	0.002155000
P	-0.728619000	-0.051855000	0.276179000
F	0.766657000	-0.450040000	0.035705000
F	-1.270044000	-0.450875000	-1.138040000

Compound 16

C	0.122055000	0.167602000	-0.000257000
C	0.707154000	1.209096000	-0.000320000
C	-0.615328000	-1.092257000	-0.000129000
F	0.246456000	-2.168867000	-0.000334000
H	1.234384000	2.130837000	-0.000352000
H	-1.243034000	-1.164122000	0.887284000
H	-1.243387000	-1.164090000	-0.887292000

Compound 17

C	0.294789000	-0.510575000	0.000000000
C	-0.294789000	0.510575000	0.000000000
F	-0.934944000	1.619322000	0.000000000
F	0.934944000	-1.619322000	0.000000000

Compound 18

F	-1.485953000	-0.113962000	-0.633416000
C	-0.090939000	-0.099379000	-0.606793000
C	0.362445000	1.341297000	-0.639392000
C	0.361187000	-0.847432000	0.625346000
H	0.233034000	-0.621067000	-1.509293000
H	1.449474000	1.393735000	-0.670635000
H	-0.035619000	1.846577000	-1.516281000
H	0.014464000	1.861119000	0.252093000
H	1.448156000	-0.901615000	0.655722000

H	-0.037741000	-1.859139000	0.625031000
H	0.013195000	-0.334338000	1.520717000

Compound 19

C	0.018360000	0.190617000	-0.344445000
C	1.284072000	1.024483000	-0.313763000
C	-1.222997000	1.044418000	-0.512466000
F	0.102137000	-0.628314000	-1.482543000
C	-0.083710000	-0.735178000	0.851737000
H	2.157668000	0.377917000	-0.257863000
H	1.281752000	1.684106000	0.552530000
H	1.355442000	1.631508000	-1.214205000
H	-2.105209000	0.412385000	-0.593592000
H	-1.345507000	1.706435000	0.343325000
H	-1.143032000	1.650067000	-1.413119000
H	0.793535000	-1.376973000	0.905363000
H	-0.150663000	-0.157707000	1.772381000
H	-0.970049000	-1.360963000	0.767159000

Compound 20

C	0.985347000	0.110644000	-0.125331000
C	0.189305000	-0.709424000	0.621868000
C	-1.164176000	-0.367872000	0.424967000
C	-1.157206000	0.679971000	-0.461264000
N	0.134614000	0.959441000	-0.785129000
F	0.626751000	-1.686925000	1.418485000
H	2.051835000	0.153558000	-0.233942000
H	-2.021584000	-0.832208000	0.875636000
H	-1.971157000	1.241365000	-0.880608000
H	0.425870000	1.681649000	-1.415383000

Geometries optimized with the pc-1 basis set

Compound 1

C	-0.003245000	-0.036193000	0.051467000
F	1.144599000	0.641735000	0.029106000
F	-1.008279000	0.839398000	0.028992000
F	-0.071855000	-0.780953000	-1.052197000
H	-0.061021000	-0.663986000	0.942531000

Compound 2

C	-0.032037000	-0.055268000	-0.000076000
F	0.658708000	1.139691000	-0.000204000
H	0.688122000	-0.879254000	-0.000080000
H	-0.657087000	-0.102589000	0.896986000
H	-0.657706000	-0.102579000	-0.896726000

Compound 3

C	-1.071689000	0.236866000	-0.007687000
F	-1.688848000	-0.221519000	1.066316000
F	-1.684158000	-0.252772000	-1.094780000
F	0.177385000	-0.249152000	-0.023442000
O	-1.092335000	1.577097000	0.023553000
H	-0.641355000	1.918481000	-0.763961000

Compound 4

C	0.548919000	-0.317980000	-0.116014000
C	-0.743597000	0.438148000	-0.101797000
F	1.596362000	0.554775000	-0.090734000

F	0.647430000	-1.087919000	1.005132000
H	0.688754000	-0.975343000	-0.980308000
H	-1.584786000	-0.259922000	-0.091908000
H	-0.811465000	1.078774000	-0.984970000
H	-0.776520000	1.058168000	0.799100000

Compound 5

C	-0.770575000	0.009398000	-0.035778000
C	0.736359000	-0.005045000	-0.047803000
F	1.206601000	-0.069999000	1.257698000
H	-1.137259000	0.877027000	0.521742000
H	-1.160909000	0.059263000	-1.057997000
H	1.131041000	-0.877612000	-0.580929000
H	-1.154971000	-0.897576000	0.441654000
H	1.149614000	0.904443000	-0.498585000

Compound 6

C	-0.231277000	-0.649654000	0.000000000
C	0.429309000	0.494248000	0.000000000
F	-1.567070000	-0.689163000	0.000000000
F	-0.204461000	1.671195000	0.000000000
H	0.251782000	-1.621010000	0.000000000
H	1.512117000	0.561684000	0.000000000

Compound 7

C	-0.222018000	-0.558876000	0.000000000
C	0.419943000	0.593079000	0.000000000
F	-1.563469000	-0.620593000	0.000000000
H	0.229417000	-1.548291000	0.000000000
H	1.505269000	0.601826000	0.000000000
H	-0.124842000	1.533654000	0.000000000

Compound 8

C	-0.316160000	-0.579366000	0.000000000
C	0.316160000	0.579366000	0.000000000
F	-1.656255000	-0.609953000	0.000000000
F	1.656255000	0.609953000	0.000000000
H	0.163681000	-1.553675000	0.000000000
H	-0.163681000	1.553675000	0.000000000

Compound 9

C	-0.240723000	-0.469145000	0.000000000
C	0.417094000	0.670274000	0.000000000
F	-1.554350000	-0.589377000	0.000000000
F	0.312038000	-1.666871000	0.000000000
H	1.500360000	0.659707000	0.000000000
H	-0.133718000	1.603111000	0.000000000

Compound 10

N	0.594844000	1.030270000	0.000000000
C	0.019427000	0.033648000	0.000000000
F	-0.614272000	-1.063919000	0.000000000

Compound 11

F	0.421351000	-0.191624000	0.000000000
H	-0.421351000	0.191624000	0.000000000

Compound 12

C	-0.553127000	0.060100000	0.000000000
O	0.608768000	0.184768000	0.000000000
F	-1.435376000	1.031229000	0.000000000
F	-1.209265000	-1.076095000	0.000000000

Compound 13

B	-0.450958000	0.132380000	-0.205958000
F	-0.670122000	1.371301000	0.174099000
F	0.643971000	-0.487372000	0.173878000
F	-1.326893000	-0.487310000	-0.965018000

Compound 14

Si	-0.966928000	0.235109000	0.038646000
H	-1.682364000	-0.271048000	1.223874000
F	-1.730542000	-0.304684000	-1.286836000
H	0.417291000	-0.270002000	0.012630000
H	-0.981457000	1.708625000	0.012687000

Compound 15

F	-0.572740000	1.506100000	0.003367000
P	-0.737469000	-0.057921000	0.291369000
F	0.789314000	-0.459200000	0.037748000
F	-1.283105000	-0.459979000	-1.156485000

Compound 16

C	0.120207000	0.165559000	-0.000242000
C	0.709593000	1.213919000	-0.000314000
C	-0.618297000	-1.097044000	-0.000130000
F	0.248018000	-2.172470000	-0.000332000
H	1.242873000	2.143261000	-0.000368000
H	-1.246870000	-1.167538000	0.893541000
H	-1.247225000	-1.167487000	-0.893556000

Compound 17

C	0.296543000	-0.513612000	0.000000000
C	-0.296543000	0.513612000	0.000000000
F	-0.940456000	1.628869000	0.000000000
F	0.940456000	-1.628869000	0.000000000

Compound 18

F	-1.482440000	-0.109811000	-0.626229000
C	-0.083485000	-0.098203000	-0.604750000
C	0.364351000	1.344411000	-0.641917000
C	0.363090000	-0.851176000	0.626784000
H	0.236599000	-0.623802000	-1.514022000
H	1.457182000	1.410309000	-0.679391000
H	-0.047620000	1.845485000	-1.523735000
H	0.009599000	1.869086000	0.252480000
H	1.455845000	-0.917483000	0.665714000
H	-0.049744000	-1.865043000	0.620356000
H	0.008323000	-0.337977000	1.527809000

Compound 19

C	0.017550000	0.197537000	-0.335070000
C	1.287024000	1.028000000	-0.312183000
C	-1.226349000	1.047630000	-0.510774000
F	0.101827000	-0.623223000	-1.477713000
C	-0.083948000	-0.736889000	0.855629000
H	2.162651000	0.371864000	-0.260500000
H	1.297126000	1.699716000	0.553455000
H	1.358956000	1.628207000	-1.225863000
H	-2.109776000	0.405866000	-0.598763000
H	-1.362036000	1.719963000	0.343700000
H	-1.143057000	1.648494000	-1.423060000
H	0.797350000	-1.386160000	0.899754000
H	-0.151649000	-0.169483000	1.790523000

H	-0.973869000	-1.368722000	0.761365000

Compound 20

C	0.988718000	0.110527000	-0.125463000
C	0.189032000	-0.711695000	0.623808000
C	-1.166832000	-0.369781000	0.426759000
C	-1.160202000	0.681380000	-0.462251000
N	0.135085000	0.960064000	-0.785687000
F	0.629828000	-1.692352000	1.422866000
H	2.062356000	0.155002000	-0.235878000
H	-2.029627000	-0.838034000	0.881104000
H	-1.977995000	1.248197000	-0.885919000
H	0.429236000	1.686890000	-1.420040000

Geometries optimized with the aug-pc-1 basis set

Compound 1

C	-0.003238000	-0.036654000	0.052178000
F	1.145951000	0.642472000	0.028819000
F	-1.009582000	0.840547000	0.029203000
F	-0.071633000	-0.781875000	-1.053368000
H	-0.061298000	-0.664490000	0.943067000

Compound 2

C	-0.034231000	-0.059831000	-0.000026000
F	0.657683000	1.139701000	0.000101000
H	0.690572000	-0.879580000	-0.000451000
H	-0.656739000	-0.099777000	0.898814000
H	-0.657285000	-0.100512000	-0.898540000

Compound 3

C	-1.071232000	0.237007000	-0.008448000
F	-1.689345000	-0.221771000	1.067142000
F	-1.684650000	-0.254647000	-1.095209000
F	0.178019000	-0.251071000	-0.023200000
O	-1.091672000	1.578942000	0.022484000
H	-0.642120000	1.920541000	-0.762769000

Compound 4

C	0.547509000	-0.317545000	-0.116582000
C	-0.745165000	0.440331000	-0.099883000
F	1.599161000	0.552711000	-0.089854000
F	0.650902000	-1.088819000	1.005237000
H	0.686803000	-0.974765000	-0.981132000
H	-1.583797000	-0.261634000	-0.093112000
H	-0.810256000	1.077442000	-0.986427000
H	-0.780058000	1.060980000	0.800254000

Compound 5

C	-0.779437000	0.024495000	-0.042442000
C	0.743294000	0.025203000	-0.043938000
F	1.219028000	-0.627822000	1.092570000
H	-1.188112000	1.039042000	-0.023660000
H	-1.188150000	-0.494685000	-0.914282000
H	1.159524000	-0.506864000	-0.905318000
H	-1.125964000	-0.496720000	0.855133000
H	1.159717000	1.037251000	-0.018063000

Compound 6

C	-0.231591000	-0.649634000	0.000000000

C	0.429148000	0.494522000	0.000000000
F	-1.567778000	-0.689301000	0.000000000
F	-0.204718000	1.671871000	0.000000000
H	0.253921000	-1.618938000	0.000000000
H	1.511418000	0.558782000	0.000000000

Compound 7

C	-0.220023000	-0.559329000	0.000000000
C	0.418624000	0.594690000	0.000000000
F	-1.564229000	-0.620550000	0.000000000
H	0.230846000	-1.547945000	0.000000000
H	1.503321000	0.598844000	0.000000000
H	-0.124239000	1.535090000	0.000000000

Compound 8

C	-0.314810000	-0.580183000	0.000000000
C	0.314810000	0.580183000	0.000000000
F	-1.656526000	-0.610204000	0.000000000
F	1.656526000	0.610204000	0.000000000
H	0.162929000	-1.554451000	0.000000000
H	-0.162929000	1.554451000	0.000000000

Compound 9

C	-0.240086000	-0.468042000	0.000000000
C	0.417728000	0.671374000	0.000000000
F	-1.554583000	-0.589370000	0.000000000
F	0.312161000	-1.667069000	0.000000000
H	1.499890000	0.658638000	0.000000000
H	-0.134410000	1.602169000	0.000000000

Compound 10

N	0.594771000	1.030142000	0.000000000
C	0.019408000	0.033613000	0.000000000
F	-0.614178000	-1.063757000	0.000000000

Compound 11

F	0.420405000	-0.191194000	0.000000000
H	-0.420405000	0.191194000	0.000000000

Compound 12

C	-0.552798000	0.060135000	0.000000000
O	0.610430000	0.184947000	0.000000000
F	-1.436411000	1.031488000	0.000000000
F	-1.210221000	-1.076568000	0.000000000

Compound 13

B	-0.450875000	0.131951000	-0.206069000
F	-0.669970000	1.370249000	0.173751000
F	0.643905000	-0.486662000	0.174124000
F	-1.327060000	-0.486539000	-0.964805000

Compound 14

Si	-0.962586000	0.238083000	0.045443000
H	-1.687682000	-0.274396000	1.224783000
F	-1.730660000	-0.304885000	-1.286875000
H	0.420929000	-0.273842000	0.008576000
H	-0.984002000	1.713040000	0.009073000

Compound 15

F	-0.572018000	1.507380000	0.002461000
P	-0.739708000	-0.059169000	0.295416000
F	0.789910000	-0.459060000	0.036179000
F	-1.282184000	-0.460151000	-1.158057000

Compound 16

C	0.119297000	0.166554000	-0.000262000
C	0.711034000	1.214204000	-0.000322000
C	-0.620299000	-1.093591000	-0.000129000
F	0.249884000	-2.170123000	-0.000333000
H	1.244354000	2.142437000	-0.000349000
H	-1.247805000	-1.170657000	0.893876000
H	-1.248166000	-1.170624000	-0.893882000

Compound 17

C	0.296435000	-0.513425000	0.000000000
C	-0.296435000	0.513425000	0.000000000
F	-0.939674000	1.627516000	0.000000000
F	0.939674000	-1.627516000	0.000000000

Compound 18

F	-1.486575000	-0.111798000	-0.629673000
C	-0.085441000	-0.098391000	-0.605078000
C	0.362859000	1.344548000	-0.641128000
C	0.361598000	-0.850559000	0.627296000
H	0.233166000	-0.624035000	-1.514430000
H	1.456729000	1.401360000	-0.674121000
H	-0.041962000	1.847140000	-1.524646000
H	0.010644000	1.868183000	0.254775000
H	1.455402000	-0.908448000	0.660592000

H	-0.044088000	-1.866663000	0.621338000
H	0.009370000	-0.335539000	1.528174000

Compound 19

C	0.017875000	0.194659000	-0.339417000
C	1.287195000	1.025840000	-0.314239000
C	-1.226008000	1.046477000	-0.512670000
F	0.101352000	-0.627366000	-1.483209000
C	-0.083917000	-0.737839000	0.853312000
H	2.163809000	0.372394000	-0.257496000
H	1.287900000	1.692493000	0.555467000
H	1.358134000	1.632217000	-1.222941000
H	-2.111348000	0.407730000	-0.594186000
H	-1.351783000	1.714911000	0.346513000
H	-1.143725000	1.651940000	-1.421054000
H	0.800244000	-1.381437000	0.904881000
H	-0.153837000	-0.160528000	1.782161000
H	-0.974091000	-1.368689000	0.763380000

Compound 20

C	0.989614000	0.110612000	-0.125596000
C	0.188662000	-0.710433000	0.622766000
C	-1.166796000	-0.369875000	0.426836000
C	-1.160400000	0.681591000	-0.462416000
N	0.135533000	0.960551000	-0.786129000
F	0.629384000	-1.692089000	1.422675000
H	2.062344000	0.154335000	-0.235313000
H	-2.029024000	-0.837758000	0.880830000
H	-1.978111000	1.247523000	-0.885341000
H	0.428395000	1.685743000	-1.419014000

Geometries optimized with the pc-2 basis set

Compound 1

C	-0.003221000	-0.036093000	0.050976000
F	1.142930000	0.640469000	0.030118000
F	-1.006891000	0.837877000	0.029998000
F	-0.071844000	-0.780861000	-1.050045000
H	-0.060773000	-0.661392000	0.938852000

Compound 2

C	-0.032149000	-0.055546000	-0.000005000
F	0.657518000	1.137735000	-0.000335000
H	0.683588000	-0.875694000	-0.000162000
H	-0.654305000	-0.103306000	0.892055000
H	-0.654653000	-0.103188000	-0.891654000

Compound 3

C	-1.070821000	0.235468000	-0.009161000
F	-1.687469000	-0.219456000	1.063906000
F	-1.683541000	-0.254449000	-1.092524000
F	0.175098000	-0.250898000	-0.022760000
O	-1.090497000	1.577506000	0.020524000
H	-0.643770000	1.920829000	-0.759985000

Compound 4

C	0.550203000	-0.316622000	-0.112866000
C	-0.744574000	0.440268000	-0.099467000
F	1.599746000	0.548697000	-0.090538000

F	0.653578000	-1.089212000	1.002137000
H	0.678629000	-0.967963000	-0.978012000
H	-1.577859000	-0.258425000	-0.094530000
H	-0.809243000	1.072123000	-0.982154000
H	-0.785381000	1.059836000	0.793930000

Compound 5

C	-0.778893000	0.024197000	-0.041934000
C	0.744573000	0.023071000	-0.040071000
F	1.221056000	-0.627869000	1.089567000
H	-1.182565000	1.033901000	-0.025774000
H	-1.182558000	-0.492889000	-0.909342000
H	1.152893000	-0.501150000	-0.903340000
H	-1.127568000	-0.492163000	0.850306000
H	1.152961000	1.032802000	-0.019411000

Compound 6

C	-0.234405000	-0.645396000	0.000000000
C	0.424026000	0.494966000	0.000000000
F	-1.565293000	-0.700280000	0.000000000
F	-0.193982000	1.675079000	0.000000000
H	0.258656000	-1.604489000	0.000000000
H	1.501396000	0.547421000	0.000000000

Compound 7

C	-0.222986000	-0.555838000	0.000000000
C	0.416804000	0.591947000	0.000000000
F	-1.560453000	-0.624867000	0.000000000
H	0.232438000	-1.536314000	0.000000000
H	1.494954000	0.595278000	0.000000000
H	-0.116457000	1.530594000	0.000000000

Compound 8

C	-0.316515000	-0.576599000	0.000000000
C	0.316515000	0.576599000	0.000000000
F	-1.653166000	-0.611370000	0.000000000
F	1.653166000	0.611370000	0.000000000
H	0.160716000	-1.544770000	0.000000000
H	-0.160716000	1.544770000	0.000000000

Compound 9

C	-0.239412000	-0.466855000	0.000000000
C	0.416303000	0.668909000	0.000000000
F	-1.548997000	-0.588430000	0.000000000
F	0.310197000	-1.661750000	0.000000000
H	1.492052000	0.659833000	0.000000000
H	-0.129441000	1.595993000	0.000000000

Compound 10

N	0.591817000	1.025027000	0.000000000
C	0.019529000	0.033824000	0.000000000
F	-0.611346000	-1.058851000	0.000000000

Compound 11

F	0.417786000	-0.190003000	0.000000000
H	-0.417786000	0.190003000	0.000000000

Compound 12

C	-0.554085000	0.059997000	0.000000000
O	0.605312000	0.184398000	0.000000000
F	-1.432938000	1.029311000	0.000000000

F	-1.207289000	-1.073704000	0.000000000

Compound 13

B	-0.451372000	0.131999000	-0.205120000
F	-0.668985000	1.365479000	0.171966000
F	0.639122000	-0.484320000	0.171912000
F	-1.322766000	-0.484159000	-0.961757000

Compound 14

Si	-0.969600000	0.233427000	0.033595000
H	-1.682570000	-0.271236000	1.217581000
F	-1.720418000	-0.298037000	-1.269260000
H	0.412290000	-0.270911000	0.009305000
H	-0.983703000	1.704756000	0.009779000

Compound 15

F	-0.571244000	1.482118000	0.002149000
P	-0.729538000	-0.052239000	0.277358000
F	0.766988000	-0.449524000	0.035367000
F	-1.270206000	-0.451354000	-1.138874000

Compound 16

C	0.120872000	0.167419000	-0.000218000
C	0.707356000	1.208652000	-0.000303000
C	-0.614787000	-1.093182000	-0.000130000
F	0.246796000	-2.169709000	-0.000336000
H	1.234457000	2.131560000	-0.000390000
H	-1.243027000	-1.163304000	0.887952000
H	-1.243368000	-1.163235000	-0.887976000

Compound 17

C	0.294898000	-0.510763000	0.000000000
C	-0.294898000	0.510763000	0.000000000
F	-0.935466000	1.620227000	0.000000000
F	0.935466000	-1.620227000	0.000000000

Compound 18

F	-1.487090000	-0.114298000	-0.634001000
C	-0.092233000	-0.099455000	-0.606926000
C	0.362535000	1.341249000	-0.639243000
C	0.361276000	-0.847279000	0.625380000
H	0.233027000	-0.621371000	-1.509819000
H	1.450387000	1.391846000	-0.669724000
H	-0.035089000	1.847443000	-1.516736000
H	0.014152000	1.861150000	0.252917000
H	1.449069000	-0.899875000	0.654547000
H	-0.037207000	-1.859969000	0.625551000
H	0.012874000	-0.333643000	1.521154000

Compound 19

C	0.018287000	0.189573000	-0.345769000
C	1.284215000	1.024060000	-0.314305000
C	-1.223220000	1.044625000	-0.512273000
F	0.101476000	-0.629439000	-1.483474000
C	-0.083643000	-0.735056000	0.852095000
H	2.158753000	0.377421000	-0.257752000
H	1.280202000	1.683789000	0.552807000
H	1.355785000	1.631549000	-1.215369000
H	-2.106206000	0.412590000	-0.595724000
H	-1.345012000	1.704639000	0.346031000
H	-1.142262000	1.652840000	-1.412047000
H	0.795017000	-1.376214000	0.906967000
H	-0.151820000	-0.155149000	1.772027000

H	-0.969772000	-1.362427000	0.767288000

Compound 20

C	0.985313000	0.110592000	-0.125284000
C	0.189119000	-0.709520000	0.621962000
C	-1.164044000	-0.367830000	0.424922000
C	-1.157414000	0.679949000	-0.461232000
N	0.134707000	0.959815000	-0.785452000
F	0.626980000	-1.687805000	1.419213000
H	2.052469000	0.153614000	-0.234032000
H	-2.022040000	-0.832554000	0.875960000
H	-1.972156000	1.241532000	-0.880682000
H	0.426663000	1.682405000	-1.416077000

Geometries optimized with the aug-pc-2 basis set

Compound 1

C	-0.003247000	-0.036374000	0.051400000
F	1.142984000	0.640660000	0.029792000
F	-1.006909000	0.838074000	0.029677000
F	-0.071818000	-0.780611000	-1.050363000
H	-0.060809000	-0.661750000	0.939393000

Compound 2

C	-0.032473000	-0.056111000	-0.000004000
F	0.657356000	1.137467000	-0.000343000
H	0.684066000	-0.875606000	-0.000157000
H	-0.654302000	-0.102942000	0.892379000
H	-0.654647000	-0.102807000	-0.891976000

Compound 3

C	-1.070849000	0.235433000	-0.009112000
F	-1.687650000	-0.219805000	1.064220000
F	-1.683687000	-0.254666000	-1.092639000
F	0.175271000	-0.251118000	-0.022688000
O	-1.090437000	1.577666000	0.020426000
H	-0.643649000	1.921491000	-0.760208000

Compound 4

C	0.549934000	-0.316774000	-0.113326000
C	-0.744700000	0.440305000	-0.099520000
F	1.599683000	0.548824000	-0.090222000
F	0.653593000	-1.088950000	1.002364000
H	0.679129000	-0.968373000	-0.978193000
H	-1.578129000	-0.258312000	-0.094511000
H	-0.809477000	1.072298000	-0.982177000
H	-0.784935000	1.059682000	0.794085000

Compound 5

C	-0.779213000	0.024169000	-0.041886000
C	0.744509000	0.023353000	-0.040561000
F	1.220920000	-0.627784000	1.089410000
H	-1.183069000	1.033727000	-0.025674000
H	-1.183063000	-0.492898000	-0.909136000
H	1.153790000	-0.501135000	-0.903170000
H	-1.127832000	-0.492180000	0.850347000
H	1.153858000	1.032648000	-0.019329000

Compound 6

C	-0.234293000	-0.645602000	0.000000000
C	0.424262000	0.494983000	0.000000000

F	-1.565382000	-0.699491000	0.000000000
F	-0.194715000	1.674767000	0.000000000
H	0.258968000	-1.604502000	0.000000000
H	1.501559000	0.547146000	0.000000000

Compound 7

C	-0.222728000	-0.556032000	0.000000000
C	0.416625000	0.592144000	0.000000000
F	-1.560452000	-0.624432000	0.000000000
H	0.232840000	-1.536394000	0.000000000
H	1.494711000	0.594752000	0.000000000
H	-0.116697000	1.530761000	0.000000000

Compound 8

C	-0.316261000	-0.576791000	0.000000000
C	0.316261000	0.576791000	0.000000000
F	-1.653053000	-0.610974000	0.000000000
F	1.653053000	0.610974000	0.000000000
H	0.160984000	-1.544993000	0.000000000
H	-0.160984000	1.544993000	0.000000000

Compound 9

C	-0.239334000	-0.466719000	0.000000000
C	0.416479000	0.669214000	0.000000000
F	-1.548932000	-0.588579000	0.000000000
F	0.310035000	-1.661767000	0.000000000
H	1.492194000	0.659568000	0.000000000
H	-0.129741000	1.595984000	0.000000000

Compound 10

N	0.591883000	1.025140000	0.000000000
C	0.019522000	0.033812000	0.000000000
F	-0.611405000	-1.058954000	0.000000000

Compound 11

F	0.418091000	-0.190142000	0.000000000
H	-0.418091000	0.190142000	0.000000000

Compound 12

C	-0.554056000	0.060000000	0.000000000
O	0.605628000	0.184432000	0.000000000
F	-1.433120000	1.029380000	0.000000000
F	-1.207452000	-1.073810000	0.000000000

Compound 13

B	-0.450588000	0.132571000	-0.206459000
F	-0.669465000	1.366267000	0.172685000
F	0.639570000	-0.485043000	0.172551000
F	-1.323518000	-0.484795000	-0.961776000

Compound 14

Si	-0.968977000	0.233760000	0.034890000
H	-1.683631000	-0.271822000	1.217758000
F	-1.720842000	-0.298371000	-1.269621000
H	0.413182000	-0.271189000	0.008669000
H	-0.983731000	1.705622000	0.009304000

Compound 15

F	-0.572007000	1.485050000	0.002892000
P	-0.729097000	-0.052307000	0.277117000
F	0.769383000	-0.451147000	0.036435000
F	-1.272279000	-0.452596000	-1.140445000

Compound 16

C	0.121499000	0.167185000	-0.000215000
C	0.707516000	1.208758000	-0.000301000
C	-0.615377000	-1.092535000	-0.000130000
F	0.247197000	-2.169253000	-0.000336000
H	1.234612000	2.131684000	-0.000393000
H	-1.243404000	-1.163855000	0.888058000
H	-1.243744000	-1.163785000	-0.888082000

Compound 17

C	0.294912000	-0.510787000	0.000000000
C	-0.294912000	0.510787000	0.000000000
F	-0.935563000	1.620394000	0.000000000
F	0.935563000	-1.620394000	0.000000000

Compound 18

F	-1.486739000	-0.114383000	-0.634148000
C	-0.091315000	-0.099381000	-0.606796000
C	0.362482000	1.341589000	-0.639307000
C	0.361223000	-0.847504000	0.625642000
H	0.233340000	-0.621380000	-1.509834000
H	1.450317000	1.393125000	-0.670591000
H	-0.036005000	1.847098000	-1.516818000
H	0.014401000	1.861417000	0.253061000
H	1.448998000	-0.901266000	0.655221000

H	-0.038123000	-1.859867000	0.625211000
H	0.013124000	-0.333651000	1.521457000

Compound 19

C	0.018172000	0.189893000	-0.345280000
C	1.284215000	1.023988000	-0.314289000
C	-1.223245000	1.044627000	-0.512380000
F	0.101378000	-0.629396000	-1.483457000
C	-0.083694000	-0.735155000	0.852032000
H	2.158824000	0.377410000	-0.257740000
H	1.280659000	1.683825000	0.552794000
H	1.355627000	1.631682000	-1.215237000
H	-2.106160000	0.412405000	-0.595411000
H	-1.345341000	1.705127000	0.345549000
H	-1.142112000	1.652574000	-1.412322000
H	0.795160000	-1.376089000	0.906661000
H	-0.151736000	-0.155685000	1.772289000
H	-0.969947000	-1.362404000	0.767292000

Compound 20

C	0.985627000	0.110602000	-0.125315000
C	0.189231000	-0.709466000	0.621909000
C	-1.164364000	-0.367956000	0.425051000
C	-1.157573000	0.679920000	-0.461196000
N	0.134727000	0.959598000	-0.785270000
F	0.627073000	-1.687919000	1.419303000
H	2.052748000	0.153862000	-0.234261000
H	-2.022367000	-0.832625000	0.876042000
H	-1.971851000	1.241895000	-0.881009000
H	0.426348000	1.682288000	-1.415956000

Geometries optimized with the pc-3 basis set (including molecules 21-25)

Compound 1

C	-0.003282000	-0.036234000	0.051275000
F	1.142507000	0.640166000	0.029836000
F	-1.006613000	0.837679000	0.029815000
F	-0.071674000	-0.780319000	-1.049674000
H	-0.060738000	-0.661292000	0.938647000

Compound 2

C	-0.032219000	-0.055972000	-0.000231000
F	0.656862000	1.137603000	-0.000025000
H	0.682912000	-0.875587000	0.000018000
H	-0.653320000	-0.102949000	0.891579000
H	-0.654236000	-0.103094000	-0.891442000

Compound 3

C	-1.070869000	0.235328000	-0.008991000
F	-1.687396000	-0.219599000	1.063686000
F	-1.683418000	-0.253832000	-1.092164000
F	0.174772000	-0.250370000	-0.022673000
O	-1.090194000	1.576926000	0.020078000
H	-0.643896000	1.920548000	-0.759936000

Compound 4

C	0.549955000	-0.316855000	-0.113428000
C	-0.744501000	0.440210000	-0.099490000
F	1.598904000	0.548304000	-0.090211000

F	0.653451000	-1.088370000	1.001643000
H	0.679569000	-0.968246000	-0.977622000
H	-1.577723000	-0.257615000	-0.094506000
H	-0.809673000	1.071953000	-0.981475000
H	-0.784884000	1.059320000	0.793589000

Compound 5

C	-0.779569000	0.024486000	-0.042436000
C	0.744572000	0.023420000	-0.040643000
F	1.220460000	-0.627723000	1.088631000
H	-1.183523000	1.032991000	-0.025831000
H	-1.183515000	-0.492967000	-0.908240000
H	1.153965000	-0.500612000	-0.902337000
H	-1.126496000	-0.491390000	0.849649000
H	1.154006000	1.031695000	-0.018792000

Compound 6

C	-0.234134000	-0.645486000	0.000000000
C	0.424218000	0.494806000	0.000000000
F	-1.564393000	-0.698673000	0.000000000
F	-0.194771000	1.673478000	0.000000000
H	0.258805000	-1.603860000	0.000000000
H	1.500674000	0.547036000	0.000000000

Compound 7

C	-0.222580000	-0.556104000	0.000000000
C	0.416546000	0.591881000	0.000000000
F	-1.559449000	-0.623889000	0.000000000
H	0.232510000	-1.535827000	0.000000000
H	1.493972000	0.594769000	0.000000000
H	-0.116699000	1.529970000	0.000000000

Compound 8

C	-0.316149000	-0.576710000	0.000000000
C	0.316149000	0.576710000	0.000000000
F	-1.652009000	-0.610497000	0.000000000
F	1.652009000	0.610497000	0.000000000
H	0.160981000	-1.544271000	0.000000000
H	-0.160981000	1.544271000	0.000000000

Compound 9

C	-0.239408000	-0.466826000	0.000000000
C	0.416258000	0.668831000	0.000000000
F	-1.548169000	-0.587999000	0.000000000
F	0.310163000	-1.660823000	0.000000000
H	1.491561000	0.659243000	0.000000000
H	-0.129704000	1.595274000	0.000000000

Compound 10

N	0.591504000	1.024484000	0.000000000
C	0.019444000	0.033677000	0.000000000
F	-0.610948000	-1.058162000	0.000000000

Compound 11

F	0.417786000	-0.190003000	0.000000000
H	-0.417786000	0.190003000	0.000000000

Compound 12

C	-0.554511000	0.059951000	0.000000000
O	0.604365000	0.184296000	0.000000000

F	-1.432240000	1.029277000	0.000000000
F	-1.206614000	-1.073522000	0.000000000

Compound 13

B	-0.451359000	0.132003000	-0.205113000
F	-0.668962000	1.365288000	0.171901000
F	0.638957000	-0.484233000	0.171853000
F	-1.322637000	-0.484060000	-0.961641000

Compound 14

Si	-0.969257000	0.233721000	0.034263000
H	-1.682946000	-0.271607000	1.216960000
F	-1.719737000	-0.297560000	-1.268014000
H	0.411935000	-0.271151000	0.008699000
H	-0.983996000	1.704596000	0.009092000

Compound 15

F	-0.571551000	1.482288000	0.002814000
P	-0.728651000	-0.051684000	0.275639000
F	0.767133000	-0.449847000	0.036123000
F	-1.270931000	-0.451757000	-1.138577000

Compound 16

C	0.122123000	0.166784000	-0.000208000
C	0.707234000	1.208294000	-0.000299000
C	-0.614942000	-1.092918000	-0.000129000
F	0.246502000	-2.169035000	-0.000336000
H	1.233481000	2.131092000	-0.000400000
H	-1.242881000	-1.163048000	0.887259000
H	-1.243217000	-1.162969000	-0.887287000

Compound 17

C	0.294855000	-0.510690000	0.000000000
C	-0.294855000	0.510690000	0.000000000
F	-0.934988000	1.619399000	0.000000000
F	0.934988000	-1.619399000	0.000000000

Compound 18

F	-1.487694000	-0.112941000	-0.631654000
C	-0.093249000	-0.099033000	-0.606196000
C	0.362673000	1.341219000	-0.639557000
C	0.361410000	-0.847535000	0.625199000
H	0.229604000	-0.620904000	-1.509013000
H	1.450023000	1.390323000	-0.667030000
H	-0.031148000	1.846234000	-1.518505000
H	0.012967000	1.862771000	0.250368000
H	1.448705000	-0.896761000	0.654590000
H	-0.033251000	-1.860907000	0.623614000
H	0.011661000	-0.336669000	1.521283000

Compound 19

C	0.018325000	0.190059000	-0.345452000
C	1.284160000	1.024230000	-0.314112000
C	-1.223095000	1.044587000	-0.512237000
F	0.101601000	-0.628146000	-1.483271000
C	-0.083685000	-0.735494000	0.851265000
H	2.158223000	0.378361000	-0.255719000
H	1.279714000	1.684693000	0.551635000
H	1.356954000	1.630444000	-1.215092000
H	-2.105790000	0.412991000	-0.592792000
H	-1.344092000	1.705941000	0.344341000
H	-1.143553000	1.650812000	-1.412638000
H	0.794098000	-1.376614000	0.906162000
H	-0.151469000	-0.156968000	1.771310000

H	-0.969591000	-1.362095000	0.767101000

Compound 20

C	0.985276000	0.110669000	-0.125347000
C	0.189330000	-0.709511000	0.621941000
C	-1.164226000	-0.367887000	0.424983000
C	-1.157272000	0.679918000	-0.461215000
N	0.134569000	0.959462000	-0.785144000
F	0.626693000	-1.686919000	1.418484000
H	2.051930000	0.153568000	-0.233957000
H	-2.021541000	-0.832504000	0.875884000
H	-1.971033000	1.241673000	-0.880877000
H	0.425874000	1.681731000	-1.415453000

Compound 21

C	-0.704680000	-0.682744000	0.000000000
C	-0.000000000	0.429006000	0.000000000
H	-1.780418000	-0.692714000	0.000000000
F	-0.095314000	-1.868359000	0.000000000
F	1.306611000	0.490752000	0.000000000
F	-0.543686000	1.623734000	0.000000000

Compound 22

C	0.000000000	0.000000000	0.502166000
F	0.000000000	1.095111000	-0.289730000
F	0.000000000	-1.095111000	-0.289730000
H	0.908722000	0.000000000	1.101074000
H	-0.908722000	0.000000000	1.101074000

Compound 23

C	0.000000000	0.000000000	1.161013000

N	0.000000000	0.000000000	2.302166000
C	0.000000000	0.000000000	-0.326234000
F	0.000000000	1.242679000	-0.782364000
F	1.076192000	-0.621340000	-0.782364000
F	-1.076192000	-0.621340000	-0.782364000

Compound 24

C	0.000000000	0.000000000	0.000000000
F	0.759743000	0.759743000	0.759743000
F	-0.759743000	-0.759743000	0.759743000
F	-0.759743000	0.759743000	-0.759743000
F	0.759743000	-0.759743000	-0.759743000

Compound 25

S	0.000506000	-0.133138000	-0.094037000
F	0.004642000	1.361777000	-0.426555000
F	-0.009989000	0.053567000	1.425957000
O	-1.235921000	-0.661518000	-0.476822000
O	1.240925000	-0.664467000	-0.459432000

Geometries optimized with the aug-pc-3 basis set

Compound 1

C	-0.003282000	-0.036234000	0.051275000
F	1.142507000	0.640166000	0.029836000
F	-1.006613000	0.837679000	0.029815000
F	-0.071674000	-0.780319000	-1.049674000
H	-0.060738000	-0.661292000	0.938647000

Compound 2

C	-0.032219000	-0.055972000	-0.000231000

F	0.656862000	1.137603000	-0.000025000
H	0.682912000	-0.875587000	0.000018000
H	-0.653320000	-0.102949000	0.891579000
H	-0.654236000	-0.103094000	-0.891442000

Compound 3

C	-1.070869000	0.235328000	-0.008991000
F	-1.687396000	-0.219599000	1.063686000
F	-1.683418000	-0.253832000	-1.092164000
F	0.174772000	-0.250370000	-0.022673000
O	-1.090194000	1.576926000	0.020078000
H	-0.643896000	1.920548000	-0.759936000

Compound 4

C	0.549955000	-0.316855000	-0.113428000
C	-0.744501000	0.440210000	-0.099490000
F	1.598904000	0.548304000	-0.090211000
F	0.653451000	-1.088370000	1.001643000
H	0.679569000	-0.968246000	-0.977622000
H	-1.577723000	-0.257615000	-0.094506000
H	-0.809673000	1.071953000	-0.981475000
H	-0.784884000	1.059320000	0.793589000

Compound 5

C	-0.779569000	0.024486000	-0.042436000
C	0.744572000	0.023420000	-0.040643000
F	1.220460000	-0.627723000	1.088631000
H	-1.183523000	1.032991000	-0.025831000
H	-1.183515000	-0.492967000	-0.908240000
H	1.153965000	-0.500612000	-0.902337000
H	-1.126496000	-0.491390000	0.849649000
H	1.154006000	1.031695000	-0.018792000

Compound 6

C	-0.234134000	-0.645486000	0.000000000
C	0.424218000	0.494806000	0.000000000
F	-1.564393000	-0.698673000	0.000000000
F	-0.194771000	1.673478000	0.000000000
H	0.258805000	-1.603860000	0.000000000
H	1.500674000	0.547036000	0.000000000

Compound 7

C	-0.222580000	-0.556104000	0.000000000
C	0.416546000	0.591881000	0.000000000
F	-1.559449000	-0.623889000	0.000000000
H	0.232510000	-1.535827000	0.000000000
H	1.493972000	0.594769000	0.000000000
H	-0.116699000	1.529970000	0.000000000

Compound 8

C	-0.316149000	-0.576710000	0.000000000
C	0.316149000	0.576710000	0.000000000
F	-1.652009000	-0.610497000	0.000000000
F	1.652009000	0.610497000	0.000000000
H	0.160981000	-1.544271000	0.000000000
H	-0.160981000	1.544271000	0.000000000

Compound 9

C	-0.239408000	-0.466826000	0.000000000
C	0.416258000	0.668831000	0.000000000
F	-1.548169000	-0.587999000	0.000000000
F	0.310163000	-1.660823000	0.000000000
H	1.491561000	0.659243000	0.000000000

H	-0.129704000	1.595274000	0.000000000

Compound 10

N	0.591504000	1.024484000	0.000000000
C	0.019444000	0.033677000	0.000000000
F	-0.610948000	-1.058162000	0.000000000

Compound 11

F	0.417786000	-0.190003000	0.000000000
H	-0.417786000	0.190003000	0.000000000

Compound 12

C	-0.554511000	0.059951000	0.000000000
O	0.604365000	0.184296000	0.000000000
F	-1.432240000	1.029277000	0.000000000
F	-1.206614000	-1.073522000	0.000000000

Compound 13

B	-0.451359000	0.132003000	-0.205113000
F	-0.668963000	1.365295000	0.171904000
F	0.638963000	-0.484236000	0.171855000
F	-1.322642000	-0.484063000	-0.961645000

Compound 14

Si	-0.969203000	0.233764000	0.034360000
H	-1.682984000	-0.271643000	1.216941000
F	-1.719735000	-0.297558000	-1.268006000
H	0.411939000	-0.271178000	0.008660000
H	-0.984016000	1.704615000	0.009045000

Compound 15

F	-0.571551000	1.482288000	0.002814000
P	-0.728651000	-0.051684000	0.275639000
F	0.767133000	-0.449847000	0.036123000
F	-1.270931000	-0.451757000	-1.138577000

Compound 16

C	0.122071000	0.167366000	-0.000252000
C	0.707103000	1.208955000	-0.000317000
C	-0.615166000	-1.092399000	-0.000129000
F	0.246352000	-2.168774000	-0.000334000
H	1.234443000	2.131143000	-0.000358000
H	-1.243076000	-1.164064000	0.887536000
H	-1.243427000	-1.164026000	-0.887547000

Compound 17

C	0.294855000	-0.510690000	0.000000000
C	-0.294855000	0.510690000	0.000000000
F	-0.934988000	1.619399000	0.000000000
F	0.934988000	-1.619399000	0.000000000

Compound 18

F	-1.486292000	-0.113761000	-0.633069000
C	-0.091678000	-0.099213000	-0.606506000
C	0.362447000	1.341245000	-0.639463000
C	0.361189000	-0.847467000	0.625266000
H	0.232250000	-0.621017000	-1.509207000
H	1.449701000	1.393213000	-0.669963000
H	-0.034566000	1.846602000	-1.517031000
H	0.014114000	1.861783000	0.251693000

H	1.448382000	-0.900767000	0.655609000
H	-0.036685000	-1.859804000	0.624677000
H	0.012839000	-0.335018000	1.521092000

Compound 19

C	0.018545000	0.190346000	-0.345191000
C	1.284683000	1.023719000	-0.315288000
C	-1.222825000	1.044565000	-0.511494000
F	0.100981000	-0.628557000	-1.482768000
C	-0.083819000	-0.734560000	0.851674000
H	2.158340000	0.377083000	-0.257389000
H	1.282040000	1.684899000	0.550031000
H	1.356770000	1.629431000	-1.216770000
H	-2.104598000	0.412127000	-0.596338000
H	-1.346942000	1.702640000	0.347308000
H	-1.142621000	1.654535000	-1.409437000
H	0.794944000	-1.374276000	0.909273000
H	-0.155349000	-0.156236000	1.771693000
H	-0.968350000	-1.362915000	0.765197000

Compound 20

C	0.985294000	0.110671000	-0.125350000
C	0.189334000	-0.709524000	0.621951000
C	-1.164247000	-0.367902000	0.424997000
C	-1.157292000	0.679914000	-0.461210000
N	0.134577000	0.959444000	-0.785130000
F	0.626698000	-1.686924000	1.418488000
H	2.051954000	0.153597000	-0.233982000
H	-2.021573000	-0.832519000	0.875899000
H	-1.971019000	1.241707000	-0.880907000
H	0.425874000	1.681735000	-1.415457000

Geometries optimized with the pc-4 basis set

Compound 1

C	-0.003288000	-0.036217000	0.051291000
F	1.142538000	0.640165000	0.029822000
F	-1.006649000	0.837701000	0.029827000
F	-0.071655000	-0.780343000	-1.049707000
H	-0.060746000	-0.661306000	0.938667000

Compound 2

C	-0.032219000	-0.055972000	-0.000231000
F	0.656862000	1.137603000	-0.000025000
H	0.682912000	-0.875587000	0.000018000
H	-0.653320000	-0.102949000	0.891579000
H	-0.654236000	-0.103094000	-0.891442000

Compound 3

C	-1.070869000	0.235328000	-0.008991000
F	-1.687396000	-0.219599000	1.063686000
F	-1.683418000	-0.253832000	-1.092164000
F	0.174772000	-0.250370000	-0.022673000
O	-1.090194000	1.576926000	0.020078000
H	-0.643896000	1.920548000	-0.759936000

Compound 4

C	0.549955000	-0.316855000	-0.113428000
C	-0.744501000	0.440210000	-0.099490000
F	1.598904000	0.548304000	-0.090211000
F	0.653451000	-1.088370000	1.001643000
H	0.679569000	-0.968246000	-0.977622000

H	-1.577723000	-0.257615000	-0.094506000
H	-0.809673000	1.071953000	-0.981475000
H	-0.784884000	1.059320000	0.793589000

Compound 5

C	-0.778905000	0.024251000	-0.042029000
C	0.744410000	0.023372000	-0.040594000
F	1.220202000	-0.627516000	1.088963000
H	-1.183014000	1.032984000	-0.025550000
H	-1.183011000	-0.492621000	-0.908439000
H	1.153698000	-0.500704000	-0.902634000
H	-1.127245000	-0.491833000	0.849730000
H	1.153766000	1.031968000	-0.019447000

Compound 6

C	-0.234134000	-0.645486000	0.000000000
C	0.424218000	0.494806000	0.000000000
F	-1.564393000	-0.698673000	0.000000000
F	-0.194771000	1.673478000	0.000000000
H	0.258805000	-1.603860000	0.000000000
H	1.500674000	0.547036000	0.000000000

Compound 7

C	-0.222580000	-0.556104000	0.000000000
C	0.416546000	0.591881000	0.000000000
F	-1.559449000	-0.623889000	0.000000000
H	0.232510000	-1.535827000	0.000000000
H	1.493972000	0.594769000	0.000000000
H	-0.116699000	1.529970000	0.000000000

Compound 8

C	-0.316149000	-0.576710000	0.000000000
C	0.316149000	0.576710000	0.000000000
F	-1.652009000	-0.610497000	0.000000000
F	1.652009000	0.610497000	0.000000000
H	0.160981000	-1.544271000	0.000000000
H	-0.160981000	1.544271000	0.000000000

Compound 9

C	-0.239408000	-0.466826000	0.000000000
C	0.416258000	0.668831000	0.000000000
F	-1.548169000	-0.587999000	0.000000000
F	0.310163000	-1.660823000	0.000000000
H	1.491561000	0.659243000	0.000000000
H	-0.129704000	1.595274000	0.000000000

Compound 10

N	0.591504000	1.024484000	0.000000000
C	0.019444000	0.033677000	0.000000000
F	-0.610948000	-1.058162000	0.000000000

Compound 11

F	0.417786000	-0.190003000	0.000000000
H	-0.417786000	0.190003000	0.000000000

Compound 12

C	-0.554511000	0.059951000	0.000000000
O	0.604365000	0.184296000	0.000000000
F	-1.432240000	1.029277000	0.000000000
F	-1.206614000	-1.073522000	0.000000000

Compound 13

B	-0.451362000	0.132008000	-0.205114000
F	-0.668978000	1.365386000	0.171933000
F	0.639020000	-0.484283000	0.171869000
F	-1.322681000	-0.484112000	-0.961687000

Compound 14

Si	-0.969152000	0.233784000	0.034434000
H	-1.682833000	-0.271528000	1.216769000
F	-1.719697000	-0.297546000	-1.267977000
H	0.411714000	-0.271118000	0.008685000
H	-0.984032000	1.704407000	0.009089000

Compound 15

F	-0.571551000	1.482288000	0.002814000
P	-0.728651000	-0.051684000	0.275639000
F	0.767133000	-0.449847000	0.036123000
F	-1.270931000	-0.451757000	-1.138577000

Compound 16

C	0.121788000	0.167493000	-0.000253000
C	0.707161000	1.208964000	-0.000318000
C	-0.615093000	-1.092501000	-0.000129000
F	0.246442000	-2.168960000	-0.000334000
H	1.234545000	2.131207000	-0.000356000
H	-1.243095000	-1.164020000	0.887313000
H	-1.243447000	-1.163983000	-0.887323000

Compound 17

C	0.294855000	-0.510690000	0.000000000
C	-0.294855000	0.510690000	0.000000000
F	-0.934988000	1.619399000	0.000000000
F	0.934988000	-1.619399000	0.000000000

Compound 18

F	-1.486251000	-0.114080000	-0.633622000
C	-0.091555000	-0.099339000	-0.606725000
C	0.362378000	1.341016000	-0.639367000
C	0.361120000	-0.847270000	0.625115000
H	0.232719000	-0.621056000	-1.509275000
H	1.449523000	1.393251000	-0.670241000
H	-0.034993000	1.846858000	-1.516376000
H	0.014472000	1.861145000	0.252112000
H	1.448204000	-0.901027000	0.655503000
H	-0.037113000	-1.859364000	0.625226000
H	0.013197000	-0.334337000	1.520748000

Compound 19

C	0.018445000	0.190028000	-0.345226000
C	1.283821000	1.024054000	-0.314159000
C	-1.222510000	1.044455000	-0.511918000
F	0.101633000	-0.628330000	-1.483211000
C	-0.083797000	-0.735083000	0.851242000
H	2.157893000	0.378058000	-0.256749000
H	1.280617000	1.684747000	0.551509000
H	1.356098000	1.630552000	-1.215042000
H	-2.105140000	0.412772000	-0.593009000
H	-1.344491000	1.706034000	0.344475000
H	-1.143016000	1.650943000	-1.412212000

H	0.794204000	-1.375914000	0.906743000
H	-0.152682000	-0.157199000	1.771691000
H	-0.969275000	-1.362316000	0.766367000

Compound 20

C	0.985251000	0.110674000	-0.125349000
C	0.189285000	-0.709505000	0.621938000
C	-1.164173000	-0.367916000	0.425004000
C	-1.157168000	0.679903000	-0.461209000
N	0.134566000	0.959395000	-0.785087000
F	0.626703000	-1.686935000	1.418497000
H	2.051879000	0.153666000	-0.234035000
H	-2.021598000	-0.832422000	0.875818000
H	-1.970888000	1.241667000	-0.880881000
H	0.425743000	1.681673000	-1.415396000

Geometries optimized with the aug-pc-4 basis set

Compound 1

C	-0.003282000	-0.036234000	0.051275000
F	1.142507000	0.640166000	0.029836000
F	-1.006613000	0.837679000	0.029815000
F	-0.071674000	-0.780319000	-1.049674000
H	-0.060738000	-0.661292000	0.938647000

Compound 2

C	-0.032219000	-0.055972000	-0.000231000
F	0.656862000	1.137603000	-0.000025000
H	0.682912000	-0.875587000	0.000018000
H	-0.653320000	-0.102949000	0.891579000
H	-0.654236000	-0.103094000	-0.891442000

Compound 3

C	-1.070869000	0.235328000	-0.008991000
F	-1.687396000	-0.219599000	1.063686000
F	-1.683418000	-0.253832000	-1.092164000
F	0.174772000	-0.250370000	-0.022673000
O	-1.090194000	1.576926000	0.020078000
H	-0.643896000	1.920548000	-0.759936000

Compound 4

C	0.549955000	-0.316855000	-0.113428000
C	-0.744501000	0.440210000	-0.099490000
F	1.598904000	0.548304000	-0.090211000
F	0.653451000	-1.088370000	1.001643000
H	0.679569000	-0.968246000	-0.977622000
H	-1.577723000	-0.257615000	-0.094506000
H	-0.809673000	1.071953000	-0.981475000
H	-0.784884000	1.059320000	0.793589000

Compound 5

C	-0.779569000	0.024486000	-0.042436000
C	0.744572000	0.023420000	-0.040643000
F	1.220460000	-0.627723000	1.088631000
H	-1.183523000	1.032991000	-0.025831000
H	-1.183515000	-0.492967000	-0.908240000
H	1.153965000	-0.500612000	-0.902337000
H	-1.126496000	-0.491390000	0.849649000
H	1.154006000	1.031695000	-0.018792000

Compound 6

C	-0.234134000	-0.645486000	0.000000000

C	0.424218000	0.494806000	0.000000000
F	-1.564393000	-0.698673000	0.000000000
F	-0.194771000	1.673478000	0.000000000
H	0.258805000	-1.603860000	0.000000000
H	1.500674000	0.547036000	0.000000000

Compound 7

C	-0.222580000	-0.556104000	0.000000000
C	0.416546000	0.591881000	0.000000000
F	-1.559449000	-0.623889000	0.000000000
H	0.232510000	-1.535827000	0.000000000
H	1.493972000	0.594769000	0.000000000
H	-0.116699000	1.529970000	0.000000000

Compound 8

C	-0.316149000	-0.576710000	0.000000000
C	0.316149000	0.576710000	0.000000000
F	-1.652009000	-0.610497000	0.000000000
F	1.652009000	0.610497000	0.000000000
H	0.160981000	-1.544271000	0.000000000
H	-0.160981000	1.544271000	0.000000000

Compound 9

C	-0.239408000	-0.466826000	0.000000000
C	0.416258000	0.668831000	0.000000000
F	-1.548169000	-0.587999000	0.000000000
F	0.310163000	-1.660823000	0.000000000
H	1.491561000	0.659243000	0.000000000
H	-0.129704000	1.595274000	0.000000000

Compound 10

N	0.591504000	1.024484000	0.000000000
C	0.019444000	0.033677000	0.000000000
F	-0.610948000	-1.058162000	0.000000000

Compound 11

F	0.417786000	-0.190003000	0.000000000
H	-0.417786000	0.190003000	0.000000000

Compound 12

C	-0.554511000	0.059951000	0.000000000
O	0.604365000	0.184296000	0.000000000
F	-1.432240000	1.029277000	0.000000000
F	-1.206614000	-1.073522000	0.000000000

Compound 13

B	-0.451362000	0.132010000	-0.205113000
F	-0.668980000	1.365397000	0.171936000
F	0.639027000	-0.484289000	0.171871000
F	-1.322687000	-0.484118000	-0.961693000

Compound 14

Si	-0.969146000	0.233786000	0.034444000
H	-1.682820000	-0.271516000	1.216757000
F	-1.719693000	-0.297543000	-1.267979000
H	0.411693000	-0.271112000	0.008686000
H	-0.984034000	1.704385000	0.009092000

Compound 15

F	-0.572025000	1.481855000	0.002283000
P	-0.728496000	-0.051775000	0.275928000
F	0.766719000	-0.450093000	0.035832000
F	-1.270199000	-0.450986000	-1.138044000

Compound 16

C	0.122172000	0.166714000	-0.000249000
C	0.707271000	1.208283000	-0.000316000
C	-0.614917000	-1.092940000	-0.000128000
F	0.246459000	-2.169266000	-0.000334000
H	1.233384000	2.131239000	-0.000361000
H	-1.242859000	-1.162938000	0.887167000
H	-1.243209000	-1.162893000	-0.887179000

Compound 17

C	0.294855000	-0.510690000	0.000000000
C	-0.294855000	0.510690000	0.000000000
F	-0.934988000	1.619399000	0.000000000
F	0.934988000	-1.619399000	0.000000000

Compound 18

F	-1.487907000	-0.112977000	-0.631712000
C	-0.093181000	-0.099038000	-0.606204000
C	0.362539000	1.340969000	-0.639491000
C	0.361283000	-0.847353000	0.625012000
H	0.230178000	-0.620809000	-1.508849000
H	1.449849000	1.390404000	-0.667154000
H	-0.031314000	1.846046000	-1.518332000
H	0.013212000	1.862673000	0.250461000
H	1.448535000	-0.896940000	0.654571000

H	-0.033439000	-1.860653000	0.623550000
H	0.011947000	-0.336525000	1.521247000

Compound 19

C	0.018345000	0.190096000	-0.345258000
C	1.283988000	1.024222000	-0.313996000
C	-1.222901000	1.044377000	-0.512341000
F	0.101832000	-0.628628000	-1.483414000
C	-0.083766000	-0.735226000	0.851367000
H	2.158062000	0.378335000	-0.256816000
H	1.280490000	1.684046000	0.552212000
H	1.356152000	1.631251000	-1.214410000
H	-2.105582000	0.412860000	-0.592983000
H	-1.344359000	1.706023000	0.343925000
H	-1.143261000	1.650521000	-1.412720000
H	0.793884000	-1.376457000	0.906059000
H	-0.151300000	-0.157018000	1.771590000
H	-0.969786000	-1.361601000	0.767285000

Compound 20

C	0.985550000	0.111420000	-0.126006000
C	0.188348000	-0.708762000	0.621375000
C	-1.165728000	-0.369337000	0.426310000
C	-1.156717000	0.680069000	-0.461377000
N	0.134521000	0.958471000	-0.784301000
F	0.629379000	-1.690120000	1.421011000
H	2.052345000	0.155898000	-0.235955000
H	-2.024917000	-0.832569000	0.876164000
H	-1.969140000	1.243115000	-0.882224000
H	0.425958000	1.682014000	-1.415698000

Geometries optimized with the 6-31G(d,p) basis set

Compound 1

C	-0.003312000	-0.035945000	0.050986000
F	1.144602000	0.641960000	0.029296000
F	-1.008387000	0.839934000	0.029267000
F	-0.071749000	-0.781609000	-1.052351000
H	-0.060954000	-0.664340000	0.942701000

Compound 2

C	-0.027886000	-0.048189000	-0.000034000
F	0.659779000	1.142151000	-0.000294000
H	0.682223000	-0.880409000	-0.000056000
H	-0.656783000	-0.106854000	0.893197000
H	-0.657333000	-0.106700000	-0.892913000

Compound 3

C	-1.071083000	0.234987000	-0.008508000
F	-1.689499000	-0.220774000	1.067284000
F	-1.684265000	-0.252288000	-1.095566000
F	0.178127000	-0.249132000	-0.023432000
O	-1.091522000	1.578953000	0.022831000
H	-0.642760000	1.917255000	-0.762609000

Compound 4

C	0.555750000	-0.319654000	-0.112607000
C	-0.743141000	0.438044000	-0.101560000
F	1.597570000	0.554901000	-0.090180000

F	0.648344000	-1.088303000	1.006027000
H	0.682509000	-0.973404000	-0.982809000
H	-1.583590000	-0.257913000	-0.093060000
H	-0.812521000	1.076884000	-0.983519000
H	-0.779822000	1.058145000	0.796208000

Compound 5

C	-0.770985000	0.010109000	-0.032475000
C	0.741285000	-0.006267000	-0.043026000
F	1.210356000	-0.071666000	1.255924000
H	-1.136378000	0.875848000	0.524074000
H	-1.160213000	0.061526000	-1.052455000
H	1.126080000	-0.875326000	-0.588195000
H	-1.156232000	-0.894813000	0.442375000
H	1.145988000	0.900490000	-0.506222000

Compound 6

C	-0.233023000	-0.649496000	0.000000000
C	0.428246000	0.495678000	0.000000000
F	-1.568080000	-0.692762000	0.000000000
F	-0.201761000	1.673911000	0.000000000
H	0.256001000	-1.615172000	0.000000000
H	1.509016000	0.555142000	0.000000000

Compound 7

C	-0.226276000	-0.558691000	0.000000000
C	0.419286000	0.593153000	0.000000000
F	-1.564751000	-0.625143000	0.000000000
H	0.232805000	-1.542179000	0.000000000
H	1.500920000	0.600117000	0.000000000
H	-0.117684000	1.533542000	0.000000000

Compound 8

C	-0.318353000	-0.579179000	0.000000000
C	0.318353000	0.579179000	0.000000000
F	-1.657243000	-0.611037000	0.000000000
F	1.657243000	0.611037000	0.000000000
H	0.163316000	-1.549319000	0.000000000
H	-0.163316000	1.549319000	0.000000000

Compound 9

C	-0.241491000	-0.470473000	0.000000000
C	0.417311000	0.670652000	0.000000000
F	-1.554812000	-0.588738000	0.000000000
F	0.312826000	-1.666950000	0.000000000
H	1.496424000	0.662238000	0.000000000
H	-0.129556000	1.600971000	0.000000000

Compound 10

N	0.596502000	1.033140000	0.000000000
C	0.019107000	0.033092000	0.000000000
F	-0.615608000	-1.066234000	0.000000000

Compound 11

F	0.419194000	-0.190643000	0.000000000
H	-0.419194000	0.190643000	0.000000000

Compound 12

C	-0.555364000	0.059859000	0.000000000
O	0.612528000	0.185172000	0.000000000
F	-1.436293000	1.032599000	0.000000000
F	-1.209871000	-1.077628000	0.000000000

Compound 13

B	-0.450900000	0.131747000	-0.205932000
F	-0.670014000	1.370139000	0.173603000
F	0.643871000	-0.486583000	0.174058000
F	-1.326958000	-0.486305000	-0.964729000

Compound 14

Si	-0.973387000	0.230656000	0.027409000
H	-1.679203000	-0.268662000	1.225888000
F	-1.729670000	-0.303769000	-1.285145000
H	0.418151000	-0.268403000	0.016467000
H	-0.979891000	1.708178000	0.016381000

Compound 15

F	-0.572292000	1.496071000	0.004007000
P	-0.732554000	-0.054739000	0.282611000
F	0.779822000	-0.455220000	0.038050000
F	-1.278976000	-0.457112000	-1.148669000

Compound 16

C	0.117489000	0.168143000	-0.000217000
C	0.713243000	1.213935000	-0.000306000
C	-0.615502000	-1.100023000	-0.000125000
F	0.252647000	-2.169939000	-0.000343000
H	1.242138000	2.140387000	-0.000392000
H	-1.250692000	-1.167186000	0.889459000
H	-1.251023000	-1.167117000	-0.889477000

Compound 17

C	0.297252000	-0.514840000	0.000000000
C	-0.297252000	0.514840000	0.000000000
F	-0.941582000	1.630820000	0.000000000
F	0.941582000	-1.630820000	0.000000000

Compound 18

F	-1.485258000	-0.110600000	-0.627597000
C	-0.094291000	-0.100728000	-0.609130000
C	0.363402000	1.343561000	-0.641925000
C	0.362142000	-0.850757000	0.626043000
H	0.240176000	-0.623969000	-1.514309000
H	1.454371000	1.402538000	-0.674717000
H	-0.041480000	1.850520000	-1.520361000
H	0.012238000	1.864730000	0.253052000
H	1.453043000	-0.909552000	0.661313000
H	-0.043608000	-1.864638000	0.626407000
H	0.010967000	-0.335308000	1.524323000

Compound 19

C	0.018489000	0.189599000	-0.345935000
C	1.287505000	1.026866000	-0.313668000
C	-1.226387000	1.046820000	-0.512567000
F	0.101903000	-0.626047000	-1.480395000
C	-0.083936000	-0.738070000	0.854510000
H	2.164418000	0.377052000	-0.256912000
H	1.288876000	1.693895000	0.552591000
H	1.360972000	1.631240000	-1.221287000
H	-2.111418000	0.411053000	-0.596486000
H	-1.354223000	1.713596000	0.344416000
H	-1.146360000	1.652644000	-1.418670000
H	0.795807000	-1.384584000	0.904887000
H	-0.150538000	-0.163910000	1.782510000

H	-0.973307000	-1.367353000	0.767506000

Compound 20

C	0.988300000	0.110917000	-0.125764000
C	0.190008000	-0.712774000	0.624654000
C	-1.167173000	-0.369229000	0.426316000
C	-1.160255000	0.681583000	-0.462419000
N	0.135549000	0.961484000	-0.786919000
F	0.630203000	-1.692463000	1.422935000
H	2.058915000	0.154152000	-0.234927000
H	-2.027602000	-0.835909000	0.879171000
H	-1.976883000	1.246152000	-0.884265000
H	0.428537000	1.686285000	-1.419482000

Geometries optimized with the 6-311G(d,p) basis set

Compound 1

C	-0.003311000	-0.035999000	0.051198000
F	1.143796000	0.640939000	0.029971000
F	-1.007668000	0.838579000	0.029752000
F	-0.071884000	-0.781275000	-1.050741000
H	-0.060732000	-0.662243000	0.939721000

Compound 2

C	-0.031026000	-0.053642000	0.000132000
F	0.658653000	1.140001000	-0.000310000
H	0.683947000	-0.877917000	-0.000326000
H	-0.655668000	-0.104188000	0.893492000
H	-0.655905000	-0.104255000	-0.893088000

Compound 3

C	-1.071155000	0.237543000	-0.008551000
F	-1.687960000	-0.220382000	1.064711000
F	-1.683740000	-0.251218000	-1.094056000
F	0.176547000	-0.247707000	-0.023375000
O	-1.091652000	1.577616000	0.022545000
H	-0.643040000	1.913148000	-0.761273000

Compound 4

C	0.551671000	-0.318594000	-0.114551000
C	-0.744261000	0.438833000	-0.101347000
F	1.597418000	0.552884000	-0.090376000
F	0.649474000	-1.088101000	1.004350000
H	0.684350000	-0.972200000	-0.979411000
H	-1.582402000	-0.257863000	-0.093019000
H	-0.811792000	1.076136000	-0.982947000
H	-0.779361000	1.057607000	0.795800000

Compound 5

C	-0.771203000	0.010918000	-0.036841000
C	0.739370000	-0.006961000	-0.045226000
F	1.205095000	-0.070284000	1.259660000
H	-1.135579000	0.876649000	0.517719000
H	-1.157256000	0.061987000	-1.056843000
H	1.127500000	-0.878774000	-0.577261000
H	-1.157008000	-0.892500000	0.437223000
H	1.148981000	0.898863000	-0.498431000

Compound 6

C	-0.233580000	-0.647312000	0.000000000

C	0.426127000	0.495149000	0.000000000
F	-1.566842000	-0.696554000	0.000000000
F	-0.197998000	1.674660000	0.000000000
H	0.257436000	-1.609737000	0.000000000
H	1.505256000	0.551096000	0.000000000

Compound 7

C	-0.224132000	-0.557334000	0.000000000
C	0.419126000	0.591982000	0.000000000
F	-1.562691000	-0.623084000	0.000000000
H	0.231947000	-1.539904000	0.000000000
H	1.499453000	0.598993000	0.000000000
H	-0.119403000	1.530148000	0.000000000

Compound 8

C	-0.317622000	-0.577487000	0.000000000
C	0.317622000	0.577487000	0.000000000
F	-1.655665000	-0.610116000	0.000000000
F	1.655665000	0.610116000	0.000000000
H	0.163392000	-1.545861000	0.000000000
H	-0.163392000	1.545861000	0.000000000

Compound 9

C	-0.240371000	-0.468520000	0.000000000
C	0.416631000	0.669474000	0.000000000
F	-1.551133000	-0.588091000	0.000000000
F	0.311552000	-1.663437000	0.000000000
H	1.494520000	0.660044000	0.000000000
H	-0.130499000	1.598230000	0.000000000

Compound 10

N	0.592449000	1.026121000	0.000000000
C	0.019288000	0.033406000	0.000000000
F	-0.611737000	-1.059528000	0.000000000

Compound 11

F	0.417009000	-0.189649000	0.000000000
H	-0.417009000	0.189649000	0.000000000

Compound 12

C	-0.553485000	0.060061000	0.000000000
O	0.605625000	0.184431000	0.000000000
F	-1.433508000	1.030318000	0.000000000
F	-1.207632000	-1.074809000	0.000000000

Compound 13

B	-0.450917000	0.131842000	-0.205894000
F	-0.669709000	1.368337000	0.173039000
F	0.642218000	-0.485743000	0.173443000
F	-1.325592000	-0.485438000	-0.963589000

Compound 14

Si	-0.969310000	0.233521000	0.034404000
H	-1.681175000	-0.269963000	1.222938000
F	-1.728374000	-0.303123000	-1.283013000
H	0.416504000	-0.269785000	0.013217000
H	-0.981644000	1.707350000	0.013454000

Compound 15

F	-0.572303000	1.496597000	0.003481000
P	-0.733812000	-0.055018000	0.284616000
F	0.779261000	-0.455584000	0.036494000
F	-1.277146000	-0.456996000	-1.148592000

Compound 16

C	0.113299000	0.170815000	-0.000242000
C	0.710230000	1.209166000	-0.000314000
C	-0.617110000	-1.095445000	-0.000136000
F	0.252840000	-2.166247000	-0.000321000
H	1.240320000	2.131732000	-0.000368000
H	-1.245457000	-1.165945000	0.890335000
H	-1.245823000	-1.165876000	-0.890354000

Compound 17

C	0.295436000	-0.511696000	0.000000000
C	-0.295436000	0.511696000	0.000000000
F	-0.936475000	1.621975000	0.000000000
F	0.936475000	-1.621975000	0.000000000

Compound 18

F	-1.484613000	-0.110996000	-0.628282000
C	-0.088906000	-0.100088000	-0.608017000
C	0.364287000	1.343780000	-0.641390000
C	0.363026000	-0.850404000	0.626500000
H	0.235233000	-0.623174000	-1.512938000
H	1.453960000	1.404029000	-0.674924000
H	-0.041883000	1.847435000	-1.519406000
H	0.011622000	1.863285000	0.252200000
H	1.452630000	-0.910474000	0.662502000

H	-0.044006000	-1.862271000	0.624210000
H	0.010349000	-0.335325000	1.522644000

Compound 19

C	0.017824000	0.191881000	-0.342645000
C	1.286697000	1.026289000	-0.313376000
C	-1.225869000	1.047052000	-0.511956000
F	0.100958000	-0.627063000	-1.480936000
C	-0.084030000	-0.736935000	0.854777000
H	2.161540000	0.375815000	-0.259125000
H	1.289291000	1.691269000	0.553032000
H	1.358150000	1.630399000	-1.219576000
H	-2.108290000	0.410291000	-0.596885000
H	-1.353807000	1.713199000	0.344021000
H	-1.143646000	1.650774000	-1.417478000
H	0.796253000	-1.380431000	0.903972000
H	-0.152221000	-0.163179000	1.781614000
H	-0.971050000	-1.366559000	0.765062000

Compound 20

C	0.987250000	0.110638000	-0.125457000
C	0.189697000	-0.711526000	0.623620000
C	-1.165976000	-0.368542000	0.425654000
C	-1.159957000	0.681611000	-0.462464000
N	0.135106000	0.961254000	-0.786695000
F	0.629328000	-1.690660000	1.421470000
H	2.056660000	0.152684000	-0.233532000
H	-2.024676000	-0.835128000	0.878312000
H	-1.976292000	1.244402000	-0.882826000
H	0.428457000	1.685465000	-1.418783000

Geometries optimized with the 6-31++G(d,p) basis set

Compound 1

C	-0.003402000	-0.037265000	0.053288000
F	1.147603000	0.643538000	0.029104000
F	-1.010982000	0.841878000	0.028810000
F	-0.072075000	-0.783027000	-1.054766000
H	-0.060945000	-0.665124000	0.943463000

Compound 2

C	-0.034098000	-0.059110000	-0.000028000
F	0.658758000	1.140798000	-0.000183000
H	0.688097000	-0.878565000	-0.000198000
H	-0.656125000	-0.101501000	0.896746000
H	-0.656632000	-0.101623000	-0.896438000

Compound 3

C	-1.071326000	0.236742000	-0.008270000
F	-1.690816000	-0.221939000	1.069703000
F	-1.685791000	-0.257242000	-1.097037000
F	0.180173000	-0.253702000	-0.023087000
O	-1.090766000	1.579014000	0.020966000
H	-0.642474000	1.926127000	-0.762275000

Compound 4

C	0.550541000	-0.319189000	-0.116421000
C	-0.746163000	0.440885000	-0.099918000
F	1.602763000	0.552743000	-0.090491000

F	0.652855000	-1.091641000	1.006504000
H	0.687201000	-0.975100000	-0.981288000
H	-1.584662000	-0.258289000	-0.093297000
H	-0.813049000	1.077448000	-0.984385000
H	-0.784389000	1.061843000	0.797797000

Compound 5

C	-0.781082000	0.024588000	-0.042605000
C	0.745556000	0.025082000	-0.043620000
F	1.222291000	-0.629100000	1.092793000
H	-1.188862000	1.037436000	-0.024693000
H	-1.188867000	-0.494662000	-0.912409000
H	1.159693000	-0.505388000	-0.905419000
H	-1.128642000	-0.494734000	0.853665000
H	1.159812000	1.036678000	-0.017711000

Compound 6

C	-0.230876000	-0.651617000	0.000000000
C	0.431168000	0.494902000	0.000000000
F	-1.569102000	-0.691997000	0.000000000
F	-0.202966000	1.674434000	0.000000000
H	0.251107000	-1.620362000	0.000000000
H	1.511069000	0.561941000	0.000000000

Compound 7

C	-0.220368000	-0.560954000	0.000000000
C	0.419928000	0.595412000	0.000000000
F	-1.566088000	-0.623511000	0.000000000
H	0.228542000	-1.548214000	0.000000000
H	1.502163000	0.603141000	0.000000000
H	-0.119878000	1.534927000	0.000000000

Compound 8

C	-0.314960000	-0.581725000	0.000000000
C	0.314960000	0.581725000	0.000000000
F	-1.658544000	-0.613231000	0.000000000
F	1.658544000	0.613231000	0.000000000
H	0.160291000	-1.555067000	0.000000000
H	-0.160291000	1.555067000	0.000000000

Compound 9

C	-0.240785000	-0.469250000	0.000000000
C	0.418349000	0.672451000	0.000000000
F	-1.557671000	-0.591568000	0.000000000
F	0.311804000	-1.670840000	0.000000000
H	1.498258000	0.663645000	0.000000000
H	-0.129255000	1.603263000	0.000000000

Compound 10

N	0.596743000	1.033557000	0.000000000
C	0.019257000	0.033353000	0.000000000
F	-0.616000000	-1.066912000	0.000000000

Compound 11

F	0.420091000	-0.191051000	0.000000000
H	-0.420091000	0.191051000	0.000000000

Compound 12

C	-0.553435000	0.060066000	0.000000000
O	0.613655000	0.185292000	0.000000000
F	-1.437977000	1.033882000	0.000000000

F	-1.211243000	-1.079240000	0.000000000

Compound 13

B	-0.450938000	0.132048000	-0.205498000
F	-0.670785000	1.373012000	0.174220000
F	0.646080000	-0.488413000	0.174476000
F	-1.328358000	-0.487648000	-0.966198000

Compound 14

Si	-0.965808000	0.235566000	0.040236000
H	-1.684910000	-0.272501000	1.224011000
F	-1.728695000	-0.303583000	-1.284172000
H	0.418840000	-0.272570000	0.010204000
H	-0.983427000	1.711087000	0.010721000

Compound 15

F	-0.573279000	1.502740000	0.003666000
P	-0.735010000	-0.056466000	0.287667000
F	0.786239000	-0.458463000	0.038266000
F	-1.281950000	-0.458812000	-1.153599000

Compound 16

C	0.120614000	0.167435000	-0.000264000
C	0.712009000	1.217750000	-0.000322000
C	-0.621005000	-1.094554000	-0.000128000
F	0.248654000	-2.174201000	-0.000333000
H	1.243943000	2.143352000	-0.000348000
H	-1.247778000	-1.170806000	0.892868000
H	-1.248137000	-1.170776000	-0.892873000

Compound 17

C	0.297405000	-0.515105000	0.000000000
C	-0.297405000	0.515105000	0.000000000
F	-0.942353000	1.632155000	0.000000000
F	0.942353000	-1.632155000	0.000000000

Compound 18

F	-1.489943000	-0.114055000	-0.633581000
C	-0.088168000	-0.099810000	-0.607536000
C	0.363010000	1.345746000	-0.641302000
C	0.361748000	-0.851309000	0.628247000
H	0.233619000	-0.624413000	-1.515084000
H	1.454965000	1.401589000	-0.674538000
H	-0.039830000	1.851567000	-1.521828000
H	0.012947000	1.868320000	0.253768000
H	1.453638000	-0.908924000	0.660580000
H	-0.041959000	-1.866433000	0.626582000
H	0.011674000	-0.336481000	1.527791000

Compound 19

C	0.018229000	0.192461000	-0.341921000
C	1.288632000	1.026638000	-0.314622000
C	-1.227105000	1.047038000	-0.513363000
F	0.101976000	-0.629865000	-1.485518000
C	-0.084194000	-0.739100000	0.854819000
H	2.165714000	0.376259000	-0.259727000
H	1.289869000	1.691188000	0.554430000
H	1.360496000	1.634587000	-1.220451000
H	-2.113034000	0.411563000	-0.594506000
H	-1.352214000	1.714754000	0.344234000
H	-1.147231000	1.652834000	-1.419951000
H	0.797956000	-1.382676000	0.908733000
H	-0.154789000	-0.162592000	1.781949000

H	-0.972503000	-1.370288000	0.766395000

Compound 20

C	0.989897000	0.111281000	-0.126181000
C	0.189092000	-0.711554000	0.623685000
C	-1.168423000	-0.370229000	0.427246000
C	-1.161007000	0.682443000	-0.463096000
N	0.135986000	0.962170000	-0.787528000
F	0.630961000	-1.694708000	1.424782000
H	2.061105000	0.153758000	-0.234743000
H	-2.028953000	-0.837326000	0.880460000
H	-1.978261000	1.246794000	-0.884714000
H	0.429200000	1.687570000	-1.420613000

Geometries optimized with the 6-311++G(d,p) basis set

Compound 1

C	-0.003379000	-0.036612000	0.051980000
F	1.144479000	0.641411000	0.029572000
F	-1.008360000	0.839373000	0.029656000
F	-0.071682000	-0.781552000	-1.051796000
H	-0.060858000	-0.662620000	0.940488000

Compound 2

C	-0.033135000	-0.057190000	0.000079000
F	0.658232000	1.139272000	-0.000289000
H	0.685864000	-0.877124000	-0.000231000
H	-0.655294000	-0.102475000	0.894468000
H	-0.655667000	-0.102484000	-0.894127000

Compound 3

C	-1.071249000	0.236952000	-0.008408000
F	-1.688578000	-0.220703000	1.065803000
F	-1.684438000	-0.254481000	-1.094358000
F	0.177170000	-0.250917000	-0.022945000
O	-1.090819000	1.577576000	0.021023000
H	-0.643087000	1.920572000	-0.761114000

Compound 4

C	0.549909000	-0.317959000	-0.115124000
C	-0.745355000	0.440562000	-0.099702000
F	1.600795000	0.550486000	-0.090253000
F	0.653195000	-1.089904000	1.004077000
H	0.683341000	-0.971646000	-0.979454000
H	-1.581431000	-0.258767000	-0.093841000
H	-0.810956000	1.075001000	-0.983615000
H	-0.784400000	1.060926000	0.796413000

Compound 5

C	-0.781017000	0.024572000	-0.042586000
C	0.745632000	0.024108000	-0.041838000
F	1.221924000	-0.628884000	1.090684000
H	-1.186483000	1.036129000	-0.025553000
H	-1.186478000	-0.494743000	-0.910839000
H	1.157289000	-0.502669000	-0.904315000
H	-1.128297000	-0.493049000	0.852481000
H	1.157330000	1.034436000	-0.018034000

Compound 6

C	-0.233196000	-0.647909000	0.000000000

C	0.426847000	0.495090000	0.000000000
F	-1.566251000	-0.695459000	0.000000000
F	-0.198634000	1.673578000	0.000000000
H	0.255969000	-1.611052000	0.000000000
H	1.505664000	0.553053000	0.000000000

Compound 7

C	-0.222132000	-0.558326000	0.000000000
C	0.417852000	0.593400000	0.000000000
F	-1.562729000	-0.623734000	0.000000000
H	0.231393000	-1.541716000	0.000000000
H	1.498424000	0.598120000	0.000000000
H	-0.118509000	1.533056000	0.000000000

Compound 8

C	-0.315727000	-0.578727000	0.000000000
C	0.315727000	0.578727000	0.000000000
F	-1.654746000	-0.611237000	0.000000000
F	1.654746000	0.611237000	0.000000000
H	0.161214000	-1.549194000	0.000000000
H	-0.161214000	1.549194000	0.000000000

Compound 9

C	-0.239930000	-0.467770000	0.000000000
C	0.416966000	0.670053000	0.000000000
F	-1.551663000	-0.589382000	0.000000000
F	0.310691000	-1.664547000	0.000000000
H	1.495035000	0.660466000	0.000000000
H	-0.130398000	1.598879000	0.000000000

Compound 10

N	0.592485000	1.026183000	0.000000000
C	0.019112000	0.033101000	0.000000000
F	-0.611597000	-1.059285000	0.000000000

Compound 11

F	0.417786000	-0.190003000	0.000000000
H	-0.417786000	0.190003000	0.000000000

Compound 12

C	-0.553355000	0.060075000	0.000000000
O	0.606370000	0.184511000	0.000000000
F	-1.433972000	1.030522000	0.000000000
F	-1.208043000	-1.075107000	0.000000000

Compound 13

B	-0.450874000	0.132251000	-0.206139000
F	-0.669921000	1.369792000	0.173645000
F	0.642751000	-0.486600000	0.173545000
F	-1.325957000	-0.486445000	-0.964050000

Compound 14

Si	-0.965062000	0.236635000	0.041823000
H	-1.685105000	-0.273205000	1.222033000
F	-1.727523000	-0.302573000	-1.281512000
H	0.417447000	-0.272622000	0.009372000
H	-0.983756000	1.709765000	0.009284000

Compound 15

F	-0.572631000	1.499130000	0.003309000
P	-0.734695000	-0.055904000	0.286254000
F	0.782871000	-0.456701000	0.037403000
F	-1.279545000	-0.457526000	-1.150966000

Compound 16

C	0.120043000	0.167743000	-0.000249000
C	0.708807000	1.211570000	-0.000317000
C	-0.617852000	-1.093097000	-0.000130000
F	0.248964000	-2.170301000	-0.000332000
H	1.238456000	2.134665000	-0.000360000
H	-1.244881000	-1.166212000	0.890739000
H	-1.245237000	-1.166168000	-0.890751000

Compound 17

C	0.295488000	-0.511784000	0.000000000
C	-0.295488000	0.511784000	0.000000000
F	-0.935903000	1.620983000	0.000000000
F	0.935903000	-1.620983000	0.000000000

Compound 18

F	-1.488250000	-0.113770000	-0.633087000
C	-0.088926000	-0.099744000	-0.607422000
C	0.362954000	1.344209000	-0.640549000
C	0.361693000	-0.849888000	0.627291000
H	0.233177000	-0.623041000	-1.512710000
H	1.453159000	1.398585000	-0.673080000
H	-0.038374000	1.849448000	-1.519990000
H	0.013103000	1.865421000	0.253253000
H	1.451835000	-0.906158000	0.658705000

H	-0.040501000	-1.863784000	0.625665000
H	0.011831000	-0.335481000	1.525022000

Compound 19

C	0.017779000	0.192069000	-0.342455000
C	1.286441000	1.025606000	-0.314589000
C	-1.225853000	1.046305000	-0.512883000
F	0.101133000	-0.629760000	-1.484064000
C	-0.083584000	-0.737347000	0.854031000
H	2.161991000	0.376200000	-0.257049000
H	1.285060000	1.689569000	0.552723000
H	1.358722000	1.631582000	-1.219688000
H	-2.109629000	0.411448000	-0.598296000
H	-1.351156000	1.709091000	0.346134000
H	-1.143411000	1.654490000	-1.415592000
H	0.796727000	-1.380799000	0.906090000
H	-0.150805000	-0.159642000	1.778571000
H	-0.971613000	-1.366010000	0.767568000

Compound 20

C	0.988197000	0.110462000	-0.125373000
C	0.189638000	-0.710713000	0.622937000
C	-1.166506000	-0.369093000	0.426156000
C	-1.160275000	0.681341000	-0.462214000
N	0.135553000	0.961314000	-0.786776000
F	0.628877000	-1.690645000	1.421488000
H	2.057675000	0.152787000	-0.233688000
H	-2.025612000	-0.835333000	0.878549000
H	-1.976695000	1.244163000	-0.882597000
H	0.428747000	1.685915000	-1.419183000

Geometries optimized with the 6-311++G(2d,2p) basis set

Compound 1

C	-0.003261000	-0.036584000	0.052204000
F	1.143175000	0.640810000	0.029489000
F	-1.007080000	0.838278000	0.029483000
F	-0.071754000	-0.780584000	-1.050214000
H	-0.060879000	-0.661919000	0.938939000

Compound 2

C	-0.033173000	-0.057482000	0.000151000
F	0.657590000	1.138627000	-0.000468000
H	0.684132000	-0.875953000	-0.000181000
H	-0.654157000	-0.102736000	0.892694000
H	-0.654391000	-0.102457000	-0.892296000

Compound 3

C	-1.070792000	0.236417000	-0.009141000
F	-1.687616000	-0.219878000	1.064067000
F	-1.683546000	-0.253519000	-1.092778000
F	0.175374000	-0.250045000	-0.022886000
O	-1.090851000	1.577450000	0.021199000
H	-0.643571000	1.918575000	-0.760461000

Compound 4

C	0.548712000	-0.317262000	-0.115116000
C	-0.744772000	0.439847000	-0.100269000
F	1.597761000	0.550896000	-0.090207000

F	0.651053000	-1.087949000	1.003092000
H	0.681674000	-0.969663000	-0.977925000
H	-1.578781000	-0.258400000	-0.093715000
H	-0.809483000	1.073329000	-0.982128000
H	-0.781066000	1.057904000	0.794769000

Compound 5

C	-0.779484000	0.024378000	-0.042241000
C	0.744444000	0.024315000	-0.042310000
F	1.220307000	-0.627595000	1.090526000
H	-1.184317000	1.033898000	-0.024748000
H	-1.184332000	-0.492561000	-0.909544000
H	1.154895000	-0.502289000	-0.902682000
H	-1.126636000	-0.492987000	0.850308000
H	1.155022000	1.032740000	-0.019308000

Compound 6

C	-0.231781000	-0.647060000	0.000000000
C	0.426819000	0.493527000	0.000000000
F	-1.564709000	-0.691795000	0.000000000
F	-0.200663000	1.670131000	0.000000000
H	0.257380000	-1.608026000	0.000000000
H	1.503353000	0.550525000	0.000000000

Compound 7

C	-0.221643000	-0.556815000	0.000000000
C	0.417615000	0.592018000	0.000000000
F	-1.561288000	-0.621659000	0.000000000
H	0.232938000	-1.537276000	0.000000000
H	1.495715000	0.595784000	0.000000000
H	-0.119038000	1.528747000	0.000000000

Compound 8

C	-0.316141000	-0.577008000	0.000000000
C	0.316141000	0.577008000	0.000000000
F	-1.653821000	-0.609819000	0.000000000
F	1.653821000	0.609819000	0.000000000
H	0.162368000	-1.544356000	0.000000000
H	-0.162368000	1.544356000	0.000000000

Compound 9

C	-0.239397000	-0.466833000	0.000000000
C	0.416473000	0.669200000	0.000000000
F	-1.549755000	-0.586949000	0.000000000
F	0.311851000	-1.661675000	0.000000000
H	1.492145000	0.658539000	0.000000000
H	-0.130616000	1.595418000	0.000000000

Compound 10

N	0.591907000	1.025182000	0.000000000
C	0.019512000	0.033795000	0.000000000
F	-0.611419000	-1.058978000	0.000000000

Compound 11

F	0.417786000	-0.190003000	0.000000000
H	-0.417786000	0.190003000	0.000000000

Compound 12

C	-0.554071000	0.059998000	0.000000000
O	0.605826000	0.184452000	0.000000000
F	-1.433317000	1.030359000	0.000000000

F	-1.207437000	-1.074808000	0.000000000

Compound 13

B	-0.450950000	0.131785000	-0.205840000
F	-0.669126000	1.365132000	0.172035000
F	0.639451000	-0.484109000	0.172479000
F	-1.323376000	-0.483810000	-0.961675000

Compound 14

Si	-0.966388000	0.235513000	0.039399000
H	-1.683136000	-0.271504000	1.217580000
F	-1.721889000	-0.298526000	-1.272731000
H	0.411883000	-0.271221000	0.008323000
H	-0.984469000	1.703738000	0.008428000

Compound 15

F	-0.571771000	1.490165000	0.002414000
P	-0.732518000	-0.054138000	0.282551000
F	0.773162000	-0.452858000	0.035092000
F	-1.272873000	-0.454171000	-1.144058000

Compound 16

C	0.123767000	0.167135000	-0.000259000
C	0.707477000	1.210007000	-0.000320000
C	-0.616953000	-1.092384000	-0.000129000
F	0.246515000	-2.170709000	-0.000330000
H	1.234409000	2.132704000	-0.000352000
H	-1.243280000	-1.164295000	0.888558000
H	-1.243635000	-1.164258000	-0.888567000

Compound 17

C	0.294825000	-0.510637000	0.000000000
C	-0.294825000	0.510637000	0.000000000
F	-0.935742000	1.620706000	0.000000000
F	0.935742000	-1.620706000	0.000000000

Compound 18

F	-1.486015000	-0.112488000	-0.630867000
C	-0.088932000	-0.099525000	-0.607044000
C	0.363181000	1.342297000	-0.640082000
C	0.361921000	-0.848529000	0.625868000
H	0.232656000	-0.621810000	-1.510578000
H	1.451167000	1.396322000	-0.671191000
H	-0.037247000	1.846406000	-1.517791000
H	0.012884000	1.861061000	0.252234000
H	1.449845000	-0.903384000	0.657692000
H	-0.039365000	-1.860363000	0.624125000
H	0.011607000	-0.334189000	1.520735000

Compound 19

C	0.017970000	0.192497000	-0.342726000
C	1.285348000	1.025048000	-0.313673000
C	-1.224360000	1.045875000	-0.511914000
F	0.101188000	-0.626912000	-1.482851000
C	-0.083778000	-0.735879000	0.852317000
H	2.158176000	0.375908000	-0.256677000
H	1.283828000	1.686904000	0.552232000
H	1.357200000	1.629490000	-1.216887000
H	-2.105515000	0.410909000	-0.592995000
H	-1.347641000	1.709148000	0.343972000
H	-1.143859000	1.649626000	-1.414870000
H	0.795423000	-1.376770000	0.905244000

H	-0.152780000	-0.159327000	1.774588000
H	-0.969402000	-1.363715000	0.764740000

Compound 20

C	0.986558000	0.110498000	-0.125291000
C	0.189171000	-0.709546000	0.621981000
C	-1.165271000	-0.368207000	0.425324000
C	-1.158611000	0.680547000	-0.461655000
N	0.135117000	0.960508000	-0.786065000
F	0.628314000	-1.688623000	1.419815000
H	2.053618000	0.152664000	-0.233307000
H	-2.022822000	-0.832914000	0.876316000
H	-1.973246000	1.241968000	-0.880976000
H	0.426771000	1.683303000	-1.416842000

Geometries optimized with the 6-311++G(3df,3pd) basis set

Compound 1

C	-0.003318000	-0.035954000	0.051113000
F	1.140774000	0.639411000	0.029223000
F	-1.005083000	0.836725000	0.029199000
F	-0.071477000	-0.778684000	-1.048525000
H	-0.060696000	-0.661498000	0.938890000

Compound 2

C	-0.032011000	-0.055497000	0.000000000
F	0.656336000	1.136018000	-0.000135000
H	0.683767000	-0.875231000	-0.000240000
H	-0.653782000	-0.102533000	0.891857000
H	-0.654310000	-0.102759000	-0.891582000

Compound 3

C	-1.070927000	0.234818000	-0.008882000
F	-1.686628000	-0.219673000	1.062321000
F	-1.682600000	-0.252515000	-1.090696000
F	0.173112000	-0.249060000	-0.022662000
O	-1.090415000	1.576109000	0.020464000
H	-0.643544000	1.919321000	-0.760545000

Compound 4

C	0.549801000	-0.317055000	-0.113863000
C	-0.744633000	0.440251000	-0.099543000
F	1.596958000	0.547861000	-0.089401000
F	0.652872000	-1.086445000	1.000871000
H	0.680285000	-0.968911000	-0.977999000
H	-1.577679000	-0.258327000	-0.094110000
H	-0.809013000	1.072309000	-0.981793000
H	-0.783493000	1.059018000	0.794339000

Compound 5

C	-0.779298000	0.024181000	-0.041900000
C	0.744910000	0.023017000	-0.040038000
F	1.220269000	-0.626329000	1.088002000
H	-1.183195000	1.033665000	-0.025192000
H	-1.183197000	-0.492342000	-0.909398000
H	1.153783000	-0.501796000	-0.902323000
H	-1.127273000	-0.492689000	0.850121000
H	1.153903000	1.032192000	-0.019272000

Compound 6

C	-0.234629000	-0.645392000	0.000000000

C	0.423892000	0.495155000	0.000000000
F	-1.563398000	-0.697661000	0.000000000
F	-0.195287000	1.672203000	0.000000000
H	0.258859000	-1.603980000	0.000000000
H	1.500963000	0.546976000	0.000000000

Compound 7

C	-0.223150000	-0.556211000	0.000000000
C	0.416286000	0.592063000	0.000000000
F	-1.558629000	-0.623903000	0.000000000
H	0.232448000	-1.536243000	0.000000000
H	1.494221000	0.594546000	0.000000000
H	-0.116876000	1.530548000	0.000000000

Compound 8

C	-0.316466000	-0.576661000	0.000000000
C	0.316466000	0.576661000	0.000000000
F	-1.650934000	-0.610337000	0.000000000
F	1.650934000	0.610337000	0.000000000
H	0.160877000	-1.544480000	0.000000000
H	-0.160877000	1.544480000	0.000000000

Compound 9

C	-0.239608000	-0.467199000	0.000000000
C	0.416170000	0.668675000	0.000000000
F	-1.546745000	-0.588542000	0.000000000
F	0.308967000	-1.659865000	0.000000000
H	1.491663000	0.659267000	0.000000000
H	-0.129745000	1.595364000	0.000000000

Compound 10

N	0.591417000	1.024334000	0.000000000
C	0.019191000	0.033239000	0.000000000
F	-0.610609000	-1.057574000	0.000000000

Compound 11

F	0.417786000	-0.190003000	0.000000000
H	-0.417786000	0.190003000	0.000000000

Compound 12

C	-0.554914000	0.059908000	0.000000000
O	0.604218000	0.184280000	0.000000000
F	-1.431746000	1.027264000	0.000000000
F	-1.206558000	-1.071450000	0.000000000

Compound 13

B	-0.450940000	0.131899000	-0.205846000
F	-0.668893000	1.363637000	0.171578000
F	0.637966000	-0.483439000	0.171915000
F	-1.322134000	-0.483099000	-0.960647000

Compound 14

Si	-0.967291000	0.234823000	0.037516000
H	-1.684019000	-0.271928000	1.217761000
F	-1.721675000	-0.298751000	-1.271297000
H	0.413400000	-0.271798000	0.008214000
H	-0.984416000	1.705654000	0.008807000

Compound 15

F	-0.571379000	1.485054000	0.002676000
P	-0.729952000	-0.052759000	0.278197000
F	0.769683000	-0.450599000	0.036154000
F	-1.272352000	-0.452697000	-1.141028000

Compound 16

C	0.122453000	0.166820000	-0.000213000
C	0.707683000	1.208973000	-0.000301000
C	-0.615115000	-1.093609000	-0.000132000
F	0.245571000	-2.167808000	-0.000331000
H	1.234228000	2.131949000	-0.000395000
H	-1.243088000	-1.164102000	0.887854000
H	-1.243431000	-1.164023000	-0.887882000

Compound 17

C	0.294876000	-0.510724000	0.000000000
C	-0.294876000	0.510724000	0.000000000
F	-0.934472000	1.618505000	0.000000000
F	0.934472000	-1.618505000	0.000000000

Compound 18

F	-1.484151000	-0.112973000	-0.631704000
C	-0.091821000	-0.099295000	-0.606649000
C	0.362713000	1.341960000	-0.639594000
C	0.361453000	-0.847938000	0.625820000
H	0.232712000	-0.621165000	-1.509462000
H	1.450360000	1.393278000	-0.670223000
H	-0.035857000	1.845995000	-1.517705000
H	0.013252000	1.861666000	0.252082000
H	1.449041000	-0.901024000	0.655538000

H	-0.037974000	-1.860085000	0.623813000
H	0.011974000	-0.334623000	1.521184000

Compound 19

C	0.018264000	0.189806000	-0.345513000
C	1.284577000	1.024379000	-0.314598000
C	-1.223642000	1.044824000	-0.512344000
F	0.101144000	-0.627884000	-1.480576000
C	-0.083599000	-0.735282000	0.852549000
H	2.158229000	0.376907000	-0.257404000
H	1.280578000	1.684609000	0.551852000
H	1.355677000	1.630515000	-1.216355000
H	-2.105727000	0.412101000	-0.597299000
H	-1.346179000	1.703767000	0.346361000
H	-1.141402000	1.653395000	-1.411523000
H	0.794949000	-1.376417000	0.906041000
H	-0.151263000	-0.155869000	1.772519000
H	-0.969805000	-1.362049000	0.766790000

Compound 20

C	0.985542000	0.110279000	-0.125035000
C	0.189455000	-0.710052000	0.622389000
C	-1.164376000	-0.368357000	0.425390000
C	-1.157520000	0.679819000	-0.461114000
N	0.134649000	0.959333000	-0.785041000
F	0.626008000	-1.686071000	1.417812000
H	2.052588000	0.153785000	-0.234183000
H	-2.021894000	-0.833302000	0.876583000
H	-1.971330000	1.242186000	-0.881290000
H	0.426477000	1.682578000	-1.416211000

Geometries optimized with the pecG-1 basis set

Compound 1

C	-0.003290000	-0.034589000	0.049048000
F	1.140491000	0.638569000	0.030231000
F	-1.004922000	0.835832000	0.029852000
F	-0.071668000	-0.778951000	-1.047774000
H	-0.060412000	-0.660861000	0.938541000

Compound 2

C	-0.027952000	-0.048460000	-0.000031000
F	0.657171000	1.137779000	-0.000291000
H	0.681192000	-0.877823000	-0.000118000
H	-0.654976000	-0.105796000	0.891257000
H	-0.655436000	-0.105700000	-0.890917000

Compound 3

C	-1.070545000	0.237280000	-0.009663000
F	-1.686159000	-0.217805000	1.061611000
F	-1.681321000	-0.249435000	-1.091436000
F	0.173058000	-0.245867000	-0.024200000
O	-1.092440000	1.576211000	0.023795000
H	-0.643594000	1.908616000	-0.760108000

Compound 4

C	0.555160000	-0.318662000	-0.111631000
C	-0.742349000	0.437889000	-0.101106000
F	1.593273000	0.550561000	-0.089595000

F	0.648732000	-1.084533000	1.001202000
H	0.681422000	-0.970862000	-0.979941000
H	-1.580742000	-0.257896000	-0.093293000
H	-0.810853000	1.074857000	-0.982390000
H	-0.779545000	1.057348000	0.795253000

Compound 5

C	-0.770345000	0.009670000	-0.032130000
C	0.739464000	-0.005777000	-0.041186000
F	1.208138000	-0.070461000	1.252080000
H	-1.138070000	0.874392000	0.520371000
H	-1.153135000	0.059583000	-1.052834000
H	1.125692000	-0.872596000	-0.584132000
H	-1.156313000	-0.893985000	0.439842000
H	1.144469000	0.899074000	-0.502011000

Compound 6

C	-0.235473000	-0.646016000	0.000000000
C	0.423966000	0.496116000	0.000000000
F	-1.563585000	-0.694164000	0.000000000
F	-0.198370000	1.670788000	0.000000000
H	0.260943000	-1.605230000	0.000000000
H	1.502919000	0.545807000	0.000000000

Compound 7

C	-0.226705000	-0.556534000	0.000000000
C	0.418077000	0.590715000	0.000000000
F	-1.558750000	-0.620887000	0.000000000
H	0.234519000	-1.536069000	0.000000000
H	1.497261000	0.595650000	0.000000000
H	-0.120102000	1.527925000	0.000000000

Compound 8

C	-0.319885000	-0.576265000	0.000000000
C	0.319885000	0.576265000	0.000000000
F	-1.651427000	-0.607661000	0.000000000
F	1.651427000	0.607661000	0.000000000
H	0.165051000	-1.542168000	0.000000000
H	-0.165051000	1.542168000	0.000000000

Compound 9

C	-0.240836000	-0.469344000	0.000000000
C	0.416357000	0.668998000	0.000000000
F	-1.547313000	-0.586446000	0.000000000
F	0.311056000	-1.659311000	0.000000000
H	1.493057000	0.657914000	0.000000000
H	-0.131620000	1.595890000	0.000000000

Compound 10

N	0.592876000	1.026861000	0.000000000
C	0.018506000	0.032051000	0.000000000
F	-0.611382000	-1.058913000	0.000000000

Compound 11

F	0.417786000	-0.190003000	0.000000000
H	-0.417786000	0.190003000	0.000000000

Compound 12

C	-0.554139000	0.059991000	0.000000000
O	0.605960000	0.184467000	0.000000000
F	-1.433130000	1.028305000	0.000000000

F	-1.207691000	-1.072761000	0.000000000

Compound 13

B	-0.451387000	0.131944000	-0.205119000
F	-0.669056000	1.365685000	0.171998000
F	0.639402000	-0.484436000	0.172016000
F	-1.322960000	-0.484194000	-0.961893000

Compound 14

Si	-0.971739000	0.231829000	0.029507000
H	-1.679953000	-0.269171000	1.220775000
F	-1.725192000	-0.301462000	-1.276952000
H	0.414234000	-0.269020000	0.013490000
H	-0.981350000	1.705825000	0.014180000

Compound 15

F	-0.572390000	1.490777000	0.003442000
P	-0.731039000	-0.053393000	0.279865000
F	0.774776000	-0.453563000	0.037061000
F	-1.275348000	-0.454822000	-1.144369000

Compound 16

C	0.121444000	0.165916000	-0.000255000
C	0.707815000	1.209601000	-0.000319000
C	-0.612794000	-1.097982000	-0.000133000
F	0.248972000	-2.165132000	-0.000328000
H	1.236495000	2.132645000	-0.000355000
H	-1.246635000	-1.163445000	0.887207000
H	-1.246998000	-1.163403000	-0.887217000

Compound 17

C	0.295722000	-0.512191000	0.000000000
C	-0.295722000	0.512191000	0.000000000
F	-0.936095000	1.621316000	0.000000000
F	0.936095000	-1.621316000	0.000000000

Compound 18

F	-1.479588000	-0.110791000	-0.627923000
C	-0.094078000	-0.099559000	-0.607108000
C	0.362547000	1.342280000	-0.641105000
C	0.361287000	-0.849407000	0.625343000
H	0.239329000	-0.621714000	-1.510407000
H	1.452005000	1.400252000	-0.674709000
H	-0.041318000	1.846686000	-1.518968000
H	0.012779000	1.864204000	0.251678000
H	1.450678000	-0.908396000	0.659339000
H	-0.043438000	-1.861518000	0.623779000
H	0.011501000	-0.336239000	1.523180000

Compound 19

C	0.018302000	0.189846000	-0.346084000
C	1.285611000	1.025291000	-0.313294000
C	-1.224579000	1.045962000	-0.511873000
F	0.101079000	-0.622216000	-1.475953000
C	-0.083832000	-0.736074000	0.852715000
H	2.160287000	0.375834000	-0.259827000
H	1.288443000	1.688366000	0.553532000
H	1.358039000	1.630750000	-1.217528000
H	-2.107922000	0.411271000	-0.593181000
H	-1.350410000	1.713262000	0.342366000
H	-1.145492000	1.647864000	-1.417980000
H	0.795944000	-1.378597000	0.905478000

H	-0.154161000	-0.162336000	1.778377000
H	-0.969510000	-1.366422000	0.763753000

Compound 20

C	0.190215000	-0.711497000	0.623558000
C	-1.164135000	-0.367854000	0.424950000
C	-1.157311000	0.679923000	-0.461216000
N	0.134907000	0.959351000	-0.785074000
F	0.626697000	-1.685540000	1.417316000
H	2.053109000	0.153926000	-0.234337000
H	-2.023167000	-0.832278000	0.875805000
H	-1.972143000	1.242113000	-0.881174000
H	0.425816000	1.681340000	-1.415120000

Geometries optimized with the pecG-2 basis set

Compound 1

C	-0.003397000	-0.036868000	0.051962000
F	1.142498000	0.640542000	0.029551000
F	-1.006557000	0.838109000	0.029339000
F	-0.071722000	-0.780115000	-1.050339000
H	-0.060622000	-0.661668000	0.939387000

Compound 2

C	-0.032447000	-0.055998000	-0.000016000
F	0.657678000	1.137950000	-0.000231000
H	0.683330000	-0.875407000	-0.000223000
H	-0.654081000	-0.103232000	0.891760000
H	-0.654481000	-0.103311000	-0.891390000

Compound 3

C	-1.071148000	0.236188000	-0.008585000
F	-1.687708000	-0.220440000	1.064310000
F	-1.683353000	-0.253267000	-1.092582000
F	0.175052000	-0.249715000	-0.022959000
O	-1.090464000	1.576881000	0.020478000
H	-0.643381000	1.919354000	-0.760662000

Compound 4

C	0.548779000	-0.316669000	-0.114169000
C	-0.744681000	0.440171000	-0.099705000
F	1.598563000	0.549089000	-0.089965000
F	0.652833000	-1.088063000	1.002206000
H	0.679553000	-0.968286000	-0.977697000
H	-1.577530000	-0.258267000	-0.094372000
H	-0.809072000	1.072008000	-0.981814000
H	-0.783347000	1.058718000	0.794016000

Compound 5

C	-0.778786000	0.024411000	-0.042304000
C	0.744487000	0.023857000	-0.041437000
F	1.218260000	-0.627962000	1.089735000
H	-1.183488000	1.032922000	-0.025359000
H	-1.183489000	-0.492757000	-0.908288000
H	1.154206000	-0.500757000	-0.902911000
H	-1.125566000	-0.492048000	0.850102000
H	1.154274000	1.032233000	-0.019537000

Compound 6

C	-0.233748000	-0.645606000	0.000000000

C	0.424580000	0.494572000	0.000000000
F	-1.565287000	-0.698721000	0.000000000
F	-0.195335000	1.674212000	0.000000000
H	0.258926000	-1.604163000	0.000000000
H	1.501263000	0.547007000	0.000000000

Compound 7

C	-0.222349000	-0.555758000	0.000000000
C	0.416987000	0.591852000	0.000000000
F	-1.560361000	-0.624134000	0.000000000
H	0.232449000	-1.535721000	0.000000000
H	1.494589000	0.594981000	0.000000000
H	-0.117015000	1.529580000	0.000000000

Compound 8

C	-0.316304000	-0.576645000	0.000000000
C	0.316304000	0.576645000	0.000000000
F	-1.653232000	-0.610921000	0.000000000
F	1.653232000	0.610921000	0.000000000
H	0.161067000	-1.544133000	0.000000000
H	-0.161067000	1.544133000	0.000000000

Compound 9

C	-0.239083000	-0.466289000	0.000000000
C	0.416505000	0.669255000	0.000000000
F	-1.548631000	-0.588402000	0.000000000
F	0.310032000	-1.661427000	0.000000000
H	1.491746000	0.659172000	0.000000000
H	-0.129868000	1.595391000	0.000000000

Compound 10

N	0.591941000	1.025241000	0.000000000
C	0.019480000	0.033739000	0.000000000
F	-0.611421000	-1.058981000	0.000000000

Compound 11

F	0.418102000	-0.190147000	0.000000000
H	-0.418102000	0.190147000	0.000000000

Compound 12

C	-0.553938000	0.060013000	0.000000000
O	0.605136000	0.184379000	0.000000000
F	-1.432924000	1.029314000	0.000000000
F	-1.207275000	-1.073703000	0.000000000

Compound 13

B	-0.451315000	0.131964000	-0.205102000
F	-0.669023000	1.365342000	0.171887000
F	0.639035000	-0.484277000	0.171879000
F	-1.322698000	-0.484030000	-0.961664000

Compound 14

Si	-0.968464000	0.234218000	0.035222000
H	-1.682731000	-0.271303000	1.216627000
F	-1.720856000	-0.298175000	-1.269326000
H	0.412176000	-0.271204000	0.009056000
H	-0.984125000	1.704465000	0.009420000

Compound 15

F	-0.571851000	1.482313000	0.001668000
P	-0.729424000	-0.052449000	0.277841000
F	0.766822000	-0.450089000	0.035048000
F	-1.269547000	-0.450775000	-1.138557000

Compound 16

C	0.121853000	0.167116000	-0.000201000
C	0.707253000	1.208639000	-0.000296000
C	-0.615870000	-1.091789000	-0.000129000
F	0.247140000	-2.169301000	-0.000337000
H	1.234184000	2.131033000	-0.000406000
H	-1.242963000	-1.163791000	0.887622000
H	-1.243297000	-1.163707000	-0.887652000

Compound 17

C	0.294840000	-0.510663000	0.000000000
C	-0.294840000	0.510663000	0.000000000
F	-0.935598000	1.620455000	0.000000000
F	0.935598000	-1.620455000	0.000000000

Compound 18

F	-1.485625000	-0.111814000	-0.629700000
C	-0.089197000	-0.099100000	-0.606309000
C	0.363175000	1.341516000	-0.639928000
C	0.361917000	-0.848006000	0.625269000
H	0.231985000	-0.621079000	-1.509314000
H	1.450347000	1.395591000	-0.670278000
H	-0.035947000	1.844617000	-1.517751000
H	0.012680000	1.861034000	0.250937000
H	1.449027000	-0.902228000	0.657513000

H	-0.038065000	-1.859436000	0.622597000
H	0.011404000	-0.335298000	1.520064000

Compound 19

C	0.018414000	0.191942000	-0.342700000
C	1.284630000	1.024547000	-0.314509000
C	-1.223203000	1.044921000	-0.511403000
F	0.101383000	-0.628041000	-1.481557000
C	-0.083644000	-0.734782000	0.852505000
H	2.157156000	0.376350000	-0.257528000
H	1.283293000	1.686659000	0.550164000
H	1.355461000	1.628388000	-1.217311000
H	-2.104166000	0.411486000	-0.597052000
H	-1.348570000	1.704474000	0.346132000
H	-1.141297000	1.652677000	-1.410669000
H	0.794509000	-1.375607000	0.906152000
H	-0.153270000	-0.158317000	1.773877000
H	-0.968896000	-1.361896000	0.764399000

Compound 20

C	0.985480000	0.110603000	-0.125306000
C	0.189204000	-0.708986000	0.621505000
C	-1.164440000	-0.367554000	0.424716000
C	-1.157618000	0.679761000	-0.461058000
N	0.134722000	0.959730000	-0.785381000
F	0.626876000	-1.687512000	1.418973000
H	2.052016000	0.153054000	-0.233528000
H	-2.021286000	-0.832613000	0.875959000
H	-1.971414000	1.241404000	-0.880623000
H	0.426059000	1.682313000	-1.415957000

Table S2. Values of equilibrium bond lengths (in Å) of compounds **1-20** calculated at the DFT(M062X) level of theory with various one-electron basis sets.

Mol.	Basis sets								
	cc-PVDZ	aug-cc-PVDZ	cc-PVTZ	aug-cc-PVTZ	cc-PVQZ	aug-cc-PVQZ	cc-PV5Z	aug-cc-PV5Z	cc-PV6Z
**1**	1.333	1.339	1.331	1.332	1.331	1.331	1.331	1.331	1.331
**2**	1.375	1.388	1.377	1.379	1.378	1.378	1.378	1.378	1.378
**3**	1.334	1.339	1.331	1.332	1.331	1.331	1.331	1.331	1.331
**4**	1.361	1.369	1.360	1.361	1.360	1.360	1.360	1.360	1.360
**5**	1.384	1.395	1.388	1.389	1.387	1.388	1.388	1.388	1.388
**6**	1.335	1.339	1.332	1.333	1.331	1.331	1.331	1.331	1.331
**7**	1.340	1.347	1.339	1.340	1.339	1.339	1.339	1.339	1.339
**8**	1.338	1.345	1.337	1.338	1.336	1.336	1.336	1.336	1.336
**9**	1.318	1.322	1.315	1.315	1.314	1.314	1.314	1.314	1.314
**10**	1.268	1.269	1.262	1.262	1.261	1.261	1.261	1.261	1.261
**11**	0.922	0.921	0.918	0.920	0.918	0.918	0.918	0.918	0.918
**12**	1.312	1.316	1.308	1.309	1.308	1.308	1.308	1.308	1.308
**13**	1.319	1.323	1.309	1.310	1.308	1.308	1.308	1.308	1.308
**14**	1.645	1.644	1.606	1.607	1.597	1.598	1.595	1.595	1.595
**15**	1.611	1.613	1.576	1.573	1.569	1.570	1.567	1.567	1.566
**16**	1.378	1.388	1.378	1.381	1.378	1.379	1.379	1.379	1.379
**17**	1.287	1.287	1.282	1.282	1.281	1.280	1.280	1.280	1.280
**18**	1.394	1.404	1.394	1.397	1.394	1.395	1.395	1.395	1.395
**19**	1.403	1.414	1.403	1.406	1.404	1.405	1.404	1.405	1.405
**20**	1.338	1.343	1.335	1.336	1.335	1.335	1.335	1.335	1.335

Table S2. Continue.

Mol.	Basis sets									
	pc-1	aug-pc-1	pc-2	aug-pc-2	pc-3	aug-pc-3	pc-4	aug-pc-4	pecG-1	pecG-2
**1**	1.333	1.335	1.331	1.331	1.331	1.331	1.331	1.331	1.327	1.331
**2**	1.380	1.385	1.378	1.379	1.378	1.378	1.378	1.378	1.370	1.379
**3**	1.334	1.335	1.331	1.332	1.331	1.331	1.331	1.331	1.328	1.331
**4**	1.364	1.365	1.360	1.361	1.360	1.360	1.360	1.360	1.354	1.361
**5**	1.389	1.394	1.388	1.388	1.388	1.388	1.388	1.388	1.377	1.389
**6**	1.337	1.337	1.332	1.332	1.331	1.331	1.331	1.331	1.329	1.333
**7**	1.343	1.346	1.339	1.339	1.339	1.339	1.339	1.339	1.334	1.340
**8**	1.340	1.342	1.337	1.337	1.336	1.336	1.336	1.336	1.332	1.337
**9**	1.319	1.320	1.315	1.315	1.314	1.314	1.314	1.314	1.312	1.315
**10**	1.267	1.267	1.262	1.262	1.261	1.261	1.261	1.261	1.260	1.262
**11**	0.926	0.924	0.918	0.919	0.918	0.918	0.918	0.918	0.918	0.919
**12**	1.312	1.313	1.308	1.309	1.308	1.308	1.308	1.308	1.308	1.309
**13**	1.314	1.314	1.308	1.309	1.308	1.308	1.308	1.308	1.308	1.308
**14**	1.622	1.631	1.595	1.597	1.594	1.594	1.594	1.594	1.600	1.597
**15**	1.599	1.602	1.567	1.570	1.566	1.566	1.566	1.566	1.577	1.567
**16**	1.381	1.384	1.379	1.380	1.378	1.379	1.379	1.379	1.372	1.381
**17**	1.288	1.286	1.281	1.281	1.280	1.280	1.280	1.280	1.281	1.281
**18**	1.399	1.401	1.395	1.396	1.395	1.395	1.395	1.395	1.386	1.397
**19**	1.409	1.411	1.404	1.405	1.404	1.404	1.404	1.405	1.394	1.406
**20**	1.340	1.341	1.336	1.336	1.335	1.335	1.335	1.334	1.330	1.336

Table S2. Continue.

Mol.	Basis sets					
	6-31G(d,p)	6-311G(d,p)	6-31++G(d,p)	6-311++G(d,p)	6-311++G(2d,2p)	6-311++G(3df,3pd)
**1**	1.333	1.332	1.337	1.333	1.332	1.329
**2**	1.375	1.379	1.386	1.382	1.381	1.376
**3**	1.334	1.333	1.338	1.334	1.332	1.329
**4**	1.360	1.361	1.367	1.364	1.362	1.358
**5**	1.383	1.387	1.395	1.391	1.391	1.386
**6**	1.336	1.334	1.339	1.334	1.334	1.330
**7**	1.340	1.340	1.347	1.342	1.341	1.337
**8**	1.339	1.338	1.344	1.339	1.338	1.335
**9**	1.319	1.316	1.323	1.317	1.316	1.313
**10**	1.269	1.262	1.270	1.261	1.262	1.260
**11**	0.921	0.916	0.923	0.918	0.918	0.918
**12**	1.312	1.310	1.316	1.310	1.309	1.306
**13**	1.314	1.312	1.316	1.313	1.308	1.307
**14**	1.606	1.612	1.621	1.620	1.606	1.602
**15**	1.584	1.585	1.593	1.589	1.577	1.571
**16**	1.378	1.380	1.386	1.383	1.381	1.376
**17**	1.289	1.282	1.290	1.281	1.282	1.279
**18**	1.391	1.396	1.402	1.400	1.397	1.393
**19**	1.400	1.405	1.411	1.409	1.407	1.401
**20**	1.338	1.337	1.343	1.338	1.337	1.333

Table S3. ^{19}F NMR shielding constants (in ppm) of molecules **1-20** calculated within the GIAO-DFT method with different exchange-correlation functionals using the pcS-3 basis set, in the gas phase.

Mol.	DFT functionals													
	PW91	TPSSh	SVWN	PBE	M062X	O3LYP	PBE0	B97-2	B3LYP	X3LYP	ωB97-XD	CAM-B3LYP	BHandH	BHandHLYP
**1**	242.17	261.25	238.46	242.71	261.19	255.53	261.19	263.22	255.94	256.23	265.45	260.62	270.01	274.52
**2**	459.20	461.07	471.85	459.63	475.54	458.39	468.71	464.57	464.25	465.18	469.65	471.73	484.04	474.20
**3**	224.31	240.74	218.02	224.86	234.72	237.49	239.93	242.43	235.63	236.01	242.69	238.93	246.27	251.83
**4**	279.67	317.34	277.33	279.99	301.52	292.48	299.16	300.81	294.20	294.44	303.86	300.22	309.08	313.20
**5**	384.67	396.44	391.57	385.21	406.58	390.58	400.79	399.44	395.19	395.61	404.55	403.30	414.07	410.74
**6**	175.31	334.44	321.36	320.57	343.85	331.37	339.98	339.53	336.92	338.09	345.30	347.38	357.15	357.90
**7**	260.16	281.95	257.04	259.95	287.25	276.37	285.28	286.70	282.94	284.01	292.61	294.36	303.47	309.16
**8**	349.63	362.60	351.00	349.71	374.73	360.21	369.07	368.06	366.31	367.71	373.35	376.62	387.65	387.26
**9**	234.33	252.09	230.98	234.30	251.87	248.43	254.03	255.79	252.30	253.16	259.60	260.71	268.15	273.69
**10**	348.91	352.65	352.83	348.41	353.10	353.12	358.26	356.73	360.86	361.98	362.51	369.76	373.03	372.10
**11**	410.27	410.99	414.46	410.35	409.35	410.62	411.94	410.91	410.49	410.84	411.83	413.43	416.01	412.42
**12**	183.42	198.63	178.34	183.92	191.70	196.98	199.56	201.75	197.25	198.11	202.65	203.44	209.69	214.98
**13**	293.94	306.57	285.21	293.98	307.15	308.51	307.59	312.34	305.68	306.15	313.05	308.81	312.54	320.14
**14**	405.12	408.55	408.77	405.61	409.19	407.95	411.21	410.75	406.83	407.16	410.91	409.59	415.51	412.59
**15**	183.33	205.36	173.19	183.56	205.43	203.12	204.89	210.14	201.14	201.32	208.11	204.78	211.62	223.18
**16**	404.60	412.10	414.72	405.41	422.81	407.29	417.64	415.12	411.90	412.36	420.31	420.01	430.77	424.32
**17**	454.45	456.49	456.05	454.44	460.64	457.12	460.53	459.27	461.01	461.95	462.50	467.15	471.04	469.28
**18**	345.99	363.42	346.05	346.17	368.86	356.45	364.24	365.15	359.33	359.46	369.07	366.64	374.68	377.29
**19**	316.65	198.63	311.44	316.35	335.82	330.03	334.09	336.53	330.71	330.92	338.33	336.68	342.83	349.32
**20**	329.28	342.51	329.66	329.28	348.96	339.44	346.86	346.96	345.27	346.32	351.73	354.58	362.93	364.39

Table S4. ^{19}F NMR shielding constants (in ppm) of molecules **1-20** calculated within the GIAO-DFT(BHandHLYP) method with different basis sets, in the gas phase.

Mol.	Basis sets								
	cc-pVDZ	aug-cc-pVDZ	cc-pVTZ	aug-cc-pVTZ	cc-pVQZ	aug-cc-pVQZ	cc-pV5Z	aug-cc-pV5Z	cc-pV6Z
**1**	293.23	288.01	277.61	278.55	276.95	276.87	274.98	274.91	274.55
**2**	468.99	475.36	471.55	473.82	474.39	474.64	474.52	474.39	474.30
**3**	279.61	266.26	257.70	256.54	255.44	254.45	252.47	252.28	251.90
**4**	331.63	324.76	315.38	316.46	315.15	315.07	313.52	313.55	313.24
**5**	413.86	414.92	411.73	411.96	411.91	411.92	411.30	411.04	410.87
**6**	366.57	367.68	359.81	359.95	359.93	359.44	358.44	358.13	358.02
**7**	313.62	322.64	307.74	312.29	309.68	311.19	309.46	309.53	309.24
**8**	389.79	395.23	385.64	388.53	387.64	388.53	387.46	387.47	387.35
**9**	287.75	289.21	274.27	277.24	275.15	275.96	273.86	274.01	273.65
**10**	377.30	380.29	370.58	373.66	373.06	373.55	372.02	372.36	372.17
**11**	421.37	419.22	414.07	413.03	413.25	413.28	412.40	412.65	412.48
**12**	237.99	233.08	219.57	220.35	218.20	217.86	215.50	215.47	215.04
**13**	325.44	330.03	319.46	324.70	320.99	321.70	320.42	320.48	320.29
**14**	431.17	434.46	416.42	422.80	416.94	418.07	413.68	413.61	412.94
**15**	248.53	250.21	230.88	235.93	229.28	229.22	224.32	224.42	223.35
**16**	437.41	427.79	429.27	425.06	426.80	425.24	424.86	424.55	424.51
**17**	469.86	468.43	466.81	468.67	470.07	469.79	469.16	469.45	469.38
**18**	391.71	385.34	378.50	378.92	378.81	378.48	377.64	377.52	377.41
**19**	371.45	360.98	351.61	350.88	351.08	350.69	349.49	349.58	349.40
**20**	375.00	374.93	364.98	365.92	365.93	365.80	364.65	364.55	364.45

Table S4. Continue.

Mol.	Basis sets															
	pc-1	aug-pc-1	pc-2	aug-pc-2	pc-3	aug-pc-3	pc-4	aug-pc-4	pcS-1	aug-pcS-1	pcS-2	aug-pcS-2	pcS-3	aug-pcS-3	pcS-4	aug-pcS-4
**1**	285.89	280.70	272.24	273.16	273.92	274.05	274.45	274.47	276.30	273.65	275.45	275.28	274.52	274.50	274.52	274.51
**2**	469.83	475.38	469.75	473.00	474.16	474.48	474.27	474.28	468.63	476.29	475.09	477.10	474.18	474.30	474.33	474.33
**3**	268.90	256.22	250.77	250.54	251.22	251.26	251.76	251.77	260.02	250.06	253.24	252.39	251.85	251.81	251.82	251.81
**4**	324.85	321.02	310.45	311.71	312.66	312.90	313.13	313.16	316.92	313.73	314.34	314.57	313.18	313.17	313.20	313.19
**5**	411.94	415.43	407.72	409.47	410.65	410.84	410.81	410.83	409.38	412.53	412.27	413.20	410.82	410.84	410.87	410.86
**6**	362.78	366.11	355.75	356.95	357.70	357.74	357.88	357.89	358.00	359.21	359.24	359.71	357.91	357.91	357.94	357.93
**7**	310.67	321.05	306.21	307.44	308.84	308.88	309.16	309.17	301.93	310.96	309.37	310.97	309.18	309.21	309.22	309.22
**8**	388.25	395.41	384.68	385.77	387.09	387.20	387.27	387.29	384.07	388.60	389.01	389.59	387.31	387.31	387.33	387.32
**9**	282.21	283.33	271.70	272.30	273.12	273.17	273.55	273.53	270.70	274.63	274.77	274.68	273.63	273.61	273.62	273.62
**10**	376.30	379.97	368.85	371.01	371.86	371.95	372.10	372.11	369.05	373.45	373.13	373.65	372.10	372.11	372.14	372.14
**11**	418.27	417.65	410.42	410.99	412.14	412.39	412.54	412.57	413.90	413.47	413.67	414.27	412.42	412.56	412.56	412.58
**12**	228.62	224.18	213.44	213.74	214.30	214.39	214.91	214.95	217.27	213.82	215.66	215.27	214.96	214.96	214.99	214.98
**13**	325.11	328.08	316.83	319.47	319.86	320.02	320.15	320.18	315.12	317.75	321.04	321.21	320.16	320.20	320.22	320.22
**14**	423.21	424.40	410.52	412.17	412.41	412.57	412.35	412.39	424.90	423.25	417.16	417.34	412.59	412.70	412.56	412.57
**15**	243.30	239.02	223.59	221.74	222.55	222.68	222.96	222.98	237.09	230.85	227.95	225.26	223.18	223.10	223.01	223.01
**16**	432.75	429.31	423.09	422.97	424.27	424.39	424.35	424.37	431.82	424.53	427.34	426.86	424.37	424.38	424.41	424.41
**17**	471.64	473.57	466.41	467.35	469.45	469.44	469.30	469.32	467.33	470.86	471.95	472.15	469.30	469.31	469.34	469.33
**18**	386.73	385.28	374.27	376.03	376.99	377.24	377.27	377.29	382.67	377.91	378.62	379.38	377.27	377.30	377.33	377.33
**19**	362.75	358.10	346.29	347.92	348.93	349.16	349.25	349.23	358.23	349.95	350.71	351.13	349.25	349.26	349.29	349.30
**20**	228.62	375.46	364.23	363.45	364.32	364.16	364.39	364.44	217.28	213.82	367.30	366.28	364.49	364.40	364.41	364.39

Table S4. Continue.

Mol.	Basis sets					
	6-31G(d,p)	6-311(d,p)	6-31++G(d,p)	6-311++G(d,p)	6-311++G(2d,2p)	6-311++G(3df,3pd)
**1**	294.42	278.54	287.35	274.61	275.22	277.49
**2**	472.39	471.47	471.50	473.46	472.90	473.15
**3**	279.33	259.96	267.38	250.41	251.83	255.56
**4**	334.19	319.20	324.20	313.61	314.10	315.76
**5**	417.71	414.64	413.54	411.20	410.59	411.63
**6**	367.86	361.95	366.29	357.94	357.72	359.34
**7**	318.08	307.13	322.36	310.71	310.24	311.68
**8**	391.75	387.12	394.01	386.83	386.46	388.10
**9**	289.11	274.84	289.24	275.71	275.11	276.98
**10**	374.57	370.58	378.74	372.70	372.95	374.72
**11**	421.40	415.91	420.21	411.95	412.92	413.70
**12**	237.07	219.65	232.68	215.30	216.01	219.67
**13**	331.23	313.01	331.85	318.59	321.17	322.06
**14**	419.24	429.01	428.40	428.96	419.08	415.82
**15**	241.49	234.67	246.16	238.90	231.19	228.44
**16**	440.88	434.71	427.31	424.03	424.11	424.31
**17**	467.43	470.37	472.39	466.38	467.01	469.81
**18**	395.89	384.46	384.86	377.36	377.75	378.79
**19**	375.72	359.59	360.27	349.12	350.27	350.86
**20**	375.88	368.97	377.01	366.37	364.87	365.83

Table S5. ^{19}F NMR shielding constants (in ppm) of molecules **1-20** calculated within the GIAO-CCSD method with different basis set schemes, in the gas phase.

Mol.	Basis sets (in the case of **A/B**: **A** – basis set on fluorine atoms, **B** – basis set on the rest of atoms)					
	pcS-2/pcS-1	pcS-2	pcS-3/pcS-1	pcS-3/pcS-2	pcS-3	pcS-4/pcS-3
**1**	313.06	291.68	298.47	292.95	290.60	290.97
**2**	482.81	474.16	484.90	481.75	481.63	482.36
**3**	305.01	273.61	285.57	273.95	268.69	268.95
**4**	352.05	325.20	342.05	330.85	327.18	327.84
**5**	438.67	416.34	436.04	424.91	421.65	422.47
**6**	391.52	372.17	392.45	382.16	379.74	380.70
**7**	350.92	327.39	354.60	338.67	333.28	334.32
**8**	426.91	401.40	423.18	412.39	409.20	410.00
**9**	320.60	296.08	316.87	303.08	297.72	298.37
**10**	397.12	387.30	401.26	396.17	392.86	393.59
**11**	406.26	415.48	413.84	416.51	418.94	419.13
**12**	274.84	246.43	262.24	250.51	242.40	242.95
**13**	349.36	337.27	343.02	338.73	335.53	336.10
**14**	460.51	430.13	461.56	434.38	424.20	424.61
**15**	295.80	267.14	277.87	257.04	245.53	245.12
**16**	461.22	433.87	456.11	443.09	438.25	438.87
**17**	488.53	474.76	488.69	493.41	487.99	488.71
**18**	415.47	383.76	410.33	393.05	388.18	389.07
**19**	391.29	355.31	385.24	364.93	357.86	358.26
**20**	414.79	375.45	418.17	394.10	386.49	387.08

Table S6. ^{19}F NMR shielding constants (in ppm) of molecules **1-20** calculated within the GIAO-DFT(BHandHLYP) method with different basis set schemes, in the gas phase.

Mol.	Basis sets (in the case of **A**/**B**: **A** – basis set on fluorine atoms, **B** – basis set on the rest of atoms)					
	pcS-2/pcS-1	pcS-2	pcS-3/pcS-1	pcS-3/pcS-2	pcS-3	pcS-4/pcS-3
**1**	277.80	275.46	275.59	274.67	274.52	274.52
**2**	474.45	475.12	473.36	473.85	474.20	474.29
**3**	257.61	253.23	254.27	252.26	251.83	251.83
**4**	316.67	314.34	314.59	313.28	313.18	313.20
**5**	412.30	412.18	410.86	410.63	410.74	410.86
**6**	360.15	359.24	359.82	357.78	357.91	357.95
**7**	311.35	309.37	311.83	309.21	309.18	309.22
**8**	389.10	389.01	389.19	387.44	387.31	387.35
**9**	277.05	274.77	275.94	273.88	273.63	273.64
**10**	373.04	373.12	373.16	371.97	372.10	372.12
**11**	412.06	413.67	411.89	412.34	412.42	412.54
**12**	218.90	215.70	217.22	215.42	214.98	214.98
**13**	322.25	322.25	322.25	322.25	322.25	322.25
**14**	428.15	417.16	425.15	415.67	412.59	412.72
**15**	239.13	239.13	239.13	239.13	239.13	239.13
**16**	428.87	427.34	425.39	424.67	424.37	424.41
**17**	471.47	471.94	470.45	469.41	469.28	469.32
**18**	381.09	378.62	379.36	377.35	377.27	377.32
**19**	353.19	350.71	351.28	349.39	349.25	349.29
**20**	371.63	367.25	370.00	365.39	364.39	364.49

Table S7. ^{19}F NMR shielding constants (in ppm) of molecules **1, 2, 4, 9, 11-13, 15, 21-25** calculated within the GIAO-CCSD method with different basis set schemes, in the gas phase.

Mol.	Basis sets (in the case of **A**/**B**: **A** – basis set on fluorine atoms, **B** – basis set on the rest of atoms)					
	pcS-2/pcS-1	pcS-2	pcS-3/pcS-1	pcS-3/pcS-2	pcS-3	pcS-4/pcS-3
**1**	313.06	291.68	298.47	292.95	290.60	290.97
**2**	482.81	474.16	484.90	481.75	481.63	482.36
**4**	352.05	325.20	342.05	330.85	327.18	327.84
**9**	320.60	296.08	316.87	303.08	297.72	298.37
**11**	406.26	415.48	413.84	416.51	418.94	419.13
**12**	274.84	246.43	262.24	250.51	242.40	242.95
**13**	349.36	337.27	343.02	338.73	335.53	336.10
**15**	295.80	267.14	277.87	257.04	245.53	245.12
**21 (=CHF)**	443.05	418.82	435.81	427.71	425.44	425.99
**21 (F trans to H)**	335.69	314.10	328.50	318.78	315.39	315.57
**21 (F cis to H)**	365.85	340.50	358.12	348.48	345.07	345.32
**22**	373.34	352.99	365.19	358.49	356.35	356.75
**23**	305.10	276.96	289.20	277.74	271.69	271.64
**24**	303.03	279.87	282.16	277.35	274.69	274.50
**25**	242.87	208.72	227.24	205.21	166.23	189.60

Table S8. Vibrational corrections to ^{19}F NMR shielding constants (in ppm) of molecules **1, 2, 4, 9, 11-13, 15, 21-25** calculated within the GIAO-DFT(BHandHLYP) method with the pcS-2 basis set, in the gas phase.

Molecule														
**1**	**2**	**4**	**9**	**11**	**12**	**13**	**15**	**21 (=CHF)**	**21 (F trans to H**	**21 (F cis to H)**	**22**	**23**	**24**	**24**
-9.05	-10.97	-12.77	-9.03	-11.04	-7.73	-3.22	-4.25	-8.96	-6.07	-4.45	-11.70	-9.71	-9.25	-9.74
